# Revision of *Cerinomyces* (*Dacrymycetes*, *Basidiomycota*) with notes on morphologically and historically related taxa

**DOI:** 10.1016/j.simyco.2021.100117

**Published:** 2021-12-01

**Authors:** A. Savchenko, J.C. Zamora, T. Shirouzu, V. Spirin, V. Malysheva, U. Kõljalg, O. Miettinen

**Affiliations:** 1 Institute of Ecology and Earth Sciences, University of Tartu, 14a Ravila, 50411, Tartu, Estonia; 2 Museum of Evolution, Uppsala University, Norbyvägen 16, SE-75236, Uppsala, Sweden; 3 Graduate School of Bioresources, Mie University, 1577 Kurima-machiya, Tsu, Mie, 514-8507, Japan; 4 Finnish Museum of Natural History, University of Helsinki, P.O. Box 7, FI-00014, Helsinki, Finland; 5 Komarov Botanical Institute, Russian Academy of Sciences, Prof. Popova str. 2, RU-197376, St. Petersburg, Russia; 6 Natural History Museum, University of Tartu, Vanemuise 46, 51003, Tartu, Estonia

**Keywords:** *Ceracea*, *Cerinomycetaceae*, Corticioid, New taxa, Phylogeny, Taxonomy, Type studies, Typification, *Cerinomyces aeneus* A. Savchenko, Miettinen & J.C. Zamora, *C. atrans* A. Savchenko, *C. borealis* Miettinen, Spirin & A. Savchenko, *C. brevisetus* Chikowski, Alvarenga & A. Savchenko, *C. concretus* A. Savchenko, *C. creber* J.C. Zamora, A. Savchenko, Trichies & Olariaga, *C. enterolaxus* Shirouzu & A. Savchenko, *C. favonius* Spirin, Miettinen & A. Savchenko, *C. fugax* A. Savchenko, *C. hesperidis* A. Savchenko, *C. inermis* A. Savchenko, *C. lipoferus* J.C. Zamora & A. Savchenko, *C. nepalensis* A. Savchenko, *C. neuhoffii* J.C. Zamora & A. Savchenko, *C. paulistanus* A. Savchenko, *C. pinguis* A. Savchenko, *C. ramosissimus* A. Savchenko, *C. tristis* Miettinen & A. Savchenko, *C. verecundus* A. Savchenko, *C. volaticus* A. Savchenko, V. Malysheva & J.C. Zamora, *Dacrymyces burdsallii* A. Savchenko, *D. grandii* A. Savchenko & Miettinen, *D. sobrius* A. Savchenko, *D. venustus* A. Savchenko, *Cerinomyces cokeri* (McNabb) A. Savchenko & J.C. Zamora, *C. enatus* (Berk. & M.A. Curtis) A. Savchenko, *C. tortus* (Willd.) Miettinen, J.C. Zamora & A. Savchenko, *Dacrymyces ceraceus* (Ginns) A. Savchenko, *D. cereus* (Rick) A. Savchenko, *D. grandinioides* (McNabb) A. Savchenko, *D. lagerheimii* (Pat.) A. Savchenko, *D. pengii* (B. Liu & L. Fan) A. Savchenko, *D. pulchrus* (Lowy) A. Savchenko, *Ceracea aureofulva* Bres., *Ce. cerea* Rick, *D. confluens* P. Karst., *Tremella enata* Berk. & M.A. Curtis, *Tremella torta* Willd

## Abstract

*Cerinomyces (Dacrymycetes*, *Basidiomycota*) is a genus traditionally defined by corticioid basidiocarps, in contrast to the rest of the class, which is characterized by gelatinous ones. In the traditional circumscription the genus is polyphyletic, and the monotypic family *Cerinomycetaceae* is paraphyletic. Aiming for a more concise delimitation, we revise *Cerinomyces s.l.* with a novel phylogeny based on sequences of nrDNA (SSU, ITS, LSU) and protein-coding genes (RPB1, RPB2, TEF1-α)*.* We establish that monophyletic *Cerinomyces s.s.* is best characterized not by the corticioid morphology, but by a combination of traits: hyphal clamps, predominantly aseptate thin-walled basidiospores, and low content of carotenoid pigments. In our updated definition, *Cerinomyces s.s.* encompasses five well-supported phylogenetic clades divided into two morphological groups: (i-iii) taxa with arid corticioid basidiocarps, including the generic type *C. pallidus*; and (iv-v) newly introduced members with gelatinous basidiocarps, like *Dacrymyces enatus* and *D. tortus*. The remaining corticioid species of *Cerinomyces s.l.* are morphologically distinct and belong to the *Dacrymycetaceae*: our analysis places the carotenoid-rich *Cerinomyces canadensis* close to *Femsjonia*, and we transfer the clamps-lacking *C. grandinioides* group to *Dacrymyces.* In addition, we address genera related to *Cerinomyces s.l.* historically and morphologically, such as *Ceracea*, *Dacryonaema* and *Unilacryma*. Overall, we describe twenty-four new species and propose nine new combinations in both *Cerinomycetaceae* and *Dacrymycetaceae*.

## Introduction

**Fig. 1:**
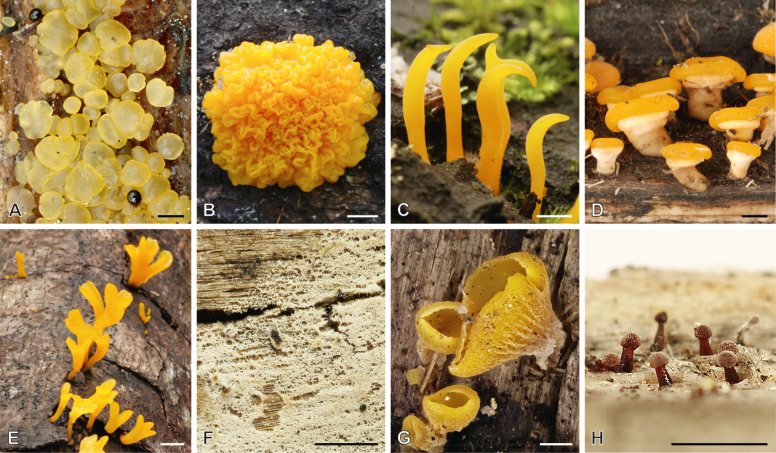
Some of the most common basidiocarp morphotypes in *Dacrymycetes*. **A.** Pustulate *Dacrymyces adpressus* (H6012680). **B.** Cerebriform *D. chrysospermus* (TU135035). **C.** Cylindrical and dendroid *Calocera furcata* (H6012626). **D.** Capitate *Ditiola radicata* (H6012689). **E.** Spathulate *Dacryopinax* sp. (H:Miettinen 13068). **F.** Corticioid *Cerinomyces borealis* (GB-0071203). **G.** Cupulate *Guepiniopsis buccina* (CWU(MYC)7014). **H.** Synnematous *Dacryonaema rufum* (H:Poelt, Fungi 242). Scale bars: A, C, D, F, G, H ≈ 1 mm; B, E ≈ 5 mm.

The *Dacrymycetes* is a phylogenetically and morphologically well-established class of basidia-bearing fungi, inhabiting dead wood and causing brown rot (Oberwinkler 2014, Floudas *et al.* 2015, Nagy *et al.* 2016). The class is relatively small, and includes two orders (*Dacrymycetales* and *Unilacrymales*), four families (*Cerinomycetaceae* Jülich*, Dacrymycetaceae* J. Schröt., *Dacryonaemataceae* J.C. Zamora & Ekman, and *Unilacrymaceae* Shirouzu, Tokum. & Oberw.), more than 10 generally accepted genera, and ca. 400 published species names. Many of these names are taxonomic synonyms, and the true number of described species is at least 120. Members of the class can be readily distinguished from other basidiomycetes by their bisterigmate Y-shaped basidia, with the single exception of *Unilacryma unispora*, which has unisterigmate basidia (Wells 1994, Shirouzu *et al.* 2013). On an ultrastructural level, dacrymycetes are characterized by dolipore septa with imperforate parenthesomes, or rarely with a single pore (Maekawa 1987, Oberwinkler 1994, Shirouzu *et al.* 2013). In terms of macromorphology, the class is dominated by so-called “jelly fungi” with pustulate-pulvinate, cupulate, dendroid, and spathulate basidiocarps, coloured with carotenoids in different tints of yellow and orange (Fig. 1, Goodwin 1953, Czeczuga 1980, Zamora & Ekman 2020). In addition to the soft-gelatinous species with prominent basidiocarps, the class also includes members with corticioid basidiocarps of arid or waxy-gelatinous consistency, attached to the substrate with an applanate subiculum. These fungi are traditionally classified in the genus *Cerinomyces* Martin. In contrast to the gelatinous dacrymycetes, basidiocarps of the traditional *Cerinomyces* species do not noticeably change in shape or swell in transition between dry and moist conditions. Most important, corticioid-resupinate morphotype was thought to be a definitive character of *Cerinomyces*. In result of such prioritization, the genus has accumulated a large variation in microscopic features over time, and its scope has become ever broader.

**Fig. 2:**
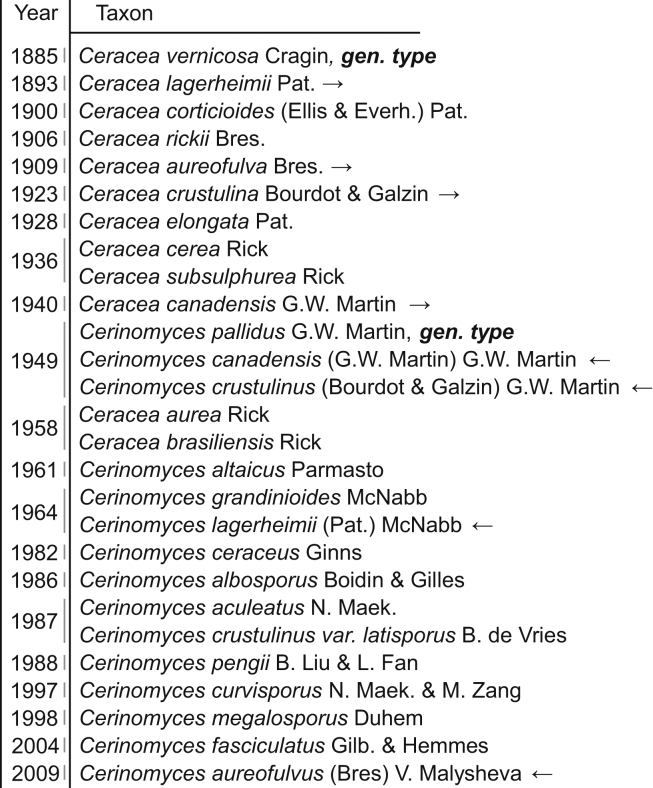
*Ceracea* and *Cerinomyces* species and varieties published and combined prior to the given work. Arrows indicate *Ceracea* species that were transferred to *Cerinomyces*.

The name *Cerinomyces* initially appeared as a typographic error in “*Ceriomyces*” in the index of the 10^th^ volume of the “Handwörterbuch der Naturwissenschaften” encyclopedia, p. 1085 (Korschelt *et al.* 1915). However, this misspelling had no formal nomenclatural value, and when Martin (1949) described *Cerinomyces* to accommodate some fungi formerly classified in *Ceracea* Cragin, his genus was published in a valid way. (We hereinafter abbreviate *Ceracea* as “*Ce.*” and *Cerinomyces* as “*C.*”). The nomenclature of *Cerinomyces* prior to Martin (1949) is tied to *Ceracea*. Cragin (1885) originally introduced *Ceracea* as a monotypic genus for an applanate brown fungus with “mostly bifurcate filaments”, believed by subsequent researchers to be a reference to the Y-shaped dacrymycete basidia, bearing elliptical aseptate spores at the apices of these basidia. The substrate of the genus type, *Ce. vernicosa* Cragin, was a polypore fungus, but in other details the vague description suited resupinate dacrymycetes. In the same year, the description was reproduced in a literature synopsis, which is sometimes erroneously cited as the genus’ original publication (Kellerman 1885). Soon after, Patouillard described from Ecuador a new species *Ce. lagerheimii*, and supplied it with imagery showing doubtlessly dacrymycete microstructures (Patouillard & Lagerheim 1893). In the beginning of the twentieth century, more species and combinations were assigned to *Ceracea* (Fig. 2): *Ce. corticioides* (Ellis & Everh.) Pat., *Ce. rickii* Bres., *Ce. aureofulva* Bres., *Ce. crustulina* Bourdot et Galzin, *Ce. elongata* Pat, *Ce. cerea* Rick, and *Ce. subsulphurea* Rick.

Lloyd reinterpreted *Ceracea* with a statement that “no doubt it is the same as *Arrhytidia*” — a genus whose type *A. flava* Berk. & M.A. Curtis is strongly similar to *Femsjonia* Fries (Fries 1851, Lloyd & Stevenson 1921). Coker (1928) agreed with Lloyd and synonymized *A. flava*, *Ceracea aureofulva* Bres.*, Dacrymyces corticioides* Ellis & Everh. and *D. involutus* Schw. as *A. involuta* (Schwein.) Coker (orth. var. *involutus*). In doing so, they started a tradition of naming effused dacrymycetes as *Arrhytidia* Berk. & M.A. Curtis. Wide use of the umbrella name *A. involuta* added further confusion, because it is a close relative if not a synonym of *D. capitatus* Schwein., a typical gelatinous *Dacrymyces* species (McNabb 1973).

Brasfield (1938) mentioned that “*Ceracea auct.* not Cragin” is a synonym to *Arrhytidia*. Later he changed his mind and purported that *Ceracea* is a correct name for species with resupinate basidiocarps without a distinct “root” in substrate. Since the type of *Ce. vernicosa* was assumed to be lost, he intended to typify *Ceracea* by *Ce. lagerheimii* (Brasfield 1940)*.* Martin (1940) accepted Brasfield’s concept of the genus and described a new species *Ce. canadensis*. Luckily, the original material of *Ce. vernicosa* was found, and Martin (1949) revealed it to represent an anamorphic ascomycete fungus, possibly a parasite of poly-pores. Thus, the name became unavailable for corticioid dacrymycetes. To encompass the latter, Martin introduced the genus *Cerinomyces* consisting of *C. pallidus* as the type, and new combinations *C. crustulinus* and *C. canadensis*. The last species of *Ceracea*, *Ce. aurea* and *Ce. brasiliensis*, appeared in a post-mortem publication of Rick (1958).

In the following years, the taxonomic position of *Cerinomyces* was a matter of discordance. Donk (1956, 1957), Eriksson (1958) and Eriksson & Ryvarden (1973) suggested the genus is affined with corticioid fungi, while others treated it as a member of dacrymycetes (Martin 1957, Kennedy 1958a, Parmasto 1961). Within the most comprehensive revision of dacrymycetes to date, McNabb (1964) accepted the latter opinion, followed by the rest of the community. For example, Donk (1966) had already cited *Cerinomyces* in the *Dacrymycetales*. In the same work, McNabb described *C. grandinioides* and combined *C. lagerheimii* — a species that differs from the rest of the genus by its absence of clamps.

By the end of the twentieth century, the genus was expanded with a number of new taxa. *Cerinomyces ceraceus* Ginns joined a morphogroup of clampless *Cerinomyces* species. Boidin and Gilles introduced *C. albosporus*, which was later accompanied with macromorphologically similar *C. aculeatus* N. Maek., *C. curvisporus* N. Maek. & M. Zang, and *C. fasciculatus* Gilb. & Hemmes. A few more taxa with less certain relations were described as *C. crustulinus var. latisporus* B. de Vries (the first strictly gelatinous taxon in the genus)*, C. pengii* Liu & Fan, and *C. megalosporus* Duhem.

Shirouzu and co-authors pioneered the phylogenetic studies of the class, and in an ongoing series of works demonstrated that most genera in the class are polyphyletic, and *Cerinomyces* is not an exception (Shirouzu *et al.* 2007, 2009, 2013, 2016, 2020). They showed that species with corticioid arid basidiocarps, three-septate basidiospores and clampless septa, like *C. lagerheimii*, *C. ceraceus*, and *C. grandinioides*, belong to the core *Dacrymycetaceae* as a sister clade to some typical *Dacrymyces* and *Guepiniopsis* Pat. species. On the other hand, species like *Dacrymyces punctiformis* Neuhoff with pustulate gelatinous basidiocarps, aseptate basidiospores and clamped hyphae clustered in the *Cerinomycetaceae* (Shirouzu *et al.* 2009, 2013). Aside from these shifts, the *Cerinomycetaceae* appeared to be a well-supported clade in most phylogenetic analyses. In a recent work, Zamora & Ekman (2020) confirmed the earlier results and clarified a mean stem age for the *Cerinomycetaceae*, estimated at 197 million years ago. They also pointed out that the studied species did not have conspicuous carotenoid contents, and young spores in the genus are binucleate, in contrast to uninucleate ones in the rest of dacrymycetes (see also Yen 1947). Finally, a continuing production of dacrymycete genomes including species of *Cerinomyces s.l.* in the 1 000 Fungal Genomes Project promises further refinement of the group phylogeny (Grigoriev *et al.* 2014).

The aims of this paper are: (i) to assess all existing species of *Cerinomyces s.l.* and describe new ones; (ii) to revise the *Cerinomycetaceae* as a family consisting of *Cerinomyces s.s.*; (iii) to find practical nomenclatural solutions for the species excluded from *Cerinomyces s.s.*; and (iv) to establish connections between phylogenetic groups and morphological characters whenever possible.

## Materials and methods

### Morphological study

Specimens were obtained from the herbaria ARIZ, BPI, CFMR, CWU, EA, FH, GB, H, HMAS, ILLS, K, KAS, L, LE, LSUM, NCSLG, NCU, NY, O, PC, PDD, PRM, S, TAAM, TNM, TNS, TRTC, TU, UBC, UPS, URM, and personal herbarium of R. Enzlin (see specimen index in Supplementary Table S1). Herbaria acronyms follow Index Herbariorum (http://sweetgum.nybg.org/science/ih/). Collector’s numbers are shown without a collector’s name abbreviation. Studied type specimens are accompanied with exclamation mark (!), and these specimens are not duplicated under “Specimens examined”. Descriptions are based primarily on sequenced specimens marked with an asterisk (∗), and specimens without sequences are incorporated only in the absence of sequenced ones or when their morphology agrees well with the adopted species concept. Detailed information on specimens, high-resolution macro photographs, and scanned notes and labels are available under CC BY 4.0 license via the PlutoF platform (https://plutof.ut.ee, Abarenkov *et al.* 2010).

**Fig. 3:**
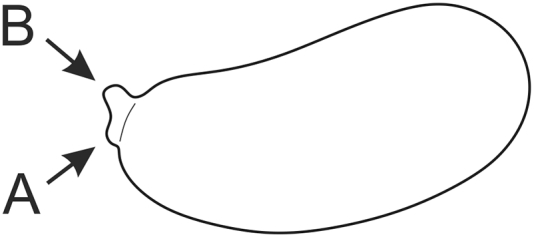
Basidiospore apiculus structure. A = hilum, B = hilar appendix.

Microscopic studies were performed with Leica DMLB, Leica DM1000 LED and Nikon Eclipse 80i microscopes. For slide preparation small parts of basidiocarps were moisturized with tap water, then cut with a razor blade and placed for a short time in a small drop of water; excess of water was removed with filter paper before further dying. The routine mountant used for measurements and drawings was Cotton Blue (CB): 0.1 mg aniline blue (Merck 1275) dissolved in 60 g of pure lactic acid. After applying a cover-glass, the slide was heated without reaching the boiling point, then the preparation squashed by tapping on the cover-glass, and excess of CB removed with filter paper. In cases when CB was not suitable for measurements, 1 % KOH with addition of water solution of Congo Red was used instead. Whenever spore measurements in KOH are reported, it is mentioned. Illustrations were produced from microscopic slides using either a drawing tube at ×1 000 magnification (×2 000 for spore drawings) or rarely from integrated camera photos; later vectorized with Wacom DTK-2700 in CorelDRAW 2017. Measurements were done using ×1 000 magnification, oil immersion, and phase contrast illumination; eyepiece scale bar with 1 μm grid was used, and dimensions were estimated with a subjectively defined accuracy of 0.1 μm; when working with Nikon hardware, spores were photographed and measured in NIS-Elements BR < v. 5.20.00. Spore statistics were produced in LibreOffice Calc ≤ v. 6.0.6.2. When preparing summaries, individual measured spores were omitted only when considered immature or overgrown; not more than five spores from original measurements were excluded per taxon. The following abbreviations are used in descriptions: L for mean spore length, W for mean spore width, Q for L/W ratio, Q’ for variation of length to width ratio of individual basidiospores. To show variation in basidiospore dimensions, 5 % of measurements from each end of the range are excluded and given in parentheses. In case of identical values, parts in parentheses are omitted. For the types and representative specimens at least 30 randomly selected mature basidiospores and well-developed basidia were measured when possible; a total number of measured structures against a number of studied specimens is shown as “n = 30/1”. By default, spore walls thickness measurements were obtained from outer walls, not septal, which are often thicker. Following Ingold (1983), we distinguish parts of two-lobed apiculus (or hilar apparatus) in basidiospores of dacrymycetes as: (A) hilum itself — the point of attachment to basidia; and (B) more prominent hilar appendix (Fig. 3). Herewith, length of apiculus is not added to the spore length. Terms “basidia” and “sterigmata” are used for parts below and above the bifurcation point, respectively. The widths of basidia were measured immediately below the bases of sterigmata. Sterile elements in hymenium are referred here as “hyphidia”, instead of traditional “dikaryophyses” (see Discussion). Raw morphometric data for the studied specimens are provided in the Supplementary Table S2. Taxonomic novelties were deposited in MycoBank (Crous *et al.* 2004).

### DNA extraction, PCR, and sequencing

Protocols for most of UPS and TNS herbaria specimens follow respectively Zamora & Ekman (2020) and Shirouzu *et al.* (2016); most other materials were processed as indicated below. DNA from the fresh specimens was routinely extracted in 100 μL of “10× reaction buffer B” (0.8 M Tris-HCl pH 8.8–8.9, 0.2 M (NH_4_)_2_SO_4_, 0.2 % w/v Tween-20; Solis Biodyne, Estonia) including 2.5 μL of proteinase K (20 mg/mL; Thermo Fisher Scientific, Waltham, Massachusetts, USA), incubated at 56 °C overnight and deactivated in 98 °C for 15 min.; supernatant then extracted and stored at -80 °C. For older specimens and types the High Pure PCR template preparation kit (Roche Applied Science, Penzberg, Germany) was used following the protocol of manufacturer. Polymerase chain reaction (PCR) was performed with the following primers; forward and reverse ones are separated by slash. The whole ITS region with a part of LSU: ITSOF (ACTTGGTCATTTAGAGGAAGT, Tedersoo *et al.* 2008) / LR5 (TCCTGAGGGAAACTTCG, Hopple & Vilgalys 1994) or LB-W (CTTTTCATCTTTCCCTCACGG, Tedersoo *et al.* 2008), or ITS4 (TCCTCCGCTTATTGATATGC, White *et al.* 1990); ITS1: ITFOF / ITS2 (GCTGCGTTCTTCATCGATGC, White *et al.* 1990); ITS2: 58A1F (GCATCGATGAAGAACGC, Martin & Rygiewicz 2005) / ITS4; LSU, both by Hopple & Vilgalys (1994): LR0R (ACCCGCTGAACTTAAGC) / LR7 (TACTACCACCAAGATCT); SSU, both modified by L. Tedersoo after White *et al.* (1990): NS1a (TCTCAAAGAYTAAGCCATGC) / NS8a (CCTCTAAATGACCRAGTTTG); TEF1-α, both by Rehner & Buckley (2005): EF1-983F (GCYCCYGGHCAYCGTGAYTTYAT) / EF1-1567R (ATGACACCRACRGCRACRGTYTG); RPB1: RPB1-Af (GARTGYCCDGGDCAYTTYGG, Stiller & Hall 1997) / RPB1-Cr (CCNGCDATNTCRTTRTCCATRTA, Matheny *et al.* 2002); RPB2, both by Liu *et al.* (1999): fRPB2-5F (GAYGAYMGWGATCAYTTYGG) / fRPB2-7cR (CCCATRGCTTGYTTRCCCAT). Routine PCR load was: 5 μL of HOT FIREPol Blend Master Mix (with 10 mM MgCl_2_; Solis BioDyne), 0.5 μL of each primer and 1–3 μL of DNA extract in 0.1× concentration, filled up to the total 25 μL volume with Milli-Q water. Amount and concentration of DNA extracts were tweaked to troubleshoot PCR problems; in case of Roche kit extractions and in all RPB1 and RPB2 amplifications we used DNA extracts in 1× concentration. SSU, ITS, LSU were amplified with the following PCR cycle: pre-denaturation at 95 °C for 15 min; 35 cycles: denaturation at 95 °C for 30 s, annealing at 55 °C for 30 s, elongation at 72 °C for 1 min; final elongation at 72 °C for 10 min. PCR cycle for TEF1α, RPB1, RPB2, designed by Zheng Wang, via the PolyPeet project: pre-denaturation at 94 °C for 15 min; 9 cycles: denaturation at 94 °C for 40 s, annealing at 60 °C −1 °C per cycle for 40 s, elongation at 72 °C for 2 min; 37 cycles: 94 °C for 45 s, 53 °C for 90 s, 72 °C for 2 min; final elongation at 72 °C for 10 min. Sequencing was performed by Macrogen Europe (Amsterdam, Netherlands) using the primers listed above except ITS5 (GGAAGTAAAAGTCGTAACAAGG, White *et al.* 1990) for ITSOF products and CTB6 (GCATATCAATAAGCGGAGG, Garbelotto *et al.* 1997) for LR0R products. The resulting sequences are available in GenBank and the accession numbers are listed in the Table 1. ITS sequences were also parsed by UNITE (https://unite.ut.ee/, Nilsson *et al.* 2018) and, if passed automated quality check, assigned to Species Hypotheses (SHs, Kõljalg *et al.* 2020). The list of taxa, numbers of sequences used in SH building and relevant SH codes at different similarity thresholds are presented in the Supplementary Table S3.

**Table 1:** Specimens and corresponding sequence accession numbers used in this study. Accession numbers of newly generated sequences are indicated in bold.

Species	Specimen ID	Country	SSU	ITS	LSU	TEF1-α	RPB1	RPB2	Reference
*Calocera cornea*	CWU(MYC)6922	Ukraine	**MW158985**	**MW191969**	**MW159089**	**MW130299**	**MW130381**	**MW130423**	this study
	UPS:F-940774	Sweden	MN593442	MN595626	MN595626	MN580325	MN580225	MN580265	Zamora & Ekman (2020)
	UPS:F-940775	Sweden	MN593443	MN595627	MN595627	MN580326	MN580226	MN580266	Zamora & Ekman (2020)
*Ca. furcata*	H:Spirin 10949	Russia	–	**MW191975**	**MW159088**	**MW130298**	–	–	this study
	TU135016	Estonia	**MW158984**	**MW191958**	**MW159087**	**MW130297**	**MW130380**	**MW130422**	this study
*Ca. glossoides*	CWU(MYC)6247	Ukraine	**MW159005**	**MW191968**	**MW159084**	**MW130307**	**MW130388**	**MW130428**	this study
*Ca. viscosa*	CWU(MYC)6937	Ukraine	**MW158986**	**MW191970**	**MW159090**	**MW130302**	**MW130382**	**MW130424**	this study
	UPS:F-940773	Sweden	MN593444	MN595628	MN595628	MN580327	MN580227	MN580267	Zamora & Ekman (2020)
*Cerinomyces aculeatus holotype*	TUMH61942 (TUFC50098)	Japan	**MW158956**	**MW191955**	**MW159053**	**MW130323**	–	–	this study
	MAFF 247114	Japan	–	–	LC492249	–	–	–	Shirouzu *et al.* (2020)
	TNS-F-15706	Japan	AB712482	AB712440	AB299050	–	–	AB712524	Shirouzu *et al.* (2007, 2013)
	TU135069	Russia	**MW158955**	**MW191883**	**MW159044**	–	–	–	this study
	TU135070	Russia	**MW158954**	**MW191884**	**MW159045**	**MW130322**	**MW130375**	**MW130410**	this study
*C. aff. aculeatus 1*	TNM-F16565	Taiwan	–	–	AY600248	–	–	–	Kirschner & Yang (2005)
*C. aeneus holotype*	H7009708	Ukraine	–	**MW191920**	–	–	–	–	this study
	H:Miettinen 15065.2	Finland	–	**MW191917**	–	–	–	–	this study
	O146179	Norway	–	**MW191918**	–	–	–	–	this study
	PRM929891	Czech Republic	–	**MW192003**	**MW192003**	**MW130354**	–	**MW130444**	this study
	PRM934335	Czech Republic	–	**MW192004**	**MW192004**	**MW130355**	–	**MW130445**	this study
	TU135065	Ukraine	**MW158966**	**MW191919**	**MW159054**	**MW130337**	**MW130367**	–	this study
	UPS:F-560919	Sweden	**MW159012**	**MW191987**	**MW191987**	–	–	–	this study
	UPS:F-946499	Sweden	**MW159013**	**MW191988**	**MW191988**	–	–	–	this study
*C. albosporus holotype*	LY11692	Réunion	–	**MW191885**	–	**MW130321**	–	–	this study
*C. atrans holotype*	GB-0071218	Canada	–	**MW191928**	–	**MW130330**	–	–	this study
	GB-0071217	Canada	–	**MW191929**	–	**MW130331**	–	–	this study
	GB-0180499	Canada	**MW159009**	**MW191984**	**MW191984**	**MW130351**	–	–	this study
	TUFC30545	?	AB712485	AB712443	AB712423	–	–	AB712527	Shirouzu *et al.* (2013)
*C. borealis holotype*	O160848	Norway	–	**MW191890**	**MW159042**	–	–	–	this study
	H:Miettinen 14094	Finland	**MW158982**	**MW191891**	**MW159037**	**MW130343**	**MW130369**	**MW130414**	this study
	H:Miettinen 21156.1	Finland	**MW158963**	**MW191889**	**MW159038**	**MW130344**	**MW130371**	**MW130411**	this study
	H:Spirin 10443	Russia	**MW158965**	**MW191888**	**MW159036**	**MW130345**	**MW130372**	**MW130413**	this study
	H6055125	Finland	**MW158964**	**MW191892**	**MW159039**	**MW130346**	**MW130370**	**MW130412**	this study
	O101812	Norway	–	**MW191893**	–	–	–	–	this study
*C. brevisetus holotype*	URM:Chikowski 1544	Brazil	**MW158957**	**MW191886**	**MW159046**	**MW130320**	–	–	this study
*"C." canadensis*	H:Spirin 8468	USA	**MW158983**	**MW191945**	**MW159069**	**MW130349**	**MW130376**	**MW130419**	this study
	TAAM007082	Russia	–	**MW191947**	–	–	–	–	this study
	TAAM061880	Russia	–	**MW191946**	–	–	–	–	this study
*C. cokeri*	TU135089	Canada	**MW159008**	**MW191983**	**MW191983**	**MW130350**	**MW130393**	–	this study
*C. concretus holotype*	O:F-919450	Colombia	–	**MW191933**	–	–	–	–	this study
*C. creber holotype*	UPS:F-946512	Spain	**MW159010**	**MW191985**	**MW191985**	**MW130352**	**MW130394**	–	this study
	H:Trichies 07077	France	–	**MW191927**	–	–	–	–	this study
	UPS:F-946506	Spain	**MW159018**	**MW191993**	**MW191993**	–	**MW130399**	**MW130435**	this study
	UPS:F-946507	Spain	**MW159019**	**MW191994**	**MW191994**	–	**MW130400**	**MW130436**	this study
	UPS:F-979574	Spain	–	**MZ147629**	**MZ147629**	–	–	–	this study
*C. aff. crustulinus 1*	UPS:F-958851	Spain	**MW159011**	**MW191986**	**MW191986**	**MW130353**	**MW130395**	**MW130431**	this study
*C. enatus*	CFMR:HHB-671	USA	–	**MW191977**	–	–	–	–	this study
	CFMR:HHB-7334	USA	–	**MW191978**	–	–	–	–	this study
	H:Spirin 10764	Russia	**MW158980**	**MW191937**	**MW159033**	**MW130338**	–	–	this study
	H:Spirin 7774	Russia	–	**MW191939**	**MW159034**	**MW130339**	–	–	this study
	H:Spirin 7780	Russia	**MW158981**	**MW191938**	**MW159035**	–	–	–	this study
	OTU_263	Japan	–	–	LC492284	–	–	–	Shirouzu *et al.* (2020)
	TNS-F-21034	Japan	AB712483	AB712441	AB472696	–	–	AB712525	Shirouzu *et al.* (2009, 2013)
	TNS-F-21035	Japan	–	–	AB472697	–	–	–	Shirouzu *et al.* (2009)
	TNS-F-21036	Japan	–	–	AB472698	–	–	–	Shirouzu *et al.* (2009)
	TNS-F-21037	Japan	**LC585259**	**LC585257**	AB472699	–	–	–	this study, Shirouzu *et al.* (2009)
	TNS-F-21064	Japan	**LC585260**	–	AB472724	–	–	–	this study, Shirouzu *et al.* (2009)
	TNS-F-61320	Japan	–	**LC585250**	LC003923	–	–	–	this study, Shirouzu *et al.* (2016)
	TNS-F-88754	Japan	–	–	LC492177	–	–	–	Shirouzu *et al.* (2020)
	TNS-F-88777	Japan	–	–	LC492200	–	–	–	Shirouzu *et al.* (2020)
*C. cf. enatus*	OTU_466	Japan	–	–	LC492292	–	–	–	Shirouzu *et al.* (2020)
*C. enterolaxus holotype*	TNS-F-61292	Japan	–	**LC585244**	LC003895	–	–	–	this study, Shirouzu *et al.* (2016)
	MAFF 247132	Japan	–	–	LC492267	–	–	–	Shirouzu *et al.* (2020)
	OTU_356	Japan	–	–	LC492290	–	–	–	Shirouzu *et al.* (2020)
	TNS-F-15723	Japan	AB712504	AB712462	AB299052	–	–	AB712546	Shirouzu *et al.* (2007, 2013)
	TNS-F-15724	Japan	–	**LC585256**	AB299056	–	–	–	this study, Shirouzu *et al.* (2007)
	TNS-F-15725	Japan	**LC585261**	**LC585258**	AB299071	–	–	–	this study, Shirouzu *et al.* (2007)
	TNS-F-61296	Japan	–	**LC585245**	LC003899	–	–	–	this study, Shirouzu *et al.* (2016)
	TNS-F-61306	Japan	–	**LC585246**	LC003909	–	–	–	this study, Shirouzu *et al.* (2016)
	TNS-F-61316	Japan	–	**LC585247**	LC003919	–	–	–	this study, Shirouzu *et al.* (2016)
	TNS-F-61317	Japan	–	**LC585248**	LC003920	–	–	–	this study, Shirouzu *et al.* (2016)
	TNS-F-61319	Japan	–	**LC585249**	LC003922	–	–	–	this study, Shirouzu *et al.* (2016)
	TNS-F-61324	Japan	–	**LC585251**	LC003927	–	–	–	this study, Shirouzu *et al.* (2016)
	TNS-F-61327	Japan	–	**LC585252**	LC003930	–	–	–	this study, Shirouzu *et al.* (2016)
	TNS-F-61334	Japan	–	**LC585253**	LC003937	–	–	–	this study, Shirouzu *et al.* (2016)
	TNS-F-61335	Japan	–	**LC585254**	LC003938	–	–	–	this study, Shirouzu *et al.* (2016)
	TNS-F-88723	Japan	–	–	LC492146	–	–	–	Shirouzu *et al.* (2020)
	TNS-F-88726	Japan	–	–	LC492149	–	–	–	Shirouzu *et al.* (2020)
	TNS-F-88728	Japan	–	–	LC492151	–	–	–	Shirouzu *et al.* (2020)
	TNS-F-88734	Japan	–	–	LC492157	–	–	–	Shirouzu *et al.* (2020)
	TNS-F-88742	Japan	–	–	LC492165	–	–	–	Shirouzu *et al.* (2020)
	TNS-F-88745	Japan	–	–	LC492168	–	–	–	Shirouzu *et al.* (2020)
	TNS-F-88753	Japan	–	–	LC492176	–	–	–	Shirouzu *et al.* (2020)
	TNS-F-88762	Japan	–	–	LC492185	–	–	–	Shirouzu *et al.* (2020)
	TNS-F-88763	Japan	–	–	LC492186	–	–	–	Shirouzu *et al.* (2020)
	TNS-F-88767	Japan	–	–	LC492190	–	–	–	Shirouzu *et al.* (2020)
	TNS-F-88768	Japan	–	–	LC492191	–	–	–	Shirouzu *et al.* (2020)
	TNS-F-88781	Japan	–	–	LC492204	–	–	–	Shirouzu *et al.* (2020)
*C. favonius holotype*	H7008893	USA	**MW158962**	**MW191895**	**MW159041**	**MW130347**	**MW130374**	**MW130416**	this study
	H7008894	USA	**MW158961**	**MW191894**	**MW159040**	–	**MW130373**	**MW130415**	this study
*C. fugax holotype*	CFMR:HHB-8856	USA	–	**MW191905**	**MW159051**	–	–	–	this study
*C. hesperidis holoype*	NY01782362	USA	–	**MW191921**	**MW159065**	–	–	–	this study
*C. inermis holotype*	PDD87816	New Zealand	–	**MW191887**	–	**MW130324**	–	–	this study
*C. lipoferus holotype*	UPS:F-940777	Sweden	MN593436	MN595620	MN595620	MN580319	MN580219	MN580259	Zamora & Ekman (2020)
	ENZ20001	The Netherlands	–	**MZ147626**	**MZ147626**	**MZ152908**	–	–	this study
	GB-0161225	Sweden	–	**MW192002**	**MW192002**	–	–	–	this study
	UPS:F-940778	Sweden	–	**MW192001**	**MW192001**	–	–	–	this study
*C. nepalensis holotype*	O:F-904088	Nepal	–	**MW191896**	–	–	–	–	this study
*C. neuhoffii holotype*	UPS:F-941020	Sweden	MN593441	MN595625	MN595625	MN580324	MN580224	MN580264	Zamora & Ekman (2020)
	CWU(MYC)6281	Ukraine	**MW158979**	**MW191925**	**MW159031**	**MW130335**	**MW130366**	–	this study
	CWU(MYC)6342	Ukraine	**MW158977**	**MW191923**	**MW159030**	**MW130334**	–	–	this study
	H:Miettinen 15893	Finland	–	**MW191924**	**MW159028**	–	–	–	this study
	H:Miettinen 20778	Finland	**MW158978**	**MW191926**	**MW159032**	**MW130336**	–	–	this study
	TU135067	Ukraine	**MW158976**	**MW191922**	**MW159029**	**MW130333**	**MW130365**	–	this study
	UPS:F-941019	Spain	MN593440	MN595624	MN595624	MN580323	MN580223	MN580263	Zamora & Ekman (2020)
	UPS:F-946501	Sweden	**MW159026**	**MW192000**	**MW192000**	**MW130364**	**MW130407**	**MW130443**	this study
	UPS:F-946503	Sweden	**MW159015**	**MW191990**	**MW191990**	**MW130356**	**MW130397**	**MW130433**	this study
	UPS:F-946505	Cyprus	**MW159017**	**MW191992**	**MW191992**	**MW130358**	**MW130398**	**MW130434**	this study
	UPS:F-946510	Sweden	**MW159022**	**MW191997**	**MW191997**	–	**MW130403**	**MW130439**	this study
*C. pallidus*	ARIZ-M-AN09245	USA	–	**MZ147624**	–	–	–	–	this study
	CFMR:WBC-39924	USA	–	**MW191932**	–	–	–	–	this study
	GB-0071214	USA	–	**MW191936**	–	–	–	–	this study
	NY:”C. pallidus №1”	USA	–	**MW191931**	–	**MW130332**	–	–	this study
*C. paulistanus holotype*	O:Ryvarden 24759	Brazil	–	**MW191935**	–	–	–	–	this study
	TAAM192120	Brazil	–	**MW191934**	–	–	–	–	this study
*C. pinguis holotype*	O:F-904085	Nepal	–	**MW191907**	**MW159043**	**MW130348**	–	–	this study
*C. ramosissimus holotype*	CFMR:FP-150848	Belize	AB712488	AB712446	AB712426	–	–	AB712530	Shirouzu *et al.* (2013)
*C. tortus neotype*	UPS:F-946515	Sweden	**MW159025**	**MW191999**	**MW191999**	**MW130363**	**MW130406**	**MW130442**	this study
	H:Miettinen 12740.1	Finland	–	**MW191909**	–	–	–	–	this study
	H:Miettinen 14095	Finland	–	**MW191914**	–	–	–	–	this study
	H:Miettinen 20996	Finland	**MW158969**	**MW191910**	**MW159060**	–	–	–	this study
	H:Miettinen 21034	Finland	**MW158974**	–	**MW159057**	–	–	–	this study
	H:Miettinen 21058	Finland	**MW158971**	**MW191908**	**MW159062**	–	–	–	this study
	H:Miettinen 21288	Finland	**MW158970**	–	**MW159058**	**MW130342**	–	–	this study
	H:Miettinen 21292	Finland	**MW158973**	–	**MW159059**	–	–	–	this study
	H:Savchenko 181108-1431	Finland	**MW158968**	–	**MW159056**	**MW130341**	–	–	this study
	O160046	Norway	–	**MW191916**	**MW159061**	–	–	–	this study
	TU135048	Estonia	**MW158975**	**MW191911**	**MW159063**	–	–	–	this study
	TU135049	Estonia	**MW158972**	**MW191915**	**MW159064**	–	–	–	this study
	TU135066	Ukraine	**MW158967**	**MW191913**	**MW159055**	**MW130340**	**MW130368**	–	this study
	UPS:F-015301	Sweden	–	**MW191912**	–	–	–	–	this study
	UPS:F-941016	Sweden	MN593438	MN595622	MN595622	MN580321	MN580221	MN580261	Zamora & Ekman (2020)
	UPS:F-941017	Sweden	MN593439	MN595623	MN595623	MN580322	MN580222	MN580262	Zamora & Ekman (2020)
	UPS:F-941018	Sweden	**MW159014**	**MW191989**	**MW191989**	–	**MW130396**	**MW130432**	this study
	UPS:F-946508	Sweden	**MW159020**	**MW191995**	**MW191995**	**MW130359**	**MW130401**	**MW130437**	this study
	UPS:F-946511	Sweden	**MW159023**	**MW191979**	–	**MW130361**	**MW130404**	**MW130440**	this study
	UPS:F-946514	Sweden	**MW159024**	**MW191998**	**MW191998**	**MW130362**	**MW130405**	**MW130441**	this study
*C. aff. tortus 1*	UPS:F-946504	Sweden	**MW159016**	**MW191991**	**MW191991**	**MW130357**	–	–	this study
	UPS:F-946509	Finland	**MW159021**	**MW191996**	**MW191996**	**MW130360**	**MW130402**	**MW130438**	this study
*C. aff. tortus 2*	UPS:F-940948	Norway	MN593437	MN595621	MN595621	MN580320	MN580220	MN580260	Zamora & Ekman (2020)
*C. aff. tortus 3*	TNS-F-88757	Japan	–	–	LC492180	–	–	–	Shirouzu *et al.* (2020)
	TNS-F-88780	Japan	–	–	LC492203	–	–	–	Shirouzu *et al.* (2020)
*C. tristis holotype*	H7009711	USA	**MW158958**	**MW191906**	**MW159050**	**MW130325**	–	**MW130409**	this study
	CFMR:FP-133094	USA	–	**MW191897**	–	–	–	–	this study
	GB-0071225	Canada	–	**MW191898**	–	–	–	–	this study
	H:OM19013	USA	–	**MZ147625**	–	–	–	–	this study
*C. verecundus holotype*	PDD93708	New Zealand	–	**MW191930**	**MW159052**	**MW130329**	–	–	this study
*C. volaticus holotype*	S:F250344	Sweden	**MW159027**	**MW191982**	–	–	–	–	this study
	GB-0071193	Sweden	–	**MW191903**	–	–	–	–	this study
	GB-0071206	Sweden	–	**MW191900**	–	–	–	–	this study
	LE242249	Russia	**MW158960**	**MW191902**	**MW159048**	**MW130327**	–	–	this study
	LE295748	Russia	**MW158959**	**MW191901**	**MW159047**	**MW130326**	–	**MW130408**	this study
	O:F-247959	Norway	MN593435	MN595619	MN595619	MN580318	MN580218	MN580258	Zamora & Ekman (2020)
	O189348	Norway	–	**MW191899**	**MW159049**	–	–	–	this study
	PC0706779	France	–	**MW191904**	–	**MW130328**	–	–	this study
*Cerinomyces sp.*	05151-1B2	Japan	–	**LC585255**	LC003874	–	–	–	Shirouzu *et al.* (2016)
	1611_131A1	Japan	–	–	LC492227	–	–	–	Shirouzu *et al.* (2020)
	NBRC110591	Japan	LC004021	LC004000	LC003883	–	–	–	Shirouzu *et al.* (2016)
	OTU_258	Japan	–	–	LC492283	–	–	–	Shirouzu *et al.* (2020)
*Dacrymyces burdsallii holotype*	CFMR:HHB-6908	USA	AB712486	AB712444	AB712424	–	–	AB712528	Shirouzu *et al.* (2013)
*D. cf. capitatus*	TU135101	Estonia	**MW158995**	**MW191962**	**MW159081**	–	–	–	this study
*D. ceraceus holotype*	CFMR:HHB-8969	USA	AB712484	AB712442	AB712422	–	–	AB712526	Shirouzu *et al.* (2013)
*D. aff. ceraceus*	CFMR:HHB-6817	USA	–	**MW191951**	–	**MW130310**	–	–	this study
*D. cereus*	URM:Chikowski 1167	Brazil	–	**MW191954**	–	–	–	–	this study
*D. chrysocomus*	UPS:F-940134	Sweden	MN593446	MN595630	MN595630	MN580329	MN580229	MN580269	Zamora & Ekman (2020)
	UPS:F-940136	Spain	MN593445	MN595629	MN595629	MN580328	MN580228	MN580268	Zamora & Ekman (2020)
*D. chrysospermus*	H:Miettinen 14818	USA	**MW159000**	**MW191961**	**MW159077**	**MW130305**	–	–	this study
	H:Spirin 10795	Russia	**MW159001**	**MW191974**	**MW159078**	**MW130306**	**MW130387**	–	this study
*D. aff. chrysospermus*	UPS:F-593536	Japan	MN593447	MN595631	MN595631	MN580330	MN580230	MN580270	Zamora & Ekman (2020)
*D. corticioides*	NY:"C. canadensis №1"	USA	–	**MW191940**	**MW159067**	**MW130315**	–	–	this study
	NY02686162	USA	**MW159006**	**MW191944**	**MW159068**	**MW130314**	–	–	this study
	TAAM102301		–	**MW191941**	–	–	–	–	this study
	TAAM126607		–	**MW191943**	**MW159066**	–	–	–	this study
	TAAM150056		–	**MW191942**	–	–	–	–	this study
*D. estonicus*	UPS:F-940137	Sweden	MN593448	MN595632	MN595632	MN580331	MN580231	MN580271	Zamora & Ekman (2020)
	UPS:F-940138	Sweden	MN593449	MN595633	MN595633	MN580332	MN580232	MN580272	Zamora & Ekman (2020)
*D. fennicus*	H:Miettinen 20574	Finland	**MW158990**	–	**MW159072**	**MW130303**	**MW130379**	**MW130430**	this study
	H:Miettinen 21174	Finland	**MW158989**	**MW191957**	**MW159071**	–	**MW130378**	**MW130421**	this study
	UPS:F-946596	Sweden	**MZ130256**	**MZ147627**	**MZ147627**	–	**MZ152906**	–	this study
	UPS:F-946597	Sweden	**MZ130257**	**MZ147628**	**MZ147628**	**MZ152909**	**MZ152907**	–	this study
*D. grandii holotype*	NCSLG21158	USA	–	**MW191953**	–	**MW130309**	–	–	this study
*D. grandinioides holotype*	K(M):237139	Kenya	–	**MW191980**	–	–	–	–	this study
	H7008841	Kenya	**MW158994**	**MW191950**	**MW159076**	**MW130312**	**MW130390**	**MW130418**	this study
	LY11615	Réunion	–	**MW191981**	–	–	–	–	this study
*D. cf. minor*	H:Miettinen 19137	Finland	**MW158998**	**MW191967**	**MW159080**	**MW130301**	**MW130385**	**MW130427**	this study
	H:Miettinen 20591	Finland	**MW158997**	**MW191965**	**MW159079**	**MW130300**	**MW130384**	**MW130426**	this study
*D. minutus*	UPS:F-940776	Finland	MN593450	MN595634	MN595634	MN580333	MN580233	MN580273	Zamora & Ekman (2020)
*D. ovisporus*	H:Miettinen 20787	Finland	**MW159004**	**MW191964**	**MW159074**	**MW130318**	**MW130392**	–	this study
	H:Spirin 11145	Norway	**MW159003**	**MW191960**	**MW159073**	**MW130317**	**MW130391**	–	this study
	UPS:F-940139	Sweden	MN593451	MN595635	MN595635	MN580334	MN580234	MN580274	Zamora & Ekman (2020)
	UPS:F-940140	Sweden	MN593452	MN595636	MN595636	MN580335	MN580235	MN580275	Zamora & Ekman (2020)
*D. pinacearum*	UPS:F-593533	Japan	MN593453	MN595637	MN595637	MN580336	MN580236	MN580276	Zamora & Ekman (2020)
	UPS:F-593535	Japan	MN593454	MN595638	MN595638	MN580337	MN580237	MN580277	Zamora & Ekman (2020)
*D. sobrius holotype*	CFMR:RLG-13487	USA	AB712487	AB712445	AB712425	–	–	AB712529	Shirouzu *et al.* (2013)
	CFMR:FP-102085	USA	–	**MW191952**	–	–	–	–	this study
*D. stillatus (anamorph)*	UPS:F-939814	Sweden	MN593455	MN595676	MN595676	MN580338	MN580238	MN580278	Zamora & Ekman (2020)
	UPS:F-939816	Sweden	**MN593457**	–	**MN593494**	**MN580340**	**MN580240**	**MN580280**	this study
*D. stillatus (teleomorph)*	UPS:F-939814	Sweden	MN593456	MN595677	MN595677	MN580339	MN580239	MN580279	Zamora & Ekman (2020)
	UPS:F-939816	Sweden	**MN593458**	–	**MN593495**	**MN580341**	**MN580241**	**MN580281**	this study
*D. cf. stillatus*	H:Miettinen 20608	Finland	**MW158996**	**MW191963**	**MW159082**	**MW130304**	**MW130383**	**MW130425**	this study
*D. venustus holotype*	O:Adane 150	Ethiopia	**MW158993**	**MW191949**	**MW159075**	**MW130311**	–	**MW130417**	this study
*D. aff. venustus 1*	LY7839	Gabon	–	**MW191948**	–	–	–	–	this study
*Dacryonaema macnabbii*	UPS:F-940949	Sweden	MN593472	MN595650	MN595650	MN580353	–	MN580292	Zamora & Ekman (2020)
	UPS:F-940992	Sweden	MN593475	MN595653	MN595653	MN580356	MN580211	MN580295	Zamora & Ekman (2020)
*D. macrosporum*	UPS:F-940998	Finland	MN593480	MN595660	MN595660	MN580360	MN580215	MN580302	Zamora & Ekman (2020)
	UPS:F-941001	Finland	MN593481	MN595661	MN595661	MN580361	MN580216	MN580303	Zamora & Ekman (2020)
*D. rufum*	UPS:F-941005	Sweden	MN593469	MN595646	MN595646	MN580349	MN580209	MN580288	Zamora & Ekman (2020)
	UPS:F-941012	Finland	MN593470	MN595649	MN595649	MN580351	–	MN580290	Zamora & Ekman (2020)
*Dacryopinax elegans*	TENN 066927	USA	MN593460	MN595640	MN595640	MN580342	MN580242	MN580282	Zamora & Ekman (2020)
*Dacryopinax sp.*	H7008759	Kenya	**MW158992**	**MW191959**	**MW159091**	–	–	–	this study
*D. spathularia*	H:Miettinen 16740.1	USA	**MW158999**	**MW191973**	**MW159085**	**MW130308**	**MW130389**	**MW130429**	this study
	H:Miettinen 20559	Indonesia	**MW159007**	**MW191976**	**MW159092**	–	–	–	this study
*Ditiola radicata*	H:Miettinen 20590.2	Finland	**MW158987**	**MW191966**	**MW159083**	**MW130313**	–	–	this study
	UPS:F-939957	Sweden	MN593461	MN595641	MN595641	MN580343	MN580243	MN580283	Zamora & Ekman (2020)
	UPS:F-939961	Finland	**MN593462**	–	–	**MN580344**	**MN580244**	**MN580284**	this study
*Femsjonia peziziformis*	H:Haikonen 24269	Finland	**MW158991**	**MW191972**	**MW159070**	**MW130316**	**MW130377**	**MW130420**	this study
	H:Haikonen 30097	Finland	MN593463	MN595642	MN595642	MN580345	MN580245	MN580285	Zamora & Ekman (2020)
*Guepiniopsis buccina*	CWU(MYC)7014	Ukraine	**MW159002**	**MW191971**	**MW159086**	**MW130319**	**MW130386**	–	this study
	UPS:F-940947	Spain	MN593464	MN595643	MN595643	MN580346	MN580246	MN580286	Zamora & Ekman (2020)
*Heterotextus alpinus*	H:Spirin 8744	USA	**MW158988**	–	–	–	–	–	this study
*H. miltinus*	TENN 42208	New Zealand	MN593465	MN595644	MN595644	MN580347	MN580247	–	Zamora & Ekman (2020)
*Unilacryma bispora*	UPS:F-941254	Sweden	MN593488	MN595670	MN595670	MN580367	MN580253	MN580312	Zamora & Ekman (2020)
	UPS:F-941268	Sweden	MN593490	MN595672	MN595672	MN580369	MN580255	MN580314	Zamora & Ekman (2020)
*U. unispora*	UPS:F-941277	Sweden	MN593483	MN595665	MN593500	MN580362	MN580248	MN580307	Zamora & Ekman (2020)
	UPS:F-941278	Sweden	MN593484	MN595666	MN595666	MN580363	MN580249	MN580308	Zamora & Ekman (2020)

### Phylogenetic analysis

General sequence management and contig assembly were done in Geneious v. 7.0.6 and 9.1.8 (https://www.geneious.com). Alignments were performed in MAFFT v. 7 online with E-INS-i method (https://mafft.cbrc.jp/alignment/server/, Katoh *et al.* 2019). Borders of ITS1, 5.8S, ITS2 and LSU were identified with ITSx (Bengtsson-Palme *et al.* 2013) as implemented at PlutoF (https://plutof.ut.ee/#/analysis) or using ITS2 database (http://its2.bioapps.biozentrum.uni-wuerzburg.de/, Ankenbrand *et al.* 2015). Parts of the alignments were excluded by hand (poorly aligned and heterogenous regions of nrDNA, most of introns in protein-coding genes). Final alignments contain positions with > 60 % of gaps; more stringent trimming (50 % gaps allowed) resulted in trees with identical topologies and similar supports (not presented here), and therefore more relaxed alignments were retained. Trimming was performed in BMGE (Criscuolo & Gribaldo 2010) as implemented at NGPhylogeny.fr (Lemoine *et al.* 2019); test trees were built in RAxML similar to described below. Manual adjustments to alignments were done with AliView v. 1.26 (Larsson 2014). Eight concatenated datasets were built with different genes (Table 2) using package evobiR (Adams 2015) in R environment (R Core Team 2019). Regions of nrDNA were treated as five separate partitions (SSU, ITS1, 5.8S, ITS2, LSU), and protein coding genes were divided into 1–2 *vs* 3 codon positions, yielding six partitions for TEF1-α, RPB1 and RPB2. To check incongruence between partitions in *Dacrymycetes* set, we compared maximum likelihood trees (built as explained below) based on separate genes (SSU, 5.8S, LSU, and not partitioned into codons TEF1-α, RPB1, RPB2). We considered lack of conflict among single-region trees when no samples were included in different supported clades across trees (≥ 70 % bootstrap support). The only discordance occurred in RPB2 tree were *Dacrymyces grandinioides* and *D. venustus* fell outside of the core *Dacrymycetaceae* clade, separated from *D. burdsallii*, *D. ceraceus*, and *D. sobrius*.

**Table 2:** List of concatenated sequence datasets and gene partitions included in them. Numbers of characters (total and parsimony-informative) are separated with slash (/).

Dataset name	Rows	Length	SSU	ITS1	5.8S	ITS2	LSU	TEF1-α, 1–2 pos.	TEF1-α, 3 pos.	RPB1, 1–2 pos.	RPB1, 3 pos.	RPB2, 1–2 pos.	RPB2, 3 pos.
*Cerinomyces albosporus* clade, Fig. 5 C	10	1 407/180	—	87/33	149/5	132/41	526/23	342/39	171/39	—	—	—	—
*C. borealis* clade, Fig. 7 A	11	3 621/254	1 454/44	96/31	149/4	136/36	1 227/94	373/23	186/22	—	—	—	—
*C. enatus* clade, Fig. 6	55	2 879/436	1 636/144	101/50	151/14	157/87	834/141	—	—	—	—	—	—
*C. pallidus* clade (*C. atrans* subclade), Fig. 5 A	12	382/109	—	90/36	149/9	143/64	—	—	—	—	—	—	—
*C. pallidus* clade (*C. volaticus* subclade), Fig. 5 B	13	1 598/143	—	76/34	149/8	147/53	1 226/48	—	—	—	—	—	—
*C. tortus* clade, Fig. 7 B	37	2 731/304	1 582/96	88/43	151/33	119/64	791/68	—	—	—	—	—	—
*Dacrymycetes*, Fig. 4	110	6 471/2 679	1 511/340	—	136/38	—	1 152/343	644/178	322/288	472/221	237/233	1 332/398	665/640
*Dacrymyces grandinioides* clade, Fig. 8	10	2 500/171	1 585/24	107/32	142/12	169/71	497/32	—	—	—	—	—	—

Bayesian inference was performed with MrBayes v. 3.2.7a (Ronquist *et al.* 2012) as implemented at CIPRES portal (Miller *et al.* 2010). Default priors were used, nucleotide substitution models were estimated with model jumping method (nst = mixed) with gamma-distributed rate variation across sites and proportion of invariable sites not estimated. Analyses were carried out in four parallel runs with four MCMC chains each, for 10 M generations, sampling trees every 5 000 generations, with temperature constant 0.1. A burn-in was set to a fraction 25 %. The analyses were automatically stopped if the average standard deviation of split frequencies dropped below 0.01. Effective sample sizes (ESS) were assumed sufficient with values reaching above 200, and potential scale reduction factor (PSRF) approximating to 1. Tracer v. 1.7.1 (Rambaut *et al.* 2018) and RWTY (Warren *et al.* 2017) were used to observe convergence of model parameters and tree topologies. The consensus tree was built using 50 % majority rule.

Maximum likelihood (ML) analyses were performed with RAxML v. 8.2.12 (Stamatakis 2014), implemented at CIPRES portal as “RAxML-HPC2 Workflow”, using the same partitioning as above, GTRGAMMA model for all datasets, with 10 randomized maximum-likelihood initial trees and 1 000 iterations of standard non-parametric bootstrap. The trees were plotted in FigTree v. 1.4.3 (http://tree.bio.ed.ac.uk/software/figtree/). The alignments and phylograms have been deposited in TreeBASE, study number S28188 (https://www.treebase.org/). Data related to the page are also available at https://plutof.ut.ee/#/doi/10.15156/BIO/1420800.

## Results

**Fig. 4:**
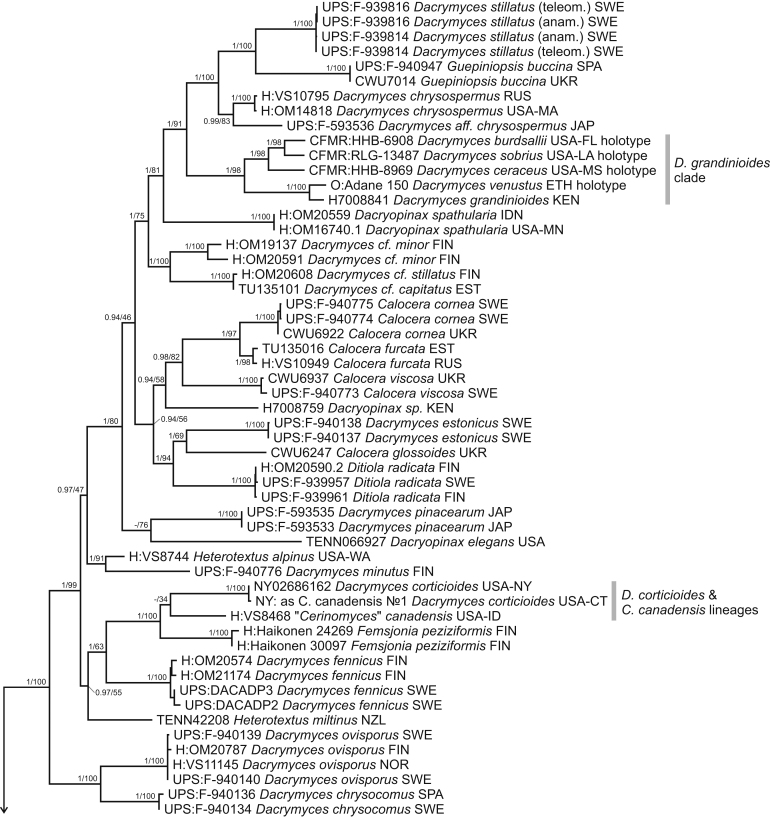

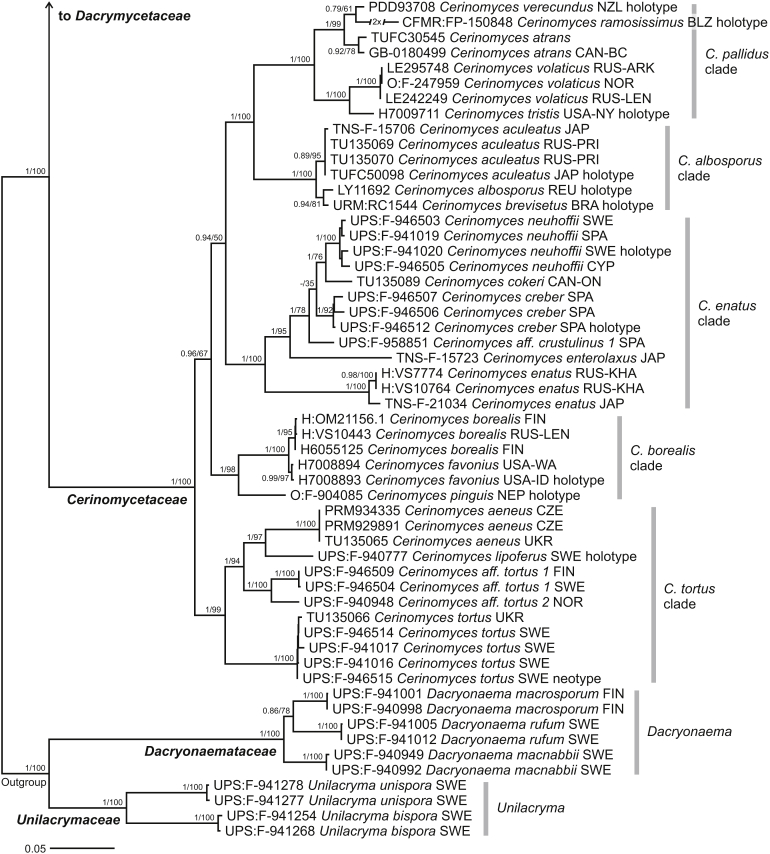
Phylogeny of the *Dacrymycetes*. Maximum-likelihood consensus tree based on SSU, 5.8S, LSU. RPB1, RPB2, and TEF1-α sequences. Numbers before and after slash (/) indicate posterior probabilities of Bayesian analysis and ML bootstrap support values. Codes after the species names denote country and admin. division of origin (ISO 3166).

A dataset of SSU, 5.8S, LSU, TEF1-α, RPB1, and RPB2 genes was used to infer the class phylogeny and resolve the positions of *Cerinomyces s.l.* clades. Our analysis confirmed the earlier reported family arrangement of the *Dacrymycetes*, with the robustly supported *Cerinomycetaceae* as a sister clade to the *Dacrymycetaceae* (Fig. 4). From a morphological perspective, the combination of characters that unites the *Cerinomycetaceae* when compared to other families is: (i) presence of simple clamps on all hyphal septa; (ii) curved-cylindrical thin-walled basidiospores that only rarely and tardily develop up to three transverse septa; (iii) low amount of carotenoid pigments in hyphae and basidiospores; and (iv) corticioid, resupinate, pustulate, pulvinate and only slightly cerebriform basidiocarps. In addition, young basidiospores of the family members appear to be binucleate, while in the rest of the class uninucleate state is usual. The character was observed in fresh material of ten *Cerinomyces* taxa and reported in descriptions.

In contrast to the traditional view, corticioid morphotype alone does not define the family: while most corticioid dacrymycetes do belong to the *Cerinomycetaceae*, a number of *Cerinomyces s.l.* are found in the *Dacrymycetaceae*. In agreement with earlier studies, one of the excluded groups — a clade containing *D. grandinioides* — is resolved as a sister to *Dacrymyces stillatus*, *D. chrysospermus* and *Guepiniopsis buccina*. Here we formally transfer all taxa related to *D. grandinioides* to *Dacrymyces*. Two other corticioid species, “*Cerinomyces*” *canadensis* and *D. corticioides*, are recovered in proximity to *Femsjonia peziziformis*. We refrain from nomenclatural rearrangements of these until a dedicated study of *Femsjonia* is undertaken.

Based on the same dataset, we empirically designated five clades within the *Cerinomycetaceae* to highlight connections between phylogenetic and morphological groups. We treat all clades as part of *Cerinomyces*: in our opinion, division of the family into several genera is impractical. Even though morphology is generally uniform within the clades, it is still not possible to identify characters that would unambiguously define every putative genus. In addition, establishing generic boundaries is not justified given the low support values in some of the deeper nodes in the family phylogeny.

**Fig. 5:**
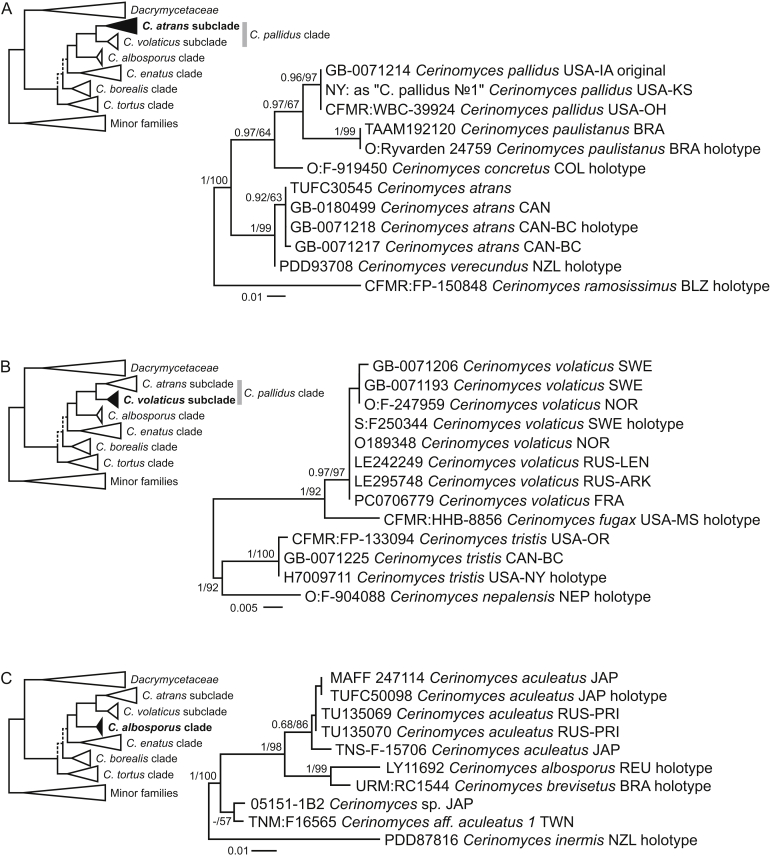
Phylogenies of *Cerinomyces* clades. **A.** The *C. atrans* subclade from the *C. pallidus* clade, based on ITS. **B.** The *C. volaticus* subclade from the *C. pallidus* clade, based on ITS and LSU. **C.** The *C. albosporus* clade, based on ITS, LSU, and TEF1-α. Mid-rooted maximum-likelihood consensus trees. Numbers before and after slash (/) indicate posterior probabilities of Bayesian analysis and ML bootstrap support values. Codes after the species names denote country and admin. division of origin (ISO 3166).

**Fig. 6:**
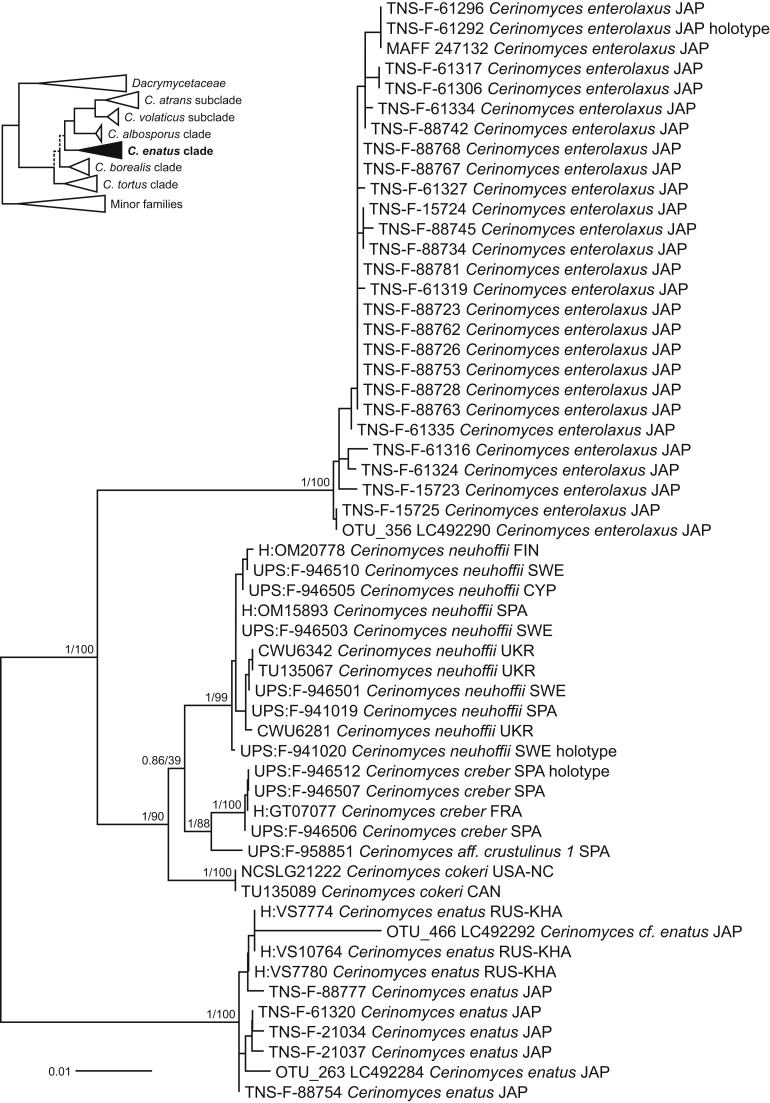
Phylogeny of the *Cerinomyces enatus* clade. Mid-rooted maximum-likelihood consensus tree based on SSU, ITS, and LSU sequences. Numbers before and after slash (/) indicate posterior probabilities of Bayesian analysis and ML bootstrap support values. Codes after the species names denote country and admin. division of origin (ISO 3166).

**Fig. 7:**
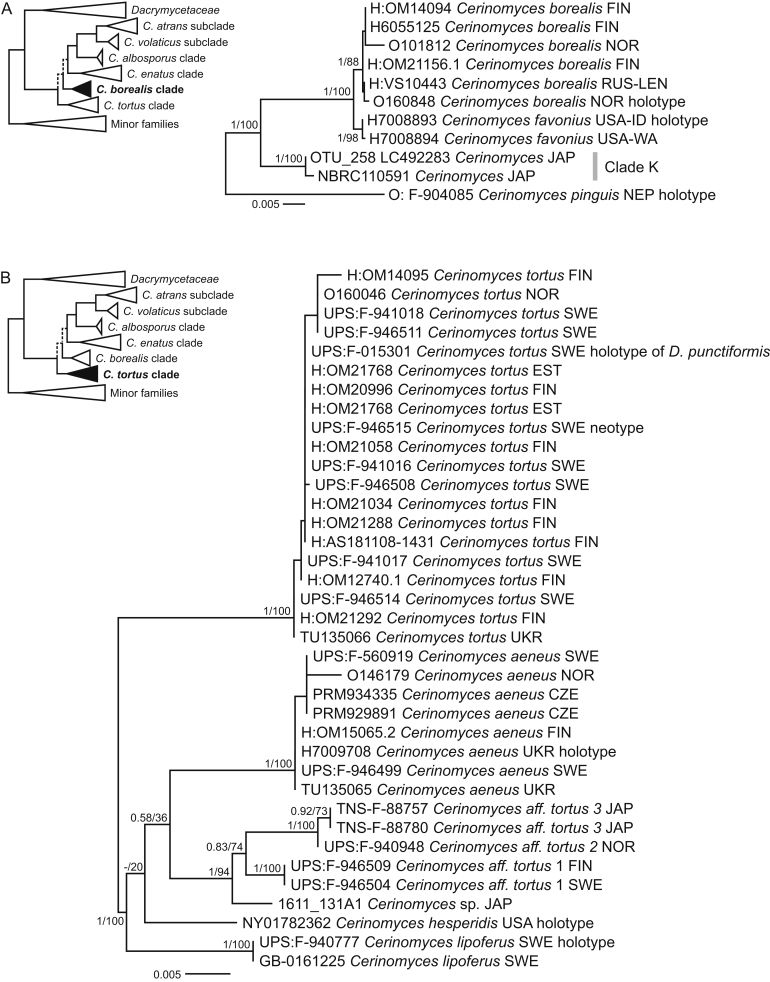
Phylogenies of *Cerinomyces* clades. **A.** The *C. borealis* clade, based on SSU, ITS, LSU, and TEF1-α. **B.** The *C. tortus* clade, based on SSU, ITS, and LSU. Mid-rooted maximum-likelihood consensus trees. Numbers before and after slash (/) indicate posterior probabilities of Bayesian analysis and ML bootstrap support values. Codes after the species names denote country and admin. division of origin (ISO 3166).

Two main types of basidiocarps, arid corticioid and gelatinous pustulate, are found in the *Cerinomycetaceae*. As shown in the Fig. 4, corticioid species are scattered across three groups (labelled here as *C. pallidus*, *C. albosporus* and *C. borealis* clades), while the gelatinous ones form two (*C. enatus* and *C. tortus* clades). To increase the resolution within these clades, we utilized ITS and TEF1-α sequences. ITS was too variable for family-wide alignments, so we divided the data into clade- or even subclade sets, presented in separate trees (Fig. 5, Fig. 6, Fig. 7). SSU and LSU were also incorporated in the concatenated sets, but predictably showed little parsimony-informative signals at the species level. For the species delimitation, we primarily used ITS as a barcoding marker available for most of the taxa. In total, the genus *Cerinomyces* includes 29 species, of which 20 are newly described here. In addition, we propose three new combinations, designate four informal taxa and exclude seven species from the genus. The clades are detailed below.

The *C. pallidus* clade encompasses the generic type *C. pallidus* and its nine corticioid relatives. These inhabit angiosperm and gymnosperm wood mostly in the temperate zones of the Northern and Southern hemispheres. Basidiocarps are pale ochraceous, varying from arachnoid to crustose with a thin cottony subiculum. *Cerinomyces pallidus* and *C. paulistanus* develop hyphal pegs, and several related species demonstrate microscopic peg-like hyphal constructions (like Fig. 39 C), but never as abundant and regular as in the *C. albosporus* clade members. Microscopic features often blend between species, requiring comparison of multiple characters for non-molecular identification. Because of the differences in ITS, we prepared separate phylogenies for the *C. atrans* and *C. volaticus* subclades (Fig. 5 A, B). Basidiocarps in the first subclade are more likely to become crustose and darken to different extents. This character was observed in all members, namely in *C. atrans*, *C. concretus*, *C. pallidus*, *C. paulistanus*, *C. ramosissimus*, and *C. verecundus.* Species in the second subclade (*C. fugax*, *C. nepalensis*, *C. tristis*, and *C. volaticus*) usually remain arachnoid or solid, but still cottony, and light-coloured.

The *C. albosporus* clade (Fig. 5 C), sister to the *C. pallidus* clade, includes four corticioid species confined to climates ranging from humid temperate to tropical. Three of these species (*C. aculeatus*, *C. albosporus*, and *C. brevisetus*) have abundant, regularly distributed hyphal pegs and larger microstructures than in other arid *Cerinomyces*. The fourth species, *C. inermis*, is distinguished by the absence of hyphal pegs and basidiospores that conform better to some members of the *C. borealis* and *C. pallidus* clades. In the absence of sequence data, our judgement from morphology indicates that further peg-bearing species are also allied with the clade: *C. fasciculatus* collected from Hawaiʻi and *C. curvisporus* from Southwest China, known for its strongly bent basidiospores.

The *C. borealis* clade includes three corticioid species growing on coniferous wood. Two closely related species from temperate Europe and North America, *C. borealis* and *C. favonius*, have the narrowest basidiospores in the genus. The third species, *C. pinguis*, was collected from mountains of Nepal, and possesses much larger basidiospores. Macromorphologically the group is difficult to distinguish from other arid *Cerinomyces* members, though it develops more delicate, often arachnoid basidiocarps without pegs. Clade K from Shirouzu *et al.* (2016, 2020) corresponds to a part of the *C. borealis* clade and represents Japanese environmental samples and strains. Their sequences differ from the specimen-based ones, suggesting undescribed diversity in the clade (Fig. 7 A).

The *C. enatus* clade members (Fig. 6) look like gelatinous *Dacrymyces* species with pustulate basidiocarps, though they lack the hallmark bright yellow tints of *Dacrymycetaceae*, being instead pale yellow, ochraceous, dark brown and reddish brown*.* Microscopically, the clade is characterized by heavily gelatinized hyphae and the presence of dendroid hyphidia in the hymenium of all the species. The clade consists of *C. cokeri*, *C. creber*, *C. enatus*, *C. enterolaxus*, and *C. neuhoffii*, all of which grow in moderate to highly humid conditions in the biogeographic Northern temperate zone. We found that widely recognized *C. enatus* (≡ *D. enatus* [Berk. & M.A. Curtis] Massee) occurs only in North America and East Asia. For European material formerly identified as *D. enatus*, we introduce a new species, *C. aeneus*, that belongs to the *C. tortus* clade. *Cerinomyces crustulinus*, whose name was massively misapplied to corticioid members, is likely to be related to the *C. enatus* clade. However, in the absence of fresh collections and sequence data, its position is difficult to resolve with confidence.

The *C. tortus* clade (Fig. 7 B) comprises four species with gelatinous basidiocarps, including *C. tortus*, one of the earliest described *Dacrymyces* species (as *D. tortus* [Willd.] Fr). The distribution and morphological characters of the clade often overlap with the *C. enatus* clade, but in the phylogeny their relation is not well supported (Fig. 4). The clade members develop mostly pustulate basidiocarps, with the exception of *C. aeneus*, that also can demonstrate coalescing to resupinate morphology. *Cerinomyces tortus* itself has a number of morphologically similar relatives, from which we formally describe only *C. lipoferus*. At least two more Nordic taxa are represented by scarce specimens that are not suitable as types, and whose intraspecific variation is poorly known. The main character that helps to effectively distinguish *C. tortus, C. hesperidis* and *C. lipoferus* from the other gelatinous species is the lack of finely branched hyphidia. In addition, *C. lipoferus* demonstrates a high amount of lipid droplets in hyphae, which is unusual for the family, and scattered 3-septate basidiospores, unique among the gelatinous species studied and very infrequent in the genus. Morphology-based identification in *C. enatus* and *C. tortus* clades is possible, though differentiating European species can be problematic if basidiocarps are young or weathered. Identification by ITS marker can also be difficult due to high levels of intragenomic polymorphism hampering Sanger sequencing and contig assembly.

All other known corticioid species of *Cerinomyces s.l.* belong to the *Dacrymycetaceae* and are considerably different from the *Cerinomycetaceae.* They have more robust, richly coloured corticioid basidiocarps with hymenial surfaces that become waxy- or firmly-gelatinous if moisturized, thick light-coloured subiculum, and fimbriate margins. In these groups we describe four new species, propose six combinations and one informal taxon.

The *C. canadensis* lineage consists of a single species collected in East Asia and North America. It has corticioid pale to dark orange basidiocarps of various shapes, sometimes effused over several centimeters. The species sometimes possesses so-called pseudoclamps that are rare among dacrymycetes (see a note under the species and Fig. 23 D). “*Cerinomyces*” *canadensis* together with *D. corticioides* are related to *Femsjonia* (Fig. 4), which is well reflected in their morphological similarity to *Femsjonia* species.

The *D. corticioides* lineage encompasses one species with circular, separate, later coalescing basidiocarps of yellow to orange colour, hyphae with clamp connections and tardily septate basidiospores. Here we show that *D. corticioides*, traditionally recognized as a North American species, has an amphi-Pacific distribution in the Northern Hemisphere and is identical to *C. altaicus* described from the Russian Far East. *Femsjonia uniseptata* with brightly yellow firm-gelatinous basidiocarps appears to belong to *D. corticioides*. European *D. confluens* is also very similar morphologically to *D. corticioides*, but due to the lack of sequence data, we treat it separately*.*

**Fig. 8:**
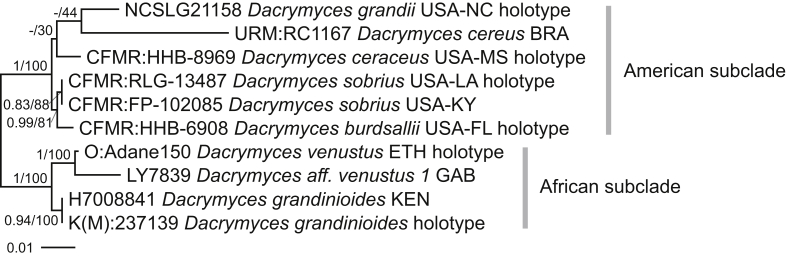
Phylogeny of the *Dacrymyces grandinioides* clade. Maximum-likelihood consensus tree based on SSU, ITS, and LSU sequences, rooted between subclades. Numbers before and after slash (/) indicate posterior probabilities of Bayesian analysis and ML bootstrap support values. Codes after the species names denote country and admin. division of origin (ISO 3166).

The seven species of the *D. grandinioides* clade tend to have relatively thick basidiocarps, a waxy-gelatinous yellow hymenial layer, clampless hyphae and three-septate, usually thick-walled basidiospores. Members of the clade occur in Africa and in the Americas, and are accordingly divided into two subclades (Fig. 8). On the grounds of morphology, we associate two non-sequenced species with this group: *D. pulchrus* and *D. lagerheimii*. We suppose “*Cerinomyces bambusicola*” *nom. prov.* belongs here as well because of its colour and prominently odontioid basidiocarps (Gminder 2016). In this group, microscopical characters vary even within a single specimen, which makes species delimitation particularly difficult.

### Taxonomy

#### Key and identification tables

The key covers all dacrymycetes with corticioid basidiocarps regardless of colour, as well as small gelatinous taxa without long stalks, of bleak yellow, ochraceous, light to dark brown and reddish brown colour. Some of them can be confused with members of the *Cerinomycetaceae*: certain *Dacrymyces* species and members of the families *Unilacrymaceae* and *Dacryonaemaceae*, excluding *Dacryonaema rufum* that has unique, easily recognizable synnematous basidiocarps (Nannfeldt 1947, Zamora & Ekman 2020). We render difficult groups as separate character tables outside of the main key: clamped, arid, pale-coloured corticioids from the *C. pallidus*, *C. borealis* and *C. albosporus* clades (Table 3), *Cerinomyces* species with gelatinous basidiocarps from the *C. enatus* and *C. tortus* clades (Table 4), and clampless, slightly gelatinous, yellow corticioid species with three-septate basidiospores — *Dacrymyces* species from the *D. grandinioides* clade (Table 5).

1. Basidiocarps pustulate, shallow-cupulate, less often cerebriform or resupinate. Always fully gelatinous when fresh…………………………………………………………2
1. Basidiocarps corticioid, either always arid or with a waxy-gelatinous hymenial surface when fresh………………19  
2. Most hyphal septa clampless. Elongated, medallion-like clamps occur in subhymenium. *Dacryonaemataceae.* The following choice #3 *fide* Zamora & Ekman (2020)…………3
2. Clamps present on all or almost all hyphal septa, of typical semi-circular form……………..…………………………….4  
3. Mature basidiospores on average < 14.0 × 5.3 μm…….….….….….….….…….…***Dacryonaema macnabbii***
3. Mature basidiospores on average > 14.5 × 5.3 μm….…….……………………***Dacryonaema macrosporum***  
4. Basidiospores subglobose or ellipsoid (Q < 2.2), basidia ≥ 60 μm in length. In internal hyphae clamps sometimes absent or appear as pseudoclamps. *Unilacrymaceae*…………………………………………….5
4. Basidiospores cylindrical to slightly curved-cylindrical (Q ≥ 2.2), basidia < 60 μm in length. Clamps present on all septa...………………………………………………………6  
5. Basidia unisterigmate, basidiospores subglobose (Q = 1.4, Q’ = 0.9–1.9)……………***Unilacryma unispora***
5. Basidia bisterigmate, basidiospores ellipsoid (Q = 2, Q’ = 1.6–2.4)……………………...…***Unilacryma bispora***  
6. Basidiospores regularly 3-septate…………………...…...7
6. Basidiospores aseptate or tardily septate…….9 and Table 4  
7. Branched hyphidia present…………………………………………………………***Dacrymyces paraphysatus s.l.***
7. Branched hyphidia absent…………………………………8  
8. Angiosperm substrates. Central European species……….…….……………..…***Dacrymyces adpressus s.l.***
8. Gymnosperm substrates. Northern European species….……….…….…………………...***Dacrymyces fennicus***  
9. Angiosperm substrates…………………………………..10
9. Gymnosperm substrates…………………………………12  
10. Basidiocarps often protrude through cracks or lenticels in bark. North American and Asian species…………………........................***Cerinomyces enatus***
10. Basidiocarps usually grow on decorticated wood. European species………………………………………………11  
11. Basidiocarps yellowish brown to reddish brown and dark brown, in mature state usually resupinate-cerebriform…………………………………***Cerinomyces aeneus***
11. Basidiocarps light yellow to light brown even when dry, not cerebriform……………………..................................***Cerinomyces crustulinus*** and ***C. aff. crustulinus 1***  
12. Hyphidia absent or rare, weakly branched and robust (e.g., Fig. 43 C)……………………………………………13
12. Hyphidia always present, abundant, finely branched (e.g., Fig. 38 B)……………………………………………15  
13. Mature basidiocarps often short stalked and centrally depressed, ≤ 1 mm in diam. North American species………………………..……***Cerinomyces hesperidis***
13. Basidiocarps sessile, rooted in substrate, but usually without visible stalk. Mature basidiocarps > 1 mm in diam. European species…………………………………14  
14. Basidiospores 0–1(–3)-septate. Hyphae with large amount of lipid droplets. Fresh basidiocarps typically whitish to yellowish or cream coloured…………………………………………………….***Cerinomyces lipoferus***
14. Basidiospores aseptate or extremely rarely 1-septate. Hyphae with low amount of lipid droplets. Fresh basidiocarps light yellowish to brown…………………………………………………………......***Cerinomyces tortus***  
15. Basidiocarps pulvinate, also resupinate and cerebriform, often > 1.5 mm in the longest dimension, commonly protruding through bark…………………………………..16
15. Basidiocarps pustulate, flattened, cupulate or slightly cerebriform, normally < 1.5 mm in diam, usually growing on decorticated wood…………………………………….17  
16. Basidiocarps light yellow or light brown when fresh, dark brown when dry. No swollen cells in subiculum………………………………..***Cerinomyces cokeri***
16. Basidiocarps dark brown when fresh, almost black when dry. Swollen cells abundant in subiculum of mature basidiocarps………………………..***Cerinomyces enatus***  
17. Subicular hyphae loosely arranged, hyphal walls in subiculum ≤ 0.5 μm in width, without a substantial gelatinous layer. Asian species……………………………………………………......***Cerinomyces enterolaxus***
17. Subicular hyphae densely arranged, hyphal walls in subiculum with a conspicuous gelatinous layer, together 0.5–1 μm in width. European species……………………………………….18 and ***Cerinomyces aff. tortus 1 & 2***  
18. Basidiocarps frequently dark brown to greyish brown when fresh, often but not always coalescing. Basidiospores on average 11.6 × 3.8 μm. Occurs on *Pinaceae* wood…...…………………………***Cerinomyces neuhoffii***
18. Basidiocarps frequently light brown when fresh, readily coalescing. Basidiospores on average 9.4 × 3.3 μm. Occurs on *Cupressaceae* wood…***Cerinomyces creber***  
19. Clamps absent on all septa……………………….Table 5
19. Clamps present on all septa………………………..……20  
20. Hyphal pegs visible to naked eye…………………….…21
20. Hyphal pegs absent or microscopic…………………..…27  
21. Basidiospores strongly curved………………………........………………..………………***Cerinomyces curvisporus***
21. Basidiospores only slightly curved………………………22  
22. Macroscopic pegs irregular, scattered, can be absent. Microscopic pegs always present. Basidiospores L < 9 μm………………………………………………....…23
22. Macroscopic pegs regular, frequent, always present. Basidiospores L > 9 μm………………………………..…24  
23. Macroscopic pegs present on most of well-developed basidiocarps. Hyphal swellings in subiculum absent or rare. North American species…………………………………………….................***Cerinomyces pallidus***
23. Well-developed basidiocarps often have no macroscopic pegs. Hyphal swellings present in subiculum. South American species……………***Cerinomyces paulistanus***  
24. Basidiospores 0–1(–3)-septate, L > 12 μm. Hyphal pegs > 150 μm in length………………………………….25
24. Basidiospores aseptate, L < 12 μm. Hyphal pegs < 150 μm in length………………………………….26  
25. Basidiocarps are the most robust among peg-bearing taxa. Found on woody *Asteraceae* shrub. African species…………………………….***Cerinomyces albosporus***
25. Basidiocarps more subtle. Grows mostly on gymnosperm wood. Asian species………..…***Cerinomyces aculeatus***  
26. The smallest basidiospores among the related species, 9.3–11.1(–11.7) × 3.5–4.4(–4.6) μm. South American species…………………………***Cerinomyces brevisetus***
26. Basidiospores slightly larger, 10–12.5(–13) × 4.5–5.5 μm. The species known only from Hawaiʻi…………………….…***Cerinomyces fasciculatus***  
27. Basidiocarps thin, white to ochraceous, subiculum and margins delicate or lacking. Hymenial surface typically arid when fresh. Mature basidiospores aseptate……………………………………………………Table 3
27. Basidiocarps thick, yellow to orange when fresh, with coarse subiculum and fimbriate margins. Hymenial surface firm waxy-gelatinous when fresh. Mature basidiospores 3-septate……………………………………….…28  
28. Basidiocarps orange to dark orange when fresh, of irregular shapes. Basidiospores < 11 × 5 μm, 0–1-septate………………………….**“*Cerinomyces*” *canadensis***
28. Basidiocarps yellow when fresh, growing as circular patches that easily coalesce. Basidiospores > 11 × 5 μm, 0–3-septate………..***Dacrymyces confluens*** & ***D. corticioides***

**Table 3:** *Cerinomyces* species with pale-coloured corticioid basidiocarps, arid or rarely slightly gelatinous when wet; *C. fasciculatus fide*Gilbertson & Hemmes (2004). Ster. — maximal sterigmata length. The table is sorted by region and substrate.

Name	Spores	L	W	Q	Basidia	Ster.	Pegs	Substrate	Region
*C. albosporus*	(11.8–)12.0–17.8(–18.0) × (5.0–)5.2–7.0(–7.1)	15.3	6.0	2.6	25–62 × 3–6.5	20	+	Angiosperm	Africa
*C. aculeatus*	(9.7–)10.5–17.2(–17.7) × 4.0–6.5(–7.4)	13.8	5.0	2.8	12–46 × 3–7	29	+	Gymnosperm, angiosperm?	Asia
*C. aff. aculeatus 1*	9.5–11.3(–11.5) × (3.2–)3.3–4.4(–4.5)	10.5	3.7	2.8	11–20 × 3–4.5	23	−	Gymnosperm	Asia
*C. curvisporus*	(16–)16.3–20.0(–20.4) × 5.9–7.1(–7.5)	18.4	6.5	n/d	41–77 × 6–10	34	+	Gymnosperm	Asia
*C. nepalensis*	(5.0–)5.2–7.7(–7.9) × (2.1–)2.5–3.4(–3.5)	6.6	3.0	2.2	7–17 × 2.5–4.5	10	−	Gymnosperm?	Asia
*C. pinguis*	(8.7–)9.0–10.5(–12.6) × 3.5–4.5(–5.0)	9.8	3.9	2.5	10–21 × 4.5–6.5	12	−	Gymnosperm?	Asia
*C. borealis*	(6.2–)7.1–11.1(–12) × (2.2–)2.3–3.4(–3.6)	9.1	2.9	3.2	9–21 × 3–6	14	−	Gymnosperm	Europe
*C. volaticus*	(6.1–)6.5–9.3(–10.1) × (2.5–)2.6–3.7(–3.9)	7.9	3.1	2.5	10–26 × 2–5	17	−	Gymnosperm	Europe
*C. inermis*	(8.1–)8.2–10.2(–10.6) × (2.9–)3.0–3.9	9.5	3.3	2.9	12–25 × 3–5	21	−	Gymnosperm	New Zealand
*C. verecundus*	(7.9–)8.0–10.1(–10.5) × (3.0–)3.1–4.5(–4.8)	9.2	3.8	2.4	12–25 × 2.5–4.5	15	−	Gymnosperm	New Zealand
*C. favonius*	(6.3–)7.1–10.3(–10.8) × 2.3–3.0(–3.1)	8.3	2.7	3.1	8–18 × 3.5–5	16	−	Gymnosperm	North America
*C. fugax*	(7.2–)7.3–9.3(–10.0) × (2.9–)3.0–3.8(–3.9)	8.5	3.3	2.6	9–22 × 2.5–5	13	−	Gymnosperm	North America
*C. tristis*	6.0–8.9(–9.7) × (2.5–)2.7–4.0(–4.1)	7.4	3.3	2.2	9–22 × 2.5–5	16	−	Gymnosperm	North America
*C. pallidus*	(6.1–)6.5–10.2(–11.3) × 2.9–4.1(–4.8)	8.0	3.3	2.4	10–27 × 2.5–5	19	+/−	Angiosperm, rarely gymnosperm	North America
*C. atrans*	(6.7–)6.9–10.8(–11.1) × (2.3–)2.4–3.9(–4.0)	8.8	3.1	2.9	13–24 × 3–4.5	21	−	Angiosperm, rarely gymnosperm?	North America
*C. fasciculatus*	10.0–12.5(–13) × 4.5–5.5	n/d	n/d	n/d	25–40 × 4–6	35	+	Angiosperm	Oceania
*C. ramosissimus*	(7.0–)7.3–9.6 × (2.7–)2.8–3.2(–3.4)	8.3	3.0	2.8	8–16 × 3–5	11	−	Gymnosperm	South & Central America
*C. brevisetus*	9.3–11.1(–11.7) × 3.5–4.4(–4.6)	10.3	3.9	2.6	12–23 × 3–5.5	19	+	Angiosperm?	South & Central America
*C. concretus*	(7.7–)7.8–10.5(–12.0) × (3.3–)3.4–4.5(–5.0)	9.0	4.0	2.2	15–28 × 3–6	11	−	Angiosperm?	South & Central America
*C. paulistanus*	(6.0–)6.5–9.6(–11.2) × (2.5–)2.6–3.9(–4.4)	7.6	3.3	2.3	10–23 × 2.5–5	13	+/−	Angiosperm?	South & Central America

**Table 4:** *Cerinomyces* species with pustulate, pulvinate, or resupinate basidiocarps, fully gelatinous in moist conditions. Sept. — number of septa in basidiospores, Ster. — maximal sterigmata length. The table is sorted by region and substrate.

Name	Spores	L	W	Q	Sept.	Basidia	Ster.	Substrate	Region
*C. enterolaxus*	9.1–13.7(–14.1) × (3.0–)3.2–4.5(–4.6)	11.6	3.9	3.0	0(–1)	14–33 × 3–6	19	Gymnosperm, angiosperm	Asia
*C. enatus*	(7.0–)7.4–13.2(–14.2) × (2.7–)2.9–4.5(–5.4)	9.5	3.5	2.7	0(–1)	13–56 × 3–6.5	27	Angiosperm, gymnosperm	Asia, North America
*C. creber*	(7.4–)7.7–11.0(–12.8) × (2.5–)2.8–4.0(–4.2)	9.4	3.3	2.9	0	14–36 × 3–5	18	Gymnosperm	Europe
*C. lipoferus*	(9.1–)9.5–15.2(–16.8) × (3.7–)3.9–5.2(–5.7)	11.8	4.5	2.6	0(–1)	19–62 × 3–6	34	Gymnosperm	Europe
*C. neuhoffii*	(8.2–)9.6–14.0(–16.1) × (2.9–)3.0–4.8(–5.1)	11.6	3.8	3.1	0	14–43 × 3–7	26	Gymnosperm	Europe
*C. tortus*	(9.0–)9.7–14.4(–15.6) × (3.0–)3.1–4.9(–5.3)	12.1	4.0	3.0	0	17–56 × 2.5–5	31	Gymnosperm	Europe
*C. aff. tortus 1*	(8.2–)9.0–12.0(–12.4) × 3.0–4.1(–4.4)	10.4	3.6	2.9	0	11–32 × 2.5–4.5	24	Gymnosperm	Europe
*C. aff. tortus 2*	7.8–10.7 × 3.3–4.2(–4.4)	9.4	3.8	2.4	0(–1)	21–40 × 2.5–4	31	Gymnosperm	Europe
*C. aeneus*	(7.9–)8.1–11.2(–13.0) × (2.7–)3.0–4.5(–5.0)	9.5	3.8	2.5	0(–1)	16–38 × 2–6	24	Angiosperm	Europe
*C. crustulinus*	(7.4–)7.7–13.4(–14.0) × 2.8–4.0	9.8	3.3	3.0	0	16–38 × 2.5–5	28	Angiosperm	Europe
*C. aff. crustulinus 1*	(8.4–)8.6–10.9(–11.6) × (3.4–)3.4–4.2(–4.2)	9.8	3.7	2.7	0	20–49 × 3–5	23	Angiosperm	Europe
*C. cokeri*	(9.6–)9.7–12.8(–13.0) × (3.4–)3.5–4.6(–5.0)	11.2	4.0	2.8	0(–1)	36–60 × 3–6	34	Gymnosperm	North America
*C. hesperidis*	(9.9–)10.0–12.3(–13.4) × (3.1–)3.2–4.2(–4.5)	11.3	3.7	3.0	0(–1)	23–41 × 2.5–4.5	35	Gymnosperm	North America

**Table 5:** Species with clampless septa and brightly coloured corticioid basidiocarps, waxy-gelatinous when wet (the *Dacrymyces grandinioides* group). Ster. — maximal sterigmata length. The table is sorted by region and substrate.

Name	Spores	L	W	Q	Basidia	Ster.	Pegs	Substrate	Region
*D. venustus*	(12.8–)13.7–16.2(–16.7) × 4.9–6.7(–6.8)	14.8	5.5	2.7	33–59 × 2.5–6	46	+	Gymnosperm	Africa
*D. aff. venustus 1*	(13.1–)13.9–18.0(–18.2) × (4.9–)5.0–6.2(–6.3)	15.4	5.6	2.8	17–60 × 3–6	34	+	n/d	Africa
*D. grandinioides*	(11.3–)12.0–16.5(–18.3) × (4.3–)4.6–6.5(–6.9)	14.3	5.5	2.6	23–48 × 2.5–5	46	+	Angiosperm, gymnosperm	Africa
*D. burdsallii*	(11.6–)11.9–15.0 × (4.8–)4.9–6.1(–6.3)	13.4	5.3	2.5	24–48 × 2.5–6	48	+	Gymnosperm	North America
*D. grandii*	(13.2–)13.6–16.3(–16.4) × (4.8–)5.0–6.2(–6.8)	14.9	5.5	2.7	20–60 × 3–6	51	+	Gymnosperm	North America
*D. ceraceus*	(11.9–)12.0–16.5(–16.6) × (4.3–)4.4–6.0	13.7	5.1	2.7	25–46 × 3.5–6.5	27	+	Angiosperm	North America
*D. sobrius*	11.0–17.1 × (4.7–)4.9–6.2(–6.3)	13.5	5.4	2.5	21–52 × 3–7	38	+	Angiosperm	North America
*D. cereus*	(9.7–)10.4–13.9(–14.0) × (4.0–)4.1–5.9(–6.1)	12.0	4.9	2.5	16–45 × 3–7	45	−	n/d	South America
*D. lagerheimii*	(12.6–)12.8–17.6(–18.5) × (3.5–)5.0–7.1(–7.6)	14.5	6.0	2.4	15–64 × 3–8	67	−	Angiosperm	South America
*D. pulchrus*	(13.8–)14.1–19.4(–20.6) × (4.5–)4.8–6.4(–6.6)	16.3	5.4	3.0	14–65 × 3.5–8	54	−	Angiosperm?	South America

#### Taxa descriptions

***Cerinomycetaceae*** Jülich, Bibliotheca Mycologica 85: 358 (1981).  

A monotypic family in the class *Dacrymycetes*, consists of the genus *Cerinomyces*.  

***Cerinomyces*** G.W. Martin, Mycologia 41: 82 (1949).  

*Typus*: *Cerinomyces pallidus* G.W. Martin, Mycologia 41: 83 (1949).  

*Description*: *Basidiocarps* either arid and corticioid or gelatinous and pustulate, pulvinate, and resupinate; colour from white to ochraceous, pale yellow, brown, and dark brown. Basidiocarps monomitic, consist of hymenium and supporting structure (subiculum), some species have a distinct margin. Hymenium amphigenous, hymenial surface either smooth or in some corticioid species bears hyphal pegs. *Hyphae* transparent, smooth, hyphal width and wall thickness can vary inside a single basidiocarp, wall gelatinization occurs in many species, gelatinous layer sometimes roughened. Clamps present on each septum except occasional clampless secondary septa inside basidia. Clamps mostly simple, loop-like clamps can occur in subicular areas. *Hymenium* includes basidia and, in some species, *hyphidia* with thickened base and thinner cylindrical apical part, which is either branched or simple. Hyphidia of the second type tend to organize in *hyphal pegs*. Mature *basidia* long-clavate, with two subulate sterigmata. Basidia occasionally bear one or rarely three sterigmata. *Basidiospores* cylindrical to curved-cylindrical, aseptate or rarely with up to three transverse septa in few species, walls thin and smooth, contents usually hyaline, germination with germ tubes or subglobose to cylindrical conidia.  

*Habitat*: decayed, usually decorticated wood of gymnosperm and angiosperm trees and shrubs. According to our observations, cause brown rot.  

*Distribution*: Occur worldwide in forested areas, as follows (informal taxa not listed). Africa: *C. albosporus*. Asia: *C. aculeatus*, *C. curvisporus*, *C. enatus*, *C. enterolaxus*, *C. nepalensis*, *C. pinguis*. Australia and Oceania: *C. fasciculatus*, *C. inermis*, *C. verecundus*. Europe: *C. aeneus*, *C. borealis*, *C. creber*, *C. crustulinus*, *C. lipoferus*, *C. neuhoffii*, *C. tortus*, *C. volaticus*. North America: *C. atrans*, *C. cokeri*, *C. enatus*, *C. favonius*, *C. fugax*, *C. hesperidis*, *C. pallidus*, *C. tristis*. South and Central America: *C. brevisetus*, *C. concretus*, *C. paulistanus*, *C. ramosissimus*.  

**Fig. 9:**
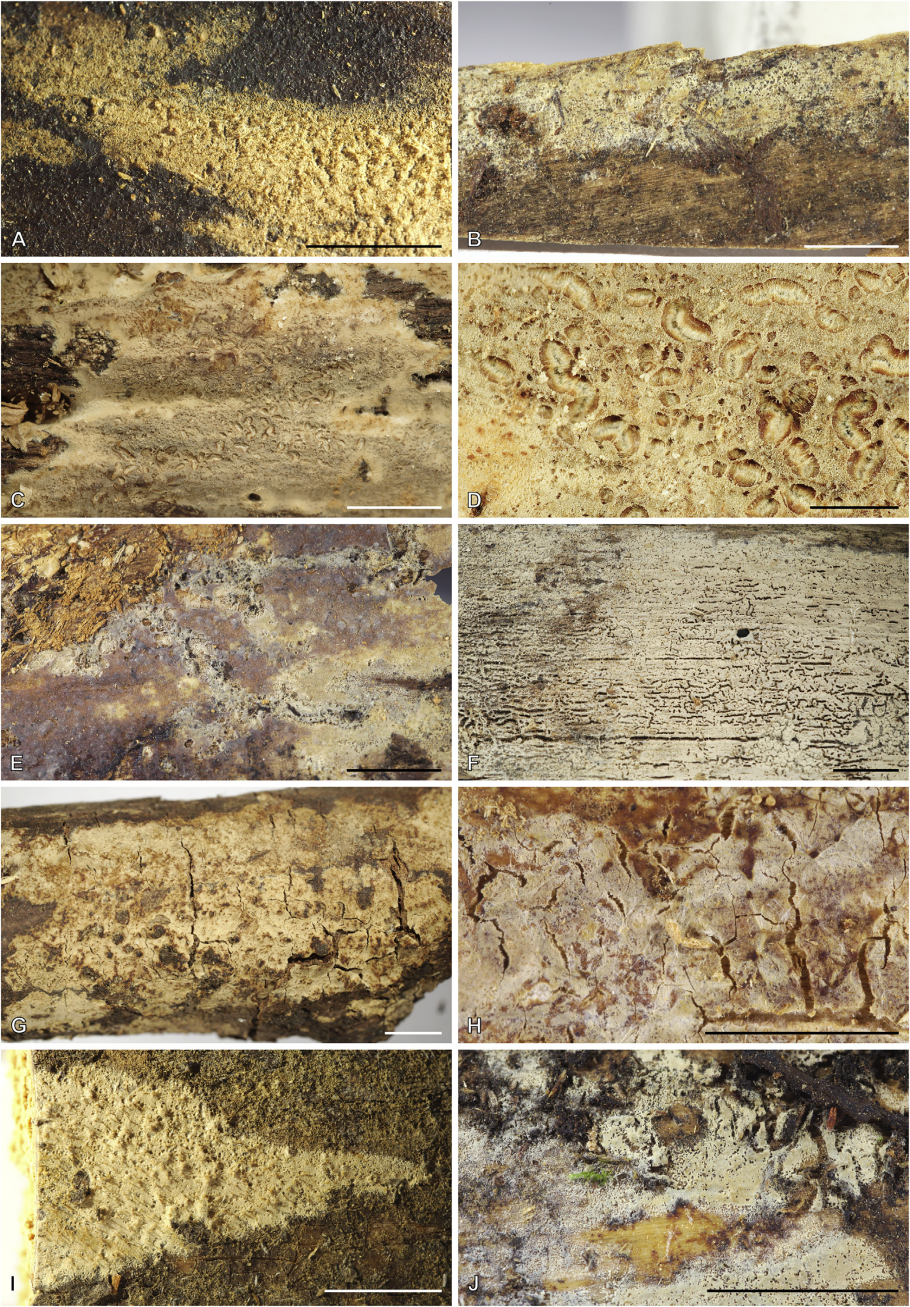
*Cerinomyces* species with arid corticioid basidiocarps. **A.***C. aculeatus* (holotype, TUMH61942). **B.***C. aff. aculeatus 1* (TNM:F16565). **C.***C. albosporus* (holotype, LY11692). **D.***C. albosporus*, cracks in basidiocarp showing dark hymenium and light subiculum (same specimen). **E.***C. atrans* (holotype, GB-0071218). **F.***C. borealis* (holotype, O160848). **G**. *C. brevisetus* (holotype, URM: Chikowski 1544). **H**. *C. concretus* (holotype, O:F-919450). **I**. *C. curvisporus* (isotype, TNS-F-11473). **J**. *C. favonius* (holotype, H7008893). Scale bars: A–C, E–J = 5 mm; D = 1 mm.

***Cerinomyces aculeatus*** N. Maek., Canadian Journal of Botany 65 (3): 583 (1987). Fig. 9, Fig. 16.

**Fig. 10:**
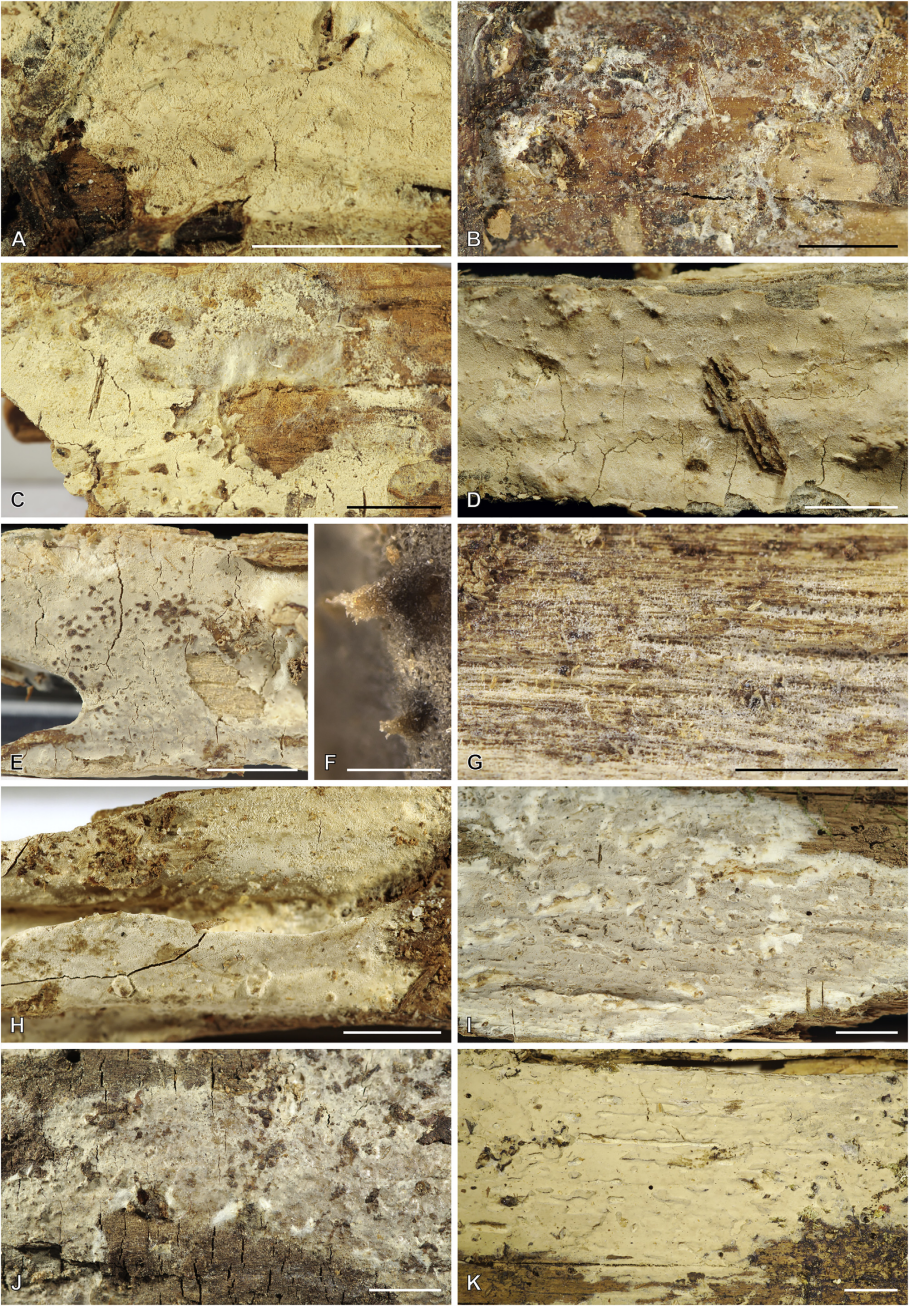
*Cerinomyces* species with arid corticioid basidiocarps. **A.***C. fugax* (holotype, CFMR:HHB-8856). **B.***C. inermis* (holotype, PDD87816). **C.***C. nepalensis* (holotype, O:F-904088). **D**. *C. pallidus* (isotype, NY02136493). **E.***C. paulistanus* (holotype, O:Ryvarden 24759). **F**. *C. paulistanus*, hyphal pegs (same specimen). **G.***C. pinguis* (holotype, O:F-904085). **H.***C. ramosissimus* (holotype, CFMR:FP-150848). **I.***C. tristis* (holotype, H7009711). **J.***C. verecundus* (holotype, PDD93708). **K.***C. volaticus* (holotype, S:F250344). Scale bars: A–E, H–K = 5 mm; F = 250 μm; G = 2.5 mm.

**Fig. 11:**
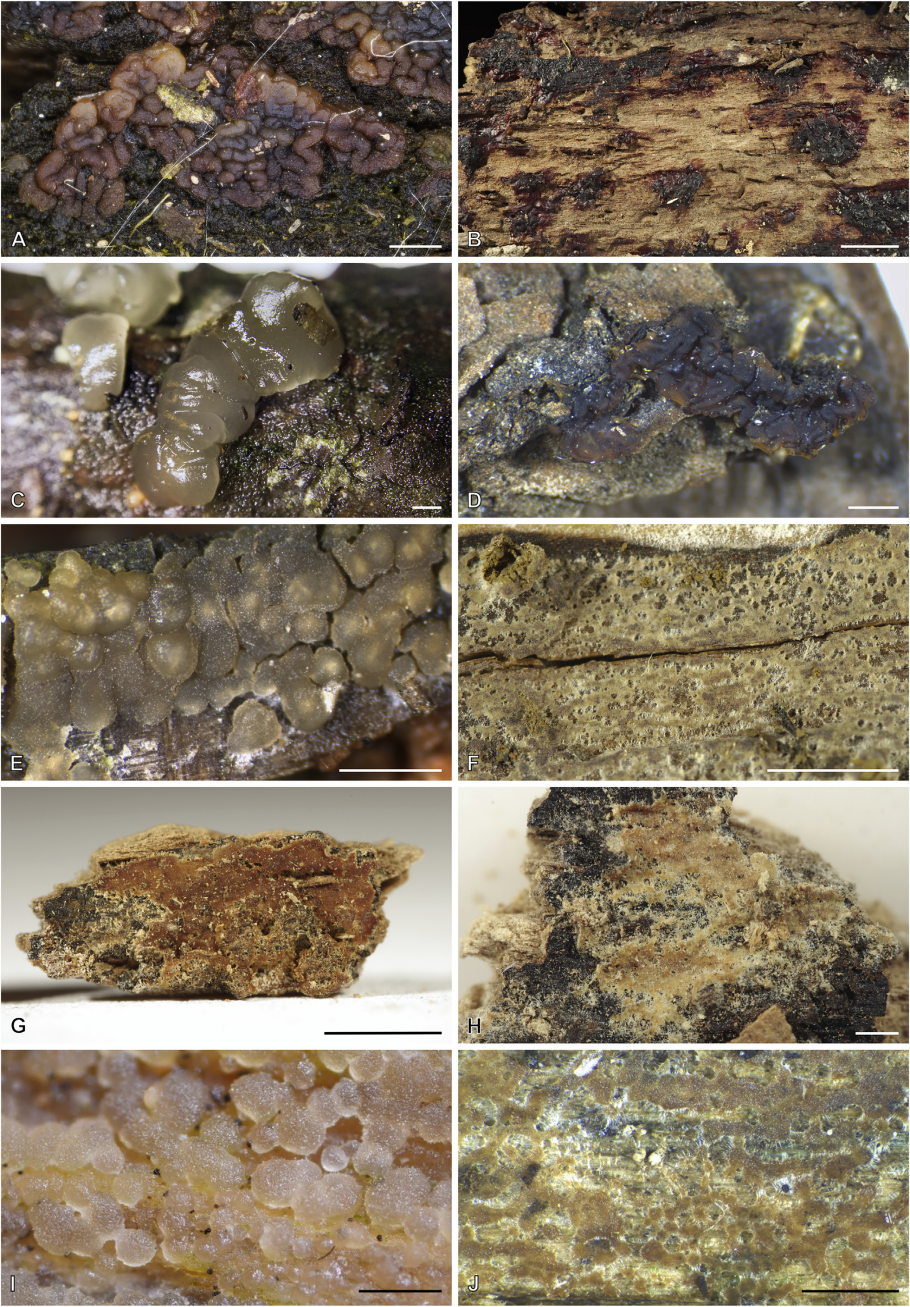
*Cerinomyces* species with gelatinous basidiocarps in rewetted and dry state. **A.***C. aeneus*, rewetted (TU135065). **B.***C. aeneus*, dry (holotype, H7009708). **C.***C. cokeri*, colourless morph, fresh (TU135089). **D.***C. cokeri*, dry (holotype, NCU-F-0031543). **E.***C. creber*, rewetted (UPS:F-946507). **F.***C. creber*, dry (H:Trichies 07077). **G**. *C. crustulinus*, dry, well-developed basidiocarp (lectotype, PC0706688). **H.***C. crustulinus*, dry, young basidiocarps (isolectotype, BPI726061). **I.***C. aff. crustulinus 1*, rewetted, (UPS:F-958851). **J.***C. aff. crustulinus 1*, dry (same specimen). Scale bars: A, C, D, E, H, I = 1 mm; B, F, G, J = 5 mm.

**Fig. 12:**
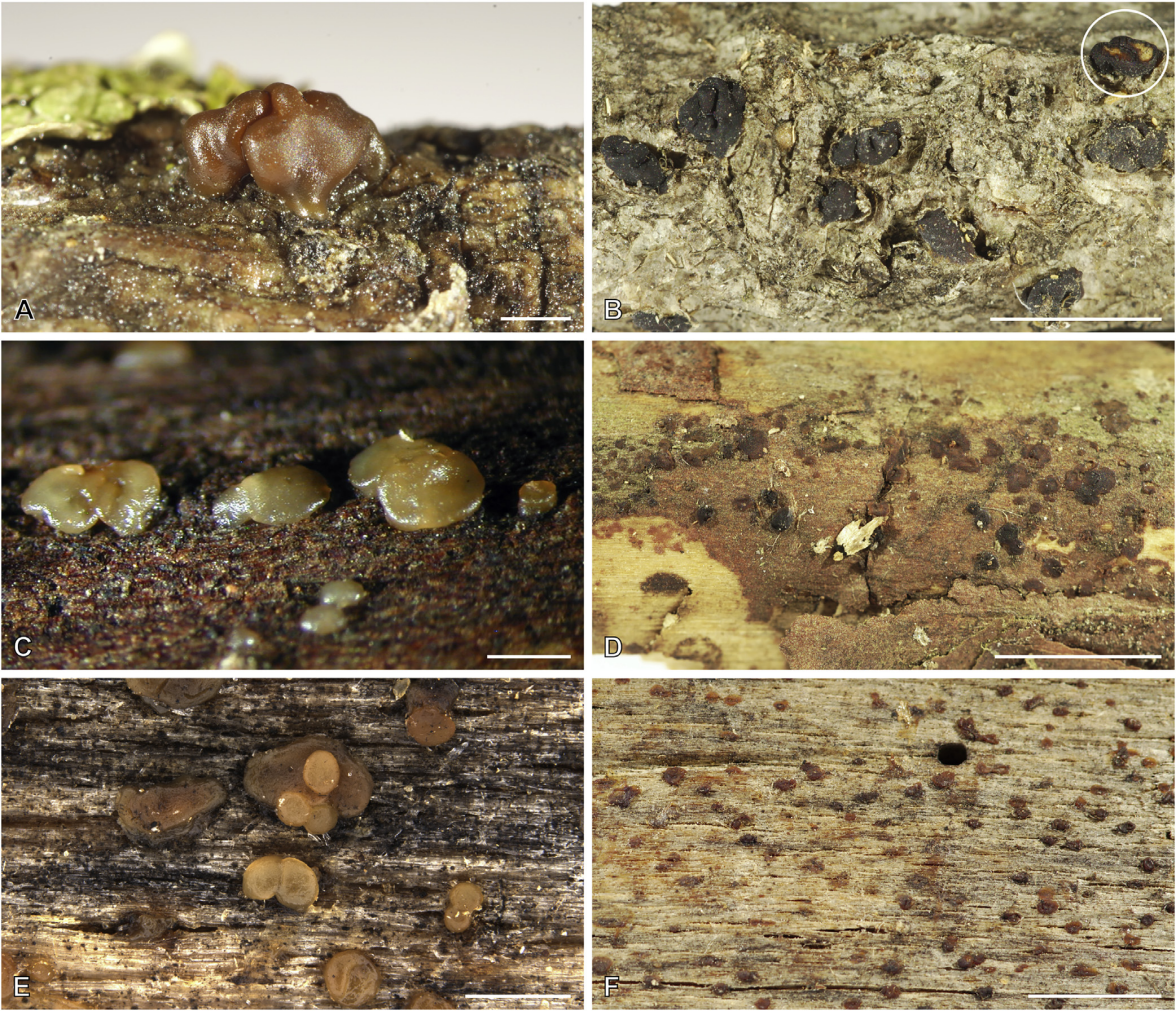
*Cerinomyces* species with gelatinous basidiocarps in rewetted and dry state. **A.***C. enatus*, rewetted (H:Spirin 7774). **B.***C. enatus*, dry (same specimen), highlight: dissected basidiocarp with white core exposed. **C.***C. enterolaxus*, rewetted (TNS-F-15723). **D.***C. enterolaxus*, dry (TNS-F-61319). **E.***C. hesperidis*, rewetted (holotype, NY01782362). **F.***C. hesperidis*, dry (same specimen). Scale bars: A, C, E = 1 mm; B, D, F = 5 mm.

**Fig. 13:**
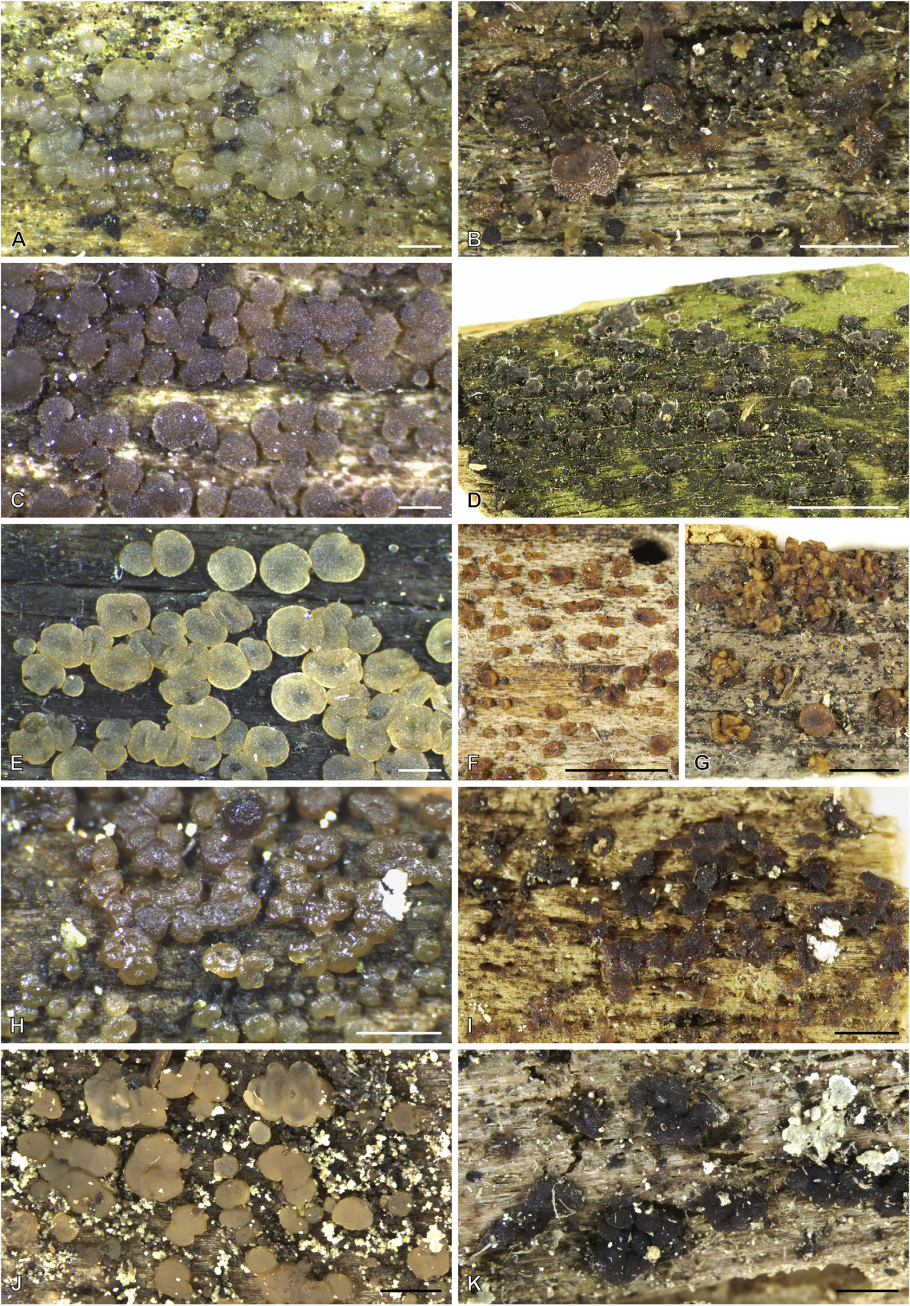
*Cerinomyces* species with gelatinous basidiocarps in rewetted and dry state. **A.***C. lipoferus*, rewetted (holotype, UPS:F-940777). **B.***C. lipoferus*, dry (same specimen). **C.***C. neuhoffii*, rewetted (holotype, UPS:F-941020). **D.***C. neuhoffii*, dry (same specimen). **E.***C. tortus*, rewetted (neotype, UPS:F-946515). **F**, **G**. *C. tortus*, dry, young and mature basidiocarps (holotype of *Dacrymyces punctiformis*, S:F-015301). **H.***C. aff. tortus 1*, rewetted (UPS:F-946504). **I.***C. aff. tortus 1*, dry (same specimen). **J.***C. aff. tortus 2*, rewetted (UPS:F-940948). **K**. *C. aff. tortus 2*, dry (same specimen). Scale bars: A, C, E, H–K = 1 mm; B, D, F, G = 5 mm.

**Fig. 14:**
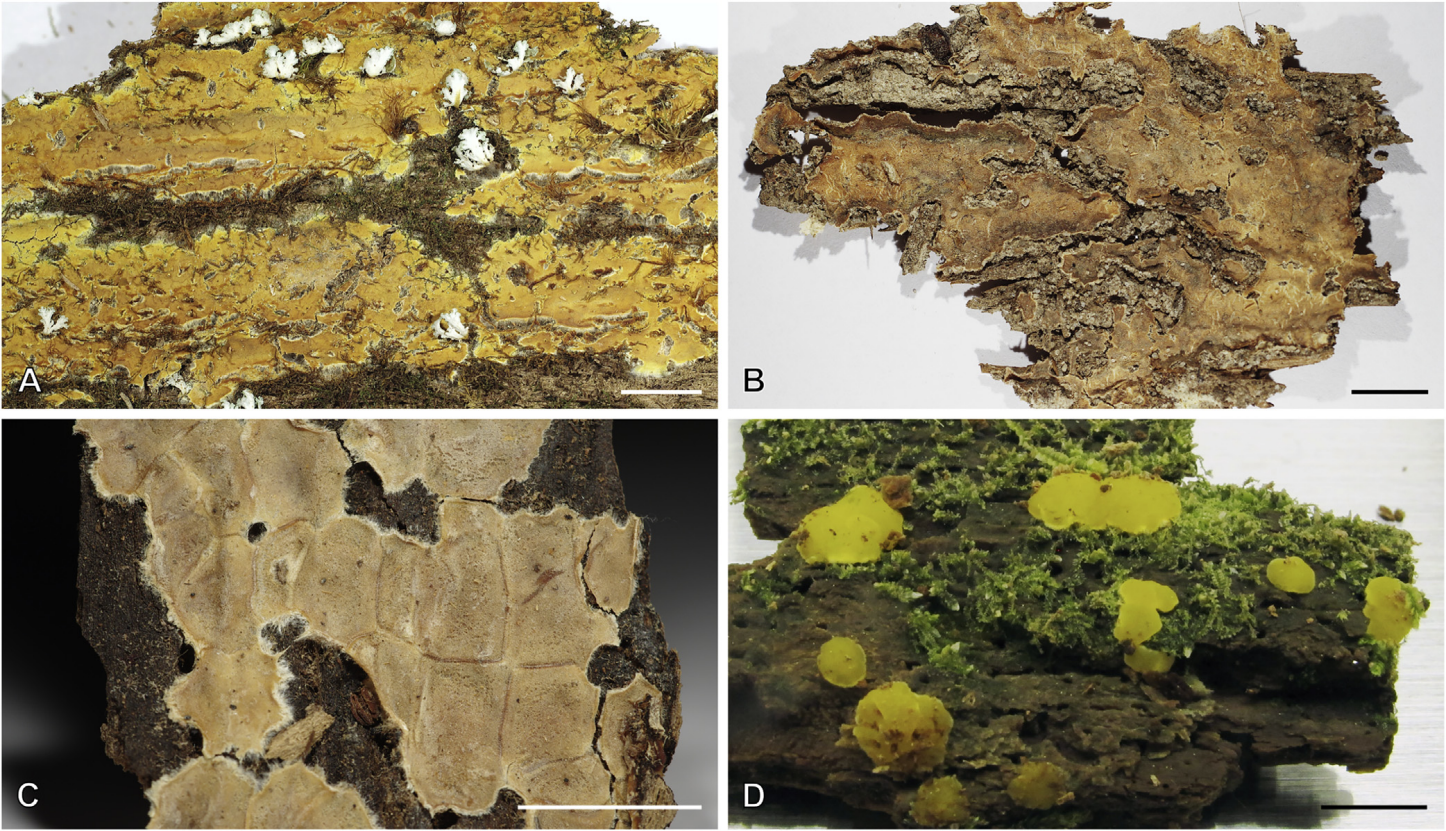
Basidiocarps of corticioid species related to *Femsjonia*, in dry state unless otherwise specified. **A.** “*Cerinomyces*” *canadensis* (H:Spirin 8468). **B.***Dacrymyces confluens* (lectotype of *Ceracea aureofulva*, H7009712). **C.***D. corticioides* (syntype, NY00738305). **D.***D. corticioides*, fresh (holotype of *Femsjonia uniseptata*, TNS-F-54019). Scale bars = 5 mm.

**Fig. 15:**
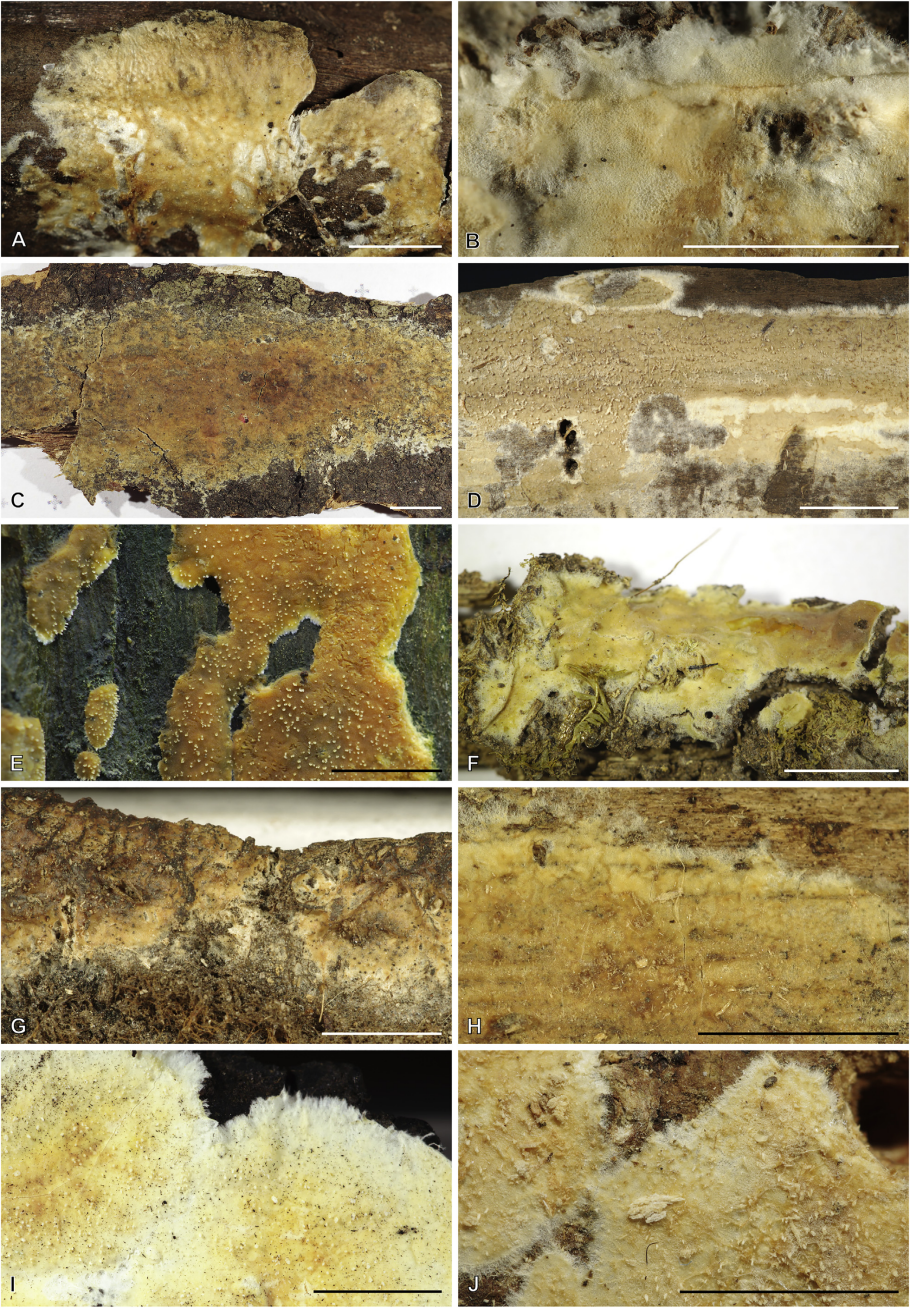
Basidiocarps of *Dacrymyces* species from the *D. grandinioides* group, in dry state unless otherwise specified. **A.***D. burdsallii* (holotype, CFMR:HHB-6908). **B**. *D. ceraceus* (holotype, CFMR:HHB-8969). **C.***D. cereus* (lectotype, FH00304801). **D.***D. grandii* (holotype, NCSLG21158). **E.***D. grandinioides*, in nature (H7008841). **F.** *D. lagerheimii*, partially rewetted (S:F19444). **G.***D. pulchrus* (holotype, LSU00135939). **H.***D. sobrius* (holotype, CFMR:RLG-13487). **I**. *D. venustus* (holotype, O:Adane 150). **J.***D. aff. venustus 1* (LY7839). Scale bars = 5 mm.

**Fig. 16:**
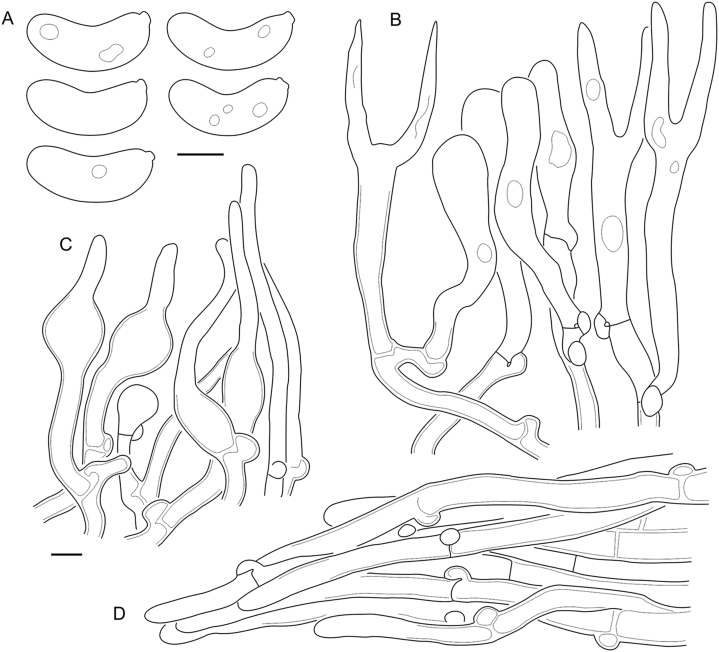
*Cerinomyces aculeatus* micromorphology. **A.** Spores. **B.** Hymenium and subhymenium. **C.** Group of hyphidia organizing in a microscopic peg. **D**. Apical part of a hyphal peg. Drawn from holotype, TUMH61942 (A); TU135070 (B, D); TNS-F-15706 (C). Scale bars = 5 μm.

*Typus*: **Japan**, Honshu, Chūgoku reg., Tottori pref., Kokufu-cho, Okamasu, on *Pinus densiflora*, 12 Jul. 1984, N. Maekawa (**holotype** TUMH61942! ex TMI794, **ex-type culture** TUFC50098∗).  

*Description*: *Basidiocarps* arid, originate as patches of white mycelium, coalesce into seamless formations up to 5 cm in the longest dimension. Hymenial surface first arachnoid, then solid, light ochraceous to buff; subiculum light ochraceous, cottony; margin similar to subiculum or arachnoid if present. In older areas hymenium cracked, brownish. Basidiocarps covered with *hyphal pegs* up to 300 μm in length. *Hyphae* clamped, gelatinized, in subiculum 2–4(–5) μm in diam, with walls 0.5–1.2 μm in width. Subhymenial hyphae of the same diam, with walls 0.3–0.7 μm in width. Hyphal pegs consist of parallel hyphae, 2–5 μm in diam, walls 0.5–1.5 μm in width, thicker in the base and core of peg. *Hymenium* composed of basidia and hyphidia. *Hyphidia* with thick-walled clavate base up to 11 μm in diam and simple cylindrical thin-walled apical part; occasional or in groups developing into hyphal pegs. *Basidia* clavate, 12–46 × 3–7 μm, with sterigmata up to 29(–66) μm in length (n = 115/4), basidial walls sometimes thickened at the base. *Basidiospores* slightly curved-cylindrical, 0–1(–3)-septate, (9.7–)10.5–17.2(–17.7) × 4.0–6.5(–7.4) μm, L = 13.8 μm, W = 5.0 μm, Q = 2.8, Q’ = 1.7–3.5 (n = 121/4), walls ∼ 0.2–0.3 μm in width.  

*Habitat and distribution*: Gymnosperm (*Pinus*) and perhaps angiosperm wood; East Asia.  

*Material examined*: **Japan**, Honshu, Chūbu reg., Nagano pref., Sugadairakougen, on *Pinus densiflora*, 18 Aug. 2006, T. Shirouzu HNo.478 (TNS-F-21063); Kansai reg., Kyoto pref., Mt. Daimonji, on *P. densiflora*, 20 Apr. 2006, T. Shirouzu HNo.191 (TNS-F-15706∗). **Russia**, Primorsk reg., Kedrovaya Pad Nature Reserve, on angiosperm wood (?), Jun. 2016, I. Viner КЮН1422 (reads “KUN1422”, H7008652), КЮН2396 (TU135069∗), КЮН2417 (TU135070∗).  

*Notes*: The species was synonymized with *C. albosporus* shortly after description (Maekawa & Zang 1997). The two species are related, but clearly different in the ITS sequences, basidiocarp appearance, spore size, and distribution. Maekawa (1987) provided a detailed study on the cultural characteristics of the species, showing that young basidiospores are binucleate, and when deposited on MEA, aseptate basidiospores develop one to four transverse septa before or rarely after germination. He also described curved-cylindrical conidia 3.6–5.6 × 1.2–1.6 μm produced from hyphae in monokaryotic culture and pictured basidiospores with conidiogenous scars. Shirouzu *et al.* (2009) reported subglobose conidia born from basidiospores, measuring 6 × 3 μm.  

**Fig. 17:**
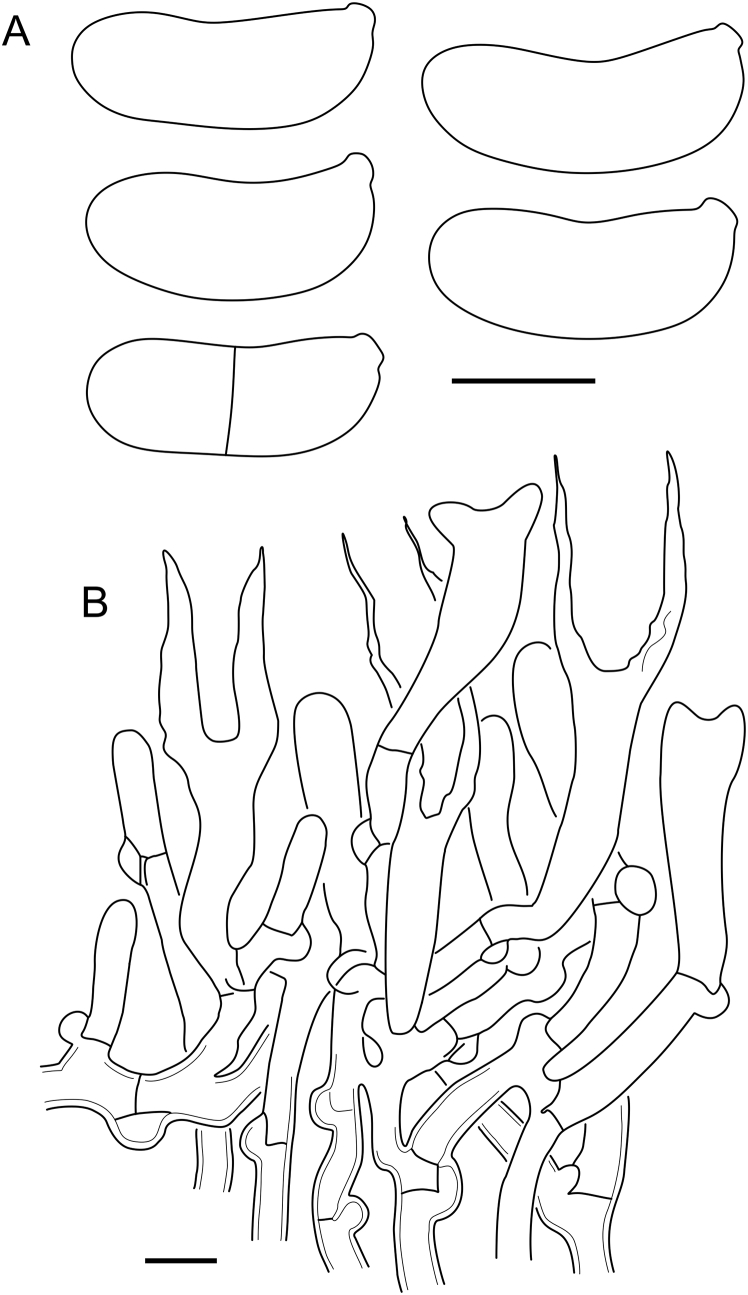
*Cerinomyces aff. aculeatus 1* micromorphology. **A.** Spores. **B**. Hymenium and subhymenium. All drawn from TNM:F16565. Scale bars = 5 μm.

***Cerinomyces aff. aculeatus 1***. Fig. 9, Fig. 17.

*Description*: *Basidiocarps* at first arachnoid, then turning into smooth brownish thin film, waxy and semitransparent when wet, in better developed (or preserved) areas more arid, solid and light ochraceous. Hyphal pegs absent. *Hyphae* clamped, subicular hyphae 2–4 μm in diam, walls 0.3–0.5 μm in width; in subhymenium 2–3 μm in diam, walls 0.3–0.4 μm in width. *Hymenium* simple, *basidia* clavate, 11–20 × 3–4.5 μm, with sterigmata up to 23 μm in length (n = 30/1). *Basidiospores* cylindrical, slightly curved, 0–1-septate, 9.5–11.3(–11.5) × (3.2–)3.3–4.4(–4.5) μm, L = 10.5 μm, W = 3.7 μm, Q = 2.8, Q’ = 2.4–3.3 (n = 30/1), walls ∼ 0.2–0.3 μm in width. Basidiospores produce cylindrical conidia 2.2–2.6 × 1.2–1.3 μm.  

*Habitat and distribution*: Gymnosperm wood (*Pinus*); East Asia.  

*Material examined*: **Taiwan**, Taipei, Peitou dist., on *Pinus luchuensis*, 17 Jul. 1999, R. Kirschner 542 (TNM:F16565∗).  

*Notes*: The taxon belongs to the *C. albosporus* clade by similarity in LSU sequences, but we postpone a formal description until more markers are sequenced or new material found. The most similar LSU sequence was obtained from a Japanese environmental sample 05151-1B2 (Shirouzu *et al.* 2016). For the same Taiwanese material, Kirschner & Yang (2005) reported *sporothrix*-like conidiogenesis in culture, with curved-cylindrical conidia < 5 μm in length.  

**Fig. 18:**
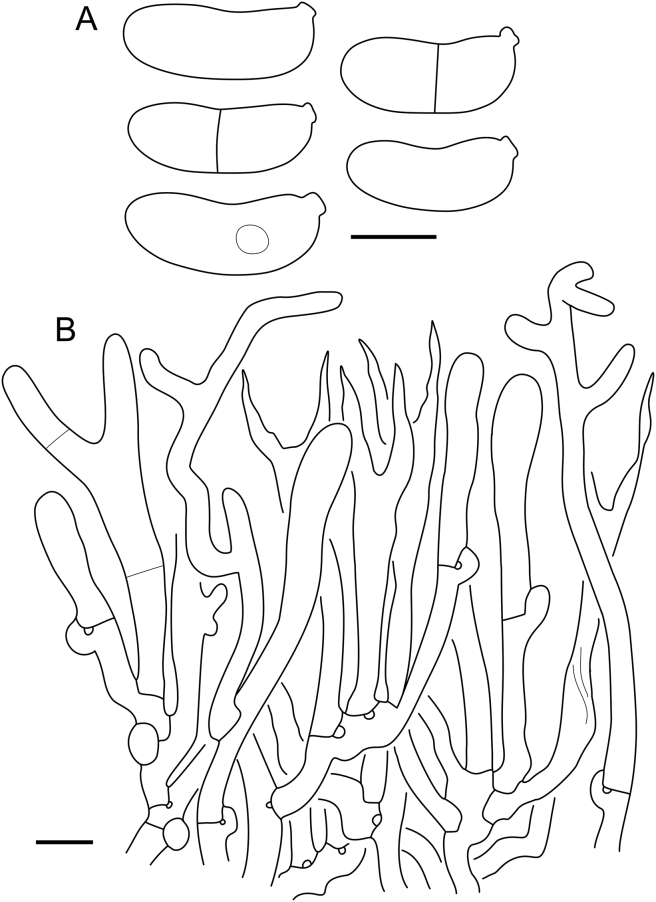
*Cerinomyces aeneus* micromorphology. **A.** Spores. **B**. Hymenium with hyphidia, and subhymenium. Drawn from holotype, H7009708 (A); TU135065 (B). Scale bars = 5 μm.

***Cerinomyces aeneus*** A. Savchenko, Miettinen & J.C. Zamora, ***sp. nov.*** MycoBank MB 839760. Fig. 11, Fig. 18.

*Typus*: **Ukraine**, Transcarpathian reg., Mizhhirya dist., Synevir National Park, 300–400 m to the east from Synevir lake, on *Fagus sylvatica*, 9 Aug. 2010, O. Akulov & A. Ordynets (**holotype** H7009708∗!, **isotypes** CWU(MYC)7500!, UPS!).  

*Etymology*: aeneus (Lat.) — bronze, referring to the colour of basidiocarps.  

*Description*: *Basidiocarps* gelatinous, first pustulate, then coalesce into smooth to densely cerebriform resupinate formations up to several cm in the longest dimension, light brownish yellow at margins, reddish brown in the center, becoming almost black when dried. Some pustulate basidiocarps can remain isolated outside of these formations. *Hyphae* clamped, in subiculum (1.5–)2–3.5 μm in diam, walls 0.3–0.5 μm in width, gelatinized, sometimes roughened, often agglutinated. Subhymenial hyphae 1.5–3 μm in diam, walls ∼ 0.3 μm in width. Marginal hyphae simple cylindrical or slightly clavate up to 4 μm in diam, rarely branched, walls 0.3–0.5 μm in width. *Hymenium* includes abundant branched cylindrical *hyphidia*, in base 2–2.5 μm in diam, with apical part 1–2 μm in diam. *Basidia* clavate, 16–38 × 2–6 μm. Sterigmata up to 24(–35) μm in length (n = 57/3). *Basidiospores* cylindrical to slightly curved-cylindrical, 0(–1)-septate, at least some binucleate, (7.9–)8.1–11.2(–13.0) × (2.7–)3.0–4.5(–5.0) μm, L = 9.5 μm, W = 3.8 μm, Q = 2.5, Q’ = 1.9–3.5 (n = 176/5), walls ∼ 0.2 μm in width.  

*Habitat and distribution*: Angiosperm wood (*Alnus*, *Carpinus*, *Fagus*, *Quercus*, and unident.); Europe.  

*Material examined*: **Czech Republic**, Vysočina reg., Jihlava dist., near Třešť, Velký Špičák Nature Reserve, on *Fagus sylvatica*, 28 Oct. 2011, M. Brom (PRM929903), same loc., on angiosperm wood, 5 Jul. 2011, M. Brom (PRM929897), near Třešť, V Klučí Nature Reserve, loco “V Ohradě”, on angiosperm wood, 17 Nov. 2010, M. Brom (PRM929896), (PRM929907), 27 Oct. 2011 (PRM929891∗); Ústecký reg., Teplice dist., Vlčí důl Nature Reserve, Osek castle, on *F. sylvatica*, 27 Sep. 2014, M. Kříž (PRM934335∗). **Finland**, Uusimaa prov., Helsinki, Vantaanjokivarsi, Patola, on *Alnus incana*, 6 Nov. 2011, O. Miettinen 15065.2 (H6013355∗). **Norway**, Sogn og Fjordane co., Balestrand mun., Suphelledalen in Fjærland, on *Alnus*, 10 Sep. 2000, S. Evans (O146179∗). **Sweden**, Kalmar co., Öland, Mörbylånga mun., Södra Ottenby lund, 100 m SW of St. Finnslätten, on *Quercus robur*, 29 Aug. 2000, T. Knutsson 2000-06 (UPS:F-560919∗); Västra Götaland co., Uddevalla mun., Uddevalla, Rimnersvallen N, on *Q. robur*, 27 Dec. 2017, J. Olsson (UPS:F-946499∗). **Ukraine**, Ternopil reg., Zalishchyky dist., Dnistrovskyi Canyon National Park, between Dnister and Dzhuryn rivers, near Ustechko village, on *Carpinus betulus*, 5 Oct. 2016, V. Hukov 5-30, AS0077 (CWU(MYC), TU135065∗); Zakarpttia reg., Velykyi Bereznyi dist., Uzhanskyi National Park, near to Kaminy mountain, on *F. sylvatica*, 30 Jul. 2014, M. Kit (CWU(MYC)7035).  

*Notes*: The species was traditionally identified as *Dacrymyces enatus* (≡ *C. enatus*). *Cerinomyces aeneus* develops resupinate basidiocarps and grows only on angiosperm, typically decorticated wood in Europe, while *C. enatus* produces mostly pulvinate or rarely resupinate basidiocarps and occurs on both angiosperm and gymnosperm wood with bark in North America and East Asia. *Cerinomyces crustulinus* and *C. aff. crustulinus 1*, similar European taxa growing on angiosperm substrates, have basidiocarps of different, lighter colour. Also, in contrast to *C. enatus*, in these species outlines of separate pustules remain visible even in mature basidiocarps.  

**Fig. 19:**
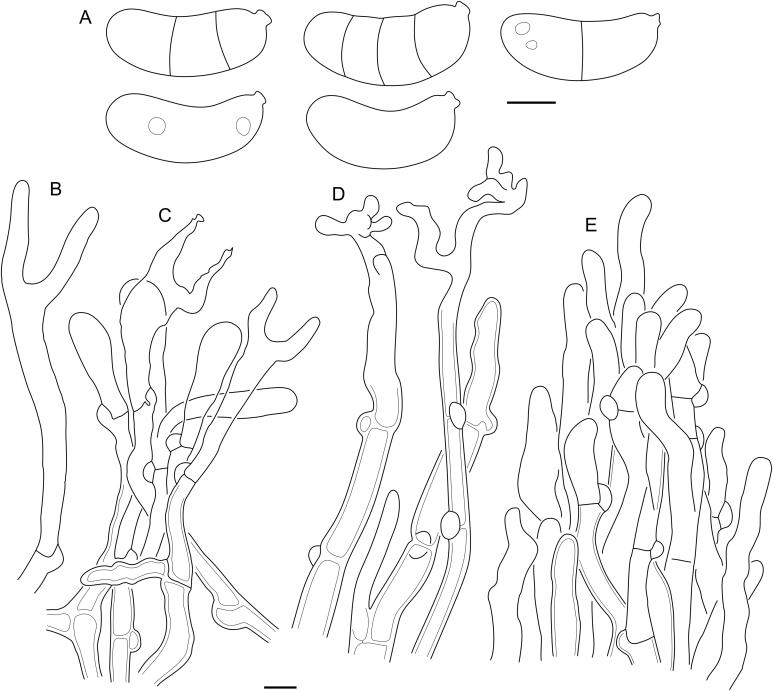
*Cerinomyces albosporus* micromorphology. **A.** Spores. **B.** Single full-sized basidium. **C.** Hymenium with short basidia, and subhymenium. **D.** Hyphidia. **E.** Apical part of a hyphal peg. All drawn from holotype, LY11692. Scale bars = 5 μm.

***Cerinomyces albosporus*** Boidin & Gilles, Bulletin de la Société Mycologique de France 102 (3): 318 (1986). Fig. 9, Fig. 19.

*Typus*: France, **Réunion**, Saint-Benoît dept., Forêt de Bébour, on *Senecio ambavilla,* 8 May 1985, J. Boidin (**holotype** LY11692∗!).  

*Description*: *Basidiocarps* arid, originate as circular patches of white mycelium that develops into thick cottony subiculum. Above the subiculum, hymenial surface arachnoid to solid, light ochraceous to buff, covered with abundant *hyphal pegs* up to 500 μm in height; margin irregular, white, fimbriate. In well-developed areas surface cracks showing darker, brownish hymenium and subhymenium, and lighter subiculum. *Hyphae* clamped, densely packed, in subiculum 2–5 μm in diam, walls 0.5–1.0 μm in width; in subhymenium of the same diam, walls 0.4–0.8 μm in width. Hyphal pegs consist of parallel hyphae, 2.5–3 μm in diam, with wall width of 0.5 μm at the top and sides of peg, thicker at the base and in the core. *Hymenium* composed of basidia and occasional cylindrical *hyphidia*, simple or, rarely, branched at the apical part, walls thin, only thickened at the base; usually occurring in groups that develop into hyphal pegs. *Basidia* clavate, 25–62 × 3–6.5 μm, with sterigmata up to 20 μm in length (n = 30/1), basidial walls sometimes thickened at the base. *Basidiospores* slightly curved-cylindrical, 0–1(–3)-septate, (11.8–)12.0–17.8(–18.0) × (5.0–)5.2–7.0(–7.1) μm, L = 15.3 μm, W = 6.0 μm, Q = 2.6, Q’ = 1.8–3.3 (n = 34/1), spore walls ∼ 0.2–0.3 μm in width. Basidiospores bear cylindrical conidia 3.1–4.1 × 1.8–2.1 μm.  

*Habitat and distribution*: Angiosperm shrub (*Senecio*); Africa (known only from the type locality).  

*Notes*: The species has the most robust basidiocarps and the largest basidiospores among the peg-bearing *Cerinomyces* species. Three-septate basidiospores are extremely rare and most easy to find in substrate scrapes made next to basidiocarps.  

**Fig. 20:**
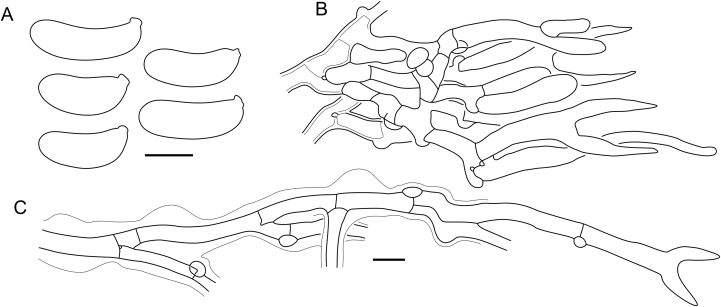
*Cerinomyces atrans* micromorphology. **A.** Spores. **B**. Hymenium and subhymenium. **C.** A single unevenly gelatinized hypha followed from subiculum up to hymenium. Drawn from holotype, GB-0071218 (A, B); GB-0071217 (C). Scale bars = 5 μm.

***Cerinomyces atrans*** A. Savchenko, ***sp. nov.*** MycoBank MB 839782. Fig. 9, Fig. 20.

*Typus*: **Canada**, British Columbia, Greater Vancouver, near the campus of University of British Columbia, on angiosperm wood, 16 Sep. 1982, N. Hallenberg 7309 (**holotype** GB-0071218∗!, **isotype** UBC:F1032!).  

*Etymology*: atrans (Lat.) — darkening; due to a brown colour of well-developed basidiocarp areas.  

*Description*: *Basidiocarps* arachnoid, arid and light ochraceous, in older areas smooth, solid, crustose, slightly waxy when wet, dark ochraceous to brown, especially in prominent or bruised areas; with thin subiculum and indistinct arachnoid margins. *Hyphae* clamped, subicular hyphae 2–3.5 μm in diam, walls ∼ 0.5 μm in width, gelatinous layer up to 4 μm in width; in subhymenium 2–3 μm in diam, walls ∼ 0.3 μm in width. *Hymenium* includes *hyphidia*, simple, cylindrical or with slightly thickened base, with apical part ∼ 2 μm in diam, up to 100 μm in total length; sometimes agglutinated, more abundant closer to margins. *Basidia* clavate, 13–24 × 3–4.5 μm, with sterigmata up to 21 μm in length (n = 40/2). *Basidiospores* cylindrical, slightly curved, aseptate, (6.7–)6.9–10.8(–11.1) × (2.3–)2.4–3.9(–4) μm, L = 8.8 μm, W = 3.1 μm, Q = 2.9, Q’ = 2.3–3.5 (n = 53/2), walls ∼ 0.2 μm in width.  

*Habitat and distribution*: Angiosperm (*Alnus* and unident.) and possibly gymnosperm wood; Western North America.  

*Material examined*: **Canada**, British Columbia, Vancouver Island, Juan de Fuca reg., Fairy lake near Port Renfrew, 12 Aug. 1988, N. Hallenberg 10674 (GB-0071217∗); British Columbia, Vancouver Island, Mesachie lake, on *Alnus*, 5–6 Aug. 1982, N. Hallenberg 7069 (GB-0180499∗). **USA**, Oregon, between Sweet Home and Cascadia, on gymnosperm wood, 20 Nov. 1937, A.M. Rogers & D.P. Rogers (NY: Herb. D.P. Rogers 396), Cascade Head Expt. Forest, Siuslaw National Forest, on *Alnus*, 11 Oct. 1972, M.J. Larsen (CFMR:FP-133356).  

*Notes*: Older, prominent, and damaged areas of the studied basidiocarps have dark brown tints, but we suppose this character can be absent in young fungi. Compared to *C. pallidus*, another North American species inhabiting mostly angiosperm wood, *C. atrans* does not produce hyphal pegs visible to the naked eye, but only microscopic aggregations of hyphidia. When dealing with occurrences on gymnosperm wood, a great attention is needed to distinguish between *C. atrans–C. pallidus* and exclusively conifers-inhabiting North American species. However, basidiocarps of the latter group do not turn into darkened crust so easily, instead remaining arachnoid or firm-cottony.  

 An earlier published ITS sequence of a culture TUFC30545 aligns well with our sequences, though we did not examine its voucher material UBC6108.  

**Fig. 21:**
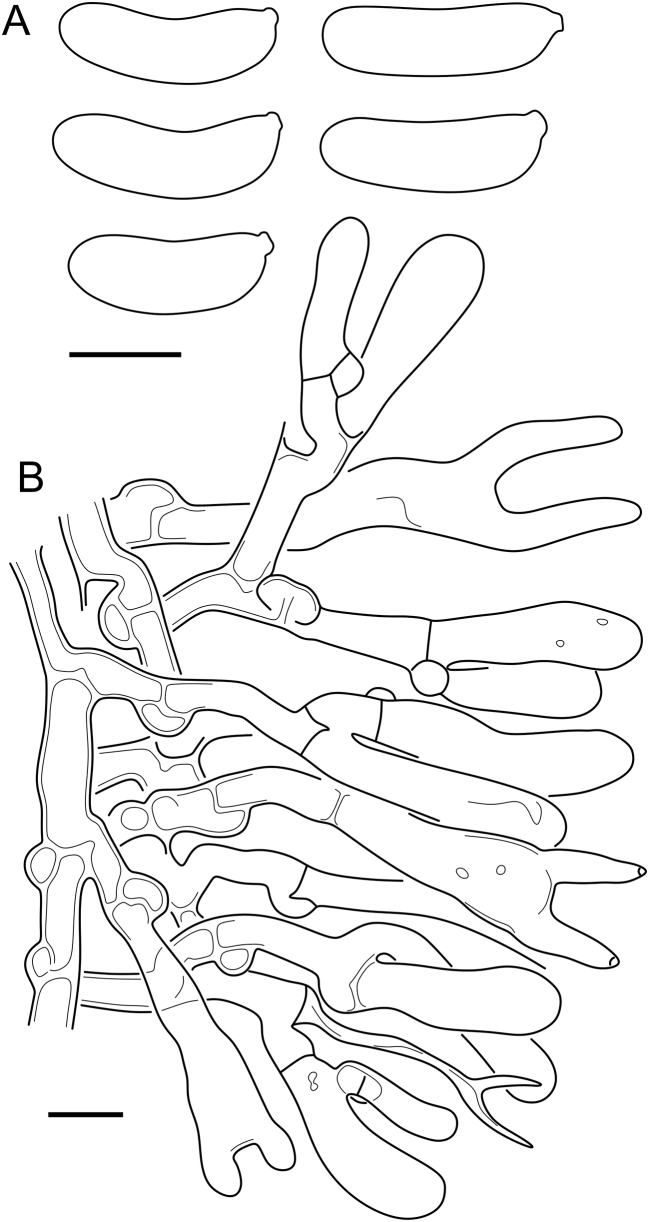
*Cerinomyces borealis* micromorphology. **A.** Spores. **B.** Hymenium and subhymenium. All drawn from holotype, O160848. Scale bars = 5 μm.

***Cerinomyces borealis*** Miettinen, Spirin & A. Savchenko, ***sp. nov.*** MycoBank MB 839783. Fig. 9, Fig. 21.

*Typus*: **Norway**, Hedmark co., Løten mun., near Ebru, on gymnosperm wood, 28 Aug. 1976, E. Høgholen 528/76 (**holotype** O160848∗!).  

*Etymology*: borealis (Lat.) — northern.  

*Description*: *Basidiocarps* appear as small patches, then coalesce into arachnoid, arid, at first white then light ochraceous or light grey mats up to 0.3 mm thick. Subiculum thin and indistinct; hymenium at first irregular, but in well-developed basidiocarps becomes smooth and solid; margin indistinct or absent. Dark brown to black underlying layers of old collapsed basidiocarps sometimes present. *Hyphae* clamped, in subiculum 2–4 μm in diam, walls ∼ 0.5 μm in width, with gelatinous layer up to 1 μm; in subhymenium 2–3 μm in diam, with walls ∼ 0.3 μm in width. Swollen up to 10 μm in width, thick-walled cells sometimes occur closer to substrate. Marginal hyphae simple, cylindrical, similar to subicular. *Hymenium* simple, *basidia* clavate, 9–21 × 3–6 μm, with sterigmata up to 14 μm in length (n = 46/5). Sometimes young basidioles are distinctly clavate. *Basidiospores* cylindrical, slightly curved, aseptate, (6.2–)7.1–11.1(–12.0) × (2.2–)2.3–3.4(–3.6) μm, L = 9.1 μm, W = 2.9 μm, Q = 3.2, Q’ = 2.2–3.9 (n = 112/5; 31 measured in 1 % KOH), walls ∼ 0.2 μm in width.  

*Habitat and distribution*: Gymnosperm wood (*Picea*, *Pinus*, and unident.); Northern Europe.  

*Material examined*: **Finland**, Etelä-Häme prov., Padasjoki mun., Vesijako Nature Reserve, on *Picea abies*, 2 Oct. 1984, K. Hjortstam 14504 (GB-0071224); Pohjois-Häme prov., Jyväskylä mun., Vuoritsalo, on *Pinus sylvestris*, 22 Jul. 2010, O. Miettinen 14094 (H6012704∗), Saarijärvi mun., Pyhä-Häkki National Park, on *Pic. abies*, 14 Aug. 2017, O. Miettinen 21156.1 (H∗); Kainuu prov., Kuhmo mun., Kuikkajärvi, Rytiniemi, on *P. sylvestris*, 14 Jun. 2015, H. Kotiranta 26863 (H6055125∗). **Norway**, Hedmark co., Løten mun., near Vesl-Bronken, PN 4232, 17 Sep. 1982, K. Hjortstam 13211 & E. Høgholen (GB-0071222), 13214 (GB-0071223), Stor-Elvdal mun., Fagervoll ved Atnsjøen, on *P. sylvestris*, 21 Aug. 1996, L. Ryvarden 39230 (O101812∗); Møre og Romsdal co., Aure mun., [N of] Lia, on *P. sylvestris*, 1 Jun. 2009, F. Oldervik 006.09 (O288478); Nordland co., Rana mun., Ørtfjellmoen, in Dunderlandsdalen, on *Pic. abies*, 1976, J. Hereng 375 (O101814), Ørtfjellmoen, on *Pic. abies*, 17 Aug. 1982, K.-H. Larsson 2771 (GB-0071221); Oppland co., Gausdal mun., Ormtjernkampen National Park, on *Pic. abies*, 3 Jul. 1975, I. Johansen 686/75 (O101811), Lunner mun., Rinilhaugen Nature Reserve, on *Pic. abies*, 17 Sep. 2016, V. Spirin 11136 (H), Øyer mun., Bårdsengbekken, on *Pic. abies*, 10 Sep. 1979, B. Bakke 1566 (O101809), 1581 (O101808); Oslo co., Sorkedalen, Svartor ved Kjellerberget, on *Pic. abies*, 28–29 Sep. 1977, K. Hjortstam 8929 & K.-H. Larsson (GB-0071220); Sør-Trøndelag co., Hemne mun., Gammelsetra, [up to] Rennsjøen, on *P. sylvestris*, 24 Oct. 2004, F. Oldervik 609.04 (O188052). **Russia**, Leningrad reg., Boksitogorsky dist., Kolp, on *Pic. abies*, 29 Jul. 2016, V. Spirin 10443 (H∗). **Sweden**, Dalarna co., Malung-Sälen mun., Lybergsgnupen, on *Pic. abies*, 7 Oct. 1982, T. Hallingbäck & K.-H. Larsson 3401b (GB-0071196); Jämtland County, Bräcke mun., between Stavre and lake Bodsjön, on gymnosperm wood, 29 Jul. 1958, J. Eriksson 8194 (GB-0071198), 30 Jul. 1958, J. Eriksson 8195 (GB-0071197); Jönköping co., Vaggeryd mun., E of Kacklesjön (NE of Marieholm), on *Pic. abies*, 1 Nov. 1981, T. Hallingbäck (GB-0071192); Norrbotten co., Jokkmokk mun., S of Muddus national park, near Rimojokk, on *P. sylvestris*, 13 Aug. 1958, J. Eriksson 8658 (GB-0071199); Värmland co., Forshaga mun., Pannkakan, on gymnosperm wood, 6 Oct. 1982, T. Hallingbäck & K.-H. Larsson 3226 (GB-0071195); Västerbotten co., Sorsele mun., Grannäs, västra Lairobäcken, on gymnosperm wood, 29 Aug. 1983, K.-H. Larsson 4194 (GB-0071200); Västernorrland co., Sollefteå mun., Junsele parish, Storhögen, S of the national forest area Ulfvik, on *P. sylvestris*, 22 Sep. 1970, K. Hjortstam 4408 (GB-0071213); Västra Götaland co., Alingsås mun., Gräskärr, Simmenäshalvön, on *Pic. abies*, 18 Oct. 1992, K. Hjortstam 17423 (GB-0087175), S of Stora Hyggesjön, on *Pic. abies*, 16 Jul. 1972, K.-H. Larsson 754b (GB-0071204), W side of lake Lille Trän, on *Pic. abies*, 14 Oct. 1971, K.-H. Larsson 545 (GB-0071203), Bengtsfors mun., Tisselskog par., W side of lake Råvarp, close to a small stream, on *Pic. abies*, 30 Sep. 1972, A. Hjortstam & K. Hjortstam 8553 (GB-0071194), Bollebygd mun., Töllsjö, Sjögaredsbergen, on *Pic. abies*, 23 Aug. 1969, K. Hjortstam 1526 (GB-0071202, GB-0071212, TAAM099151), SW from Kolsjöhatt, on *P. sylvestris*, 8 Jul. 1969, K. Hjortstam 1408 (GB-0071208), Borås mun., NE of Hemsjön lake, on *Pic. abies*, 10 Sep. 1969, K. Hjortstam 2115/a (GB-0071207), Grästorp mun., Hunneberg Nature Reserve, Jonstorpsmossen, on *Pic. abies*, 1 Oct. 1978, K.-H. Larsson 2414 (GB-0071211), Karlsborg mun., Undenäs par., "Trollkyrkoreservatet" (Tivedens National Park?), on *Pic. abies*, 19 Aug. 1977, K. Hjortstam 8553 & J. Ginns (O101815).  

*Notes*: *Cerinomyces borealis* is a relatively widespread species in Northern Europe. In Nordic herbaria, the majority of the specimens labelled as *C. crustulinus* belong to *C. borealis* — *e.g.*, GB-0071198, cited as such in “The *Corticiaceae* of North Europe” (Eriksson & Ryvarden 1973). The species can be distinguished from similarly looking *C. volaticus* by longer, thinner basidiospores (mostly ≤ 3.0 μm in width) and the more delicate, arachnoid basidiocarps. Intermediate forms with large basidiospores also occur, but even then, higher Q values of *C. borealis* still hold.  

 In three specimens identified as *C. borealis* chiefly by the thin basidiospores, we found hyphidia: long cylindrical with thickened bases, typical for the *C. pallidus* clade (GB-0071209), and branched, either only slightly (GB-0071221), or with knot-like heads, more characteristic for some gelatinous *Cerinomyces* species (GB-0071210). Sequencing attempts of these specimens were unsuccessful.  

**Fig. 22:**
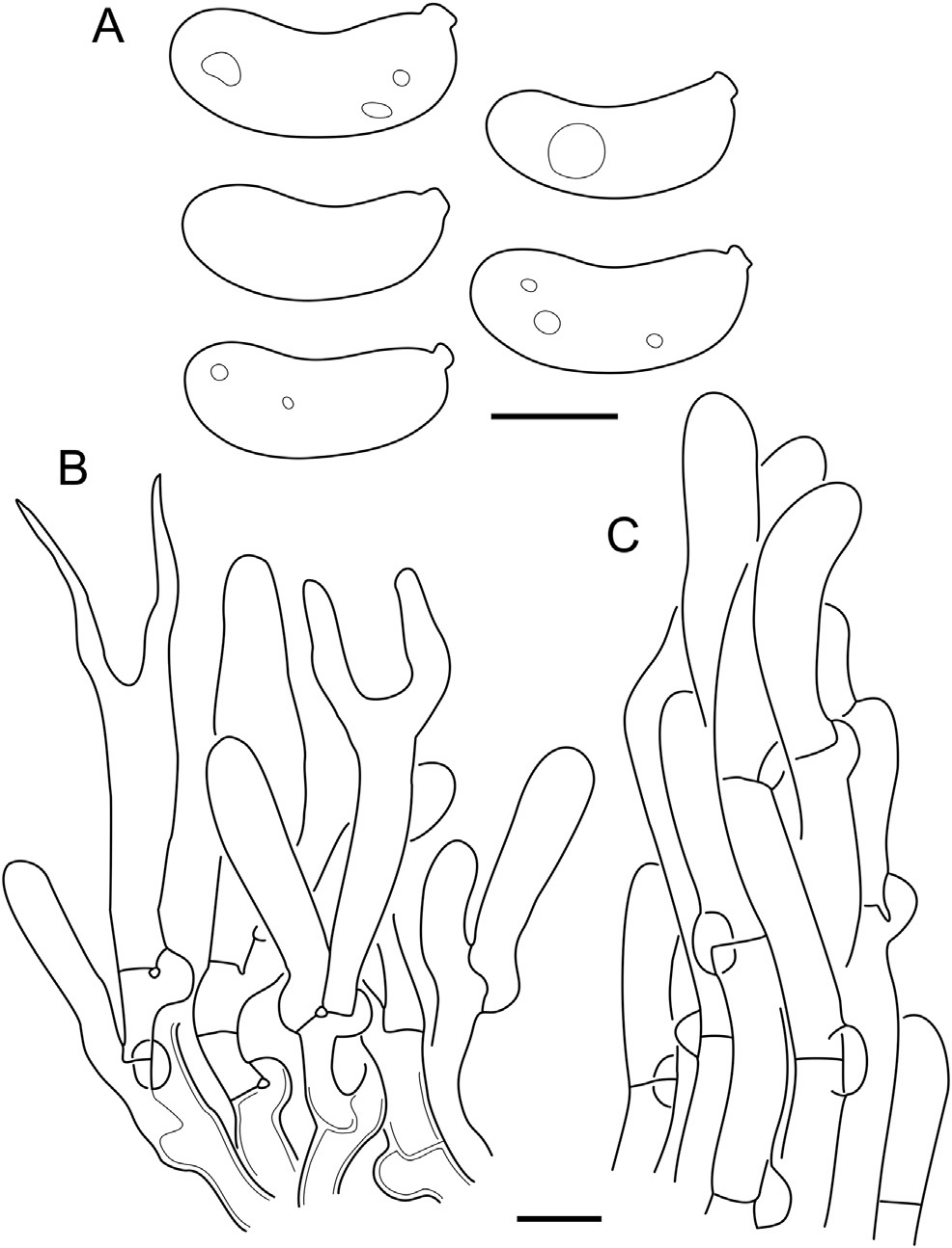
*Cerinomyces brevisetus* micromorphology. **A.** Spores. **B.** Hymenium and subhymenium. **C.** Apical part of a hyphal peg. All drawn from holotype, URM:Chikowski 1544. Scale bars = 5 μm.

***Cerinomyces brevisetus*** Chikowski, Alvarenga & A. Savchenko, ***sp. nov.*** MycoBank MB 839784. Fig. 9, Fig. 22.

*Typus*: **Brazil**, Pernambuco, Igarassu mun., Refugio Ecológico Charles Darwin, 12 May 2017, R. Chikowski 1544 (**holotype** URM∗!, **isotypes** H!, TU135139!).  

*Etymology*: brevi (Lat.) — short; seta (Lat.) — bristle-like organ, peg herein.  

*Description*: *Basidiocarps* arid, at first arachnoid, later mature areas become crustose, covering thin cottony subiculum; hymenial surface solid, light ochraceous, with regular brownish *hyphal pegs* up to 80 μm in height. *Hyphae* clamped, subicular hyphae 2–3 μm in diam, walls 0.5–1.0 μm in width; in subhymenium 2–4 μm in diam, walls ∼ 0.3 μm in width. Pegs rooted in subiculum, their hyphae parallel and densely arranged, core hyphae similar to subicular, outer hyphae 3.5–4.5 μm in diam, walls 0.3–0.4 μm in width. *Hymenium* includes rare simple *hyphidia* with cylindrical or obclavate base and cylindrical long tip; scattered or organized in microscopic pegs. *Basidia* clavate, 12–23 × 3–5.5 μm, with sterigmata up to 19 μm in length (n = 30/1). *Basidiospores* cylindrical, slightly curved, aseptate, 9.3–11.1(–11.7) × 3.5–4.4(–4.6) μm, L = 10.3 μm, W = 3.9 μm, Q = 2.6, Q’ = 2.4–2.9 (n = 30/1), walls ∼ 0.2–0.3 μm in width.  

*Habitat and distribution*: Unidentified wood; South America (known only from the type locality).  

*Notes*: *Cerinomyces brevisetus* has the shortest pegs in the genus, which helps to differentiate it from the closest relatives in the *C. albosporus* group, as well as from the similarly looking *C. pallidus* and *C. paulistanus*. In addition, all other members of the *C. albosporus* clade have longer basidiospores, while the *C. pallidus* clade is characterized by the shorter ones.  

**Fig. 23:**
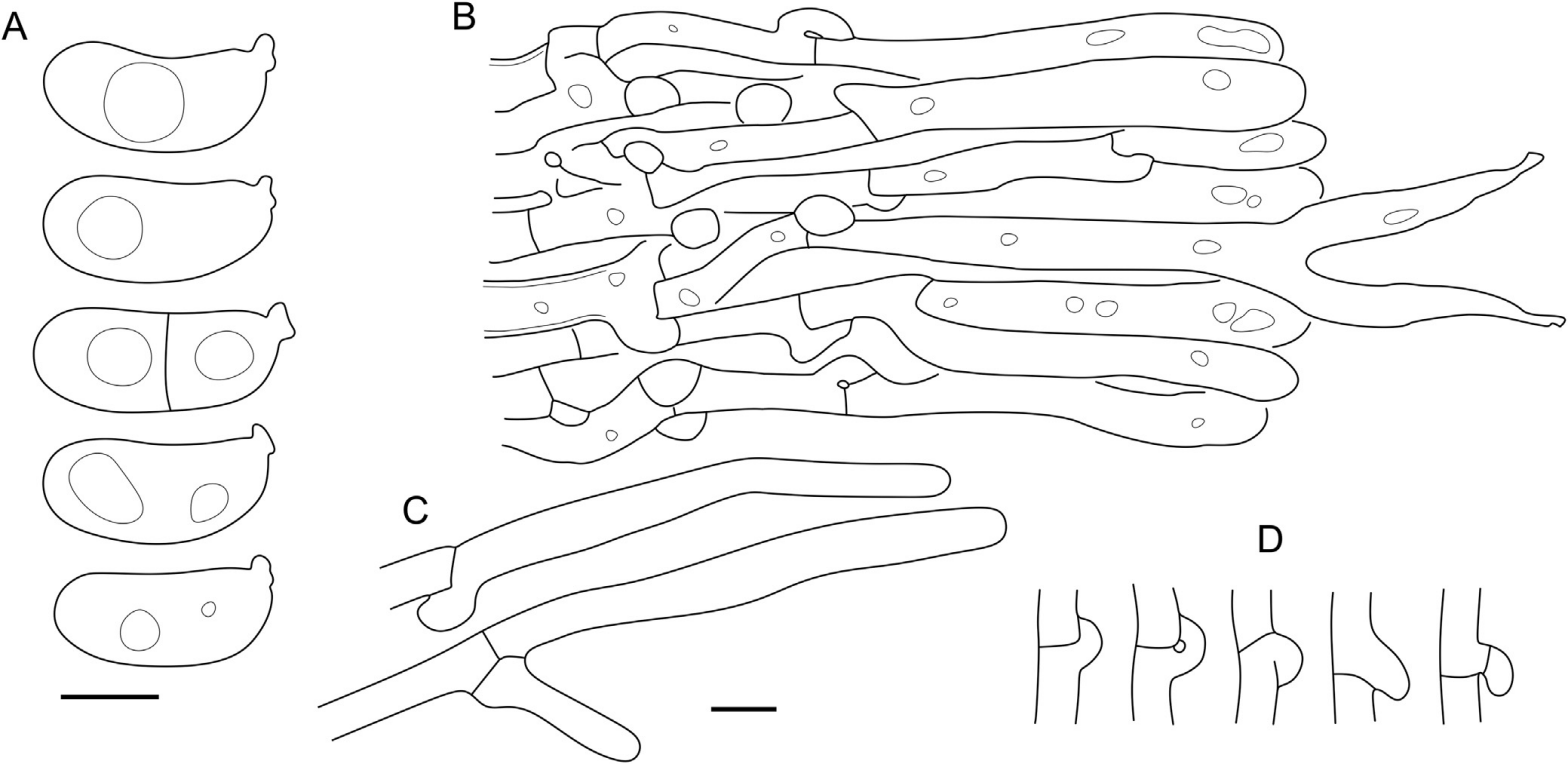
“*Cerinomyces” canadensis* micromorphology. **A.** Spores. **B.** Hymenium and subhymenium. **C.** Marginal hyphae. **D.** Different types of clamps. All drawn from H:Spirin 8468. Scale bars = 5 μm.

***Cerinomyces canadensis*** (H.S. Jacks. & G.W. Martin) G.W. Martin, Mycologia 41: 85 (1949). Fig. 14, Fig. 23.

*Basionym: Ceracea canadensis* H.S. Jacks. & G.W. Martin, Mycologia 32: 693 (1940).  

*Typus*: **Canada**, Ontario, Port Alexander, on gymnosperm wood, 13 Sep. 1939, H.S. Jackson (**holotype** TRTC14103).  

*Description*: *Basidiocarps* corticioid, of variable shapes, cover up to several cm in the longest dimension, with cottony thick white subiculum and fimbriate white margins. In younger areas margins indistinct, arachnoid. Hymenial surface smooth and solid, waxy-gelatinous when fresh, crust-like when dried, from yellow to deep orange colour, in old collections bleaks to pale ochraceous. *Hyphae* clamped, pseudoclamps occasionally present, subicular hyphae 2–4 μm in diam, walls 0.2–0.3 μm in width; in subhymenium 2–3.5 μm in diam, walls of the same width; marginal hyphae cylindrical, similar to subicular. *Hymenium* consists of clavate *basidia* 19–43 × 3–6 μm, with sterigmata up to 31 μm in length (n = 54/3). *Basidiospores* cylindrical to slightly curved-cylindrical, often with conspicuous lipid droplets, 0–1-septate, (8.5–)9.2–14.7(–16.5) × (4.1–)4.3–6.2(–6.8) μm, L = 11.7 μm, W = 5.1 μm, Q = 2.3, Q’ = 1.6–3.2 (n = 126/4; 75 measured in 1 % KOH), walls ∼ 0.2 μm in width.  

*Habitat and distribution*: Gymnosperm wood (*Abies*, *Picea*, *Tsuga*, and unident.); East Asia and North America.  

*Material examined*: **Russia**, Krasnoyarsk reg., Yenisey dist., E of mouth of the river Kolchim, on gymnosperm wood, 14 Aug. 1958, E. Parmasto (TAAM007082∗); Primorsk reg., Chuguyev dist., Lesosetshnaya river, 9 km upstream from Bulyga-Fadejevo / Sandagou village, on *Abies nephrolepis*, 7 Sep. 1961, E. Parmasto (TAAM016177); Sakhalin, Tymovsky dist., Nabili Mountains, in the walley of Pilenga river, on *Picea jezoensis*, 22 Aug. 1970, B. Kullman & A. Raitviir (TAAM061880∗). **USA**, Idaho, Bonner co., Trapper Creek, on *Tsuga heterophylla*, 14 Oct. 2014, V. Spirin 8468 (H∗, UPS).  

*Notes*: The species does not belong to *Cerinomyces s.s.*, but we postpone raising a new synonym (see Discussion). The type material could not be located in TRTC hebarium (S. Margaritescu, 15 Sep. 2016, pers. comm.), but morphology of the studied specimens agrees well with the original concept of *Ceracea canadensis*. It can be confused with *Dacrymyces corticioides*, though the latter species possesses larger microstructures, basidiospores with up to three septa and basidiocarps of well-defined circular form. Maekawa (1987) reported ovoid to straight cylindrical conidia born from hyphae in a monokaryotic strain of *C. canadensis* (CCCM 0194), but we did not check identity of this strain.  

 Under pseudoclamp we understand a clamp which development was arrested before the terminal part of a clamp fused with the parental hypha (upper Fig. 23 C, three rightmost pictures on Fig. 23 D, Clémençon *et al.* 2004). The character is rather rare in the species but was found in all studied specimens. Sequences from TAAM007082 and TAAM061880 were not used in the analyses, but they are similar to the ITS of Spirin 8468.  

**Fig. 24:**
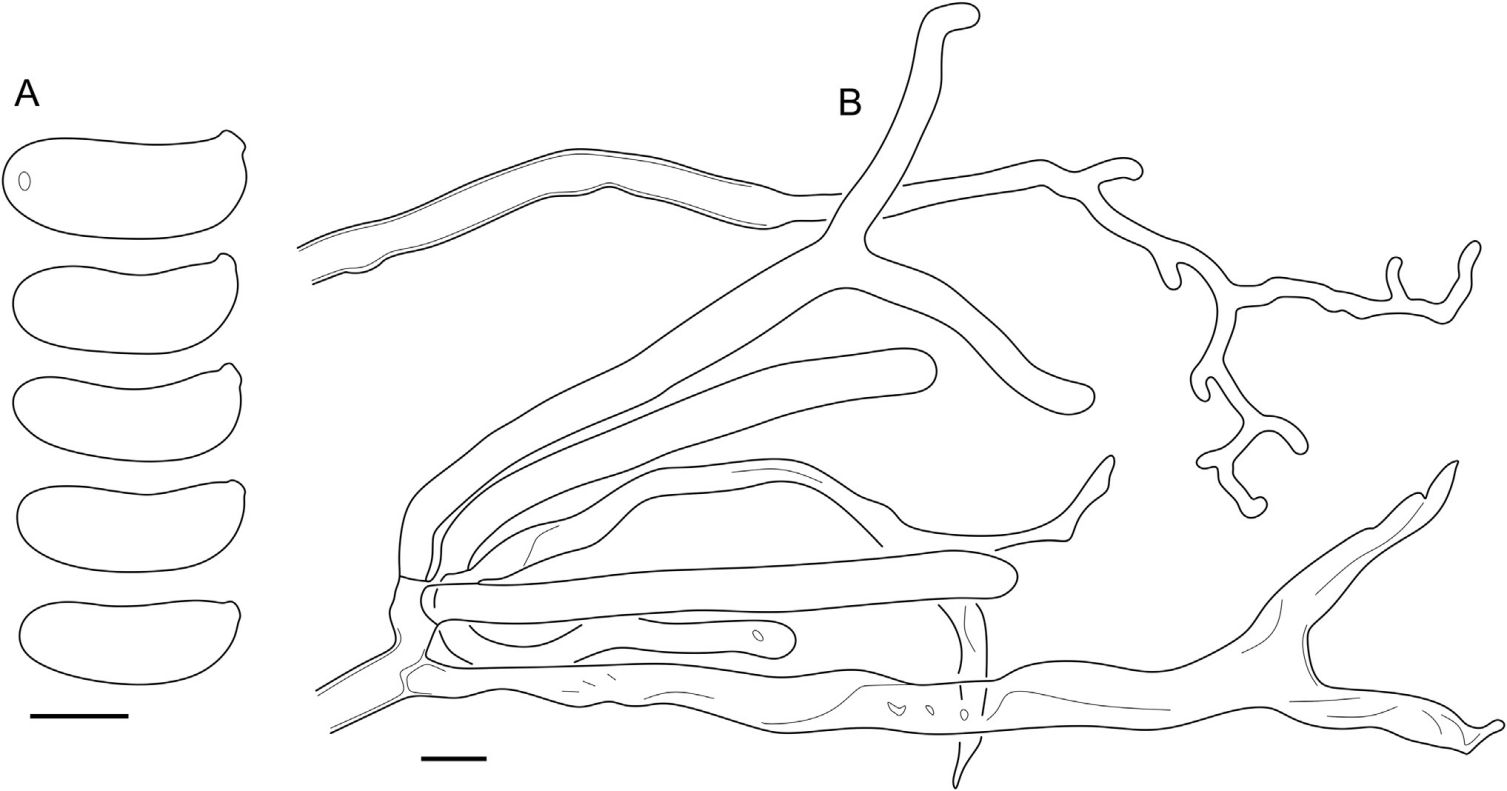
*Cerinomyces cokeri* micromorphology. **A.** Spores. **B.** Hymenium and a single hyphidium. Drawn from holotype, NCU-F-0031543 (A); NCSLG21222 (B). Scale bars = 5 μm.

***Cerinomyces cokeri*** (McNabb) A. Savchenko & J.C. Zamora, ***comb. nov.*** MycoBank MB 839809. Fig. 11, Fig. 24.

*Basionym: Dacrymyces cokeri* McNabb, New Zealand Journal of Botany 11: 475 (1973).

*Synonym: Dacrymyces pallidus* Coker, Journal of the Elisha Mitchell Scientific Society 35: 171 (1920) *nom. illeg.,* not *D. pallidus* Lloyd, 1919.  

*Typus*: **USA**, North Carolina, Orange co., Chapel Hill, on gymnosperm wood, 4 Feb. 1920, J.N. Couch 4072 (**holotype** NCU-F-0031543!).  

*Description*: *Basidiocarps* gelatinous, of irregular shapes, pustulate or cerebriform with thin resupinate margins, up to 15 mm in the longest dimension, pale yellow when fresh, light brown at margins to brown at middle, dark brown when dried, often erumpent through bark. *Hyphae* clamped, in subiculum 1.5–2.5 μm in diam, walls 1.5–3.0 μm in width together with gelatinous layer; small knots of ramificated hyphae rarely occur; subhymenial hyphae of the same diam, walls 0.2–0.3(–0.5) μm in width; margins covered with simple cylindrical or slightly clavate anastomosing hyphae 1.5–2.5 μm in diam, walls 0.2–0.5 μm in width. *Hymenium* includes dendroid cylindrical *hyphidia* with base 2–3.5 μm in diam and apical part 1–2 μm in diam; up to 40 μm in total length. *Basidia* clavate, 36–60 × 3–6 μm. Sterigmata up to 34 μm in length (n = 30/1). *Basidiospores* cylindrical to slightly curved-cylindrical, 0(–1)-septate, at least some binucleate, (9.6–)9.7–12.8(–13.0) × (3.4–)3.5–4.6(–5.0) μm, L = 11.2 μm, W = 4.0 μm, Q = 2.8, Q’ = 2.3–3.2 (n = 55/2), walls ∼ 0.2 μm in width.  

*Habitat and distribution*: Gymnosperm wood (*Pinus* and unident.); North America.  

*Material examined*: **Canada**, Ontario, Ottawa, on *Pinus resinosa*, 27 Jan. 2020, I. Khomenko 2020-034 (UPS:F-979575∗, TU135089). **USA**, North Carolina, Wake co., Raleigh, Lake Johnson Nature Park, on *Pinus taeda*, 28 Oct. 1972, J.A. Menge 396 (NCSLG21222∗).  

*Notes*: *Cerinomyces cokeri* can be confused with *C. enatus*, both occuring on coniferous wood with bark. *Cerinomyces enatus* is generally darker coloured, becoming almost black in dry conditions, while *C. cokeri* remains dark brown. Also, we did not find in *C. cokeri* swollen hyphal compartments and such thick gelatinous layer on hyphae as in *C. enatus*.  

**Fig. 25:**
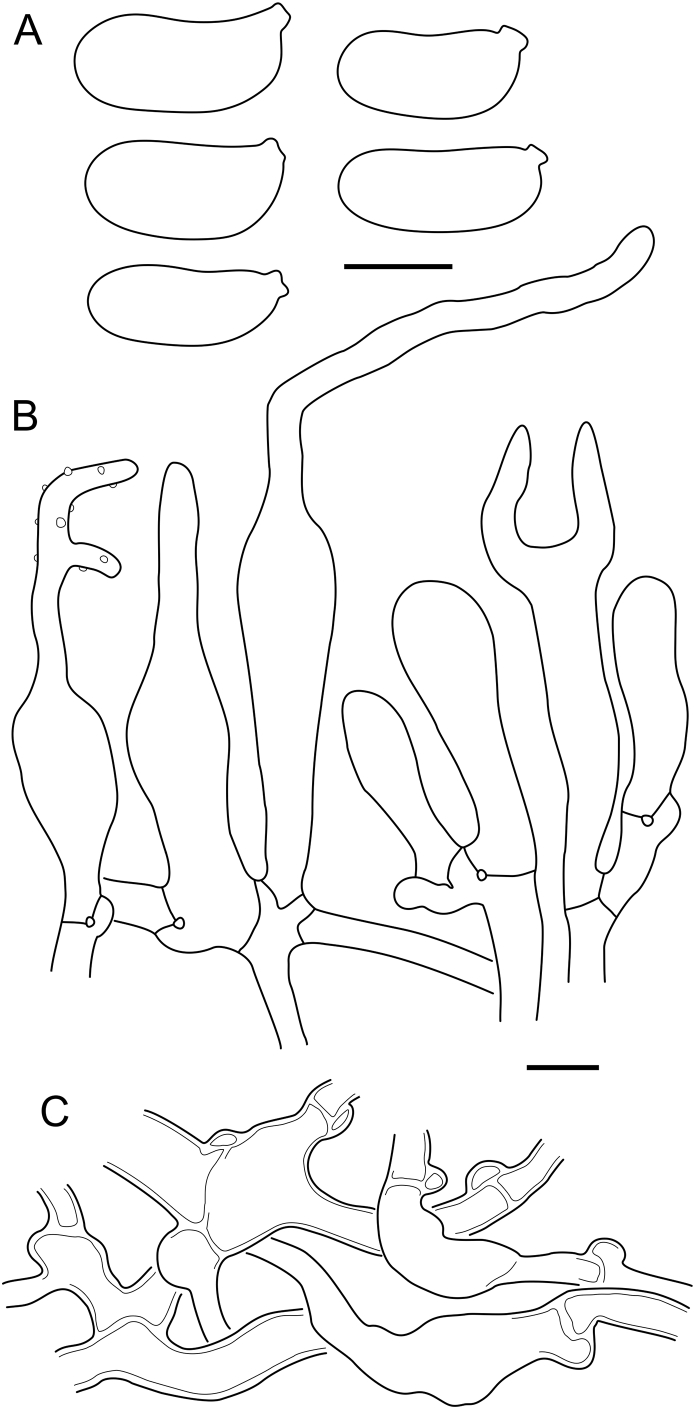
*Cerinomyces concretus* micromorphology. **A.** Spores. **B.** Hymenium with hyphidia. **C.** Subhymenial hyphae and swollen cells. All drawn from holotype, O:F-919450. Scale bars = 5 μm.

***Cerinomyces concretus*** A. Savchenko, ***sp. nov.*** MycoBank MB 839785. Fig. 9, Fig. 25.

*Typus*: **Colombia**, Magdalena, Santa Marta, Park Sierra Nevada de Santa Marta, Reserva Forestal San Lorenzo, 17–19 Jun. 1978, L. Ryvarden (**holotype** O:F-919450∗!).  

*Etymology*: concretus (Lat.) — grown together, condensed. Reference to a dense hymenium becoming crustose.  

*Description*: *Basidiocarps* arid, crustose, slightly gelatinous when rewetted, with thin subiculum and indistinct margins, hymenial surface smooth and solid but cracking in older areas, of pale ochraceous to yellowish brown colour. *Hyphae* clamped, in subiculum loose, 1.5–3(–3.5) μm in diam, walls 0.3–0.4(–0.8) μm in width, covered with small crystals (≤ 1 μm) that can cluster in amorphic masses. Subhymenial hyphae of the similar type, densely packed, with occasional swellings. *Hymenium* includes simple *hyphidia* of total length up to 70 μm, with thickened clavate base 4–5 μm in diam, and apical cylindrical part 2 μm in diam, up to 50 μm in length, occasionally branched or incrusted with minute crystals. *Basidia* clavate, 15–28 × 3–6 μm. Sterigmata up to 11(–19) μm in length, widely spaced (n = 30/1). *Basidiospores* slightly curved-cylindrical, aseptate, (7.7–)7.8–10.5(–12.0) × (3.3–)3.4–4.5(–5.0) μm, L = 9.0 μm, W = 4.0 μm, Q = 2.2, Q’ = 1.9–3.0 (n = 30/1), wall ∼ 0.2–0.3 μm in width.  

*Habitat and distribution*: Unidentified wood; Central America.  

*Material examined*: **Venezuela**, La Silla, boundary between Distrito Federal and Estado Miranda, 22 Aug. 1975, A.E. Liberta & A.J. Navas 22-43, ex ILL1499 (BPI1106573).  

*Notes*: Comparing to other arid *Cerinomyces*, *C. concretus* is characterized by the large basidiospores. Two species with spore W > 3.5 μm, *C. pinguis* and *C. verecundus*, do not overlap with *C. concretus* in distribution. The type specimen has a slight meruloid pattern under hymenium that may be an evidence of an old, overgrown basidiocarp. A lone 1-septate spore was found in the type.  

**Fig. 26:**
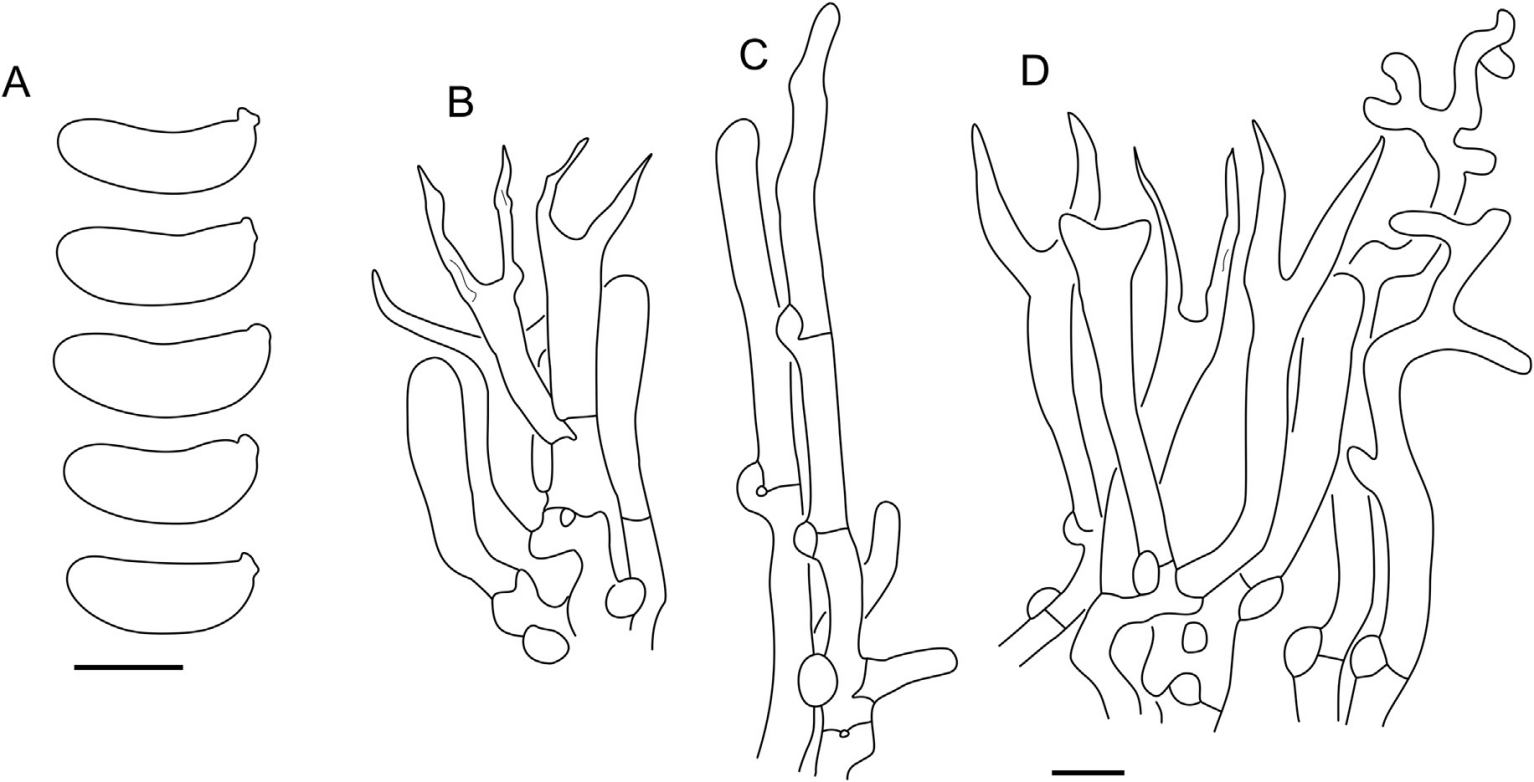
*Cerinomyces creber* micromorphology. **A.** Spores. **B.** Hymenium with short basidia and subhymenium. **C.** Marginal hyphae. **D.** Hymenium with full-sized basidia, hyphidia, and subhymenium. All drawn from H:Trichies 07077. Scale bars = 5 μm.

***Cerinomyces creber*** J.C. Zamora, A. Savchenko, Trichies & Olariaga, ***sp. nov.*** MycoBank MB 839786. Fig. 11, Fig. 26.

*Typus*: **Spain**, Castilla–La Mancha comm., Toledo, pinar de la Bastida, on *Cupressus arizonica*, date unknown, J. De Esteban (**holotype** UPS:F-946512∗!).  

*Etymology*: creber (Lat.) — close, numerous; referring to masses of coalescing basidiocarps.  

*Description*: *Basidiocarps* gelatinous, pustulate, up to 1 mm in diam, readily coalescing with outlines of separate basidiocarps remaining visible. Young basidiocarps pale yellow to pale cream, later light brown to greyish brown, becoming brown and dark brown when dried. Internal or marginal areas can look opalescent in well-developed basidiocarps; margin becomes fimbriate upon drying. *Hyphae* clamped, subicular hyphae 2.5–4.5 μm in diam, walls 0.4–0.8 μm in width; in subhymenium 2–3.5 μm in diam, walls 0.2–0.8 μm in width. *Hymenium* includes dendroid cylindrical *hyphidia* with thickened base 2–2.5 μm in diam and branching apical part 1.5–2 μm in diam; up to 50 μm in total length, from rare to abundant in different areas. *Basidia* clavate, rarely asymmetric, 14–36 × 3–5 μm, with sterigmata up to 18 μm in length (n = 55/2). *Basidiospores* cylindrical, slightly curved, aseptate, binucleate, (7.4–)7.7–11.0(–12.8) × (2.5–)2.8–4.0(–4.2) μm, L = 9.4 μm, W = 3.3 μm, Q = 2.9, Q’ = 2.4–3.6 (n = 61/2), walls ∼ 0.2 μm in width.  

*Habitat and distribution*: Gymnosperm wood (*Cupressus*, *Juniperus*, and unident.); Western and South-Western Europe.  

*Material examined*: **France**, Moselle dept., Neufchef, carreau de l'ancienne mine de fer du Conroy, on gymnosperm plywood, 7 Mar. 2007, G. Trichies 07077 (H∗). **Spain**, Castilla–La Mancha comm., Guadalajara prov., Tamajón, near ermita de la Virgen de los Enebrales, on *Juniperus thurifera*, 28 Dec. 2019, J.C. Zamora & al. (UPS:F-979574∗); Madrid comm., Hoyo de Manzanares, collado del Portacho, on *J. oxycedrus*, 4 Jan. 2018, I. Olariaga (UPS:F-946506∗), Hoyo de Manzanares, cerro Camorrila, on *J. oxycedrus*, 8 March 2018, I. Olariaga (UPS:F-946507∗).  

*Notes*: *Cerinomyces creber* is similar to *C. neuhoffii* in the branched hyphidia and tendency to develop dense coalescing basidiocarp groups, but the former species has smaller basidiospores, slightly smaller basidia, and often more light-coloured basidiocarps that coalesce more readily. Also, it seems that *C. creber* is confined to *Cupressaceae* wood (according to the substrates we were able to identify), while all studied specimens of *C. neuhoffii* were found on *Pinus* wood. *Cerinomyces aff. crustulinus 1* resembles *C. creber* by macro- and micromorphology but represents a distinct taxon with even paler basidiocarps growing on angiosperm wood. Sequence of UPS:F-979574 was produced during the revision of this study; it was not used in the analyses but matches other included sequences.  

**Fig. 27:**
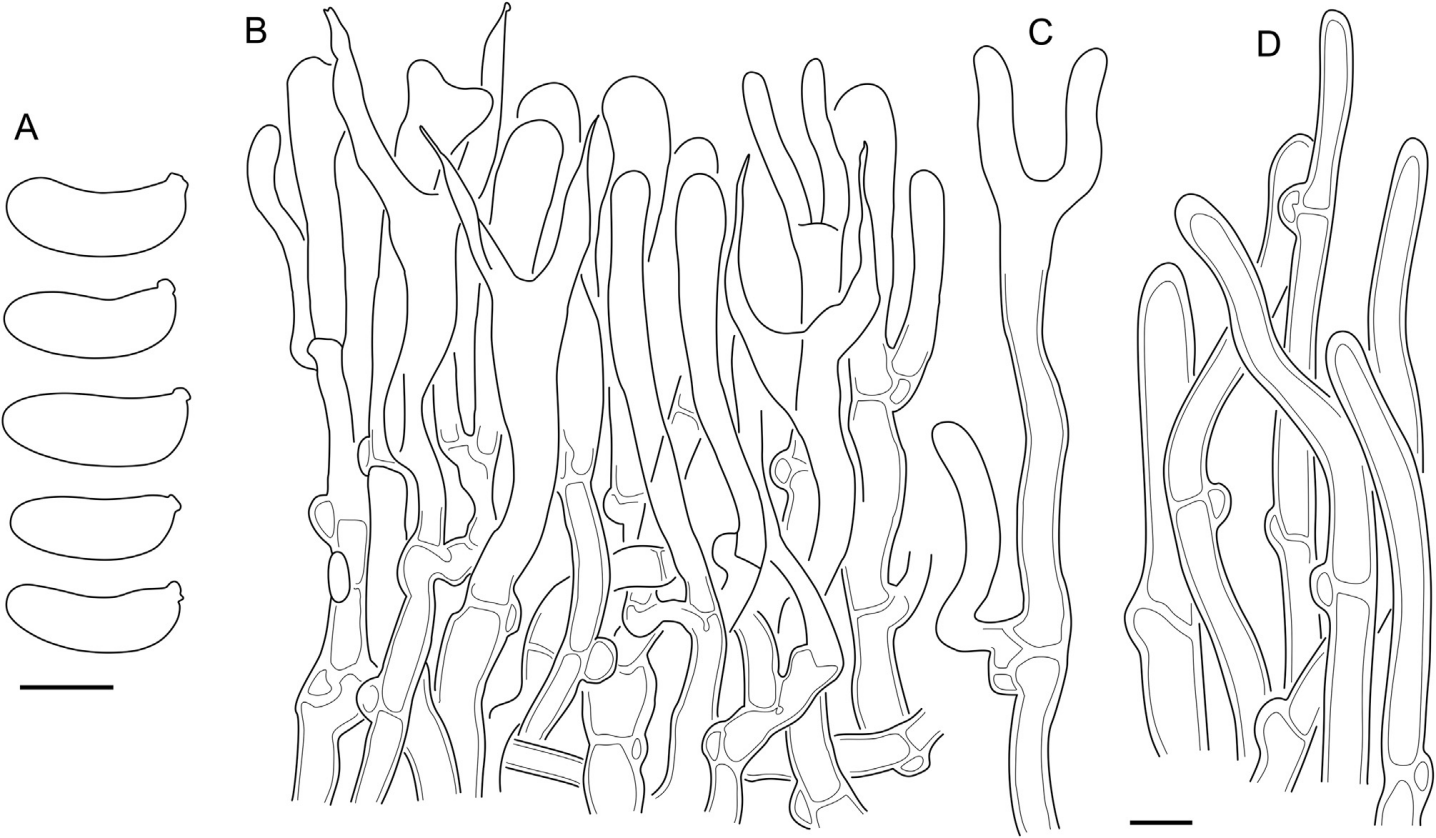
*Cerinomyces crustulinus* micromorphology. **A.** Spores. **B.** Hymenium and subhymenium. **C.** Single basidium. **D.** Marginal hyphae. All drawn from isotype, BPI726061. Scale bars = 5 μm.

***Cerinomyces crustulinus*** (Bourdot & Galzin) G.W. Martin, Mycologia 41: 85 (1949). Fig. 11, Fig. 27.

*Basionym: Ceracea crustulina* Bourdot & Galzin, Bulletin de la Société Mycologique de France 39: 266 (1923).  

*Typus*: **France**, Gard dept., Saint-Guiral, on *Fagus*, 6 May 1910, A. Galzin 5793, H. Bourdot herb. (?) n. 7352 (**lectotype** PC0706688!, **isolectotype** BPI726061!).  

*Description*: *Basidiocarps* gelatinous or waxy-gelatinous, originate as small gregarious pustules, coalesce into thin resupinate conglomerate with outlines of separate basidiocarps remaining visible, ochraceous to dull orange. Margin and subiculum become fimbriate yellowish white when dry. *Hyphae* clamped; subicular hyphae 2.5–4 μm in diam, walls 0.5–1.0 μm in width; subhymenial hyphae 2–3(–5) μm with walls 0.6–0.8(–1.0) μm in width; marginal hyphae resembling subicular, with cylindrical or slightly clavate endings. *Hymenium* simple, occasional *hyphidia* cylindrical. *Basidia* clavate, 16–38 × 2.5–5 μm with two sterigmata up to 28 μm in length (n = 60/2). *Basidiospores* cylindrical, slightly curved, aseptate, (7.4–)7.7–13.4(–14.0) × 2.8–4.0 μm, L = 9.8 μm, W = 3.3 μm, Q = 3.0, Q’ = 2.1–3.6 (n = 58/2), walls ∼ 0.2 μm in width.  

*Habitat and distribution*: Angiosperm wood (*Fagus*); Europe (known only from the type locality).  

*Notes*: No dendroid hyphidia were observed in contrast to an earlier report (McNabb 1964). We assume hyphidia were unevenly distributed in basidiocarps and destroyed with past preparations. Among the species described in this revision, the closest morphological relative to *C. crustulinus* is resupinate *C. aeneus* that also inhabits angiosperm wood in Europe, but differs in darker cerebriform-resupinate basidiocarps with reddish tint and abundant branched hyphidia. The extremely scanty type material and absence of fresh collections that fully agree with our observations prevent resolution of the exact position of *C. crustulinus* (but see *C. aff. crustulinus 1* below). Roberts (2006) reported the species from Jamaica, and while it does resemble *C. crustulinus*, the robust gelatinous discoid to coalescing brown basidiocarps with light margins, abundant branched hyphidia, larger spores (11.4–)11.8–13.8(–14.5) × 4.0–5.2(–5.7) μm place it closer to either *C. enatus* or even members of the *Dacrymycetaceae*.  

**Fig. 28:**
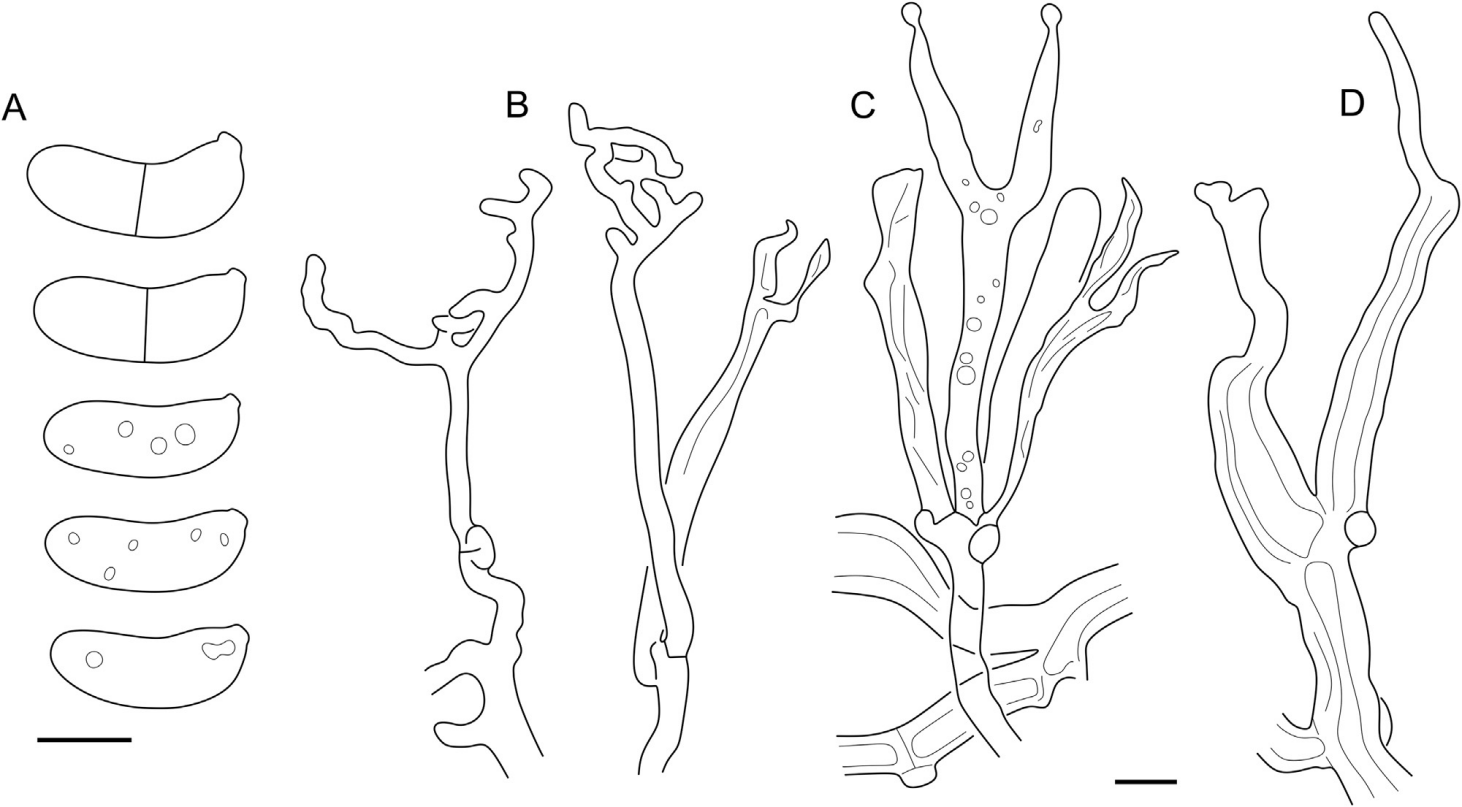
*Cerinomyces aff. crustulinus 1* micromorphology. **A.** Spores. **B.** Hyphidia. **C.** Hymenium and subhymenium. **D.** Marginal hyphae. All drawn from UPS:F-958851. Scale bars = 5 μm.

***Cerinomyces aff. crustulinus 1***. Fig. 11, Fig. 28.

*Description*: *Basidiocarps* gelatinous, pustulate and gregarious, < 1 mm in diam, readily coalescing into resupinate conglomerate with shapes of separate basidiocarps remaining visible, from yellowish white to light yellow when fresh, light brown when dry. Margin slightly opalescent when fresh and fimbriate when dry. *Hyphae* clamped; subicular and subhymenial hyphae similar, 2–3.5 μm in diam, walls 0.2–0.3(–0.5) μm in width with gelatinous layer up to 1.5 μm in width; marginal hyphae slightly clavate, sometimes with thinner rarely branching apical part. *Hymenium* includes abundant dendroid cylindrical *hyphidia* with base either thickened to 2–4 μm in diam or of the same width as apical branching part of 1.5–2 μm in diam; up to 60 μm in total length. *Basidia* clavate, 20–49 × 3–5 μm, walls often thickened towards the base, with sterigmata up to 23 μm in length (n = 40/1). *Basidiospores* cylindrical, slightly curved, 0(–1)-septate, at least some apparently binucleate, (8.4–)8.6–10.9(–11.6) × (3.4–)3.4–4.2(–4.2) μm, L = 9.8 μm, W = 3.7 μm, Q = 2.7, Q’ = 2.3–3.0 (n = 45/1), walls ∼ 0.2 μm in width.  

*Habitat and distribution*: Angiosperm wood; Europe.  

*Material examined*: **Spain**, Asturias comm., Somiedo, Coto de la Buena Madre, on angiosperm wood, 2 Jun. 2018, E. Rubio 7557 (UPS:F-958851∗).  

*Notes*: The taxon is the closest match to *C. crustulinus* except for difference in branched hyphidia (but see commentaries on *C. crustulinus* above about previous report of hyphidia in this species). In absence of additional material, we are inclined to keep this specimen and the type material of *C. crustulinus* as two separate taxa.  

**Fig. 29:**
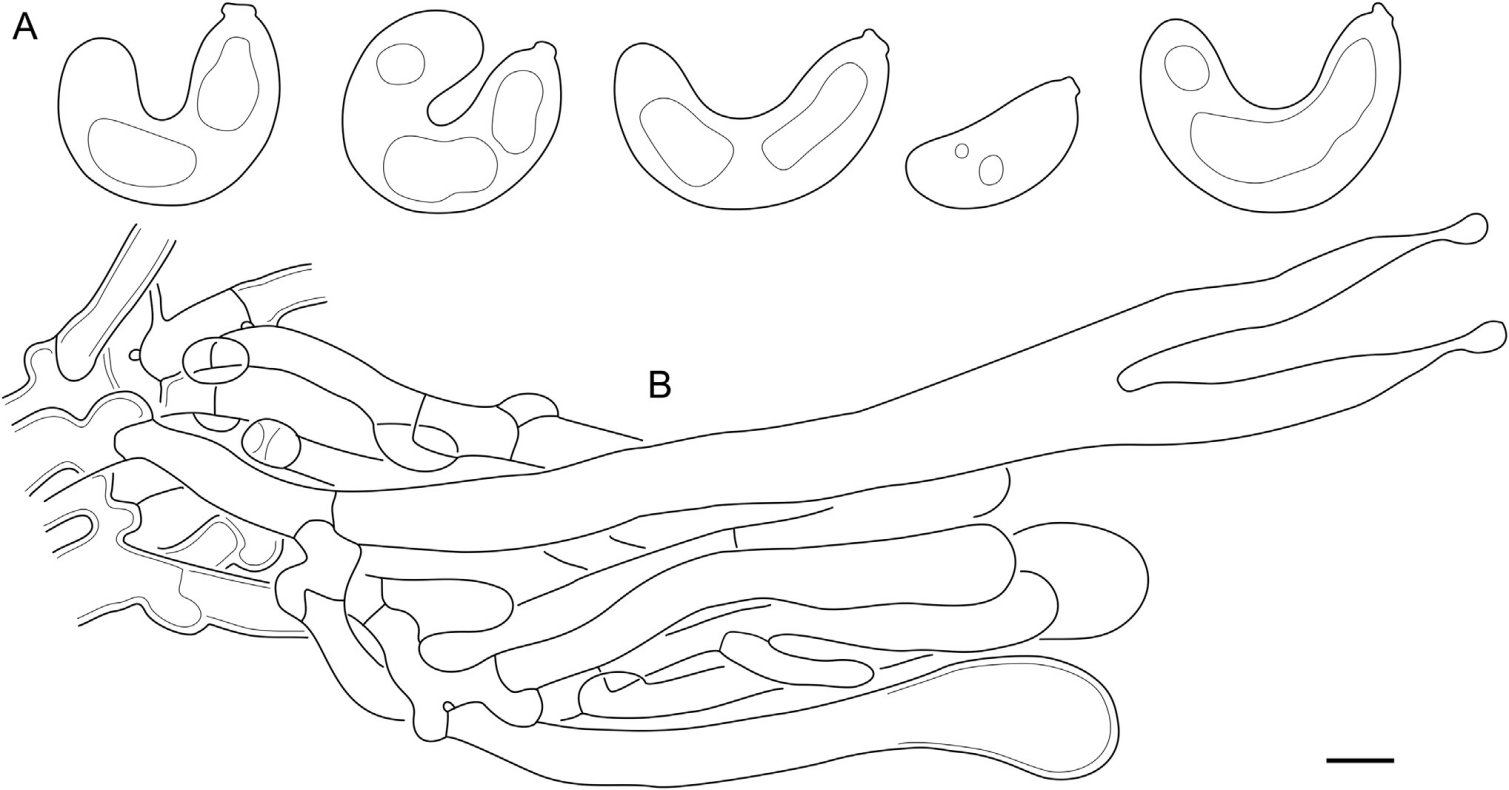
*Cerinomyces curvisporus* micromorphology. **A.** Spores. **B.** Hymenium and subhymenium. All drawn from isotype, TNS-F-11473. Scale bar = 5 μm.

***Cerinomyces curvisporus*** N. Maek. & M. Zang, Mycotaxon 61: 344 (1997). Fig. 9, Fig. 29.

*Typus*: **China**, Yunnan, Lijiang co., Ganhaizi, at the foot of Yulong mountain, on *Pinus densata*, 16 Sep. 1993, N. Maekawa 93091618 (**holotype** HKAS27392!, **isotypes** TMI-18048, TNS-F-11473!).  

*Description*: *Basidiocarps* arid, arachnoid on margins and solid in well developed areas, subiculum thin, hymenial surface light ochraceous, covered with regular *hyphal pegs* up to 300 μm in height. *Hyphae* clamped, in subiculum 2–3(–5) μm in diam, walls 0.5–1.0 μm in width, with minute crystals; in subhymenium 2.5–5 μm in diam, walls 0.3–0.5 μm in width. Internal hyphae in hyphal pegs parallel, similar to subicular, 3.5–5 μm in diam, walls 0.5–1.0 μm in width; at the top 3–5 μm in diam, walls 0.3–0.5 μm in width. *Hymenium* consists of clavate *basidia* 41–77 × 6–10 μm, with sterigmata up to 34 μm in length (n = 30/1), some probasidia have thickened walls in upper parts. *Basidiospores* strongly curved-cylindrical, 0(–3)-septate, (16–)16.3–20.0(–20.4) × 5.9–7.1(–7.5) μm, L = 18.4 μm, W = 6.5 μm (n = 30/1), spore walls ∼ 0.2–0.3 μm in width.  

*Habitat and distribution*: Gymnosperm wood (*Pinus*); Southeast Asia (known only from the type locality).  

*Notes*: The species is easy to recognize by large, strikingly curved basidiospores. Sequencing was not attempted because the basidiocarp was difficult to separate from another, overgrown corticioid fungus. Judging from abundance of hyphal pegs, *C. curvisporus* belongs to the *C. albosporus* group. Septate basidiospores are extremely rare, 1-septate ones were reported in the original description (Maekawa & Zang 1997), and in addition we found a single collapsed 3-septate spore. The longest spore dimension was measured as length. Q values are not shown, being not comparable to other *Cerinomyces* species with straighter basidiospores.  

**Fig. 30:**
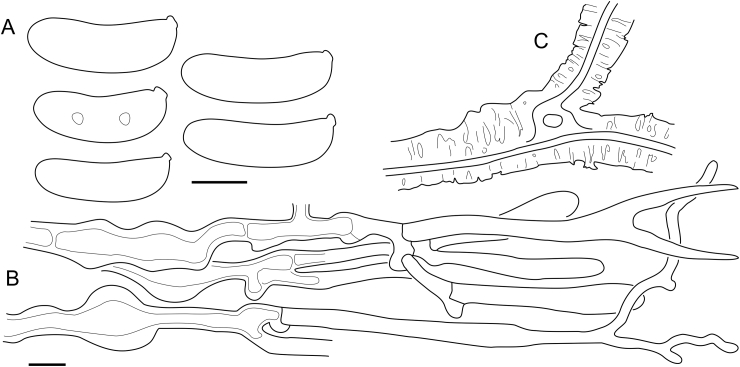
*Cerinomyces enatus* micromorphology. **A.** Spores. **B**. Subicular hyphae with roughened gelatinous layer. **C.** Hymenium with hyphidium, and gelatinized subhymenium with swellings. All drawn from H:Spirin 7774. Scale bars = 5 μm.

***Cerinomyces enatus*** (Berk. & M.A. Curtis) A. Savchenko, ***comb. nov.*** MycoBank MB 839812. Fig. 12, Fig. 30.

*Basionym: Tremella enata* Berk. & M.A. Curtis, Grevillea 2 (14): 20 (1873).

*Synonyms: Dacrymyces enatus* (Berk. & M.A. Curtis) Massee, Journal of Mycology 6 (4): 182 (1891).

*Dacrymyces fuscominus* Coker, Journal of the Elisha Mitchell Scientific Society 35: 171 (1920).

*Arrhytidia enata* (Berk. & M.A. Curtis) Coker, Journal of the Elisha Mitchell Scientific Society 43: 237 (1928).

*Dacrymyces gangliformis* Brasf., Lloydia 3: 105 (1940).  

*Typus*: **USA**, South Carolina, Darlington co., Society Hill, on *Quercus*, Jan. 1850, H.W. Ravenell 2456 (**lectotype** [designated here]: FH00596873!, **isolectotypes** K(M):94215!, K(M):141181!). MycoBank typification MBT10001414.  

*Description*: *Basidiocarps* gelatinous, variable in shape, pulvinate when young, in maturity most often pustulate, but also slightly cerebriform or flat and resupinate, up to 3(–4) mm in diam, from reddish brown when fresh to dark brown and black when dried; margins of resupinate basidiocarps light yellow and semitransparent. Basidiocarps often grow through holes in bark; when growing on decorticated wood can develop short stalk or, inversely, become resupinate. Light subicular core visible through darker hymenium in well-developed basidiocarps. *Hyphae* clamped, in subiculum 2–5 μm in diam, walls 0.5–1.0 μm in width, with gelatinous layer up to 6 μm in width, agglutinated, often deeply roughened. Swollen cells abundant in upper subiculum of well-developed basidiocarps, up to 20 μm in diam, with thickened roughened walls. Subhymenial hyphae 2–3.5 μm in diam, walls 0.3–0.5 μm in width. Sterile surfaces and margins covered with simple cylindrical or attenuating hyphae. *Hymenium* includes abundant branched cylindrical *hyphidia* with base ∼3 μm in diam and apical part 1–2 μm in diam; up to 30 μm in total length. Occasional simple, not branched hyphidia up to 50(–100) μm in length present, single or in peg-like groups. *Basidia* clavate, 13–56 × 3–6.5 μm, with walls 0.3–0.5 μm in width. Sterigmata up to 27 μm in length (n = 208/9). *Basidiospores* cylindrical to slightly curved-cylindrical, 0(–1)-septate, (7.0–)7.4–13.2(–14.2) × (2.7–) 2.9–4.5(–5.4) μm, L = 9.5 μm, W = 3.5 μm, Q = 2.7, Q’ = 1.7–3.8 (n = 196/9), walls ∼ 0.2 μm in width. Basidiospores bear ellipsoid to cylindrical conidia 3.0–4.0 × 1.5–1.7 μm.  

*Habitat and distribution*: Angiosperm (*Alnus*, *Castanopsis*, *Clethra*, *Quercus*, *Rhododendron*, and unident.) and gymnosperm (*Pinus*) wood; East Asia, North America.  

*Material examined*: **Japan**, Honshu, Chūbu reg., Nagano pref., Shioda, on *Pinus densiflora*, 27 Sep. 2006, T. Shirouzu (same coll. for all Japanese collections) HNo.505 (TNS-F-21064∗), Sugadairakougen, on *P. densiflora*, 7 Nov. 2016, HNo.1169 (TNS-F-88754∗), same loc. and substrate, 3 Sep. 2018, HNo.1212 (TNS-F-88777∗); Kansai reg., Kyoto pref., Mt. Daimonji, on *P. densiflora*, 20 Apr. 2006, HNo.199 (TNS-F-21034∗), Takaragaike, on *Clethra barbinervis*, 21 Apr. 2006, HNo.208 (TNS-F-21035∗), Midorogaike, on *Rhododendron macrosepalum*, 21 Apr. 2006, HNo.216 (TNS-F-21036∗), Mt. Kiyomizu, on *Castanopsis cuspidata*, 22 Apr. 2006, HNo.219 (TNS-F-21037∗); Kantō reg., Ibaraki pref., on *P. densiflora*, 14 Oct. 2014, HNo.1113 (TNS-F-61320∗). **Russia**, Khabarovsk reg., Solnechnyi dist., Igdomi, on *Alnus hirsuta*, 2 Sep. 2016, V. Spirin 10764 (H∗), Verkhnebureinsky dist., Dublikan Nat. Res., on *Alnus alnobetula ssp. fruticosa*, 21 Aug. 2014, V. Spirin 7774 (H∗), 7780 (H∗). **USA**, Maryland, Prince Georges co., Greenbelt, Greenbelt park, Campgrounds, on *Pinus*, 17 Jun. 1968, H.H. Burdsall (CFMR:HHB-671∗); Massachusetts, Norfolk co., Canton, on *P. strobus*, 7 Nov. 1932, D.H. Linder, det. id.: Whelden 142 (**holotype** of *Dacrymyces gangliformis*, FH00304774); North Carolina, Chapel Hill, back of Athletic Field, on oak bark, 4 Feb. 1920, Couch 4075 (**holotype** of *Dacrymyces fuscominus*, NCU-F-0009150); South Carolina, on *Alnus*, date unknown, M.A. Curtis (UPS:F-116976), same loc., on unidentified wood, (UPS:F-116977); Wisconsin, Dane co., Madison, University of Wisconsin Arboretum, Leopold Pines, on *P. resinosa*, 16 Jul. 1973, H.H. Burdsall (CFMR:HHB-7334∗).  

*Notes*: *Cerinomyces enatus* is similar to *C. cokeri* but demonstrates darker colouration, swollen cells and thicker gelatinous layer on internal hyphae. In all studied specimens septate basidiospores are extremely rare. The species shows high variability in morphological characters, nrDNA sequences and substrate preferences. In all publications of Shirouzu *et al.* Japanese materials were cited as *C. canadensis* or *C. pallidus*. For American specimens CFMR:HHB-671 and CFMR:HHB-7334 we were able to sequence only 5.8S and part of ITS2. The sequences were too short to use in the phylogeny reconstruction but sufficient to identify them as *C. enatus*. Interestingly, Seifert (1983) studied a culture UBC 6124 from CFMR:HHB-671 (*D. punctiformis* in his paper), and found in a wood block decay test that it was almost unable to degrade *Pinus* wood — a substrate, from which it was originally collected.  

 The studied types of *C. enatus* from K and FH are contaminated by mycoparasites, contain only few, collapsed basidiospores, and show a highly agglutinated hymenial layer, which altogether deny proper measurements. Nevertheless, characteristic macromorphology, presence of branched hyphidia, thick-walled subicular hyphae with occasional swollen compartments and simple marginal hyphae help to connect type material to the other collections cited here.  

 *Dacrymyces gangliformis* has 0–1-septate basidiospores that are slightly wider than typically in *C. enatus* — 9.2–12 × 4.5–6 μm. The species-defining “ganglia” were found to be altered swollen basidiospores, germinating with hyphae. The type specimen is scanty, poorly preserved and contaminated with anamorphic fungi. Considering similarities in microstructures and basidiocarp, we believe the specimen belongs to *C. enatus*. *Dacrymyces fuscominus* type is also in a rather bad condition, but observed morphology allows us to treat the species as a synonym of *C. enatus*, which has already been suggested by Kennedy (1958b).  

**Fig. 31:**
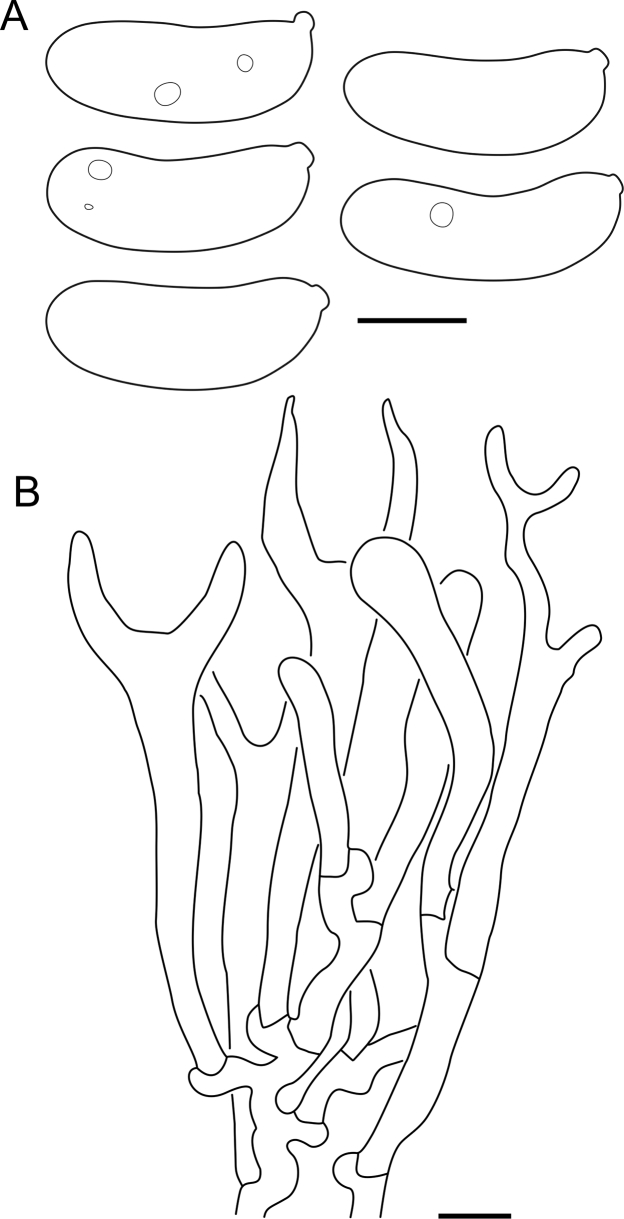
*Cerinomyces enterolaxus* micromorphology. **A.** Spores. **B.** Hymenium with hyphidium, and subhymenium. Drawn from holotype, TNS-F-61292 (A); TNS-F-61319 (B). Scale bars = 5 μm.

***Cerinomyces enterolaxus*** Shirouzu & A. Savchenko, ***sp. nov.*** MycoBank MB 839790. Fig. 12, Fig. 31.

*Typus*: **Japan**, Honshu, Kantō reg., Ibaraki pref., Sakuragawa, Mt. Tsukuba, on *Pinus densiflora*, 15 May 2013, T. Shirouzu HNo.1082 (**holotype** TNS-F-61292∗!).  

*Etymology*: έντερα (Gr.) — intestines; laxus (Lat.) — loose; in reference to wide and loosely arranged subicular hyphae.  

*Description*: *Basidiocarps* gelatinous, pustulate to cerebriform, up to 1(–2) mm in diam, can coalesce into slightly cerebriform film, from almost transparent and yellow to dark brown with reddish tint. *Hyphae* clamped, in subiculum embedded in gelatinous hyaline matrix, loosely arranged, 2–4 μm in diam, walls 0.3–0.5 μm in width. Subhymenial hyphae densely organized, 1.5–3 μm in diam, walls 0.3(–0.5) μm in width. In sterile areas terminal hyphae branched or simple, cylindrical or clavate, 2–4 μm in diam. *Hymenium* includes abundant branched cylindrical *hyphidia* 1.5–2 μm in diam. *Basidia* clavate, 14–33 × 3–6 μm. Sterigmata up to 19 μm in length (n = 62/8). *Basidiospores* cylindrical to slightly curved-cylindrical, occasionally slightly obpyriform, 0(–1)-septate, 9.1–13.7(–14.1) × (3.0–)3.2–4.5(–4.6) μm, L = 11.6 μm, W = 3.9 μm, Q = 3.0, Q’ = 2.4–3.8 (n = 92/9), walls ∼ 0.2 μm in width. Basidiospores produce cylindrical conidia 2.5–3.0 × 1.0–1.2 μm.  

*Habitat and distribution*: Gymnosperm (*Pinus*) and rarely angiosperm (*Clethra*) wood; East Asia.  

*Material examined*: **Japan**, Honshu, Chūbu reg., Nagano pref., Shioda, Ueda-shi, on *Pinus densiflora*, 20 May. 2006, T. Shirouzu (same coll. for all collections) HNo.285 (TNS-F-15725∗), Sugadairakougen, on *P. densiflora*, 11 Jul. 2016, HNo.1135 (TNS-F-88723∗), same loc. and substr., 11 Jul. 2016, HNo.1138 (TNS-F-88726∗), same loc. and substr., 11 Jul. 2016, HNo.1140 (TNS-F-88728∗), same loc. and substr., 11 Jul. 2016, HNo.1148 (TNS-F-88734∗), same loc. and substr., 1 Sep. 2016, HNo.1156 (TNS-F-88742∗), same loc. and substr., 1 Sep. 2016, HNo.1159 (TNS-F-88745∗), same loc. and substr., 7 Nov. 2016, HNo.1168 (TNS-F-88753∗), same loc. and substr., 12 Jul. 2017, HNo.1181 (TNS-F-88762∗), same loc. and substr., 12 Jul. 2017, HNo.1182 (TNS-F-88763∗), same loc. and substr., 12 Jul. 2017, HNo.1187 (TNS-F-88767∗), same loc. and substr., 12 Jul. 2017, HNo.1188 (TNS-F-88768∗), same loc. and substr., 3 Sep. 2018, HNo.1216 (TNS-F-88781∗); Kansai reg., Kyoto pref., Mt. Daimonji, on *P. densiflora*, 20 Apr. 2006, HNo.196 (TNS-F-15723∗), Takaraga-ike, on *Clethra barbinervis*, 21 Apr. 2006, HNo.213 (TNS-F-15724∗); Kantō reg., Ibaraki pref., Sakuragawa, Mt. Tsukuba, on *P. densiflora*, 14 Jun. 2013, HNo.1086 (TNS-F-61296∗), 15 Jul. 2013, HNo.1097 (TNS-F-61306∗), 14 Oct. 2013, HNo.1109 (TNS-F-61316∗), HNo.1110 (TNS-F-61317∗), HNo.1112 (TNS-F-61319∗), 12 Dec. 2013, HNo.1117 (TNS-F-61324∗), 14 Mar. 2014, HNo.1120 (TNS-F-61327∗), HNo.1128 (TNS-F-61334∗), HNo.1129 (TNS-F-61335∗).  

*Notes*: *Cerinomyces enterolaxus* is distinct from most of European gelatinous species by septate basidiospores, thin-walled internal hyphae without heavy gelatinous layer and generally lighter basidiocarp colour. A single collapsed and deformed 3-septate spore was noted. Specimens TNS-F-61327 and TNS-F-61334 are infected by an intrahymenial *Tremella* species. Compared to other *C. enterolaxus* sequences, LSU of TNS-F-88726 contains few short indels and a 20 bp-long duplicating insertion that were cut from the final alignment. In the earlier publications of Shirouzu *et al.* the species was cited as *Dacrymyces punctiformis*.  

***Cerinomyces fasciculatus*** Gilb. & Hemmes, Memoirs of the New York Botanical Garden 89: 81 (2004).  

*Typus*: **USA**, Hawaii, Island of Hawaiʻi, Kaʻu dist., Hawaiʻi Volcanoes National Park, Kīpuka Puaulu, on *Pipturus albidus*, 17 Nov. 1998, R.L. Gilbertson 22101 (**holotype** BPI, **isotype** ARIZ).  

Description *fide* Gilbertson & Hemmes (2004): *Basidiocarps* annual, resupinate, arid, membranous, effused to 10 cm; hymenial surface light buff, smooth, with projecting sterile *hyphal pegs*, margin thinning out, whitish with radiating fibrils at the edge; pegs up to 40 μm diam and projecting up to 70 μm, composed of thin-walled clamped hyphae, arising in the subiculum and appearing as more compactly interwoven columns in the surrounding subicular tissue, apices with projecting rounded hyphal ends. *Hyphae* in subiculum hyaline, with abundant clamps, 2–4.5 μm diam. *Hymenium*. Some irregularly lobed or branched hymenial *hyphidia* present; *basidia* clavate at first, 25–40 × 4–6 μm, becoming bifurcate, sterigmata to 35 μm long and 3–4 μm diam, tapering to pointed apex. *Basidiospores* curved-cylindrical, aseptate, hyaline, smooth, thin-walled, negative in Melzer's reagent, 10–12.5(–13) × 4.5–5.5 μm.  

*Habitat and distribution*: Angiosperm shrubs (*Pipturus*); known only from Hawaiʻi.  

*Notes*: We have not seen the specimens, but according to the described morphology, *C. fasciculatus* is related to the *C. albosporus* clade. The types could not be traced in BPI (several loan requests) or ARIZ (A.E. Arnold, 7 Jul. 2019, pers. comm.).  

**Fig. 32:**
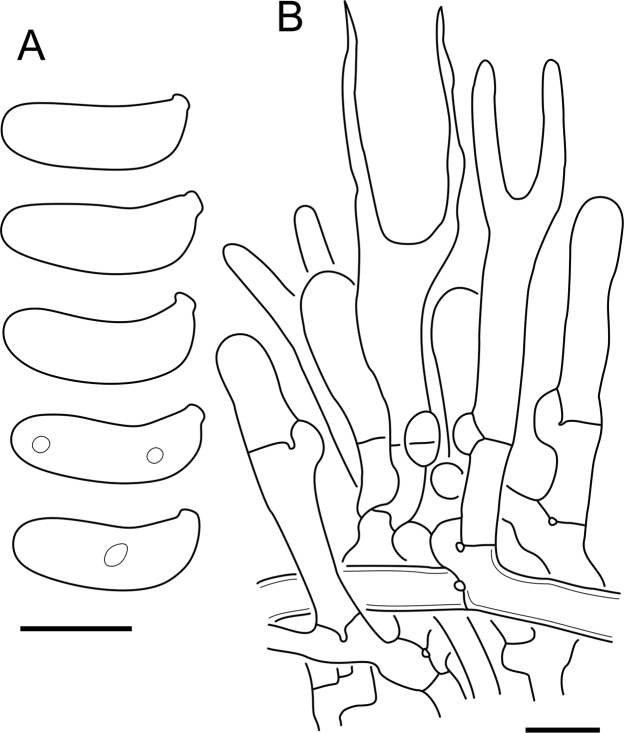
*Cerinomyces favonius* micromorphology. **A.** Spores. **B.** Hymenium and subhymenium. All drawn from holotype, H7008893. Scale bars = 5 μm.

***Cerinomyces favonius*** Spirin, Miettinen & A. Savchenko, ***sp. nov.*** MycoBank MB 839791. Fig. 9, Fig. 32.

*Typus*: **USA**, Idaho, Bonner co., Trapper Creek, on *Tsuga heterophylla*, 14 Oct. 2014, V. Spirin 8473 (**holotype** H7008893∗!).  

*Etymology*: Favonius (Lat.) — Roman god of west wind.  

*Description*: *Basidiocarps* arid, subiculum thin, filling depressions of substrate under the hymenial crust; well-developed hymenial surface smooth and solid, pale ochraceous to pale brown; margin arachnoid, indistinct, concolourous with hymenium. Substrate under old basidiocarps is often dark brown. *Hyphae* clamped, loosely interwoven in subiculum, 2–3 μm in diam, with walls 0.3–0.6 μm in width; tightly and vertically arranged in subhymenium, 2–2.5(–3) μm in diam, walls 0.3–0.4 μm in width. *Hymenium* simple, *basidia* clavate, 8–18 × 3.5–5 μm, with sterigmata up to 16 μm in length (n = 40/2). *Basidiospores* cylindrical, slightly curved, aseptate, (6.3–)7.1–10.3(–10.8) × 2.3–3.0(–3.1) μm, L = 8.3 μm, W = 2.7 μm, Q = 3.1, Q’ = 2.5–3.9 (n = 60/2), walls ∼ 0.2 μm in width.  

*Habitat and distribution*: Gymnosperm wood (*Abies*, *Picea*, *Pinus*, *Tsuga*); North America.  

*Material examined*: **Canada**, British Columbia, Fraser-Fort George reg. dist., McLeod lake co., pine forest near the McKenzie road, on *Pinus contorta*, 27 Jun. 1969, B. Eriksson & J. Eriksson 12222 (GB-0071216), 12223 (GB-0071215). **USA**, Oregon, Willamette National Forest, Frog Camp, on *Abies lasiocarpa*, 15 Jul. 1958, A.M. Rogers & D.P. Rogers 2811 (BPI1106574); Washington, Pend Oreille co., Gypsy Meadows, on *Picea engelmannii*, 17 Oct 2014, V. Spirin 8688b (H7008894∗); Wisconsin, Vilas co., on *Tsuga*, 16 Jul. 1964, A.E. Liberta 550 (BPI1106575).  

*Notes*: *Cerinomyces favonius* can be separated from other corticioid North American species by its narrower basidiospores and often by thinner basidiocarps. Though, specimen GB-0071216, that was identified as *C. favonius* primarily by the spores, has relatively robust basidiocarps and cylindrical hyphidia with thickened bases that arrange into microscopic hyphal pegs, which is more characteristic to the *C. pallidus* clade members. The closest phylogenetic relative to *C. favonius*, European *C. borealis*, features slightly larger basidiospores and basidia.  

**Fig. 33:**
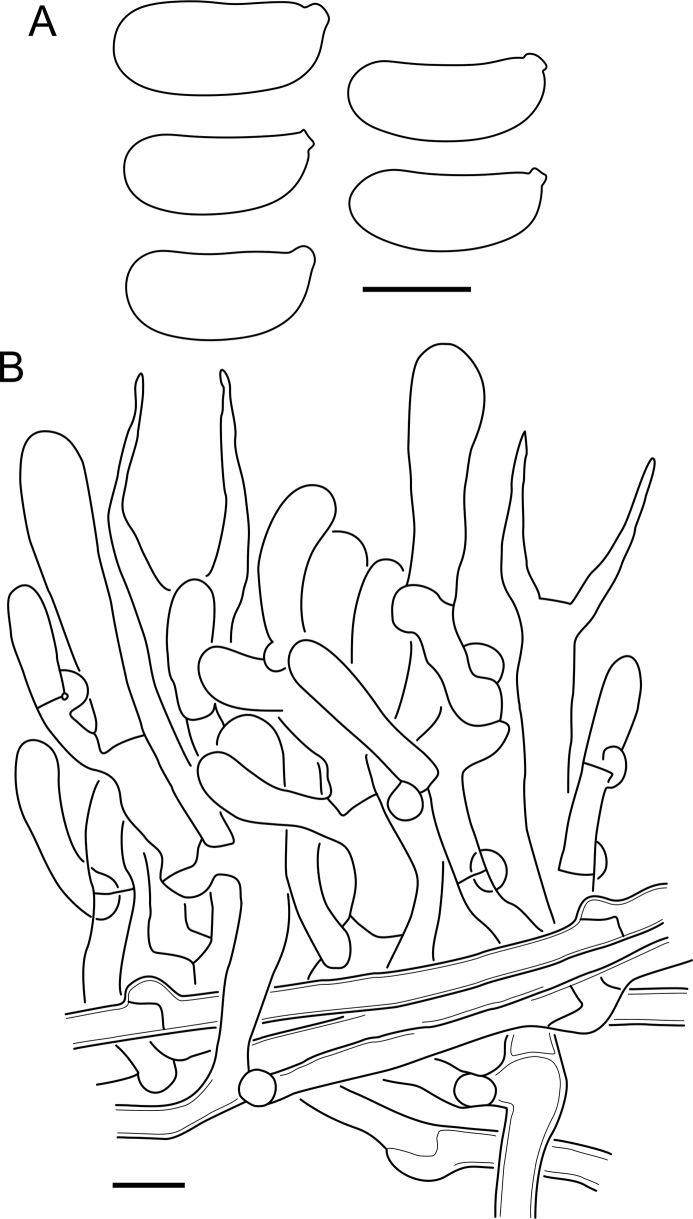
*Cerinomyces fugax* micromorphology. **A.** Spores. **B.** Hymenium and subhymenium. All drawn from holotype, CFMR:HHB-8856. Scale bars = 5 μm.

***Cerinomyces fugax*** A. Savchenko, ***sp. nov.*** MycoBank MB 839792. Fig. 10, Fig. 33.

*Typus*: **USA**, Mississippi, Stone co., 5 miles west of Wiggins, S of Red Rock, on *Pinus taeda*, 30 Mar. 1976, H.H. Burdsall (**holotype** CFMR:HHB-8856∗!).  

*Etymology*: fugax (Lat.) — shy, timid; reference to subtle basidiocarps.  

*Description*: *Basidiocarps* arid, appear as loose cottony subiculum patches, later covered with solid smooth hymenium, light ochraceous, without distinct margin. *Hyphae* clamped, subicular hyphae 2–3.5 μm in diam, walls 0.5 μm in width; in subhymenium 2–3 μm in diam, walls 0.3 μm in width. *Hymenium* simple, *basidia* clavate, 9–22 × 2.5–5 μm, with sterigmata up to 13 μm in length (n = 30/1). *Basidiospores* cylindrical, slightly curved, aseptate, (7.2–)7.3–9.3(–10.0) × (2.9–)3.0–3.8(–3.9) μm, L = 8.5 μm, W = 3.3 μm, Q = 2.6, Q’ = 2.4–3.0 (n = 30/1), walls ∼ 0.2 μm in width.  

*Habitat and distribution*: Gymnosperm wood (*Pinus*, *Pseudotsuga*); North America.  

*Material examined*: **Canada**, British Columbia, Vancouver Island, Lake Cowichan provincial forest, on *Pseudotsuga menziesii*, 6 Sep. 1967, B. Eriksson & J. Eriksson 7507 (GB-0071219).  

*Notes*: *Cerinomyces fugax* together with *C. favonius* and *C. tristis* form a difficult morphogroup of conifers-dwelling North American species. However, basidiospores of *C. fugax* are wider than of *C. favonius* and longer than of *C. tristis*. Micromorphologically, *C. fugax* is almost indistinguishable from its closest phylogenetic neighbor — European *C. volaticus*. Among the specimens that we were unable to sequence but tentatively identified as *C. fugax*, one has larger basidiospores 9.0–10.9 × 2.9–4.2 μm, L = 10.0 μm, W = 3.4 μm, Q = 3.0, basidia 13–20 × 2.5–4.5 μm, and sterigmata up to 18 μm in length; it can represent either a better developed *C. fugax* or yet another species in this complex (USA, Oregon, Cascade Head Expterimental Forest, Siuslau National Forest, on gymnosperm wood, 11 Oct. 1972, M.J. Larsen [CFMR:FP-133350]).  

**Fig. 34:**
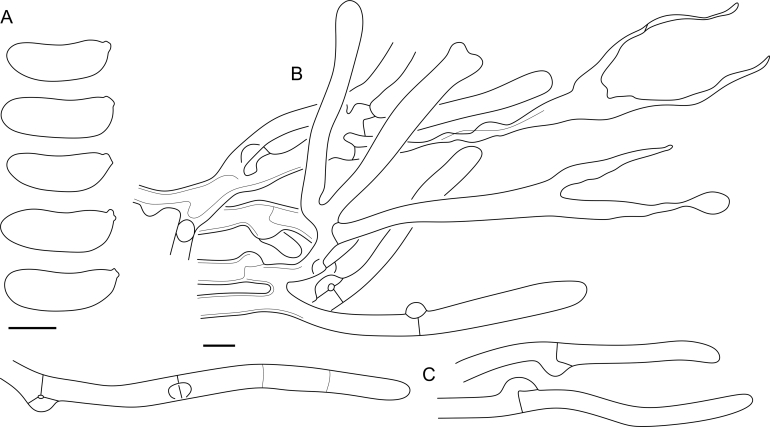
*Cerinomyces hesperidis* micromorphology. **A.** Spores. **B.** Hymenium and subhymenium. **C.** Marginal hyphae. All drawn from NY01782362. Scale bars = 5 μm.

***Cerinomyces hesperidis*** A. Savchenko, ***sp. nov.*** MycoBank MB 839793. Fig. 12, Fig. 34.

*Typus*: **USA**, Arizona, Pima co., Coronado National Forest, Santa Catalina Mountains, General Hitchcock Camp, on *Pinus ponderosa*, 31 Jan. 1968, R.L. Gilbertson 7794 (**holotype** NY01782362∗!).  

*Etymology*: hesperidis (Gr., latinized) — of evening, western.  

*Description*: *Basidiocarps* gelatinous, pustulate to shallow cupulate, sometimes slightly cerebriform, often with short stalk, up to 1 mm in diam, growing in groups, but usually not coalescing, from pale yellow-brown when fresh to dark brown when dried. Rarely young basidiocarps grow directly on older ones. *Hyphae* clamped, internal hyphae 2.5–4 μm in diam, walls ∼ 0.3 μm in width, with gelatinous layer 0.5–1.0 μm in width, sometimes roughened. Subhymenial hyphae (2–)2.5–3 μm in diam, walls ∼ 0.3 μm in width, with gelatinous layer up to 0.5 μm in width. Marginal hyphae simple cylindrical or slightly clavate, 2.5–3.5 μm in diam, walls of the same width as in subiculum. When stalks present, they covered in palisade of clavate or rarely slightly conical terminal cells 4–8 μm in diam, walls together with gelatinous layer ∼ 1.0 μm in width. *Hymenium* consists of basidia and rare cylindric *hyphidia*, simple or slightly branched. *Basidia* clavate, 23–41 × 2.5–4.5 μm. Sterigmata up to 35 μm in length (n = 30/1). *Basidiospores* slightly curved-cylindrical, 0(–1)-septate, (9.9–)10.0–12.3(–13.4) × (3.1–)3.2–4.2(–4.5) μm, L = 11.3 μm, W = 3.7 μm, Q = 3.0, Q’ = 2.6–3.9 (n = 30/1), walls ∼ 0.2 μm in width, collapsed basidiospores often constricted in the middle and have slightly attenuated apical part.  

*Habitat and distribution*: Gymnosperm wood (*Pinus* and unident.); North America.  

*Material examined*: **USA**, Montana, Flathead co., North Fork Flathead River, Glacier Nature Park, on *Pinus ponderosa*, 3 Aug. 1964, R.L. Gilbertson 4907 (NY01782364); New York, Warren co., Picnic area, north end of Pack Forest, on gymnosperm wood (?), 17 May 1968, H.H. Burdsall (CFMR:HHB-553)  

*Notes*: *Cerinomyces hesperidis* differs from European gelatinous *Cerinomyces* species by basidiocarps that are more regularly shaped, often with a central depression or of cupulate form, rooted in substrate with a short stalk, without a tendency to coalesce, resembling some collections of *C. tortus*. Cited here non-sequenced specimens fit well in the concept of *C. hesperidis*, but differ from the type by having thick-walled, pronouncedly clavate terminal hyphae covering basidiocarp stems.  

**Fig. 35:**
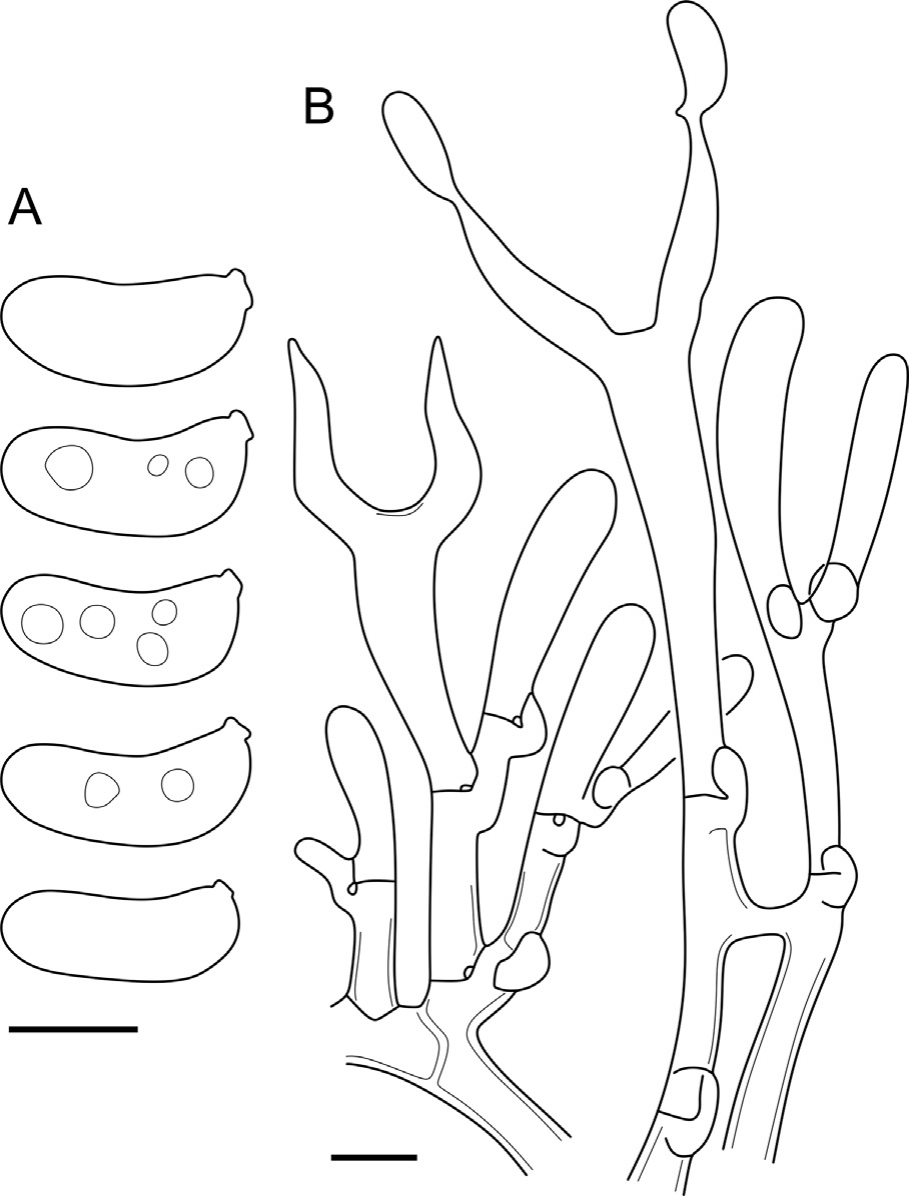
*Cerinomyces inermis* micromorphology. **A.** Spores. **B.** Hymenium and subhymenium. All drawn from holotype, PDD87816. Scale bars = 5 μm.

***Cerinomyces inermis*** A. Savchenko, ***sp. nov.*** MycoBank MB 839794. Fig. 10, Fig. 35.

*Typus*: **New Zealand**, Te Ika-a-Māui, Bay of Plenty reg., Te Urewera, Tarapounamu, UA 2 NW 16m, on gymnosperm wood, 10 Oct. 2004, P.R. Johnston & B.C. Paulus 752 (**holotype** PDD87816∗!).  

*Etymology*: inermis (Lat.) — unarmored, referring to the absence of hyphal pegs.  

*Description*: *Basidiocarps* originate as arid white subicular patches, turning into smooth and solid, light yellowish brown to dark brown, semitransparent, thin film, waxy when moisture applied, with thin subiculum and white fimbriate margins. Hyphal pegs absent. *Hyphae* clamped, gelatinized, subicular hyphae 2–3(–4) μm in diam, walls ∼ 0.3 μm in width; in subhymenium 1.5–3 μm in diam, walls 0.3–0.5 μm in width. *Hymenium* simple, *basidia* clavate, 12–25 × 3–5 μm, with sterigmata up to 21 μm in length (n = 30/1). *Basidiospores* cylindrical, slightly curved, often with lipid droplets within, aseptate, (8.1–)8.2–10.2(–10.6) × (2.9–)3.0–3.9 μm, L = 9.5 μm, W = 3.3 μm, Q = 2.9, Q’ = 2.3–3.4 (n = 30/1), walls ∼ 0.2–0.3 μm in width.  

*Habitat and distribution*: Gymnosperm wood; New Zealand (known only from the type locality).  

*Notes*: *Cerinomyces inermis* can be confused with the *C. pallidus* clade members, but it has averagely longer basidiospores. In contrast to its relatives from the *C. albosporus* clade, basidiocarps of *C. inermis* lack hyphal pegs and are prone to gelatinize when re-wetted, which puts it close to *C. aff. aculeatus 1* morphology-wise.  

**Fig. 36:**
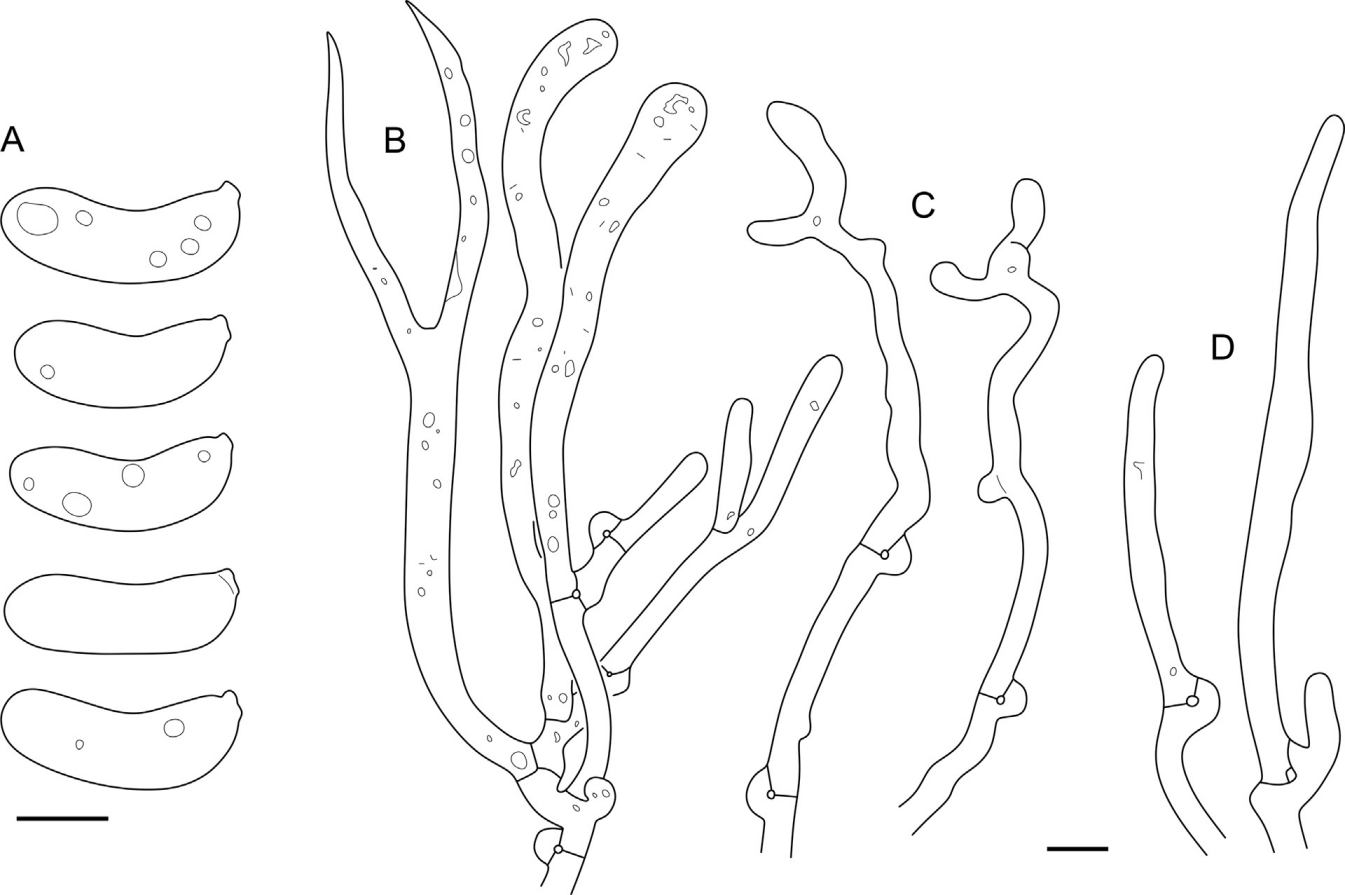
*Cerinomyces lipoferus* micromorphology. **A.** Spores. **B.** Hymenium and subhymenium. **C.** Hyphidia. **D.** Marginal hyphae. All drawn from GB-0161225. Scale bars = 5 μm.

***Cerinomyces lipoferus*** J.C. Zamora & A. Savchenko, ***sp. nov.*** MycoBank MB 839795. Figs 13 A, B, 36.

*Typus*: **Sweden**, Uppsala co., Uppsala mun., Stadsskogen Nature Reserve, on *Pinus sylvestris*, 26 Sep. 2018, J.C. Zamora & L. Martín (**holotype** UPS:F-940777∗!, **isotype** H7009042!).  

*Etymology*: λίπος (Gr.) — fat; φέρω (Gr.) — carry; reference to a large amount of lipid droplets.  

*Description*: *Basidiocarps* gelatinous, pustulate, gregarious, can coalesce with shapes of single basidiocarps remaining visible, whitish to pale yellow or cream coloured, rarely pale brown when fresh, yellowish brown to dark brown when dried, single basidiocarps up to 1.5(–2) mm in diam. *Hyphae* clamped, with comparatively high content of lipid droplets; clamps often have a hole under the loop (see Fig. 36 B–D). Subicular hyphae 2–3.5(–4.5) μm in diam, walls 0.3–0.5 μm in width, gelatinized and sometimes roughened. Subhymenial hyphae densely arranged, (1.5–)2–3.5 μm in diam, walls 0.3–0.4 μm in width. Sterile areas and margins covered with simple cylindrical, slightly inflated or narrowly clavate hyphae 2–4 μm in diam, walls of the same width as in subiculum. *Hymenium* includes rare, weakly branched cylindrical *hyphidia* 2–3.5 μm in diam, and clavate *basidia* 19–62 × 3–6 μm. Sterigmata up to 34 μm in length (n = 54/2). *Basidiospores* slightly curved-cylindrical, 0–1(–3)-septate, at least some binucleate before septation (but a few uninucleate basidiospores also seen), (9.1–)9.5–15.2(–16.8) × (3.7–)3.9–5.2(–5.7) μm, L = 11.8 μm, W = 4.5 μm, Q = 2.6, Q’ = 2.1–3.6 (n = 155/2), walls ∼ 0.2 μm in width.  

*Habitat and distribution*: Gymnosperm wood (*Picea*, *Pinus*, and unident.), Northern Europe.  

*Material examined*: **Sweden**, Uppsala co., Uppsala mun., Stadsskogen Nature Reserve, on *Pinus sylvestris*, 24 Oct. 2018, J.C. Zamora & L. Martín (UPS:F-940778∗), same loc., 15 Oct. 2019, J.C. Zamora & M. Westberg (UPS:F-946513); Västra Götaland co., Göteborg city, Haga area, Haga Nygata nr., on vertical wood wall, 12 Nov. 1976, J. Eriksson (GB-0161225∗ — herb. GB scripsit “19548”). **The Netherlands**, Gelderland, Hoge Veluwe, Deelense Straal, on *Pinus*, 5 Jan. 2020, N. Dam ND20004 (L.3983554); Overijssel, near Enschedé, Haagse Bos, on *Picea abies*, 26 Jan. 2020, R. Enzlin ENZ20001∗ (herb. Enzlin).  

*Notes*: The species resembles *C. tortus* in rarely branched hyphidia but differs in high number of lipid bodies in hyphae, presence of 1- or rarely 3-septate basidiospores, and paler fruitbodies. Zamora & Ekman (2020) referred to the species as to *Dacrymyces tortus s. lat. 1.* Sequences of ENZ20001 were produced during the revision of this study; they were not used in the analyses but match other included sequences.  

 Similarly large amount of lipid drops and matching spore measurements were observed in a specimen from a montane habitat (China, Jilin, Huang Song Pu, Chang Bai Shan Forest Reserve, 1 200 m a.s.l., on *Larix olgensis*, 11 Sep. 1983, L. Ryvarden 21458 [K(M): 34585]). We do not assign it to *C. lipoferus* due to the geographical distance between occurrences.  

**Fig. 37:**
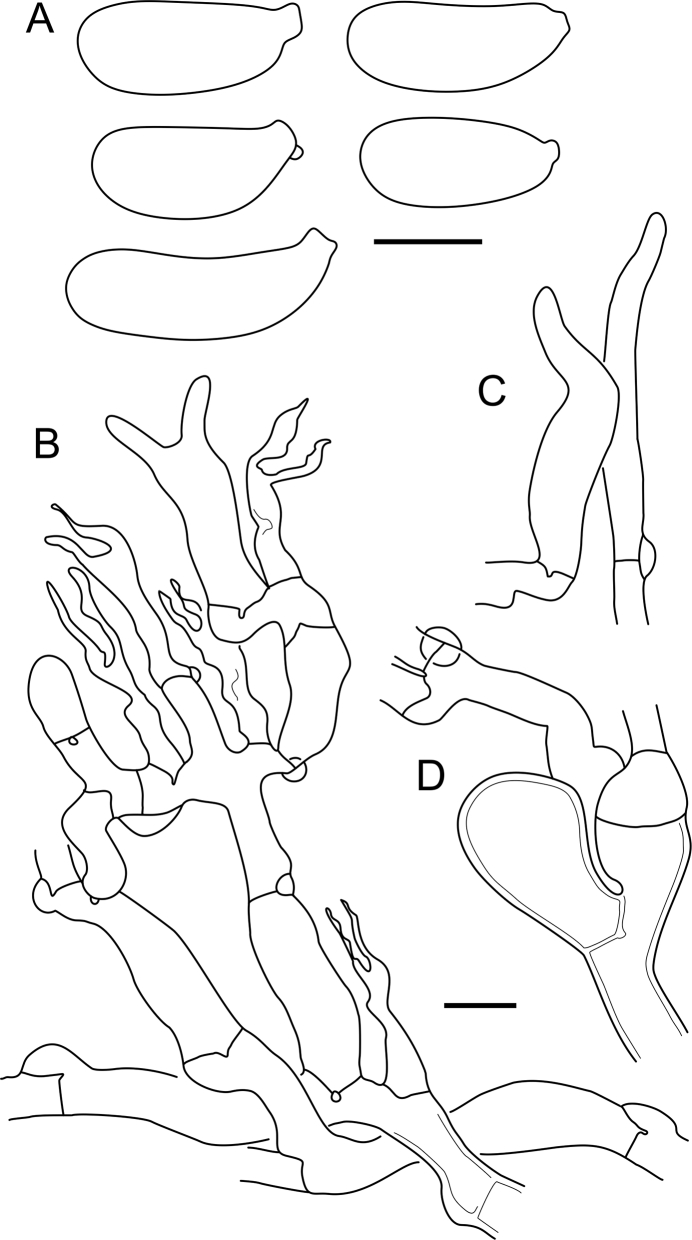
*Cerinomyces nepalensis* micromorphology. **A.** Spores. **B.** Hymenium, subhymenium and subiculum. **C.** Hyphidia. **D.** Subhymenial hyphae with swollen cells. All drawn from holotype, O:F-904088. Scale bars = 5 μm.

***Cerinomyces nepalensis*** A. Savchenko, ***sp. nov.*** MycoBank MB 839796. Fig. 10, Fig. 37.

*Typus*: **Nepal**, Dhaulagiri zone, Myagdi dist., Ghorepani, 30 Oct. 1979, L. Ryvarden 18670B (**holotype** O:F-904088∗!).  

*Etymology*: Reference to the type collection locality.  

*Description*: *Basidiocarps* arid, solid and smooth, with crustose pale fawn hymenial surface and cottony white subiculum. *Hyphae* clamped, in subiculum agglutinated in strands, loose, 2–4 μm in diam, walls 0.3–0.6 μm in width. Subhymenial hyphae of the same diam, walls up to 0.8 μm in width, decreasing to 0.3 μm towards hymenium, with occasional swollen cells. *Hymenium* includes *hyphidia* with cylindrical to obclavate base up to 4 μm in diam and simple apical part ∼ 1.5 μm in diam; of total length up to 25 μm, in groups or scattered among basidia. *Basidia* clavate to almost cylindrical, 7–17 × 2.5–4.5 μm. Sterigmata up to 10(–15) μm in length (n = 30/1). *Basidiospores* ellipsoid to slightly curved-cylindrical, often with long apiculus and distinct hilar appendix, aseptate, (5.0–)5.2–7.7(–7.9) × (2.1–)2.5–3.4(–3.5) μm, L = 6.6 μm, W = 3.0 μm, Q = 2.2, Q’ = 1.8–2.8 (n = 30/1), wall ∼ 0.2 μm in width.  

*Habitat and distribution*: Unidentified, probably gymnosperm wood; South Asia (known only from the type locality).  

*Notes*: The species has the shortest basidiospores in the genus. In our opinion, pronounced apicular part of the basidiospores (and large space between hilum and its appendix) can indicate their immaturity, so the reported measurements may not represent the full range for the trait. Type locality of *C. pinguis* is also in Nepal, but this species has substantially larger microstructures than *C. nepalensis*. The type specimen was first reported as *C. pallidus* by Hjortstam & Ryvarden (1984).  

**Fig. 38:**
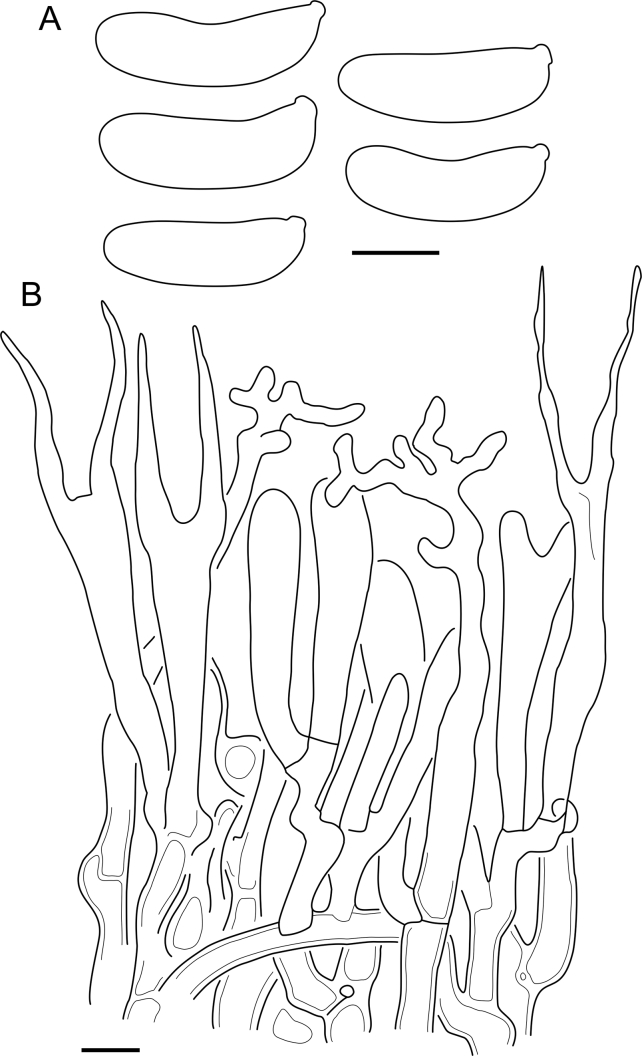
*Cerinomyces neuhoffii* micromorphology. **A.** Spores. **B.** Hymenium with hyphidia, and subhymenium. Drawn from TU135067 (A); CWU(MYC)6342 (B). Scale bars = 5 μm.

***Cerinomyces neuhoffii*** J.C. Zamora & A. Savchenko, ***sp. nov.*** MycoBank MB 839797. Fig. 13, Fig. 38.

*Typus*: **Sweden**, Stockholm co., Stockholm, Årsta, on *Pinus sylvestris*, 10 Sep. 2017, J.C. Zamora, P. Posio (**holotype** UPS:F-941020∗!, **isotype** H7009709!).  

*Etymology*: In honour of the German mycologist Walther Neuhoff.  

*Description*: *Basidiocarps* gelatinous, pustulate to pulvinate, rarely slightly cerebriform or centrally depressed, growing in dense groups, often coalescing with shapes of initial basidiocarps remaining visible. Rarely, basidiocarps stay clearly isolated and only gregarious. Separate basidiocarps up to 1(–1.5) mm in diam, from pale brown when fresh and young to dark brown or dark greyish brown, in maturity with or without a whitish subicular core, becoming almost black when dried. *Hyphae* clamped, gelatinized, densely arranged, in subiculum 2.5–4(–5) μm in diam, walls 0.4–1.0 μm in width. Subhymenial hyphae 2–3.5 μm in diam, walls 0.3–0.6 μm in width. Marginal hyphae similar to subicular, simple cylindrical or slightly clavate, gelatinized. Some branched anastomosing hyphae observed between fertile and sterile areas. *Hymenium* consists of basidia and well-branched cylindrical *hyphidia* with base 2–3.5 μm in diam and apical part 1.5–2 μm in diam; up to 60 μm in total length, from occasional to abundant in different basidiocarps. *Basidia* clavate, some of them slightly curved,14–43 × 3–7 μm, with walls up to 0.5 μm in width. Sterigmata up to 26 μm in length (n = 120/8). *Basidiospores* slightly curved-cylindrical, aseptate, binucleate, (8.2–)9.6–14.0(–16.1) × (2.9–)3.0–4.8(–5.1) μm, L = 11.6 μm, W = 3.8 μm, Q = 3.1, Q’ = 2.3–3.9 (n = 180/9), walls ∼ 0.2–0.3 μm in width. Conidia subglobose, 1.8–2.1 μm in diam.  

*Habitat and distribution*: Gymnosperm wood (*Pinus* and unident.), Europe.  

*Material examined*: **Cyprus**, Limassol, Mesa Potamos, picnic area, on *Pinus brutia*, 2 Dec. 2017, J.C. Zamora (UPS:F-946505∗). **Finland**, Uusimaa prov., Helsinki, Myllypuro, on gymnosperm wood, 10 Jun. 2017, O. Miettinen 20778 (H∗). **Spain**, Andalucía comm., Jaén, Venta de los Santos, close to the Dañador dam, on *P. pinaster*, 15 May 2017, B. Zamora, J. Señoret (UPS:F-941019∗), Málaga comm., Mijas village area, on *Pinus*, 16 Nov. 2012, O. Miettinen 15893 (H∗). **Sweden**, Kalmar co., Öland, Borgholm mun., Bödakusten västra Nature Reserve, Byrums Sandvik, on *P. sylvestris*, 2 Oct. 2017, J.C. Zamora (UPS:F-946503∗); Stockholm co., Stockholm, Masmo, on *P. sylvestris*, 15 Oct. 2017, J.C. Zamora & P. Posio (UPS:F-941252), Solna mun., Bergshamra, on *P. sylvestris*, 23 Jun. 2018, J.C. Zamora (UPS:F-941021); Uppsala co., Uppsala mun., Norra Lunsen Nature Reserve, on *P. sylvestris*, 20 Aug. 2017, J.C. Zamora (UPS:F-946501∗); Värmland co., Lekvattnet, on *P. sylvestris*, 7 Oct. 2018, J.C. Zamora (UPS:F-946510∗). **Ukraine**, Kharkiv reg., Krasnokutsk dist., Slobozhanskyi National Park, forest lot 42, on *P. sylvestris*, 2 Nov. 2013, A. Savchenko (CWU(MYC)6342∗), forest lot 58, on *P. sylvestris*, 1 Dec. 2012, A. Savchenko (CWU(MYC)6281∗); Ternopil reg., Zalishchyky dist., Dzhuryn river valley, on *Pinus*, 5 Oct. 2016, O. Akulov AS0060 (CWU(MYC), TU135067∗).  

*Notes*: *Cerinomyces neuhoffii* and *C. tortus* are among the most common gelatinous *Cerinomyces* species. The former one is widespread in Europe, while the latter occurs mostly in its northern part. Basidiocarps of *C. neuhoffii* are generally smaller, darker, with a softer consistency, and often organized in large, closely crowded groups. On microscopic level, *C. tortus* very rarely forms dendroid hyphidia, in contrast to the finely branched, abundant structures of *C. neuhoffii*. Also, *C. neuhoffii* has shorter and stouter basidia, and at least some of them tend to be slightly bent or asymmetric. *Cerinomyces creber* is highly similar to *C. neuhoffii* in hyphidia and gregarious coalescing basidiocarps, but it is often more light-coloured, has smaller basidiospores, slightly smaller basidia, and seems to be associated with *Cupressaceae*. Two undescribed taxa designated below as *C. aff. tortus 1* and *2* both have branched hyphidia, but their basidiospores are on average shorter than of *C. neuhoffii.* We also found that basidiospores of *C. neuhoffii* specimens from Southern and Eastern Europe are slightly wider compared to the Northern collections. Zamora & Ekman (2020) cited the species as *Dacrymyces tortus s. lat. 4.*  

 Neuhoff (1934) actually distinguished two putative species (*D. punctiformis* and *D. romelii*) in what had been previously considered as *Dacrymyces tortus s.l.* Although the original descriptions may partially cover the morphological characteristics of *C. neuhoffii*, they also fit within the variation of *C. tortus*. The type specimens of both *D. punctiformis* and *D. romelii* turned out to be conspecific to *C. tortus* (see note under that species), so in absence of available names we describe *C. neuhoffii* as a new species, while honouring Neuhoff’s clue that *C. tortus* has to be split.  

**Fig. 39:**
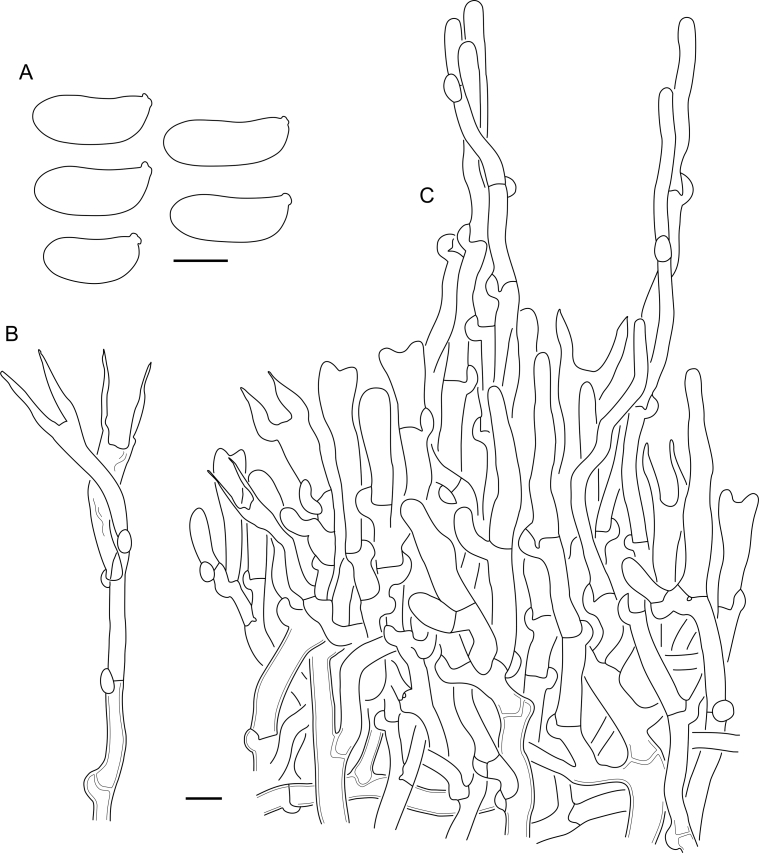
*Cerinomyces pallidus* micromorphology. **A.** Spores. **B.** Single hypha followed from subiculum to hymenium. **C.** Hymenium, subhymenium, subiculum and two developing hyphal pegs. All drawn from BPI726052. Scale bars = 5 μm.

***Cerinomyces pallidus*** G.W. Martin, Mycologia 41: 83 (1949). Fig. 10, Fig. 39.

*Typus*: **USA**, Iowa, Johnson co., Iowa City, on *Quercus*, 28 Jul. 1939, G.W. Martin 4673 (**holotype** TRTC, **isotypes** BPI726049! ex US, BPI726050! ex MO186440, FH00489909!, ISC-V-0045114, K, LSU00135942 ex MO186440, NY02136493!, UC940258).  

*Description*: *Basidiocarps* arid, appear as small white patches of cottony subiculum, later coalescing, covering areas up to 15 × 3 cm or more (*fide* Martin 1949), 0.1–0.5 mm thick. Well-developed hymenial surface solid, crust-like, light ochraceous, becoming pale fawn to pale brown in older, prominent, and bruised areas; smooth or irregularly covered with *hyphal pegs* up to 300 μm in height. Margin fimbriate or indistinct, concolourous with hymenium surface; subiculum relatively thick, filling depressions of substrate under the hymenial crust. Older basidiocarps can be found under new ones as collapsed dark brown to black layers. *Hyphae* clamped, in subiculum interwoven, 2–3 μm in diam, walls 0.5–0.6(–0.8) μm in width; in subhymenium mostly perpendicular to substrate, 2–3.5 μm in diam, walls 0.3–0.5(–0.7) μm in width. Pegs formed by parallel, compact and often agglutinated hyphae 1–2.5 μm in diam, walls 0.3–0.7 μm in width. *Hymenium* consists of basidia and rare *hyphidia* with thickened base up to 5 μm in diam and simple cylindrical apical part 2–2.5 μm in diam; hyphidia tend to form small groups, developing into microscopic hyphal pegs. *Basidia* clavate, 10–27 × 2.5–5(–6.5) μm, with sterigmata up to 19 μm in length (n = 116/6). *Basidiospores* cylindrical, slightly curved, aseptate, (6.1–)6.5–10.2(–11.3) × 2.9–4.1(–4.8) μm, L = 8.0 μm, W = 3.3 μm, Q = 2.4, Q’ = 1.7–3.5 (n = 176/6), walls ∼ 0.2 in width.  

*Habitat and distribution*: Angiosperm (*Malus*, *Quercus*, and unident.), very rarely gymnosperm (*Pinus*) wood; North America.  

*Material examined*: **USA**, Iowa, Johnson co., Iowa City, on *Malus*, 21 Aug. 1939, G.W. Martin 3916 (original material, BPI726052), on *Quercus*, 1 Nov. 1940, G.W. Martin 5180 (original material, GB-0071214∗); Kansas, Leavenworth co., Ravine near Piper river, on angiosperm wood, 29 Sep. 1972, T.R. Rockett (NY: as “C. pallidus №1”∗); Louisiana, East Feliciana par., [Bob R. Jones-] Idlewild Research Station of LSU, on *Pinus taeda*, 23 Aug. 1986, R.L. Gilbertson 15966, AN027337 (ARIZ-M-AN09245∗); Maryland, Prince Georges co., Hoffman Hill Rd., Beltsville Expt. Forest, on *Quercus*, 6 Nov. 1968, H.H. Burdsall (CFMR:HHB-1768); Ohio, Delaware co., Camp Lazarus Reserve, on angiosperm wood, 2 Sep. 1968, W.B. Cooke & V.G. Cooke (CFMR:WBC-39924∗).  

*Notes*: *Cerinomyces pallidus* can be identified by association with angiosperm substrates and by the presence of pegs in mature basidiocarps. It shares these features with *C. paulistanus*, but the latter species occurs only in South America and slightly differs in morphology. In some collections, pegs can be very rare or even absent, leading to confusion with another North American angiosperm-inhabiting species, *C. atrans*, that has similar micromorphology, but darker basidiocarps. Both species can occasionally appear on gymnosperm wood, and then it is difficult to tell them apart from strictly conifers-dwelling taxa.  

 In the protologue of *C. pallidus* Martin assigned a “type” and, perhaps already after publication, distributed its duplicates to a number of herbaria. Maekawa (1987) cited a holotype deposited in TRTC, but the specimen could not be traced (S. Margaritescu, 14 Sep. 2016, pers. comm.). Worth noting, that under the same number 4673, Martin put another gathering from 28 July of 1948 (ISC-V-0045115), also marked as a type. Besides of the same locality (Iowa City) and substrate (oak), it is unknown how exactly the specimen is connected to the original type material from 1939. Martin (1949) reported ellipsoid conidia produced at the tops of its hyphal pegs, but we did not see any anamorphic structures in *C. pallidus*.  

 A non-sequenced specimen UBC:F873 (Canada, British Columbia) deserves a specific mention having unusually short, ellipsoid basidiospores (6.5–8 × 3–4 μm, L = 7.4 μm, W = 3.6 μm, Q = 2.1, Q’ = 1.6–2.6, n = 16/1), and can represent either an immature *C. pallidus* or a potential new species. Sequence of ARIZ-M-AN09245 was produced during the revision of this study; it was not used in the analyses but matches other included sequences.  

**Fig. 40:**
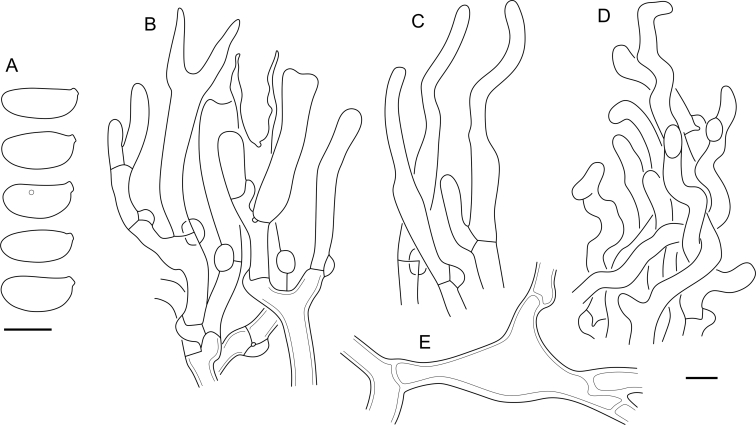
*Cerinomyces paulistanus* micromorphology. **A.** Spores. **B.** Hymenium and subhymenium. **C**. Hyphidia. **D.** Apical part of a hyphal peg. **E.** Swellings on subicular hyphae. Drawn from TAAM192129 (A, B); holotype, O:Ryvarden 24759 (C–E). Scale bars = 5 μm.

***Cerinomyces paulistanus*** A. Savchenko, ***sp. nov.*** MycoBank MB 839798. Fig. 10, Fig. 40.

*Typus*: **Brazil**, São Paulo, Cananéia, Parque Estadual Ilha do Cardoso, 2–5 Feb. 1987, D.N. Pegler, K. Hjortstam & L. Ryvarden 24759 (**holotype** O∗!, **isotype** TU135088!).  

*Etymology*: Reference to the type collection locality.  

*Description*: *Basidiocarps* arid, appear as white arachnoid patches that develop into a relatively thick whitish subiculum. Hymenial surface arachnoid to solid, from pale ochraceous to brown in older or damaged areas. Margin fimbriate, concolourous with subiculum. Well-developed basidiocarps covered with scattered *hyphal pegs* 300(–500) μm in height. *Hyphae* clamped, subicular hyphae 2–3.5(–6) μm in diam, with occasional swellings up to 10 μm in diam, walls 0.3–0.8 μm in width; in subhymenium 2–4 μm in diam, walls 0.2–0.6 μm in width. Hyphae inside of pegs 3–4 μm in diam, originating from subiculum and becoming gradually thinner and more thin-walled towards the top of peg, being 2–3(–4) μm in diam on sides and at the top. *Hymenium* consists of basidia and *hyphidia* that have slightly swollen base 2.5–3 μm in diam and simple cylindrical apical part 1.5–2 μm in diam; ≤ 50 μm in total length. Hyphidia tend to arrange in agglutinated groups, microscopic pegs. *Basidia* clavate, 10–23 × 2.5–5 μm, with sterigmata up to 13 μm in length (n = 49/7). *Basidiospores* cylindrical, slightly curved, aseptate, (6.0–)6.5–9.6(–11.2) × (2.5–)2.6–3.9(–4.4) μm, L = 7.6 μm, W = 3.3 μm, Q = 2.3, Q’ = 1.8–3.1 (n = 126/7, 91 measured in 1 % KOH), walls ∼ 0.2 μm in width.  

*Habitat and distribution*: Unidentified, probably angiosperm wood; South America.  

*Material examined*: **Brazil**, São Paulo, Parque do Estado, [near] Instituto de Botânica, 18 Mar. 1964, K. Wells (all specimens) 1380-2 (TAAM192122), 1380-3 (BPI726051), (LSU00173761), 2 Apr. 1964, 1391-3 (BPI726053), 1391-4 (TAAM192121), 6 May 1964, 1542-2 (TAAM192120∗), 1542-4 (BPI726054).  

*Notes*: *Cerinomyces paulistanus* is a close relative to *C. pallidus* in phylogeny, being also highly similar morphologically, but having slightly shorter basidia, swellings in subicular hyphae, and occurring in South instead of North America. They are the only two species in *Cerinomyces* outside of the *C. albosporus* clade that develop macroscopic hyphal pegs, though we noted that in *C. paulistanus* many of otherwise well-developed basidiocarps have no such pegs.  

**Fig. 41:**
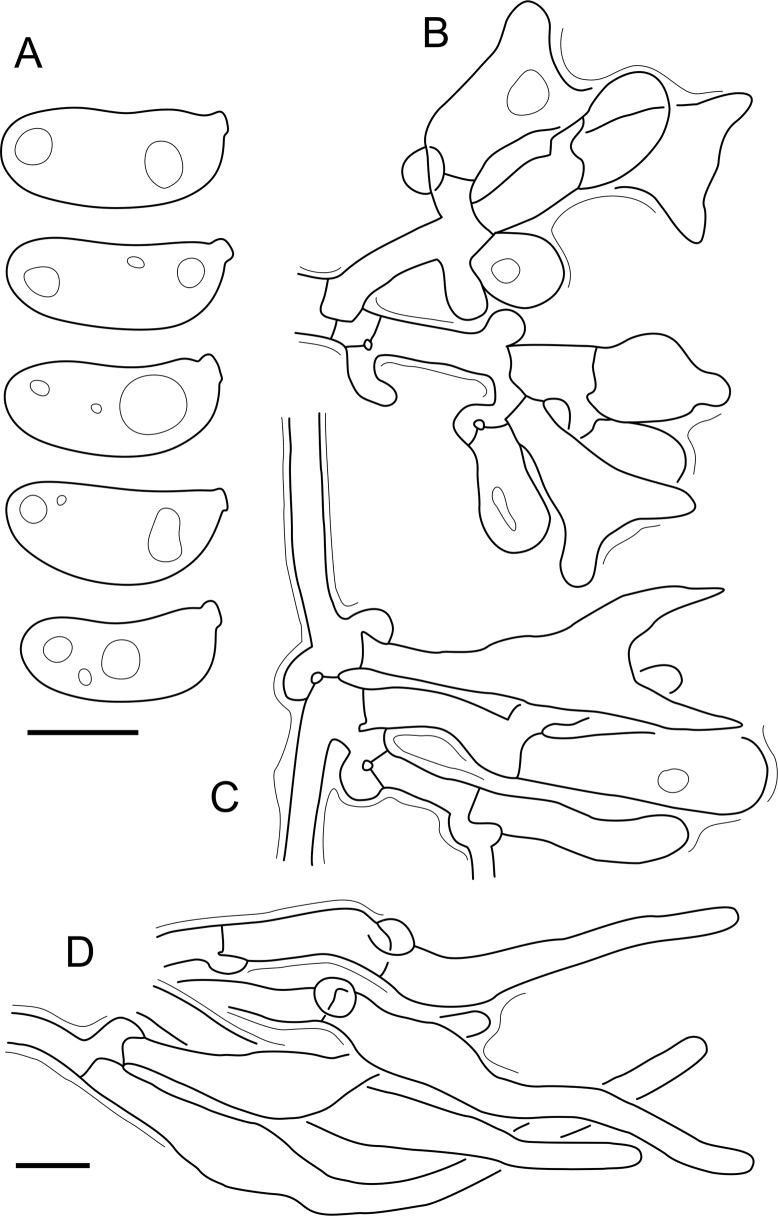
*Cerinomyces pinguis* micromorphology. **A.** Spores. **B.** Hymenium with short gelatinized basidia. **C.** Gelatinized hymenium with full-sized basidia, and subhymenium. **D.** Group of hyphidia. All drawn from holotype, O:F-904085. Scale bars = 5 μm.

***Cerinomyces pinguis*** A. Savchenko, ***sp. nov.*** MycoBank MB 839799. Fig. 10, Fig. 41.

*Typus*: **Nepal**, Gandaki zone, Kaski dist., Annapura trek, Kuldi [Khuldighar?], 7 Nov. 1979, L. Ryvarden 18922 (**holotype** O:F-904085∗!).  

*Etymology*: pinguis (Lat.) — fat; reference to the shapes of some basidia.  

*Description*: *Basidiocarps* arid, arachnoid, white to pale fawn, without distinct subiculum or margin, hardly visible by the naked eye. *Hyphae* clamped, not differentiated. Subhymenial hyphae 2–3 μm in diam, walls 0.3–0.5 μm in width, gelatinized, agglutinated in strands closer to substrate. *Hymenium* includes *hyphidia* with thickened base 3–5 μm in diam and simple cylindrical apical part 1.5–2 μm in diam; up to 50 μm in total length. *Basidia* clavate to broadly clavate, 10–21 × 4.5–6.5 μm. Sterigmata up to 12(–16) μm in length (n = 30/1), sometimes covered with gelatinous layer. *Basidiospores* slightly curved-cylindrical, aseptate, (8.7–)9.0–10.5(–12.6) × 3.5–4.5(–5.0) μm, L = 9.8 μm, W = 3.9 μm, Q = 2.5, Q’ = 2.2–2.9 (n = 30/1), walls ∼ 0.2 μm in width.  

*Habitat and distribution*: Unidentified, probably gymnosperm wood; South Asia (known only from the type locality).  

*Notes*: *Cerinomyces pinguis* has the largest basidiospores and arguably the most delicate basidiocarps among the peg-lacking corticioid *Cerinomyces* species. The type specimen was originally reported as *C. crustulinus* by Hjortstam & Ryvarden (1984).  

**Fig. 42:**
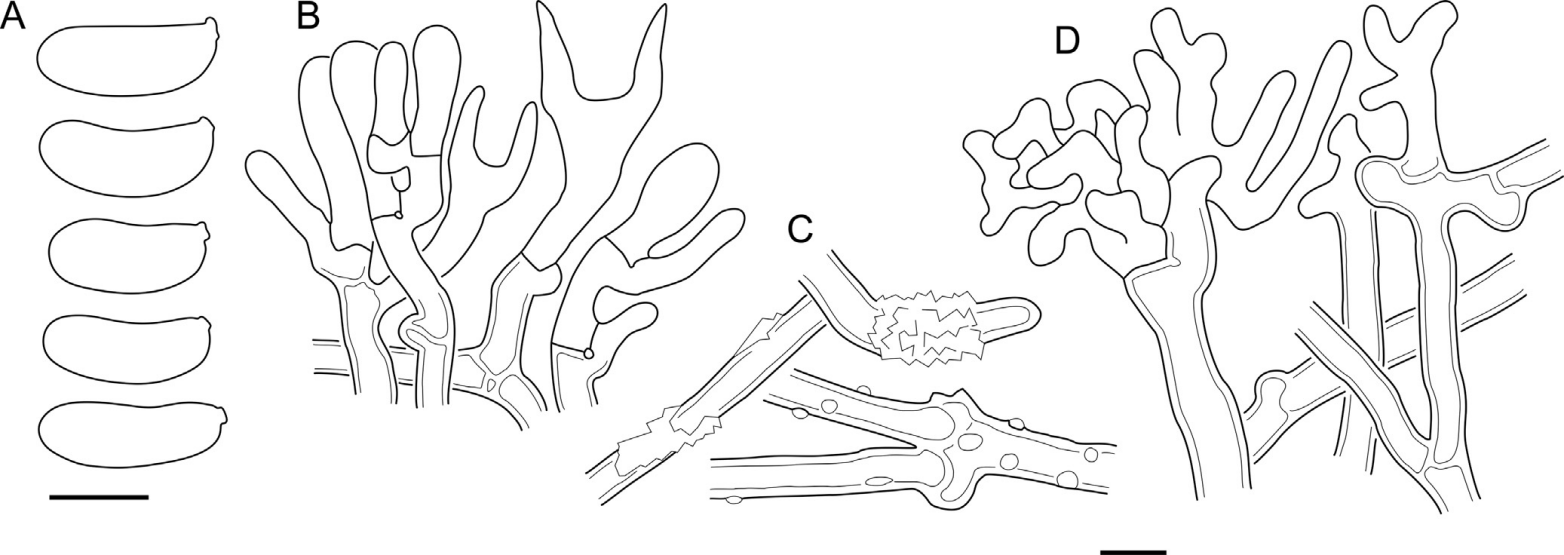
*Cerinomyces ramosissimus* micromorphology. **A.** Spores. **B.** Hymenium and subhymenium. **C.** Subicular hyphae with crystals. **D.** Dendroid hyphae covering sterile areas. All drawn from holotype, CFMR:FP-150848. Scale bars = 5 μm.

***Cerinomyces ramosissimus*** A. Savchenko, ***sp. nov.*** MycoBank MB 839800. Fig. 10, Fig. 42.

*Typus*: **Belize**, Cayo dist., Mountain Pine Ridge, Five Sisters Lodge, Lower Nature Train, on *Pinus*, 20 Nov. 2001, K. Nakasone (**holotype** CFMR:FP-150848∗!).  

*Etymology*: ramosissimus (Lat.) — very much branched; reference to a structure of subhymenium.  

*Description*: *Basidiocarps* arid, at first arachnoid, later covered with solid smooth hymenium, light ochraceous to brown in damaged areas, with cottony whitish subiculum and indistinct margin. *Hyphae* clamped, densely packed, in subiculum often arranged in threads, 2–4 μm in diam, walls 0.8–1.0 μm in width, bearing crystals: from scattered transparent pustulate ≤ 2 μm in height to amorphic brownish coating. In some subicular areas crystals can be completely absent. Subhymenium develops from subiculum as tangles of ramificated hyphae. Subhymenial hyphae well-branched and tightly arranged, 2–3 μm in diam, walls 0.3–0.6 μm in width. Some sterile areas covered with the same type of branched hyphae. *Hymenium* consists of rare robust weakly branched *hyphidia* and clavate *basidia*, 8–16 × 3–5 μm. Sterigmata up to 11 μm in length (n = 30/1). *Basidiospores* slightly curved-cylindrical, aseptate, (7.0–)7.3–9.6 × (2.7–)2.8–3.2(–3.4) μm, L = 8.3 μm, W = 3.0 μm, Q = 2.8, Q’ = 2.4–3.3 (n = 30/1), walls ∼ 0.2 μm in width.  

*Habitat and distribution*: Gymnosperm wood (*Pinus*); Central America (known only from the type locality).  

*Notes*: The species is characterized with highly branched subhymenial hyphae and presence of crystals.  

**Fig. 43:**
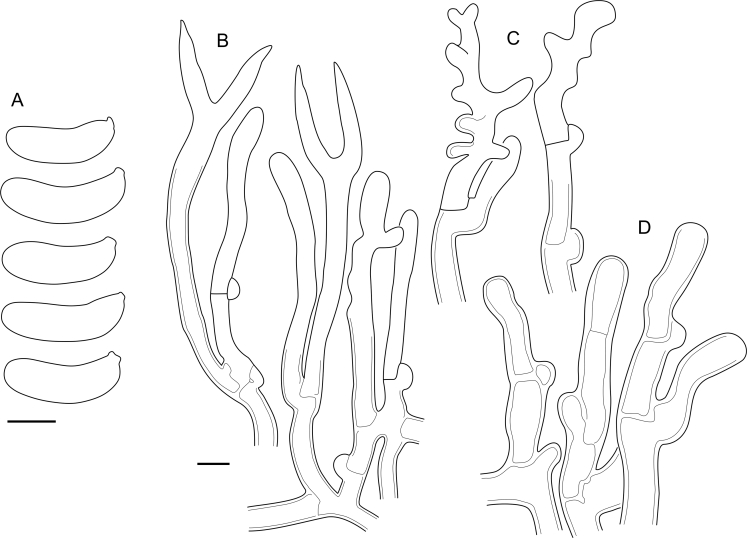
*Cerinomyces tortus* micromorphology. **A.** Spores. **B.** Hymenium with a single hyphidium, and subhymenium. **C.** Hyphidia. **D.** Marginal hyphae. All drawn from *Dacrymyces punctiformis* holotype, UPS:F-015301. Scale bars = 5 μm.

***Cerinomyces tortus*** (Willd.) Miettinen, J.C. Zamora & A. Savchenko, ***comb. nov.*** MycoBank MB 839813. Fig. 13, Fig. 43.

*Basionym: Tremella torta* Willd., Botanisches Magazin 2 (4): 18 (1788).

*Synonyms: Dacrymyces tortus* (Willd.) Fr., Elenchus Fungorum 2: 36 (1828), *nom. sanct*.

*Dacrymyces deliquescens var. castaneus* Bourdot, Bulletin de la Société Mycologique de France 48: 206 (1932).

*Dacrymyces punctiformis* Neuhoff, Schweizerische Zeitschrift für Pilzkunde: 81 (1934).

*Dacrymyces romellii* Neuhoff, Schweizerische Zeitschrift für Pilzkunde. 12: 82 (1934).

*Dacrymyces tortus f. romellii* (Neuhoff) Raitv., Живая природа Дальнего Востока: 131 (1971).  

*Typus*: **Sweden**, Uppsala co., Uppsala mun., Norra Lunsen Nature Reserve, on *Pinus sylvestris*, 24 May 2019, J.C. Zamora & S. Ekman (**neotype** [designated here] UPS:F-946515∗!, **isoneotypes** H7009710!, TU135086!). MycoBank typification MBT10001415.  

*Description*: *Basidiocarps* gelatinous, first pustulate, then flattened, often centrally depressed, discoid or slightly cerebriform, sometimes with short stalk. Gregarious, occasionally can coalesce with shapes of single basidiocarps remaining visible; separate basidiocarps up to 3 mm in diam. Colour from translucent light yellow to brown with lighter internal core of subiculum inside of mature basidiocarps, dark brown to black when dried, very rarely of orange shades. Sometimes young basidiocarps grow directly on older ones. *Hyphae* clamped, in subiculum heavily gelatinized, 2.5–3.5 μm in diam, rarely swollen, walls 0.3–0.8 μm in width. Subhymenial hyphae densely arranged, 2.5–4.5 μm in diam, walls 0.3–0.8 μm in width. In cupulate basidiocarps sterile areas and margins covered with terminal hyphae 3.5–5 μm in diam, walls 0.5–1.0 μm in width. On borders between fertile and sterile areas branched anastomosing hyphae are often abundant. *Hymenium* consists of basidia and rare cylindrical *hyphidia* with few short branches, 2–3.5 μm in diam, walls width varies from up to 0.8 μm in the base of hyphidium to ∼ 0.3 μm at the top. *Basidia* clavate 17–56 × 2.5–5 μm, with thicker walls in the bases. Sterigmata up to 31(–38) μm in length (n = 87/9). *Basidiospores* slightly curved-cylindrical, aseptate, binucleate, (9.0–)9.7–14.4(–15.6) × (3.0–)3.1–4.9(–5.3) μm, L = 12.1 μm, W = 4.0 μm, Q = 3.0, Q’ = 2.4–4.2 (n = 140/10), walls ∼ 0.2–0.3 μm in width.  

*Habitat and distribution*: Gymnosperm wood (*Picea*, mostly *Pinus*, and unident.); Europe, mostly northern part.  

*Material examined*: **Estonia**, Viljandi co., Põhja-Sakala par., Soomaa National Park, on gymnosperm wood, 16 Sep. 2018, O. Miettinen (OM henceforth) 21763 (TU135048∗), 21768 (TU135049∗). **Finland**, Pohjois-Häme prov., Jyväskylä mun., Vuoritsalo, on *Pinus sylvestris*, 22 Jun. 2008, OM 12740.1 (H6012436∗), on *Pinus* (?), 22 Aug. 2010, OM 14095 (H∗), on *Pinus sylvestris*, 15 Jul. 2017, OM 20996 (H∗), Kivijärvi mun., Viinakangas, on *P. sylvestris,* 31 Aug. 2017, OM 21288 (H∗), 21292 (H∗), Saarijärvi mun., Pyhä-Häkki National Park, on *P. sylvestris*, 24 Jul. 2017, OM 21034 (H∗); Etelä-Häme prov., Urjala mun., Nuutajärvi, on *P. sylvestris*, 6 Aug. 2017, OM 21058 (H∗). **France**, Aveyron dept., on *Pinus*, 17 May 1915, A. Galzin 17717 or Herb. H. Bourdot 19460 (**lectotype** of *Dacrymyces deliquescens var. castaneus*, PC). **Norway**, Innlandet co., Åsnes mun., Vermunden, on *P. sylvestris*, 3 Jun. 1983, A.-E. Torkelsen 27/83 (O160046∗). **Sweden**, Kalmar co., Nybro mun., Rismåla naturreservat, on *Pinus*, 5 Jul. 2017, T. Knutsson (UPS:F-946511∗), Öland, Borgholm mun., Bödakusten västra naturreservat, Byrums Sandvik, on *P. sylvestris*, 2 Oct. 2017, J.C. Zamora (UPS:F-946508∗); Norrbotten co., Övertorneå mun., Luppioberget, on *P. sylvestris*, J.C Zamora & P. Posio (UPS:F-946514∗); Stockholm co., Stockholm, Årsta, on *P. sylvestris*, 10 Sep. 1017, J.C. Zamora & P. Posio (UPS:F-941018∗), Stockholm, on gymnosperm wood, 1893, L. Romell 2461 (**isotype** of *Dacrymyces romellii*, UPS:F-729991); Uppsala co., Uppsala mun., Årby skog, Storvreta, gymnosperm wood, 14 May 1930, S. Lundell 862 (**holotype** of *Dacrymyces punctiformis*, UPS:F-015301∗), Uppsala mun., Vänge, close to the east limit of Fiby Urskog Nature Reserve, on gymnosperm wood, 7 Apr. 2017, J.C. Zamora, S. Ekman, M. Westberg & M. Svensson (UPS:F-941016∗), Tierp mun., forest 5 km NW of Nilsbo, on *P. sylvestris*, 31 Mar. 2017, S. Ryman 9264d (UPS:F-941017∗). **Ukraine**, Zakarpattia reg., Rakhiv dist., Carpathian Biosphere Reserve, near Lysycha alpine meadow, on *Picea abies*, 8 Aug. 2015, O. Akulov AS0078.1 (CWU(MYC), TU135066∗).  

*Notes*: Basidiocarps of *C. tortus* are similar to the ones of *C. neuhoffii*, but generally larger, more light-coloured, occasionally yellowish when fresh or dull orange when dry. In addition, *C. tortus* has a northern distribution and does not produce such easily coalescing groups as *C. neuhoffii* or *C. creber*. The branched hyphidia of *C. tortus* are rare and more robust than of any other gelatinous *Cerinomyces* species. *Cerinomyces lipoferus* is different from *C. tortus* by its in average wider basidiospores, as well as by longer basidia and much higher content of lipid droplets in hyphae. In *C. lipoferus*, single-septate basidiospores are rare but consistently present, whereas in *C. tortus* they occur only in exceptional cases (*e.g.*, in the specimen Miettinen 21768). Other affined taxa, *C. aff. tortus 1* and *2*, possess well-branched hyphidia. In Zamora & Ekman (2020) *C. tortus sensu typi* was referred to as *Dacrymyces tortus s. lat. 3.*  

 When describing *Tremella torta*, Willdenow (1788) mentioned that it is a rare fungus occurring in hedges near Berlin, but he did not elaborate much on morphology. Fries (1828) transferred the species to *Dacrymyces* and specified *Pinus* as the only substrate. He also synonymized *Dacrymyces lacrymalis sensu* Sommerfeld with *D. tortus.* It caused some confusion, since Sommerfeld’s concept of *D. lacrymalis* is a mix of two species: one growing on gymnosperm wood and another one on angiosperm (Sommerfelt 1826). Fries’ herbarium in UPS contains two specimens that both support the current concept of *C. tortus* as a conifers-dwelling species. We could not prove that this material was used for the sanctioning description, and therefore it is unavailable for lectotypification (ICN 2018, Art. 9.4).  

 Later Bourdot (1932) described *Dacrymyces deliquescens var. castaneus* characterized by discoid, short stalked, brown with orange tint basidiocarps, absence of hyphidia, and occasionally 1-septate basidiospores. We think the variety represents a well-developed *C. tortus s.l.* (For information on *D. castaneus* Rabenh. see the next section.) Neuhoff (1934) added superfluous *D. punctiformis* and *D. romellii*. Excellent condition of *D. punctiformis* holotype allowed us to sequence ITS region that, together with the typical morphology, confirmed the type identity to *C. tortus*. We have checked *D. romellii* isotype and found that it is very scanty, contains no spores, and is massively contaminated by intrahymenial *Tremella* species. Curiously enough, Neuhoff in pers. comm. to Kennedy (1957) said that *D. romellii* is a “luxuriant state” of *D. punctiformis*, which is opposite to the state of the types. Basidiocarps and hyphal structure of *D. romellii* are similar to *C. tortus*, though some microstructural differences are present, such as hyphal knots in subiculum and more thick-walled hyphidia. We consider it a synonym of *C. tortus*, assuming morphological peculiarities could be caused by the parasite and the old specimen age, respectively.  

**Fig. 44:**
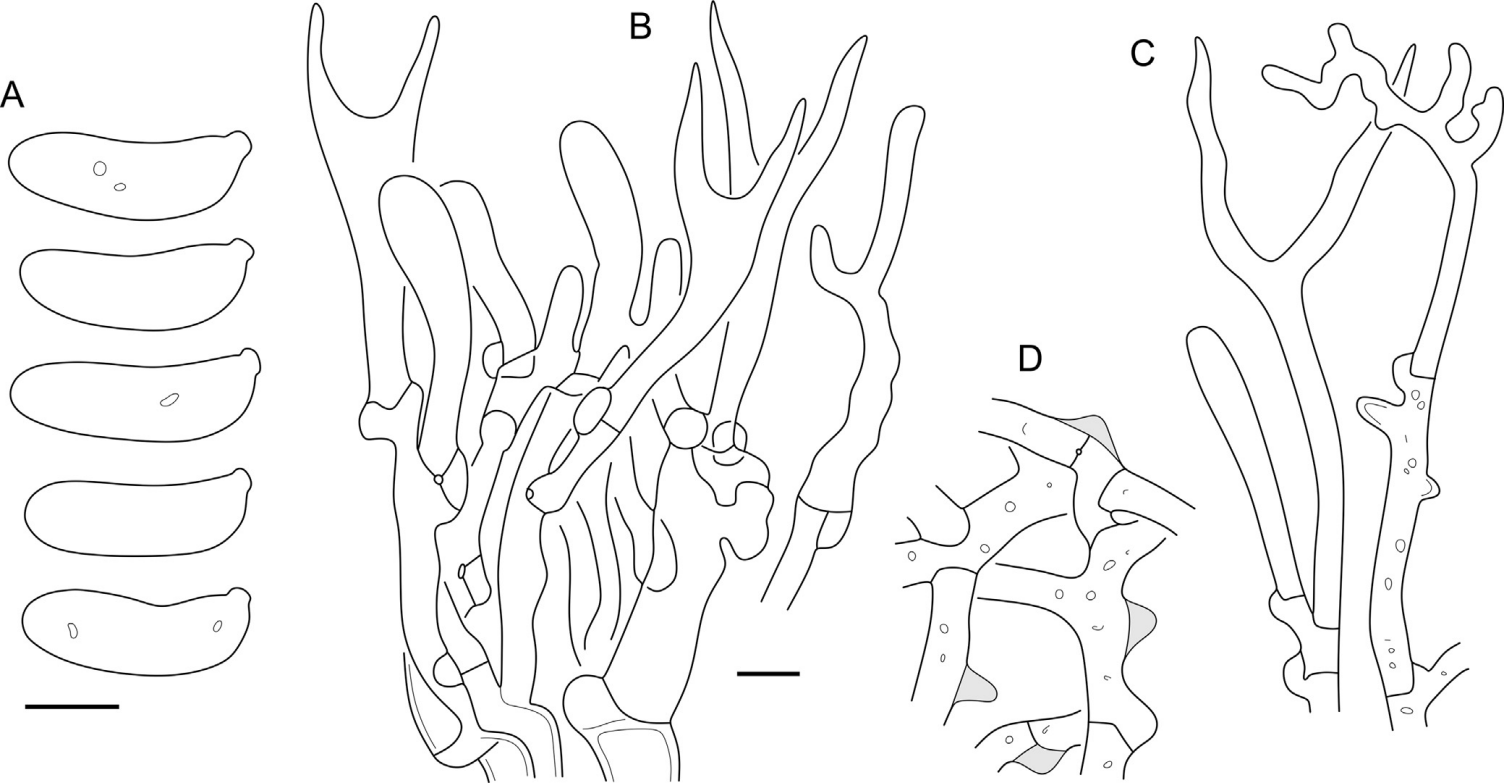
*Cerinomyces aff. tortus 1* micromorphology. **A.** Spores. **B.** Hymenium with short basidia and hyphidium, and subhymenium. **C.** Full-sized basidia and well-developed hyphidium. **D.** Internal hyphae with gelatinous “pouches”. Drawn from UPS:F-946509 (A, B); UPS:F-946504 (C, D). Scale bars = 5 μm.

***Cerinomyces aff. tortus 1***. Fig. 13, Fig. 44.

*Description*: *Basidiocarps* gelatinous, pustulate, often with a central depression when mature, rooted in substrate. Colour from translucent light yellow to brown when fresh, dark brown to black when dried. Basidiocarps up to 0.5(–1) mm in diam, gregarious, close groups can coalesce with the shapes of separate basidiocarps remaining visible. *Hyphae* clamped, in subiculum agglutinated, 2.5–4.5 μm in diam, walls 0.3–0.5 μm in width with uneven gelatinous layer up to 1.5 μm. Subhymenial hyphae in a very thin layer, 2–3(–5) μm in diam, walls 0.3 μm in width. Sterile areas and margins covered with terminal hyphae 3–4 μm in diam, walls 0.3 μm in width, gelatinized. *Hymenium* consists of basidia and branched *hyphidia* up to 70 μm in length, with base 2–3 μm in diam, and apical part 1–2 μm in diam. *Basidia* clavate, 11–32 × 2.5–4.5 μm, with sterigmata up to 24 μm in length (n = 60/2). *Basidiospores* cylindrical, slightly curved, aseptate, binucleate, (8.2–)9.0–12.0(–12.4) × 3.0–4.1(–4.4) μm, L = 10.4 μm, W = 3.6 μm, Q = 2.9, Q’ = 2.3–3.7 (n = 60/2), walls ∼ 0.2 μm in width.  

*Habitat and distribution*: Gymnosperm wood (*Pinus*); Northern Europe.  

*Material examined*: **Finland**, Perä-Pohjanmaa prov., Rovaniemi, on *Pinus sylvestris*, 14 Jul. 2018, J.C. Zamora & P. Posio (UPS:F-946509∗, H6014413). **Sweden**, Kalmar co., Öland, Borgholm mun., Bödakusten västra Nature Reserve, Byrums Sandvik, on *P. sylvestris*, 2 Oct. 2017, J.C. Zamora (UPS:F-946504∗).  

*Notes*: The taxon is similar in basidiocarp appearance and distribution to *C. tortus*, *C. lipoferus*, *C. neuhoffii*, and *C. aff. tortus 2*, though the first two do not have abundant, finely branched dendroid hyphidia. In turn, basidiospores of *C. aff. tortus 1* are averagely smaller than of *C. neuhoffii* and larger than of *C. aff. tortus 2*, though additional collections are needed to confidently delimit the taxa*.* Related Japanese material is discussed under *C. aff. tortus 2.*  

**Fig. 45:**
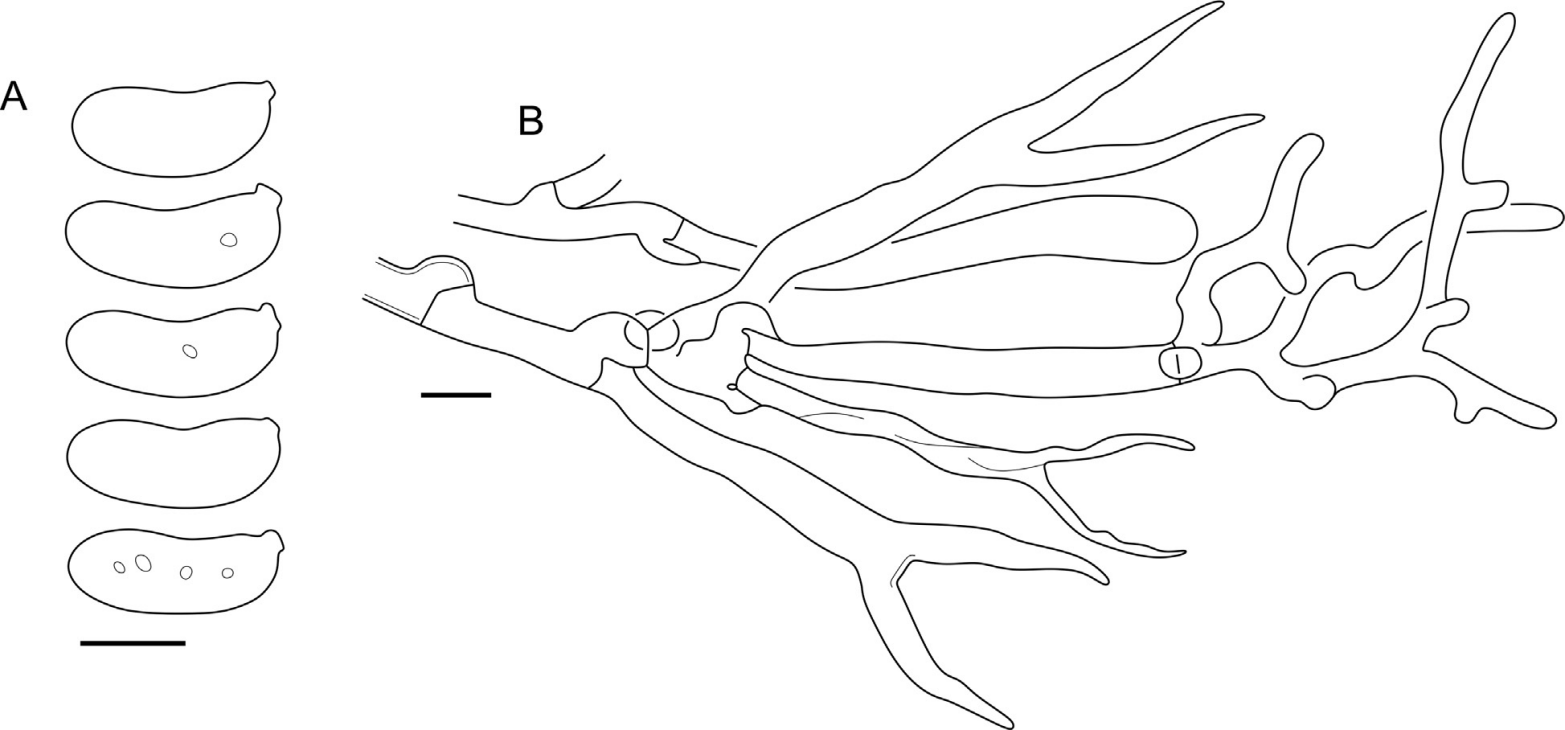
*Cerinomyces aff. tortus 2* micromorphology. **A.** Spores. **B.** Hymenium with hyphidium, and subhymenium. All drawn from UPS:F-940948. Scale bars = 5 μm.

***Cerinomyces aff. tortus 2***. Fig. 13, Fig. 45.

*Description*: *Basidiocarps* gelatinous, pustulate, often centrally depressed, later slightly cerebriform, rooted in substrate. Fresh basidiocarps of light yellow to brown colour, becoming dark brown to black when dried. Basidiocarps up to 1.5 mm in diam, gregarious, coalescing when close, with the outlines of separate basidiocarps remaining visible. *Hyphae* clamped, in subiculum 2.5–4 μm in diam, walls 0.4–0.7(–1.0) μm in width, gelatinized; in subhymenium 2–3(–5) μm in diam, walls 0.3–0.5 μm in width. Margins covered with slightly swollen hyphae 2–3.5 μm in diam, apical parts slightly attenuated, and walls thickened. *Hymenium* includes branched *hyphidia* up to 70 μm in total length, with thickened base 2–3 μm in diam, and apical part 1.5–2 μm in diam. *Basidia* clavate 21–40 × 2.5–4 μm, with sterigmata up to 31 μm in length (n = 30/1). *Basidiospores* slightly curved-cylindrical, 0(–1)-septate, binucleate, 7.8–10.7 × 3.3–4.2(–4.4) μm, L = 9.4 μm, W = 3.8 μm, Q = 2.4, Q’ = 1.9–3.0 (n = 38/1), walls ∼ 0.2 μm in width.  

*Habitat and distribution*: Gymnosperm wood (*Pinus*); Northern Europe.  

*Material examined*: **Norway**, Hedmark co., Kongsvinger mun., Kubergskogen, on *Pinus sylvestris* (?), 6 Oct. 2018, J.C. Zamora & P. Posio (UPS:F-940948∗, H7009043).  

*Notes*: The taxon highly resembles *C. tortus* and *C. lipoferus* in basidiocarp morphology, and *C. neuhoffii* with *C. aff. tortus 1* in presence of well-branched hyphidia. Among them *C. aff. tortus 2* has the on average shortest basidiospores. Zamora & Ekman (2020) cited the taxon as *Dacrymyces tortus s. lat. 2.*  

 Shirouzu *et al.* (2020) added two undescribed taxa related to *C. aff. tortus 1* & *2*. Two collections are similar in the LSU sequences to the Norwegian specimen, but they are likely to represent a separate taxon, cited here as *C. aff. tortus 3* (Japan, Honshu, Chūbu reg., Nagano pref., Sugadairakougen, on *Pinus densiflora*, 12 Jul. 2017, T. Shirouzu HNo.1176 [TNS-F-88757∗], 3 Sep. 2018, HNo.1215 [TNS-F-88780∗]). Another, slightly different, LSU sequence was obtained from a Japanese environmental strain 1611_131A1, but we are not aware of any specimens supporting this taxon.  

**Fig. 46:**
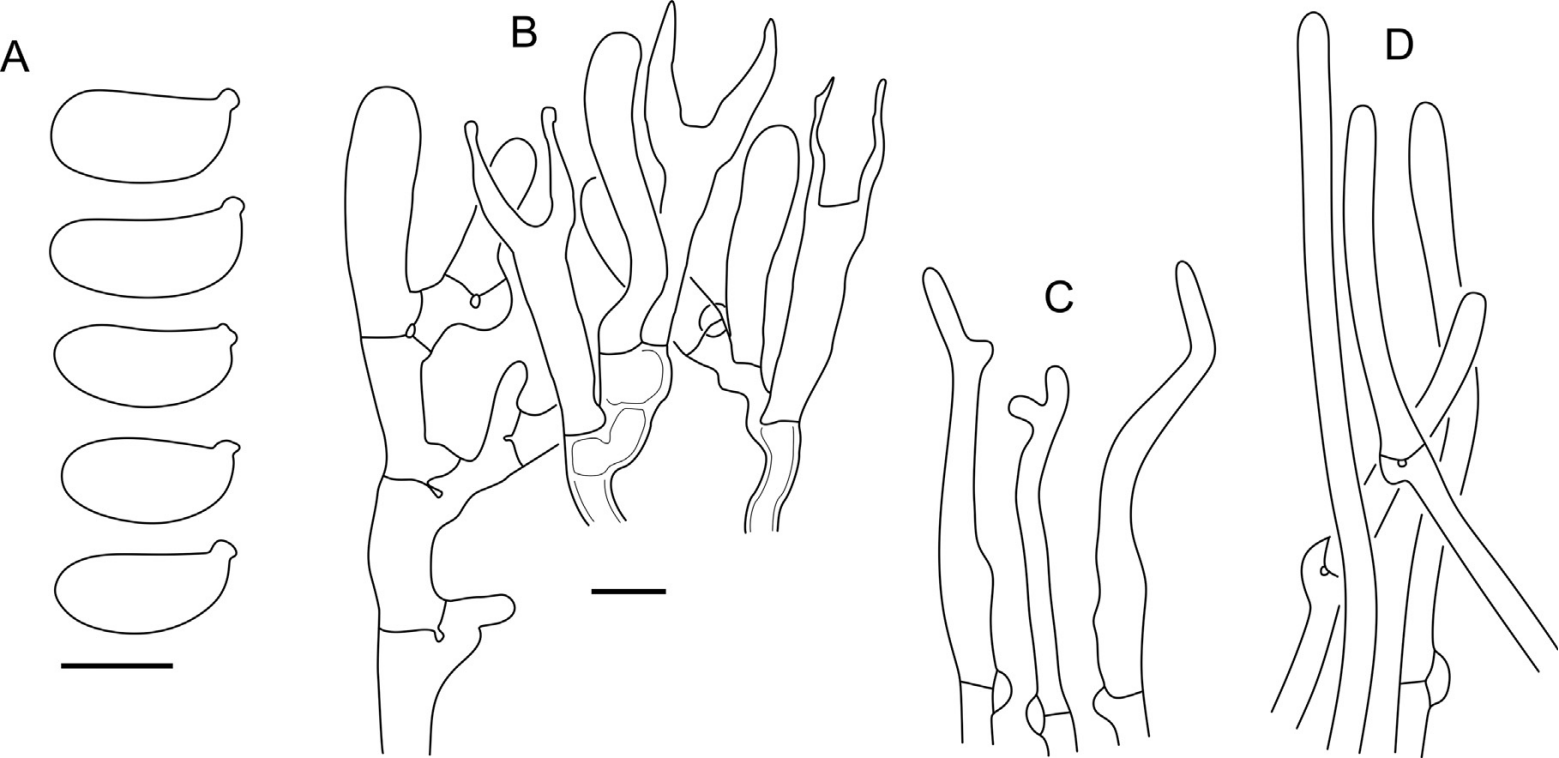
*Cerinomyces tristis* micromorphology. **A.** Spores. **B.** Hymenium and subhymenium. **C.** Hyphidia. **D.** Marginal hyphae. All drawn from holotype, H7009711. Scale bars = 5 μm.

***Cerinomyces tristis*** Miettinen & A. Savchenko, ***sp. nov.*** MycoBank MB 839801. Fig. 10, Fig. 46.

*Typus*: **USA**, New York, Arbutus Lake, on *Picea* (?), 17 Sep. 2013, O. Miettinen 16934 (**holotype** H7009711∗!, **isotype** NY!).  

*Etymology*: tristis (Lat.) — dull coloured; reference to basidiocarp colour.  

*Description*: *Basidiocarps* arid, with relatively thick cottony subiculum, hymenial surface solid and smooth, light ochraceous with whitish margin; covering several cm in the longest dimension. *Hyphae* clamped, in subiculum loose, 2–4 μm in diam, walls 0.2–0.6 μm in width. Subhymenial hyphae of the same type, wall thickness decreases towards hymenium. Margin covered with simple or rarely branched thin-walled hyphae 1.5–2 μm in diam. *Hymenium* consists of basidia and rare *hyphidia* with thickened base up to 3 μm in diam and rarely branched cylindrical apical part up to 2 μm in diam; 15–40 μm in total length, often in groups. *Basidia* clavate, 9–22 × 2.5–5 μm. Sterigmata up to 16 μm in length (n = 45/3). *Basidiospores* long-ellipsoid to slightly curved-cylindrical, aseptate, 6.0–8.9(–9.7) × (2.5–)2.7–4.0(–4.1) μm, L = 7.4 μm, W = 3.3 μm, Q = 2.2, Q’ = 1.6–2.8 (n = 108/3), walls ∼ 0.2 μm in width.  

*Habitat and distribution*: Gymnosperm wood (*Larix*, *Picea* [?], *Pinus*, *Tsuga*, and unident.); North America.  

*Material examined*: **Canada**, British Columbia, Columbia-Shuswap dist., Bugaboo Provincial Park, on gymnosperm wood, 19 Aug. 1982, N. Hallenberg & L. Hallenberg 6649 (GB-0071225∗); Ontario, Nipissing dist., Lake Temagami, Bear Island, on *Pinus*, 14 Aug. 1946, H.S. Jackson (NY:ex TRTS20941). **USA**, Oregon, Cascade Head Expt. Forest, Siuslaw Nat. Forest, on *Larix*, 17 Nov. 1971, M.J. Larsen (CFMR:FP-133094∗); Washington, Callam co., Olympic National Park & Forest, Rugged Ridge, on *Tsuga heterophylla*, 21 Oct. 2014, O. Miettinen 19013 (H∗).  

*Notes*: *Cerinomyces trisis*, together with *C. atrans*, *C. favonius*, and *C. fugax*, belongs to a difficult morphological group of North American conifers-dwelling species. Among them, *C. tristis* is characterized by the shortest basidiospores.  

 A possibly related to *C. tristis* specimen demonstrates some of the lowest Q values in *Cerinomyces*, having basidiospores (5–)5.7–7(–7.2) × (2.9–)3.1–3.9(–4) μm, L = 6.3 μm, W = 3.5 μm, Q = 1.8, basidia 11–19 × 3.5–4 μm, and sterigmata up to 13 μm in length (Canada, British Columbia, Vancouver island, China beach, on *Picea sitchensis*, 20 Sep. 1967, B. Eriksson, J. Eriksson 8401 & J. Ginns [GB-0071226]). Hilar appendix in spores of this specimen is often distanced from the hilum itself. This pattern was also observed in the holotype of *C. nepalensis*, potentially indicating spore immaturity. For the same Canadian specimen, authors of “The *Corticiaceae* of North Europe” reported 3- to 4-sterigmate basidia (Eriksson & Ryvarden 1973). We could not confirm any of these, but 3-sterigmate basidia do occasionally occur in other species of *Cerinomyces* (own observations). Interestingly, the specimen NY:ex TRTS20941 bears a name “*Ceracea temagamensis* Jacks.” — the author correctly assumed it belongs to a new species, but never published the result. Sequence of O. Miettinen 19013 was produced during the revision of this study; it was not used in the analyses but matches other included sequences.  

**Fig. 47:**
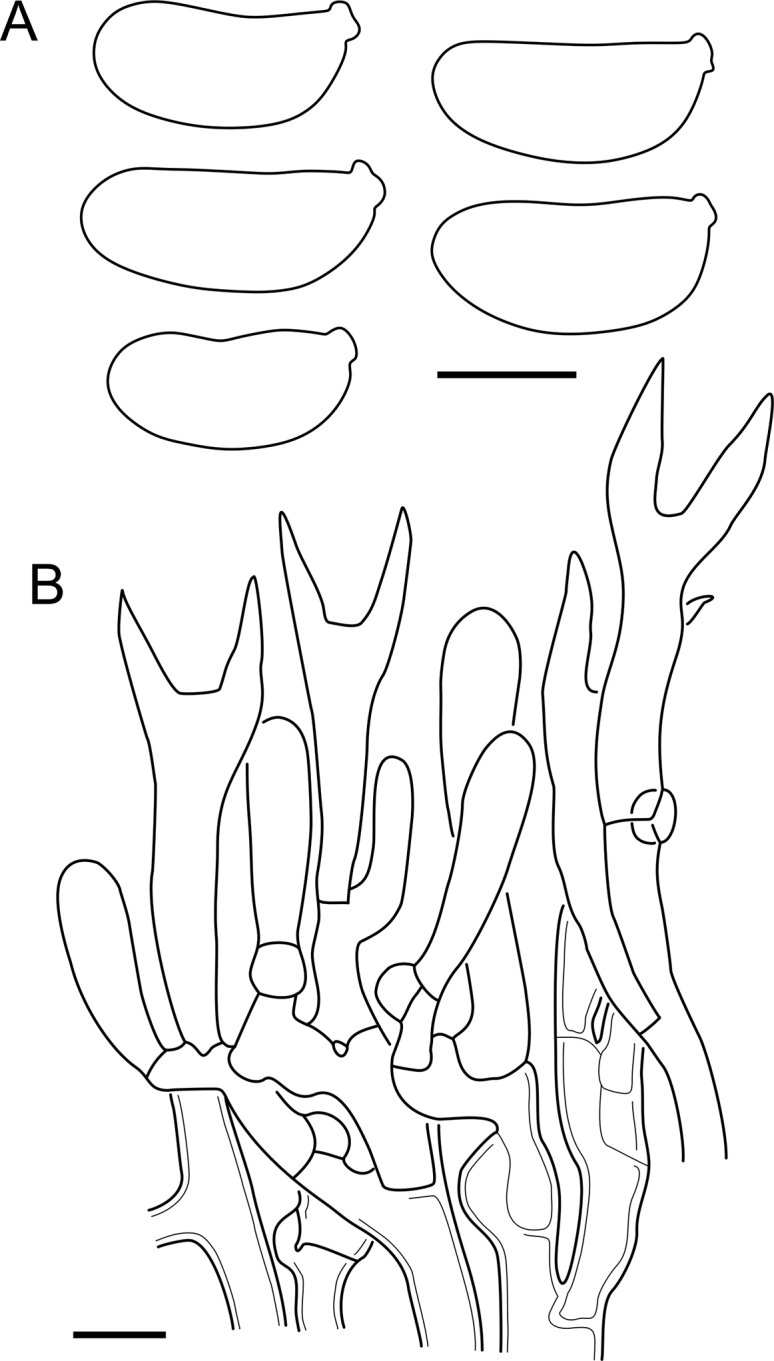
*Cerinomyces verecundus* micromorphology. **A.** Spores. **B.** Hymenium and subhymenium. All drawn from holotype, PDD93708. Scale bars = 5 μm.

***Cerinomyces verecundus*** A. Savchenko, ***sp. nov.*** MycoBank MB 839802. Fig. 10, Fig. 47.

*Typus*: **New Zealand**, Northland, near Whangarei, Glenbervie Pine Forest, on *Pinus radiata*, 8 Aug. 2007, A.J. O’Donnell & B.C. Paulus 4851 (**holotype** PDD93708∗!, **isotype** TU135087!).  

*Etymology*: verecundus (Lat.) — modest, shy; as a reference to bleak basidiocarps.  

*Description*: *Basidiocarps* arid, hymenial surface solid, crustose, dull grey to pale ochraceous, damaged or prominent areas brown; subiculum thin or indistinct; margin either fimbriate and white or absent. *Hyphae* clamped, not differentiated, 2–3 μm in diam, walls 0.3–0.5 μm in width, gelatinized. *Hymenium* consists of broadly clavate *basidia* 12–25 × 2.5–4.5 μm. Sterigmata up to 15(–17) μm in length (n = 30/1). *Basidiospores* cylindrical, slightly curved, aseptate, (7.9–)8.0–10.1(–10.5) × (3.0–)3.1–4.5(–4.8) μm, L = 9.2 μm, W = 3.8 μm, Q = 2.4, Q’ = 2.0–2.9 (n = 30/1), walls ∼ 0.2 μm in width.  

*Habitat and distribution*: Gymnosperm wood (*Pinus*); New Zealand (known only from the type locality).  

*Notes*: Comparing *C. verecundus* to its closest relative, *C. atrans*, the former has larger basidiospores and occurs only in New Zealand. Notably, there are more differences in TEF1-α sequences of the two species than in ITS regions that are nearly identical.  

**Fig. 48:**
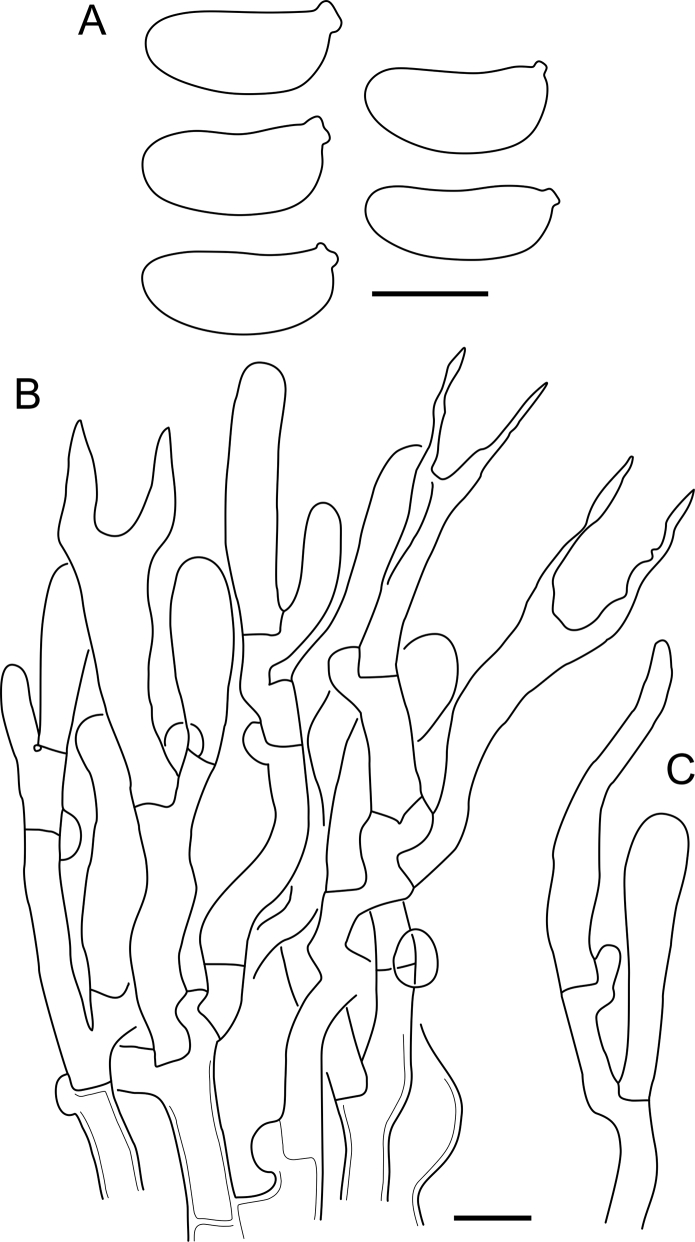
*Cerinomyces volaticus* micromorphology. **A.** Spores. **B.** Hymenium and subhymenium. **C.** Hyphidium and young probasidium. Drawn from holotype, S:F250344 (A); GB:K.-H. Larsson 8688 (B, C). Scale bars = 5 μm.

***Cerinomyces volaticus*** A. Savchenko, V. Malysheva & J.C. Zamora, ***sp. nov.*** MycoBank MB 839803. Fig. 10, Fig. 48.

*Typus*: **Sweden**, Uppsala co., Älvkarleby mun., 6 km NNW from Gårdskär, Långsandsörarna isl., on gymnosperm wood, 30 Aug. 1991, N. Lindqvist 18723 (**holotype** S:F250344∗!).  

*Etymology*: volaticus (Lat.) — unreliable, inconstant; reference to variation in basidiocarp robustness and in spore sizes among specimens.  

*Description*: *Basidiocarps* arid, first arachnoid and remaining so on indistinct white margins; in the middle thicker, smooth and solid, light to rarely dark ochraceous. Subiculum thin, whitish, visible in well-developed areas through cracks. Collapsed old basidiocarps often form a dark brown underlayer. *Hyphae* clamped, subicular hyphae 2–4 μm in diam, walls 0.3–0.5(–1.0) μm in width, slightly gelatinized; in subhymenium 2–3.5 μm in diam, walls 0.2–0.3 μm in width. *Hymenium* contains occasional simple cylindrical or barely branched *hyphidia* with thickened base and thin apical part; up to 40 μm in total length. *Basidia* clavate, 10–26 × 2–5 μm (n = 108/7), with sterigmata up to 17 μm in length. *Basidiospores* cylindrical, slightly curved, aseptate, (6.1–)6.5–9.3(–10.1) × (2.5–)2.6–3.7(–3.9) μm, L = 7.9 μm, W = 3.1 μm, Q = 2.5, Q’ = 2.0–3.4 (n = 178/7), walls ∼ 0.2 μm in width.  

*Habitat and distribution*: Gymnosperm wood (*Picea*, *Pinus*, and unident.); Europe, mostly northern part.  

*Material examined*: **Estonia**, Saare co., Torgu par., [near] Mässa, forest div. block 208, 12 Aug. 1999, E. Larsson 23-99 (GB, TU114612). **France**, Haute-Savoie dept., cirque du Fer-à-Cheval, forest at a foot of "Cornes de Chamois", on *Picea*, 29 Aug. 1997, B. Duhem 3683 (PC0706779∗). **Norway**, Finnmark co., Sør-Varanger mun., Ødevann i Øvre Pasvik National Park, on *Pinus sylvestris*, 10 Aug. 1976, L. Ryvarden 13602 (O101810), 13605 (O160847); Møre og Romsdal co., Aure mun., Skålvassdalen, on *P. sylvestris*, 26 Aug. 2003, F. Oldervik 402.03 (O189348∗); Rogaland co., Hjelmeland mun., Furunesvatnet, on *P. sylvestris*, 15 Oct. 1998, K.-H. Larsson 8688 (GB); Sør-Trøndelag co., Orkdal mun., Grytdalen Nature Reserve, on *Picea abies*, 26 Aug. 1982, L. Ryvarden 20204 (O101813); Vest-Agder co., Sirdal mun., Skotet, on *P. sylvestris*, 17 Sep. 2012, J. T. Klepsland 12-S025 (O:F-247959∗). **Russia**, Arkhangelsk reg., Krasnoborsky dist., Verkhne-Vashkinskiy forest, near Sestra river, *P. sylvestris*, 5 Aug. 2013, V. Kotkova (LE295748∗, TU135064∗); Leningrad reg., Vyborgsky dist., Zapadnyi Beryozovyi island, on *Pic. abies*, 8 Jul. 2004, V. Kotkova (LE242249∗, TU135063∗). **Sweden**, Jönköping co., Värnamo mun., Björs, on *Pic. abies*, 16 Oct. 1960, J. Eriksson, Herb. Erikss. 873 (GB-0071193∗); Västra Götaland co., Alingsås mun., E of Valebråta, 8 Sep. 1969, K. Hjortstam 1926 (GB-0071206∗), Lerum mun., Östad parish, SW of Skäfthult, on *Pic. abies*, 11 Jun. 1970, K. Hjortstam 3277 (GB-0071201), Vårgårda mun., 400 m N of Kampetå, on *Pic. abies*, 19 Aug. 1972, K.-H. Larsson 890 (GB-0071205).  

*Notes*: *Cerinomyces volaticus* seems to be a common species in Northern Europe, and it was also found in France and reported from Germany as *C. pallidus* (Huckfeldt & Hechler 2004). Many of the specimens in European herbaria labelled as *C. crustulinus* belong to this species. It is similar in appearance to *C. borealis*, but can be separated by its shorter and thicker basidiospores, presence of hyphidia, and more robust basidiocarps. Note though, that some specimens show intermediate morphologies and can be difficult in identification. Spore shape and size vary between individuals: for instance, the collection O189348 has more ellipsoid, smaller than usual, and probably immature basidiospores, measuring 5.1–7.8 × 2.4–3.5 μm, Q = 2.3. A specimen presumably belonging to *C. volaticus* was recorded in Italy from *Tamarix gallica* (Losi 2003), but otherwise *C. volaticus* is strictly associated with gymnosperm wood. The most closely related species to *C. volaticus* is the North American *C. fugax*, and the two are hardly distinguishable by morphology.  

**Fig. 49:**
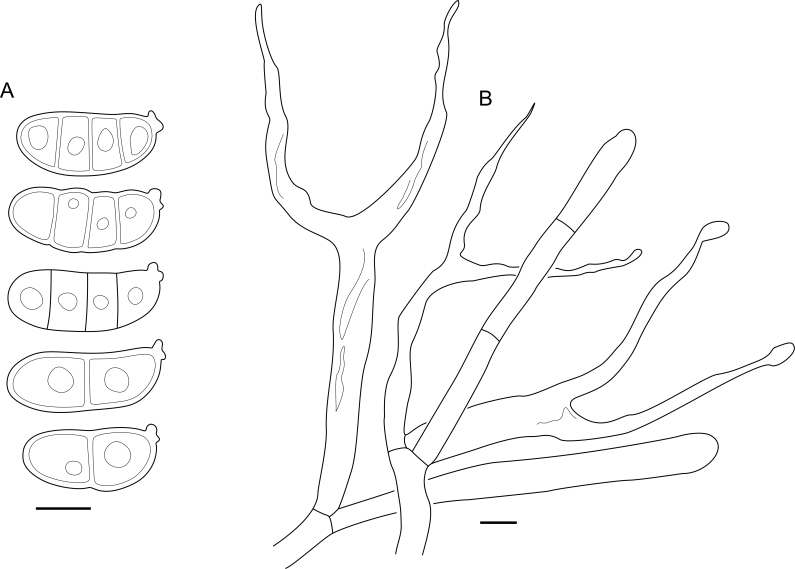
*Dacrymyces burdsallii* micromorphology. **A.** Spores. **B.** Hymenium. All drawn from holotype, CFMR:HHB-6908. Scale bars = 5 μm.

***Dacrymyces burdsallii*** A. Savchenko, ***sp. nov.*** MycoBank MB 839804. Fig. 15, Fig. 49.

*Typus*: **USA**, Florida, Marion co., Ocklawaha river, on *Taxodium distichum*, 3 Aug. 1972, H.H. Burdsall (**holotype** CFMR:HHB-6908∗!).  

*Etymology*: In honour of the North American mycologist Harold H. Burdsall.  

*Description*: *Basidiocarps* appear as patches of cottony white subiculum, later coalescing. Subiculum thick, covered with solid, arid to waxy, light ochraceous to yellowish brown hymenium; margin wide, fimbriate, white. *Hyphal pegs* present in older areas, rare, irregularly scattered, up to 250 μm in height. *Hyphae* without clamps, subicular hyphae 2–4 μm in diam, walls ∼ 0.5 μm in width; in subhymenium of the same diam, walls ∼ 0.3 μm in width; in margin terminal hyphae cylindrical, 2–3 μm in diam, walls 0.3–0.4 μm in width. Hyphae in pegs agglutinated, densely arranged, similar to marginal. Octahedral transparent crystals abundant in subiculum, with the longest edge 15–30 μm and more. Crystal agglomerations visible by the naked eye. Small crystals also occur in margin. *Hymenium* simple, *basidia* clavate, 24–48 × 2.5–6 μm, with sterigmata up to 48 μm in length (n = 30/1). *Basidiospores* cylindrical, slightly curved, 0–3-septate, (11.6–)11.9–15.0 × (4.8–)4.9–6.1(–6.3) μm, L = 13.4 μm, W = 5.3 μm, Q = 2.5, Q’ = 2.1–3.0 (n = 30/1), walls ∼ 0.3–0.6(–1.0) μm in width.  

*Habitat and distribution*: Gymnosperm wood (*Taxodium*); North America.  

*Material examined*: **USA**, Florida, Alachua co., Austin Cory Forest, picnic area, on *Taxodium distichum*, 20 Jul. 1972, H.H. Burdsall (CFMR:HHB-6685).  

*Notes*: Compared to the allied North American species, *D. burdsallii* has smaller microstructures and much less abundant pegs than *D. grandii* — another species that also inhabits gymnosperm wood. At the same time, *D. ceraceus* and *D. sobrius* apparently prefer angiosperm substrates. Aside the intron TTGAGAG present in the end 5.8S, *D. burdsallii* is different from relatives by changes in the ITS, SSU, LSU and RPB2 regions. A non-sequenced specimen CFMR:HHB-6685 possesses swollen up to 6.5 μm cells in subhymenial hyphae, but never as wide as in *D. ceraceus*.  

**Fig. 50:**
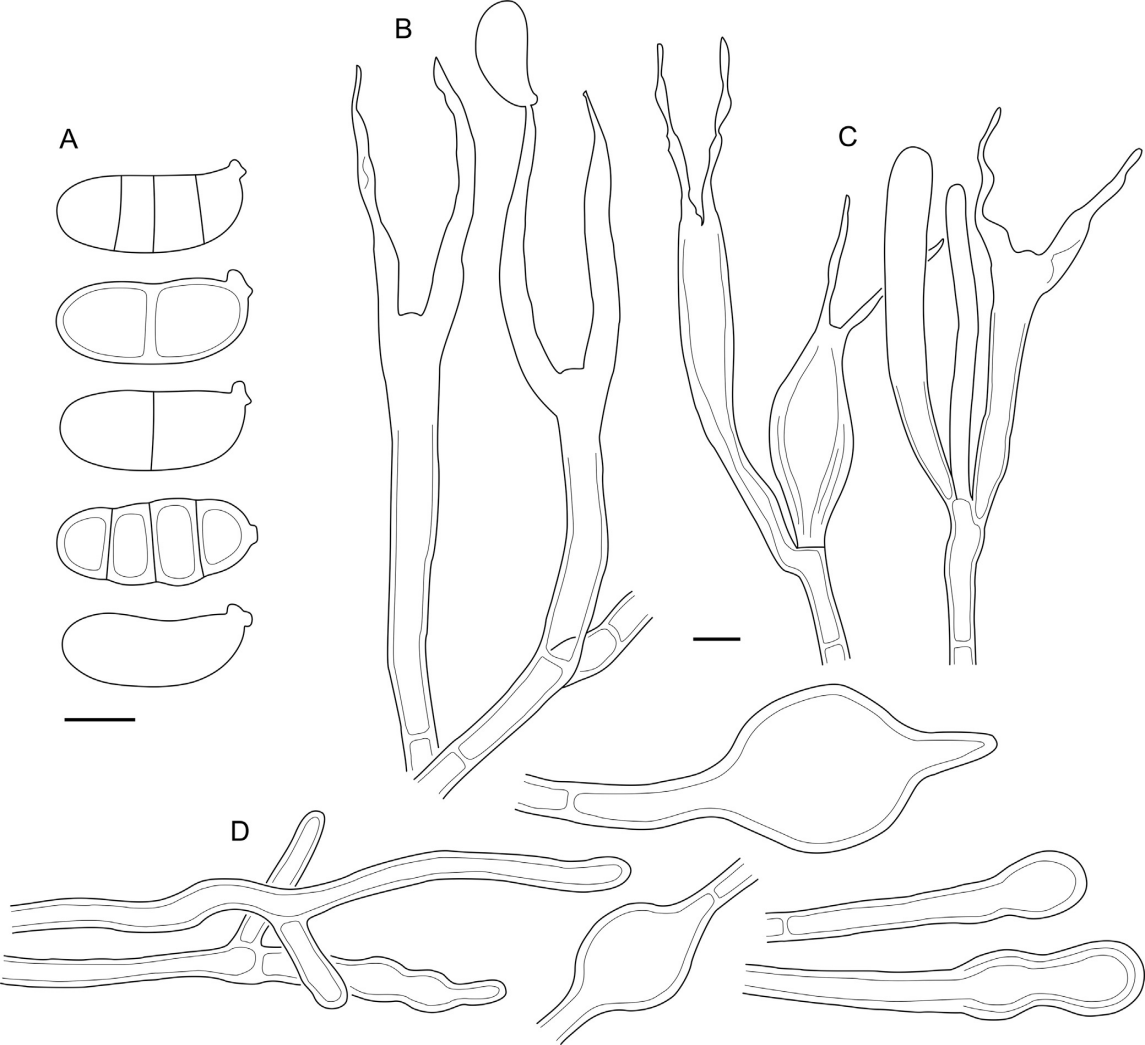
*Dacrymyces ceraceus* micromorphology. **A.** Spores. **B.** Hymenium with full-sized basidia. **C**. Hymenium with short basidia. **D.** Marginal hyphae. All drawn from holotype, CFMR:HHB-8969. Scale bars = 5 μm.

***Dacrymyces ceraceus*** (Ginns) A. Savchenko, ***comb. nov.*** MycoBank MB 839814. Fig. 15, Fig. 50.

*Basionym: Cerinomyces ceraceus* Ginns, Canadian Journal of Botany 60 (4): 519 (1982).  

*Typus*: **USA**, Mississippi, Harrison co., Choctaw Creek Basin, Harrison Expt. Forest, Rd H5, Desoto National Forest, on *Magnolia grandiflora*, 5 Apr. 1976, H.H. Burdsall (**holotype** CFMR:HHB-8969∗!, **isotype** UBC 6160?).  

*Description*: *Basidiocarps* appear as cottony white subicular patches, later covered with solid, ochraceous to yellowish brown hymenium, arid to waxy, with rare scattered *hyphal pegs* up to 200 μm in height. Margin wide, fimbriate, white. *Hyphae* without clamps, subicular hyphae 2–4 μm in diam, walls 0.5–1.0 μm in width; subhymenial hyphae 2–3.5 μm in diam, walls 0.3–0.7 μm in width. Swollen cells up to 20 μm, with thickened walls, occasional to abundant, occurring primarily in subhymenium. Marginal hyphae have cylindrical to clavate ends often with few constrictions, 3–5 μm in diam, with walls 0.5–1.2 μm in width. Margin contains crystal aggregations, marginal and hymenial hyphae often covered with crystal shielding. *Hymenium* densely arranged, *basidia* clavate, 25–46 × 3.5–6.5 μm, with sterigmata up to 27 μm in length (n = 30/1), basidial wall often slightly thickened towards the base. *Basidiospores* slightly curved-cylindrical, 0–3-septate, (11.9–)12.0–16.5(–16.6) × (4.3–)4.4–6.0 μm, L = 13.7 μm, W = 5.1 μm, Q = 2.7, Q’ = 2.2–3.3 (n = 30/1), walls ∼ 0.6–0.8(–1.0) μm in width. Many basidiospores constricted at septa and have swollen walls.  

*Habitat and distribution*: Angiosperm wood (*Magnolia*); North America (known only from the type locality).  

*Notes*: *Dacrymyces ceraceus* is the most similar species to *D. sobrius*, while related *D. burdsallii* and *D. grandii* differ in growing on coniferous substrates*.* Marginal cells, often with moniliform constrictions that were not found in other species, can help in identification. *Dacrymyces ceraceus* is conventionally characterized by the presence of wide swollen compartments in subhymenial hyphae. However, abundance of swollen cells varies highly in preparations even from the same basidiocarp, and sometimes they can be found in other species (*D. burdsallii*, *D. grandinioides*, and *D. sobrius*) so this character should be used with caution. Another character that was utilized in the key by Ginns (1982), thickness of hyphal wall and its level of gelatinization in KOH, have doubtful value as an identification cue. It is often difficult to tell apart the wall itself and gelatinous layer, while the extent of gelatinization varies, depending on time of exposure to water, age of specimens, and nature conditions before collection. At this point, the most reliable way to identify the species is sequencing of ITS marker. As for culture characteristics, Ginns (1982) reported obovoid to cylindrical, 3–4.5 × 2 μm conidia produced in monokaryotic culture.  

 The ITS region of CFMR:HHB-6817 (USA, Florida, Levy co., State Route 24, near Otter creek, on *Taxodium distichum*, 27 Jul. 1972, H.H. Burdsall) aligns well with the *D. ceraceus* ex-type sequence but differs in a few base pairs. Considering low sequence quality, we cannot conclude whether it is an indication of a separate species or intraspecific variation. Notably, the specimen also has slightly moniliform terminal cells.  

**Fig. 51:**
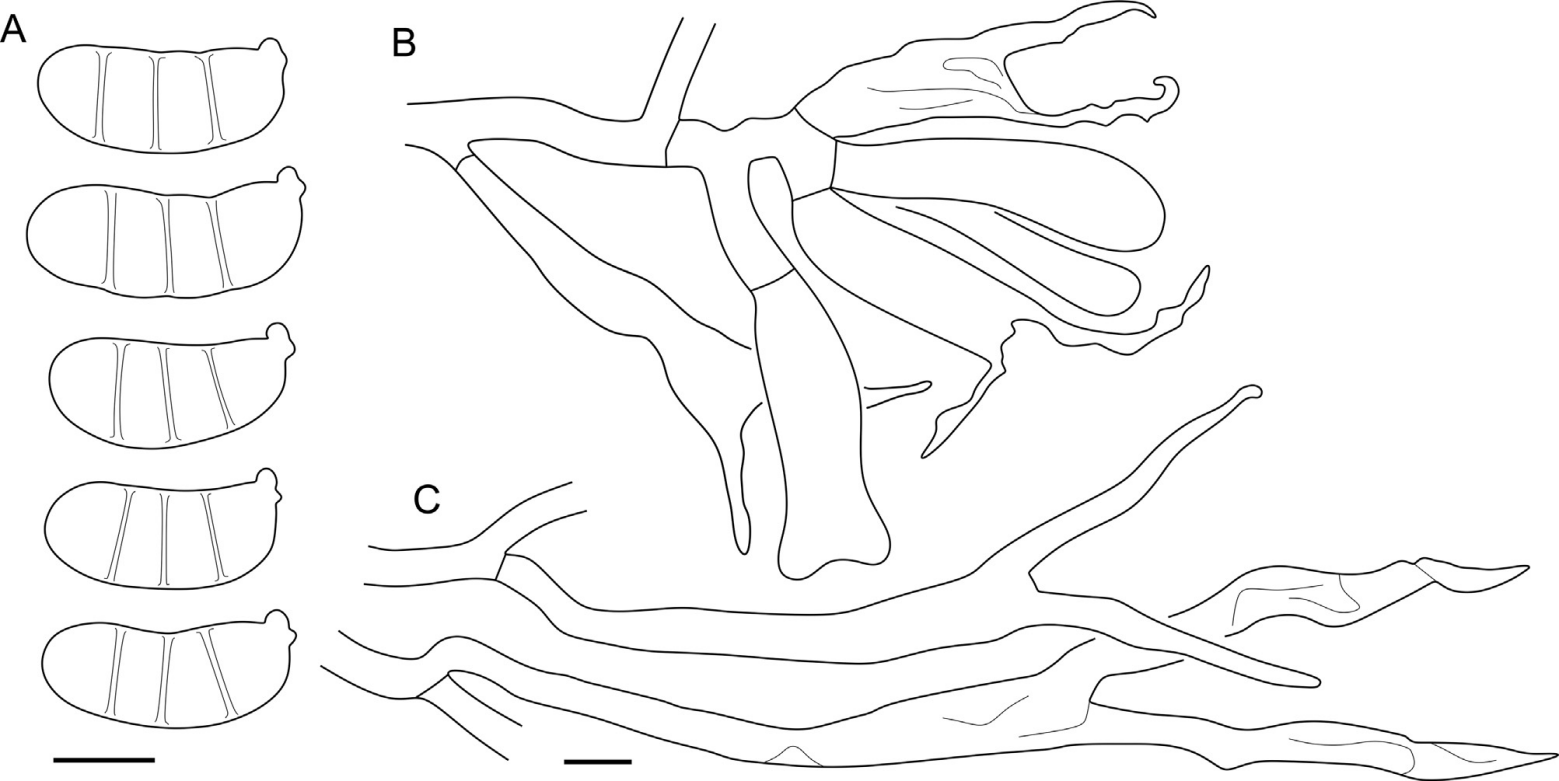
*Dacrymyces cereus* micromorphology. **A.** Spores. **B.** Hymenium with short basidia. **C.** Hymenium with full-sized basidia. Drawn from BPI726063 (A); lectotype, FH00304801 (B, C). Scale bars = 5 μm.

***Dacrymyces cereus*** (Rick) A. Savchenko, ***comb. nov.*** MycoBank MB 839815. Fig. 15, Fig. 51.

*Basionym: Ceracea cerea* Rick, Brotéria, Ciências Naturais 5: 75 (1936).  

*Typus*: **Brazil**, Rio Grande do Sul, São Leopoldo, 1933, J.E. Rick (**lectotype** [designated here] FH00304801!). MycoBank typification MBT10001416.  

*Description*: *Basidiocarps* corticioid, hymenial surface waxy-gelatinous, smooth and solid, mostly light yellow, becoming dark yellow and light brown in the older areas; subiculum thin, cottony; margin indistinct. *Hyphae* without clamps, in subiculum 2–4 μm in diam, with walls 0.4–0.8 μm in width, gelatinized. Subhymenial hyphae 2–3 μm in diam, walls 0.3–0.6 μm in width. *Hymenium* consists of basidia and rare cylindrical *hyphidia*. *Basidia* clavate, 16–45 × 3–7 μm, with sterigmata up to 45 μm in length (n = 70/3). *Basidiospores* slightly curved-cylindrical, 0–3-septate, sometimes constricted at septa, (9.7–)10.4–13.9(–14.0) × (4.0–)4.1–5.9(–6.1) μm, L = 12.0 μm, W = 4.9 μm, Q = 2.5, Q’ = 1.8–3.9 (n = 69/3), walls ∼ 0.2–0.4 μm in width.  

*Habitat and distribution*: Unidentified wood; South America.  

*Material examined*: **Brazil**, Rio Grande do Sul, São Leopoldo, 1930, J.E. Rick (original material, BPI726063); Sergipe, Areia Branca, Parna Serra de Itabaiana, 17 Nov. 2014, R. Chikowski 1167 (URM∗).  

*Notes*: The species is similar to *D. lagerheimii* but has smaller basidiospores and much more delicate basidiocarps. Basidiocarp in the specimen BPI726063 is partially covered with visible by the naked eye large crystal agglomerations of unknown origin. Another South American species that does not demonstrate hyphal pegs, *D. pulchrus*, has substantially longer basidiospores with up to five septa.  

 A specimen without pegs, with similarly small basidiospores 11.7–13.6 × 4.3–5.1 μm, and abundant conidia 3.0–3.3 × 2.2–3.0 μm was collected from Tahiti (Punaauia, on *Hibiscus tiliaceus*, 21 May 1956, L.S. Olive T272 [BPI726045]) and reported as *Arrhytidia lagerheimii* (Olive 1958a), but we refrain from assigning it to *D. cereus* considering scanty material and geographical distance between the finds.  

**Fig. 52:**
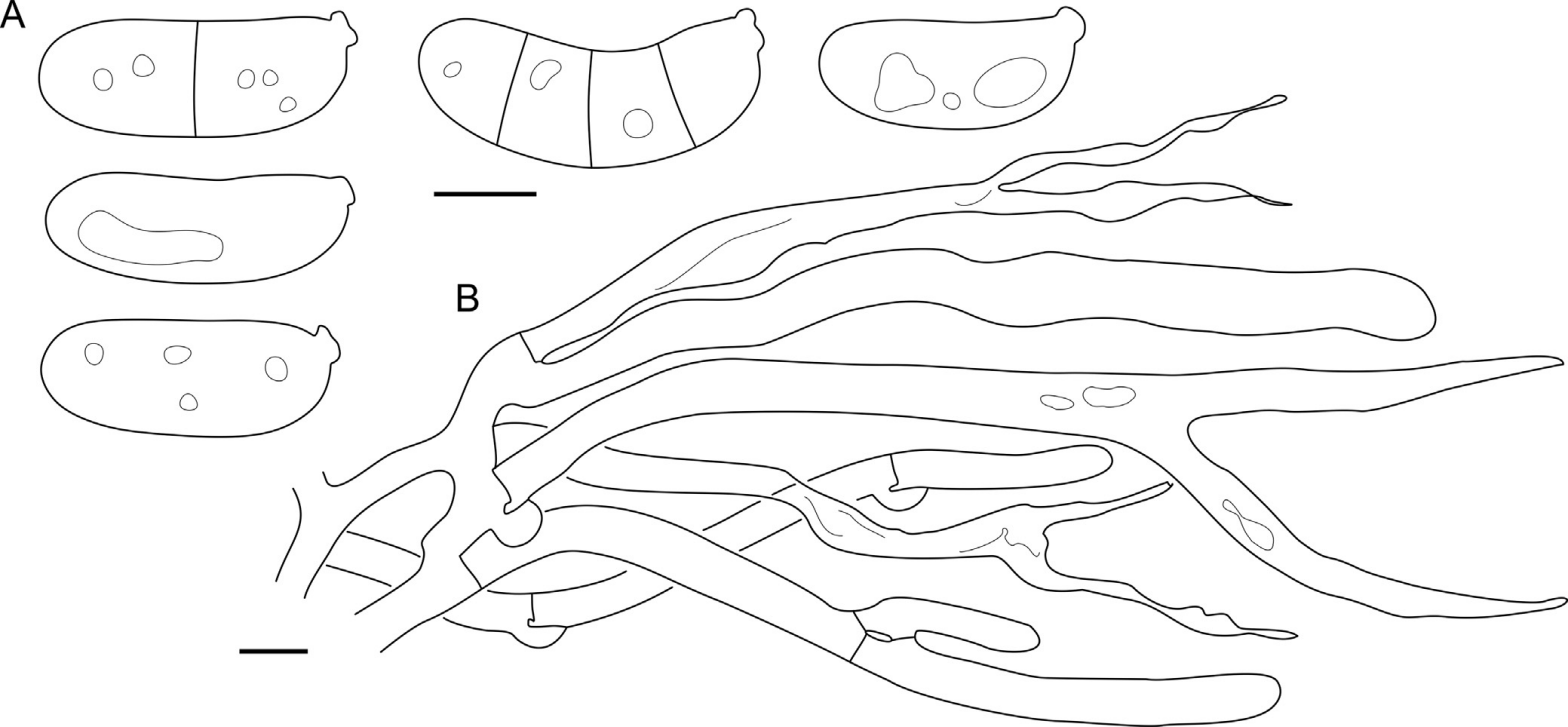
*Dacrymyces confluens* micromorphology. **A.** Spores. **B.** Hymenium with hyphidia, and subhymenium. Drawn from lectotype, H6049587 (A); *Ceracea aureofulva* lectotype, H7009712 (B). Scale bars = 5 μm.

***Dacrymyces confluens*** P. Karst., Fungi Europaei et Extraeuropaei Exsiccati, Centurie XXXVI, no. 3522 (1886). Fig. 14, Fig. 52.

*Synonyms: Dacrymyces paradoxus* P. Karst., Hedwigia 25: 232 (1886).

*Ceracea aureofulva* Bres., Annales Mycologici 4 (1): 39 (1906).

*Cerinomyces aureofulvus* (Bres.) V. Malysheva, Acta Mycologica Warszawa 44 (1): 4 (2009), *nom. illeg*.  

*Typus*: **Finland**, Etelä-Häme prov., Mustiala, on *Pinus sylvestris*, Sep. 1886, P. Karsten, Karsten Herb. No. 1344, in Rabenhorst & Winter, Fungi Eur. et Extraeur. Exsicc., no. 3522 (**lectotype** [designated here] H6049587!). MycoBank typification MBT10001417.  

*Description*: *Basidiocarps* originate as small fimbriate white patches, becoming smooth and solid with waxy-gelatinous hymenial surface, yellow to orange when fresh, ochraceous and brown when dried, with thick white cottony subiculum and margin, up to 1 cm in the longest dimension, of relatively circular form, can coalesce with boundaries of separate basidiocarps remaining visible. *Hyphae* clamped, gelatinized, in subiculum 2.5–4.5(–6) μm in diam, walls ∼ 0.5 μm in width; subhymenial hyphae 2–3.5 μm, walls ∼ 0.3 μm in width; marginal hyphae simple, with cylindrical or slightly clavate endings, 2.5–4.5 μm, walls ∼ 0.3 μm in width. *Hymenium* consists of rare simple cylindrical *hyphidia* up to 80 μm in length and 2–3 μm in diam, and clavate *basidia* 30–71 × 2.5–6 μm, with sterigmata up to 32 μm in length (n = 41/3). *Basidiospores* slightly curved-cylindrical, with lipid droplets, 0–1(–3)-septate, (11.6–)12.1–17.4(–19.7) × (4.0–)4.3–6.8(–7.5) μm, L = 14.3 μm, W = 5.6 μm, Q = 2.6, Q’ = 2.0–3.6 (n = 146/5, 50 measured in 1 % KOH), walls ∼ 0.3 μm in width.  

*Habitat and distribution*: Gymnosperm wood (*Abies*, *Pinus* and unident.); Europe.  

*Material examined*: **Finland**, Etelä-Häme prov., Mustiala, Salois (Saloinen), on gymnosperm wood, Sep. 1886, P. Karsten, Karsten Herb. No. 1407 (probable original material of *D. confluens* H6049575), same loc., on *Pinus*, 1 Nov. 1886, P. Karsten, Karsten Herb. No. 1321 (original material of *D. paradoxus*). **Germany**, Saxony, Königstein, on *Abies*, date unknown, W. Krieger (probable original material of *Ceracea aureofulva*: S:F19431), Elbe Mountains, near Schrammsteine, Oct. 1905, W. Krieger, Fungi Saxon. Exs. 1909 (**lectotype** of *Ceracea aureofulva* [designated here]: H7009712, **isolectotypes** LE22381, S:F19424, S:F19438; MycoBank typification MBT10001418).  

*Notes*: Three-septate basidiospores are extremely rare in specimens from both Finland and Germany; perhaps, septation occurs only immediately before germination.  

 We synonymize *Ceracea aureofulva* to *Dacrymyces confluens* considering their high morphological similarity and European distribution. *Ceracea aureofulva* types have smaller basidiospores than specimens of *D. confluens*, but we expect this to be a particularity of the group, drawing a parallel from *Cerinomyces altaicus*–*D. corticioides* case of similarly wide spore variation. Another synonym, *D. paradoxus*, was formally published in the same year as *D. confluens*, but the latter name has a priority: the 25^th^ issue of Hedwigia, where *D. paradoxus* was first described, also contains a reference to the already published *D. confluens* (Winter 1886). Further synonymization of *D. confluens* and *D. corticioides*, first proposed by von Höhnel (1908), is not carried out considering lack of sequences and gap in the geographic distribution. Specimen S:F19431 of *Ceracea aureofulva* bears an early provisional name *Ce. aurea* provided by G. Bresadola and should not be confused with *Ce. aurea* Rick. *Cerinomyces aureofulvus* is a superfluous name, because in its protologue *D. corticioides* was cited as a synonym. Meanwhile, *Dacrymyces corticioides* is an older name than *Ce. aureofulva*, and therefore ought to have been adopted as a basionym (ICN 2018, Art. 52.1).  

**Fig. 53:**
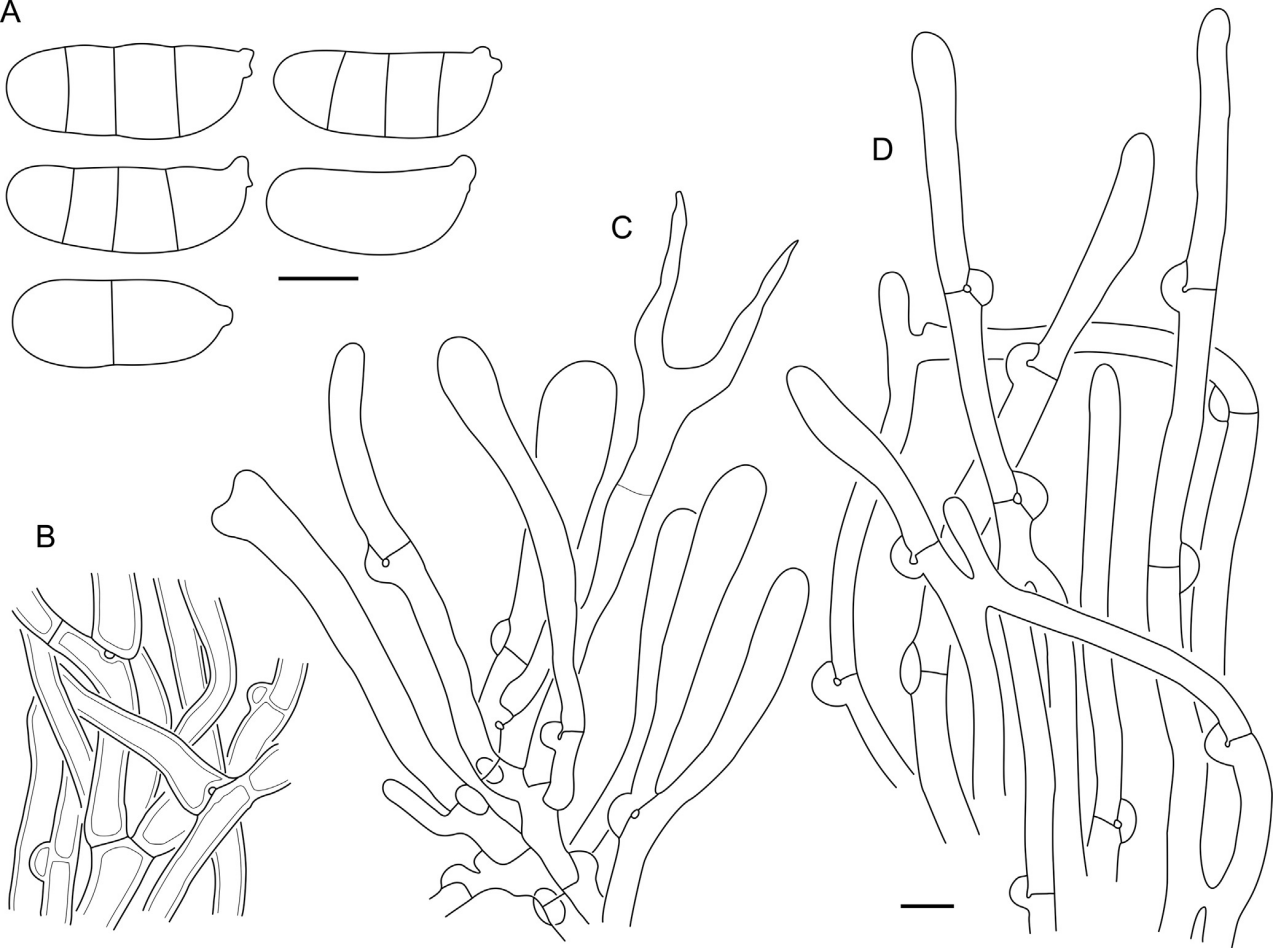
*Dacrymyces corticioides* micromorphology. **A.** Spores. **B.** Subicular hyphae. **C.** Hymenium with hyphidia, and subhymenium. **D.** Marginal hyphae. Drawn from NY00738307 (A, B); syntype, H7009713 (C, D). Scale bars = 5 μm.

***Dacrymyces corticioides*** Ellis & Everh., Journal of Mycology 1 (12): 149 (1885). Fig. 14, Fig. 53.

*Synonyms: Ceracea corticioides* (Ellis & Everh.) Pat., Essai taxonomique sur les familles et les genres des Hyménomycètes: 29 (1900).

*Cerinomyces altaicus* Parmasto, Eesti NSV Teaduste Akadeemia toimetised. Bioloogia: 233 (1961).

*Femsjonia uniseptata* Shirouzu, Phytotaxa 312 (2): 273 (2017).  

*Typus*: **USA**, New Jersey, Gloucester co., Newfield, on *Pinus*, Oct. 1885, J.B. Ellis & B.M. Everhart, Exsiccate: North American fungi. Series II, 1587 (**syntypes** H7009713!, K, NY00738305!, NY00738306!).  

*Description*: *Basidiocarps* originate as circular white sterile patches. Mature basidiocarps waxy-gelatinous when fresh, up to 1 cm in the longest dimension, tend to coalesce, but boundaries between coalesced basidiocarps remain visible. Hymenial surface solid and smooth of yellow to orange colour that bleaks to light or dark ochraceous with time. Subiculum cottony, thick, white; margin fimbriate, white. *Hyphae* clamped, heavily gelatinized, in subiculum 2.5–4.5(–6) μm in diam, walls 0.5–1.0 μm in width; subhymenial hyphae 2–4(–5) μm in diam, walls 0.3–0.5 μm in width; marginal hyphae simple, with cylindrical endings 2–3 μm in diam, walls 0.3–0.5 μm in width, often anastomosing, becoming slightly clavate closer to hymenium. *Hymenium* consists of basidia and rare simple cylindrical *hyphidia* up to 70 μm in length and 2.5–3(–3.5) μm in diam. *Basidia* clavate, 30–56 × 3–6(–7) μm, with sterigmata up to 35 μm in length (n = 40/4). *Basidiospores* slightly curved, cylindrical, 0–3-septate, (11.2–)11.8–16.3(–17.0) × (4.5–)4.9–7.0(–7.9) μm, L = 13.9 μm, W = 5.6 μm, Q = 2.5, Q’ = 1.9–3.3 (n = 81/6; 30 measured in 1 % KOH), walls ∼ 0.2–0.3 μm in width.  

*Habitat and distribution*: Gymnosperm (*Abies*, *Picea*, *Pinus*, and unident.) and perhaps angiosperm wood; East Asia, North America.  

*Material examined*: **Japan**, Honshu, Kantō reg., Ibaraki pref., Sakuragawa, Mt. Tsukuba, on *Pinus densiflora*, 26 Oct. 1985, T. Hosoya (TNS-F-24345). **Russia**, Altai rep., Turochaksky dist., near Telezkoje lake, middle course of the river Oyor, on *P. sibirica* (?), 20 Aug. 1959, E. Parmasto (**holotype** of *Cerinomyces altaicus*, TAAM008610); Khabarovsk reg., Khabarovsk dist., Bolshekhekhtsirsk forest experimental area, Klyutch Levyi (?), on *P. koraiensis*, 1 Sep. 1979, P. Gordienko & E. Parmasto (TAAM102301∗), Nanai dist., Arsenyevo village, on *P. koraiensis*, 19 Sep. 1979, B. Kullman (TAAM120963); Primorsk reg., Chuguyevsky dist., Bulyga-Fadejevo / Sandagou village, Narzan, on *Abies nephrolepis*, 11 Sep. 1975, E. Parmasto (TAAM059772), same village, Kljuch Bolshoi Medvezhi, on *P. koraiensis*, 12 Sep. 1975, E. Parmasto (TAAM059890), Kavalerovsky dist., Gornorechensk / Kentsukhe, on *P. koraiensis*, 9 Oct. 1977, E. Parmasto (TAAM101900, H7019017), Sikhote-Alin Nature Reserve, Ust-Serebryanka river, on *P. koraiensis*, 14 Sep. 1987, E. Parmasto (TAAM150056∗), Terneysky dist., Kabaniy, on angiosperm wood (?), 18 Sep. 1990, I. Parmaso (TAAM126607∗), same dist., Maysa river, on *P. koraiensis*, 10 Sep. 1990, E. Parmasto (TAAM150903). **USA**, Connecticut, Litchfield co., Catlin Woods, E side of Miry Brool, woods of White Memorial Foundation, SW side of Litchfield, 30 Sep. 1978, C.T. Rogerson (NY:as “C. canadensis №1”∗); Georgia, Rabun co., Line branch of Tallulah River, 19 Oct. 1960, C.T. Rogerson 3955 (NY); New Carolina, Macon Co., Highlands Biological Station, on gymnosperm wood, 15 Oct. 1960, C.T. Rogerson 3959 (LSU00173760, NY); New Hampshire, Carroll co., Chocorua, 1906–1907, W.G. Farlow, Exsiccate: Thaxter, Reliquiae Farlowianae 304 (FH00621439, S:F250273), Grafton co., Enfield, shore of lake Mascoma, 24 Sep. 1957, J.A. Stevenson (BPI726048); New Jersey, Gloucester co., Newfield, on *Pinus*, Oct. 1885, J.B. Ellis & B.M. Everhart Exsiccate (?): North American fungi. Series II, 1587 (original material or syntype: NY00738307), same state, no locality, on *Pinus*, Oct. 1892, C.L. Shear 2082 (FH00486013); New York, Albany co., Alcove, on *P. strobus*, Oct. 1892, C.L. Shear, Exsiccate: Shear, New York Fungi 119 (FH00486162, FH00621438), Franklin co., Paul Smith’s, on *Picea*, 8 Oct. 1960, R.L. Gilbertson (CFMR:RLG-2685), Suffolk co., Shirley, Wertheim NWR, on *Pinus rigida*, 28 Nov. 2015, M. Horman (NY02686162∗); Maryland, Prince Georges co., Cornell Rd. at Fomes Rd., Beltsville Expt. Forest, Laurel, 5 Feb. 1968, H. Burdsall & H.H. Burdsall (CFMR:HHB-416).  

*Notes*: *Dacrymyces corticioides* can be identified by conspicuous circular coalescing basidiocarps with white margins that are fimbriate in dry state. Basidiospores are mostly aseptate but become 3-septate at maturity. Majority of the specimens were reported from gymnosperm wood with a single possible exception of TAAM126607. Sequences from TAAM102301, TAAM126607, and TAAM150056 were not used in the analyses, but they are similar to the other included sequences.  

 Basidiocarps of *Femsjonia uniseptata* are much more gelatinized and brightly coloured than most of the studied specimens. However, DNA sequences and micromorphology of *F. uniseptata* are highly similar to *D. corticioides*: Shirouzu *et al.* (2017) reported simple hyphidia, basidia 30–40 × 4 μm, and basidiospores 14–17 × 5–6 μm that agree with our description above. The difference in basidiocarp appearance can be due to the old age of *C. altaicus* and *D. corticioides* collections that have lost characteristic pigmentation over time — a common tendency in dacrymycetes (Zamora & Ekman [2020] and personal observations). On a side note, many of the *D. corticioides* specimens from the 20^th^ century bear a misapplied name *Arrhytidia involuta*.  

**Fig. 54:**
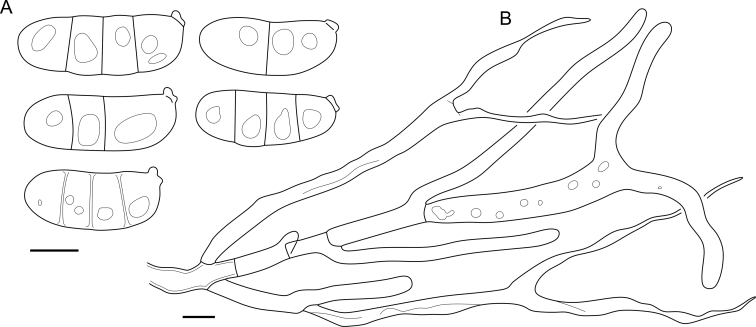
*Dacrymyces grandii* micromorphology. **A.** Spores. **B.** Hymenium. All drawn from holotype, NCSLG21158. Scale bars = 5 μm.

***Dacrymyces grandii*** A. Savchenko & Miettinen, ***sp. nov.*** MycoBank MB 839805. Fig. 15, Fig. 54.

*Typus*: **USA**, North Carolina, Wayne co., cliffs of the Neuse State Park, on *Taxodium distichum*, 16 Jul. 1999, L.F. Grand & C. Vernia (**holotype** NCSLG21158∗!).  

*Etymology*: In honour of the North American mycologist Larry F. Grand.  

*Description*: *Basidiocarps* first appear as patches of cottony white to light ochraceous subiculum, becoming corticioid, with slightly waxy-gelatinous, solid, ochraceous hymenial surface, thick cottony subiculum and distinct fimbriate margins, covering areas up to 5 cm in the longest dimension. Abundant light-coloured *hyphal pegs* cover mature areas, up to 350 μm in height, often with fringy apices. *Hyphae* without clamps, subicular hyphae 2.5–3.5 μm in diam, walls 0.4–0.6 μm in width, sometimes with small crystals. In subhymenium 2–3.5 μm in diam, walls ∼ 0.3 μm in width. Internal hyphae in pegs agglutinated, densely arranged. Terminal hyphae at the top of pegs and in margins simple cylindrical, of the same type as in subhymenium. *Hymenium* consists of clavate *basidia* 20–60 × 3–6 μm, with sterigmata up to 51 μm in length (n = 30/1). *Basidiospores* cylindrical, slightly curved, 0–3-septate, (13.2–)13.6–16.3(–16.4) × (4.8–)5.0–6.2(–6.8) μm, L = 14.9 μm, W = 5.5 μm, Q = 2.7, Q’ = 2.2–3.3 (n = 36/1), walls ∼ 0.3–0.5 μm in width.  

*Habitat and distribution*: Gymnosperm wood (*Taxodium*); North America.  

*Notes*: The species has averagely the largest basidiospores and basidia among its North American relatives, as well as the most abundant and prominent hyphal pegs.  

**Fig. 55:**
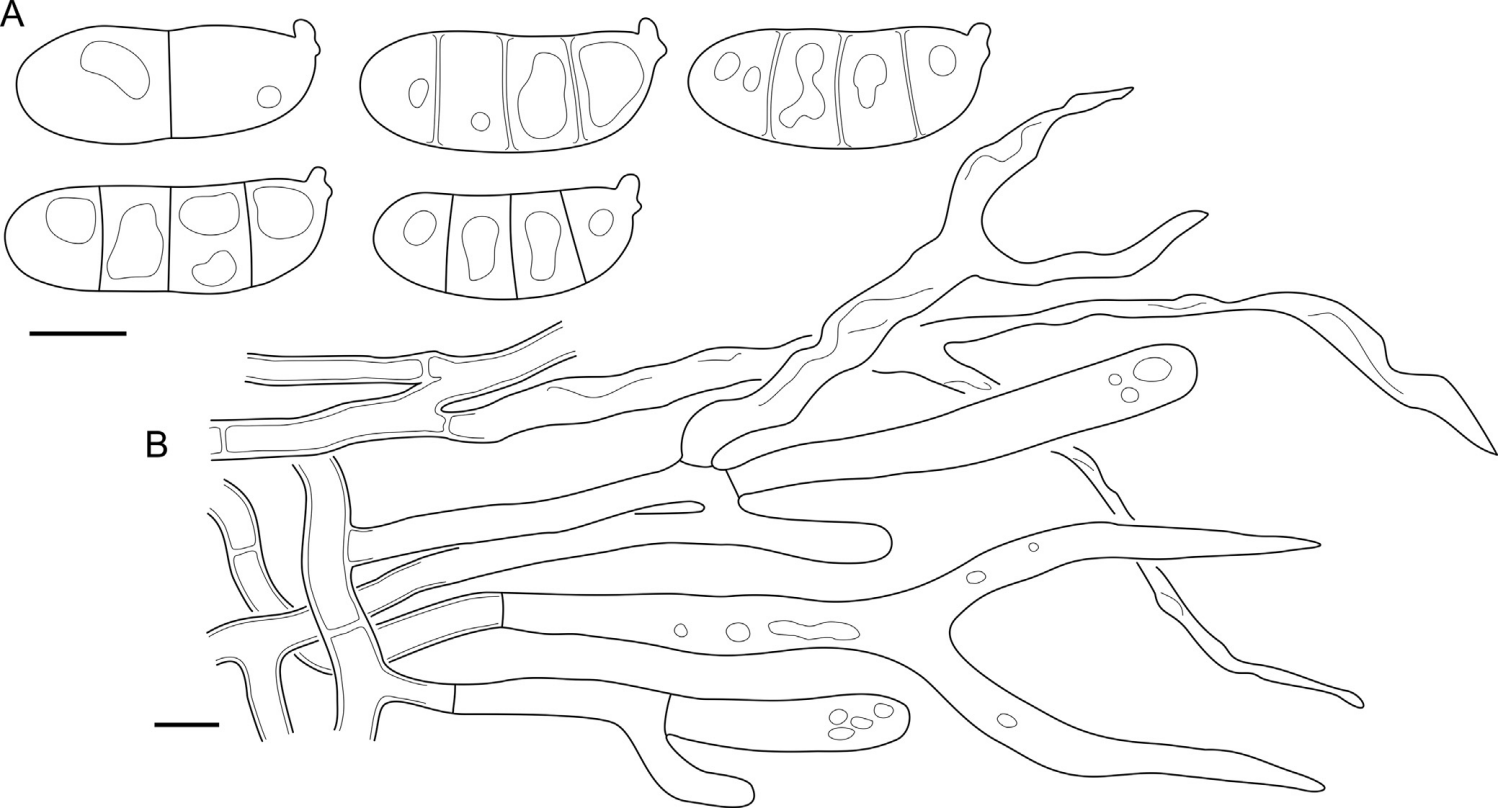
*Dacrymyces grandinioides* micromorphology. **A.** Spores. **B.** Hymenium and subhymenium. All drawn from H7008841. Scale bars = 5 μm.

***Dacrymyces grandinioides*** (McNabb) A. Savchenko, ***comb. nov.*** MycoBank MB 839816. Fig. 15, Fig. 55.

*Basionym: Cerinomyces grandinioides* McNabb, New Zealand Journal of Botany 2: 422 (1964).  

*Typus*: **Kenya**, Bungoma co., mount Elgon, 17 Apr. 1963, P.H. Irwin 539 (**holotype** K(M):237139∗!, **isotype** PDD25551).  

*Description*: *Basidiocarps* appear as small white subicular patches, later coalescing. Well-developed basidiocarps can cover up to 10 cm in the longest dimension, with thick, solid, waxy-gelatinous, yellow to orange hymenial surface, thick cottony subiculum, white fimbriate margins, and abundant light-coloured *hyphal pegs* or denticles up to 300 μm in height, oriented downwards. *Hyphae* without clamps, subicular hyphae 2.5–4.5 μm in diam, with some compartments and hyphal junctions swollen up to 5 μm in diam, walls 0.5–1.0 μm in width; subhymenial hyphae 2–3.5 μm in diam, walls 0.3–0.5 μm in width. Marginal hyphae and hyphae of pegs have cylindrical terminal parts, 2.5–3.5 μm in diam, with walls 0.2–0.4 μm in width. Scattered or aggregated crystals present. *Hymenium* consists of clavate *basidia* 22–48 × 2.5–5 μm, with sterigmata up to 46(–55) μm in length (n = 120/4). *Basidiospores* slightly curved-cylindrical, 0–3-septate, (11.3–)12.0–16.5(–18.3) × (4.3–)4.6–6.5(–6.9) μm, L = 14.3 μm, W = 5.5 μm, Q = 2.6, Q’ = 2.1–3.1 (n = 120/3), walls ∼ 0.2–0.4(–0.5) μm in width.  

*Habitat and distribution*: Angiosperm (*Tabernaemontana* and unident.) and gymnosperm (*Cryptomeria*) wood; East Africa.  

*Material examined*: France, **Réunion**, Saint-Benoît co., Forêt de Bébour, on *Cryptomeria japonica,* 5 May 1985, J. Boidin (LY11601), (LY11604), (LY11615∗, PDD48050); Saint-Paul co., La Petit France, Route de Maïdo, Ravine Baptiste, alt. ca. 1 500 m (Fundort 5), 14 Mar. 1998, G. Langer & E. Langer (KAS:GEL4761). **Kenya**, Taita-Taveta co., Taita Hills, Chawia forest, on angiosperm wood, 27 Nov. 2017, A. Savchenko 171127/1129F (H7008890, EA), on *Tabernaemontana stapfiana*, 27 Nov. 2017, A. Savchenko 171127/1559A (H7008841∗, EA).  

*Notes*: *Dacrymyces grandinioides* and *D. venustus* are morphologically cryptic African species. However, their ITS sequences are distinct enough for easy delimitation: for example, *D. grandinioides* lacks insertion AACGTA in the end of 5.8S region, which is characteristic to the other species. The type specimen of *D. grandinioides* has smaller basidiospores and more subtle basidiocarps compared to the recent collections, which are also of much deeper orange colour. In the type we recorded cylindrical to curved-cylindrical conidia 3.7–4.1 × 1.3–1.8 μm but were unable to confirm their origin.  

**Fig. 56:**
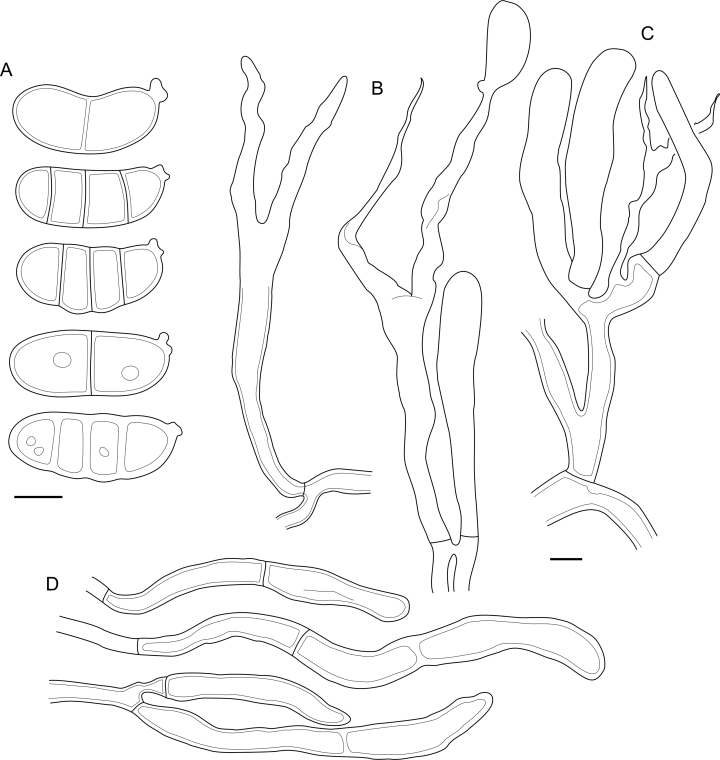
*Dacrymyces lagerheimii* micromorphology. **A.** Spores. **B.** Full-sized basidia. **C.** Hymenium with short basidia, and subhymenium. **D.** Marginal hyphae. Drawn from lectotype, FH00304786 (A, C); FH00304787 (B, D). Scale bars = 5 μm.

***Dacrymyces lagerheimii*** (Pat.) A. Savchenko, ***comb. nov.*** MycoBank MB 839817. Fig. 15, Fig. 56.

*Basionym: Ceracea lagerheimii* Pat., Bulletin de la Société Mycologique de France 9: 141 (1893).

*Synonyms: Arrhytidia lagerheimii* (Pat.) L.S. Olive, Bulletin of the Torrey Botanical Club 85: 104 (1958).

*Cerinomyces lagerheimii* (Pat.) McNabb, New Zealand Journal of Botany 2: 421 (1964).  

*Typus*: **Ecuador**, San Jorge, 1892, N.G. von Lagerheim (**lectotype** FH00304786!).  

*Description*: *Basidiocarps* corticioid, pale ochraceous to yellow and orange, brown in old basidiocarps, hymenial surface smooth and solid, waxy-gelatinous when moist; subiculum thick, cottony, lighter than surface; margin indistinct, fimbriate, white. *Hyphae* without clamps, in subiculum 3–5(–7) μm in diam, with walls 1.0–2.0 μm in width. Subhymenial hyphae 2–4.5 μm in diam, with walls 1.0–1.5 μm in width, but sometimes swollen to 3.5 μm, gelatinized. Marginal hyphae simple cylindrical, similar to subicular. *Hymenium* consists of clavate *basidia* 15–64 × 3–8 μm, with sterigmata up to 67 μm in length (n = 80/4), basidial wall often thickened towards the base. *Basidiospores* slightly curved-cylindrical, 0–3-septate, sometimes constricted at septa, (12.6–)12.8–17.6(–18.5) × (3.5–)5–7.1(–7.6) μm, L = 14.5 μm, W = 6.0 μm, Q = 2.4, Q’ = 1.8–3.7 (n = 125/4), walls ∼ 0.3–0.5(–1.0) μm in width.  

*Habitat and distribution*: Angiosperm wood (*Chusquea* and unident.); South America (known only from the type locality).  

*Material examined*: **Ecuador**, San Jorge, Jul. 1892, N.G. von Lagerheim (**syntypes** FH00304787, FH00304788), same loc., on *Chusquea*, 1892, same collector (**syntype** FH00304790), same loc., no date, same coll. (original material?, S:F19444), no loc., no date, same coll. (original material?, S:F19448).  

*Notes*: *Dacrymyces cereus* and *D. pulchrus* are morphologically related to *D. lagerheimii*, except *D. cereus* has smaller basidiospores and delicate basidiocarps, and *D. pulchrus* exhibit large, 1–3(–5)-septate basidiospores. Unfortunately, the lectotype of *D. lagerheimii* contains old basidiocarps of unrepresentative dull brown colour and deformed microstructures, while better preserved specimens like S:F19444 demonstrate yellow colouration. In the absence of sequenced material, and considering the high morphological variability in the group, we limit the scope of the species exclusively to the Ecuadorian collections.  

 Edward A. Burt labelled but never published “*Ceracea triseptata* Burt, n. sp.”, that was intended to be different from *Ce. lagerheimii* by 3-septate basidiospores — the protologue of *Ce. lagerheimii* mentions only 1-septate spores — and crystalline matter in basidiocarps (Trinidad, 1912–1913, R. Thaxter, FH00304802). It is not known if the specimen stands for a distinct taxon, but small spores and presence of crystals place it closer to *D. cereus*.  

 Ginns (1982) suggested to separate *Cerinomyces lagerheimii* from *C. ceraceus* and *C. grandinioides* on the basis of its smooth surface, gelatinization of hyphae in 2 % KOH, and predominantly 1-septate basidiospores. While the absence of pegs is indeed a key feature, the latter two characters can vary depending on the condition and treatment of a specimen, or development stage, respectively.  

 We did not see conidia in the studied specimens, but Martin (1949) presented illustrations of ellipsoid to cylindrical conidia from the lectotype. Measurements of these conidia (5–7 × 2–3 μm) provided by Ginns (1982) were apparently made from the Martin’s illustration and overestimate the real sizes. McNabb (1964) reported globose to broadly oval conidia 4.5 × 3.5 μm originating from phialide-like conidiophores, but this data most certainly originates from North American specimens that he assigned to the species.  

**Fig. 57:**
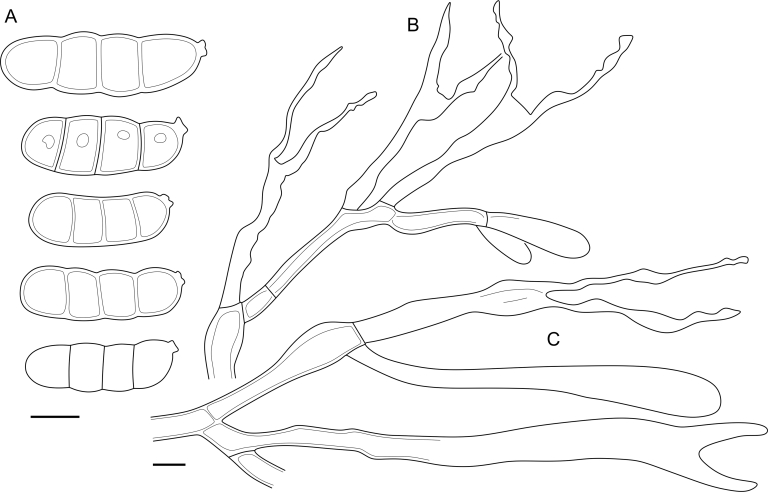
*Dacrymyces pulchrus* micromorphology. **A.** Spores. **B.** Hymenium with short basidia. **C.** Hymenium with full-sized basidia, and subhymenium. All drawn from holotype, LSU00135939. Scale bars = 5 μm.

***Dacrymyces pulchrus*** (Lowy) A. Savchenko, ***comb. nov.*** MycoBank MB 839818. Fig. 15, Fig. 57.

*Basionym: Arrhytidia pulchra* Lowy, Mycologia 64(4): 904 (1972).  

*Typus*: **Argentina**, Buenos Aires prov., El Tigre, 7 Sep. 1968, J.E. Wright T110 (**holotype** LSU00135939!).  

*Description*: *Basidiocarps* corticioid, hymenial surface waxy-gelatinous, smooth and solid, pale ochraceous to yellow and yellow brown; subiculum thin, cottony, white; margin inconstant, fimbriate, white. *Hyphae* without clamps, subicular hyphae loosely interwoven, 3–5.5 μm in diam, with walls 0.5–1.0 μm in width, gelatinized; subhymenial hyphae densely arranged, of the same type. *Hymenium* consists of clavate *basidia* 14–65 × 3.5–8 μm, with sterigmata up to 54 μm in length (n = 30/1), basidial wall often thickened towards the base. *Basidiospores* cylindrical, slightly curved, 0–3(–5)-septate, sometimes constricted at septa, (13.8–)14.1–19.4(–20.6) × (4.5–)4.8–6.4(–6.6) μm, L = 16.3 μm, W = 5.4 μm, Q = 3.0, Q’ = 2.4–3.7 (n = 29/1), walls ∼ 0.3–0.5 μm in width.  

*Habitat and distribution*: Unidentified, probably angiosperm wood; South America (known only from the type locality).  

*Notes*: *Dacrymyces pulchrus* is largely similar to *D. lagerheimii* but differs in presence of 5-septate basidiospores. In contradiction to the protologue, we did not find clamps on hyphae.  

**Fig. 58:**
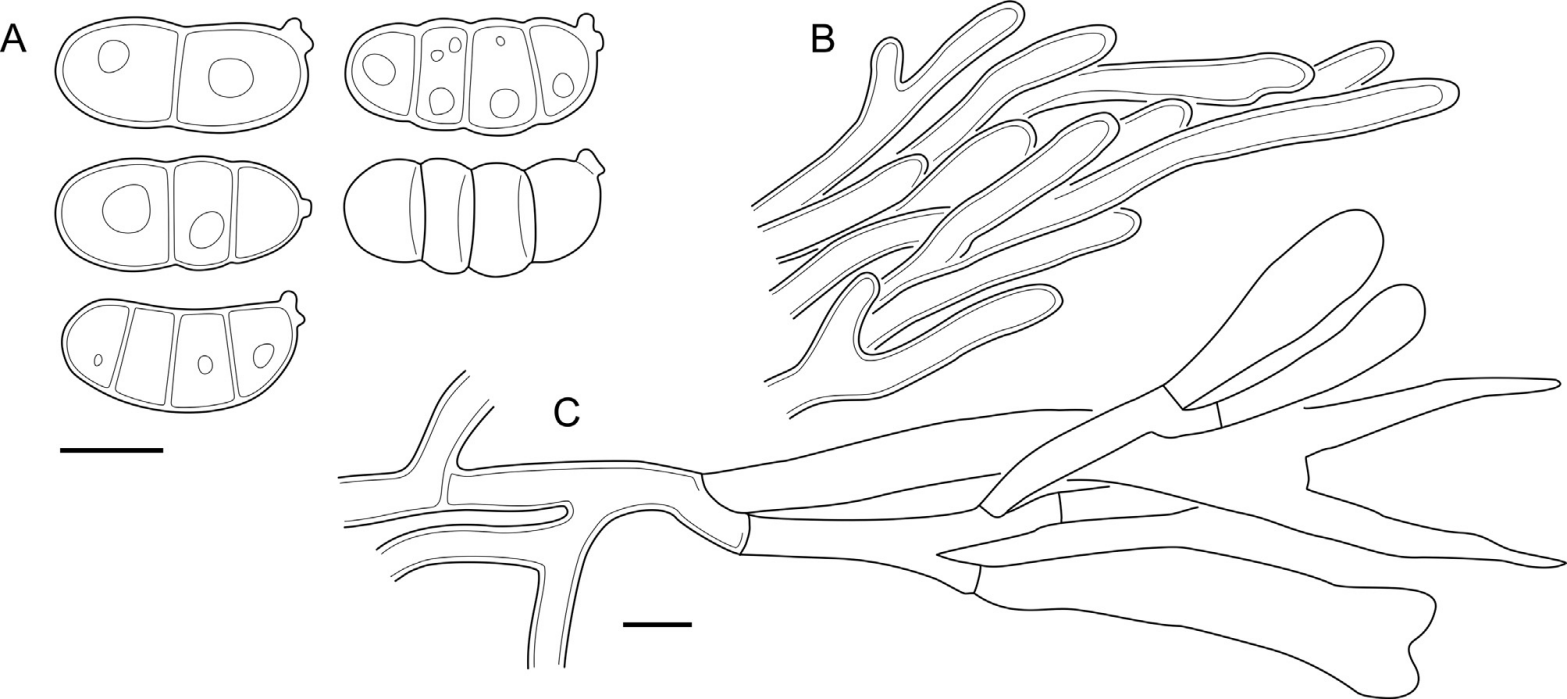
*Dacrymyces sobrius* micromorphology. **A.** Spores. **B.** Apical part of a hyphal peg. **C.** Hymenium and subhymenium. All drawn from holotype, CFMR:RLG-13487. Scale bars = 5 μm.

***Dacrymyces sobrius*** A. Savchenko, ***sp. nov.*** MycoBank MB 839806. Fig. 15, Fig. 58.

*Typus*: **USA**, Louisiana, East Baton Rouge par., east bank of Mississippi river, near Profit Island Road, on *Celtis,* 1 Sep. 1981, R.L. Gilbertson (**holotype** CFMR:RLG-13487∗!).  

*Etymology*: sobrius (Lat.) — temperate, sober; referring to morphological similarity to related species.  

*Description*: *Basidiocarps* corticioid, appear as patches of subiculum, later coalescing, covering areas up to 10 cm in the longest dimension. Thick, cottony, white subiculum covered with solid, waxy-gelatinous, light ochraceous to yellowish brown hymenium; margin fimbriate, white. *Hyphal pegs* present in mature areas, irregularly scattered, up to 250 μm in height. *Hyphae* without clamps, subicular hyphae 2–3 μm in diam, walls 0.4–1.0 μm in width; in subhymenium 2.5–4.5 μm in diam, very rarely swollen, walls of the same width; in margin terminal hyphae cylindrical to clavate, 2–3.5 μm in diam, walls of the same width. Hyphae in pegs agglutinated, densely arranged, similar to marginal. Aggregations of crystals occur in subhymenium. *Hymenium* simple, *basidia* clavate, 21–52 × 3–7 μm, with sterigmata up to 38 μm in length (n = 41/2). *Basidiospores* cylindrical, slightly curved, 0–3-septate, 11.0–17.1 × (4.7–)4.9–6.2(–6.3) μm, L = 13.5 μm, W = 5.4 μm, Q = 2.5, Q’ = 1.9–3.2 (n = 50/2), walls ∼ 0.3–0.7 μm in width.  

*Habitat and distribution*: Angiosperm wood (*Celtis*, *Populus*); North America.  

*Material examined*: **USA**, Kentucky, Hancock co., Hawesville, Wilamette Industries, on *Populus deltoides*, 22 May 1986, K. Nakasone (CFMR:FP-102085∗).  

*Notes*: The species is nearly indistinguishable from *Dacrymyces ceraceus* and *D. burdsallii*, except the latter one was collected from coniferous wood, and the rest from deciduous. We saw that swollen cells of *D. ceraceus* are larger and more abundant than of *D. sobrius*, in which they can be totally absent in some areas of basidiocarp.  

**Fig. 59:**
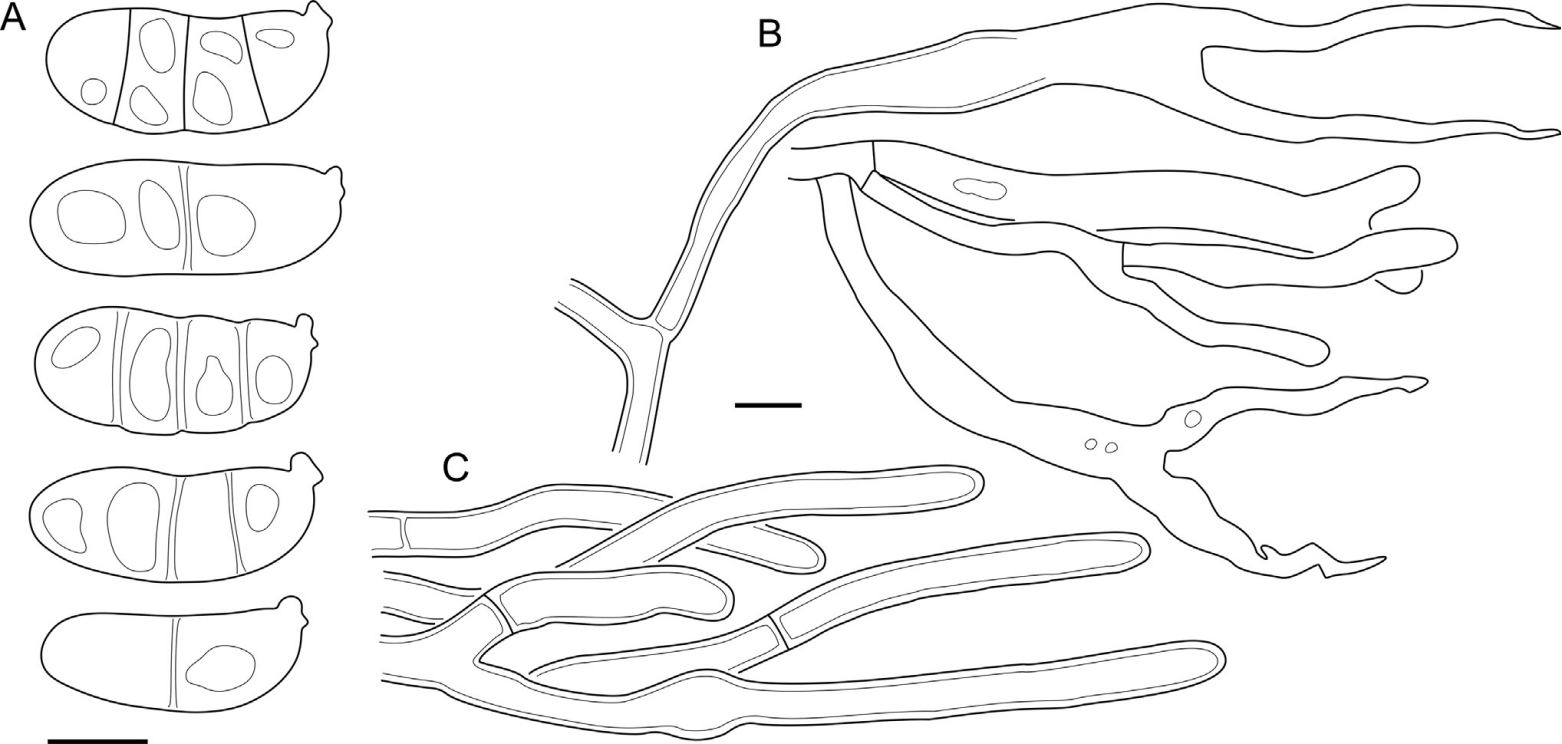
*Dacrymyces venustus* micromorphology. **A.** Spores. **B.** Hymenium with basidia of different length. **C.** Apical part of a hyphal peg. All drawn from holotype, O:Adane 150. Scale bars = 5 μm.

***Dacrymyces venustus*** A. Savchenko, ***sp. nov.*** MycoBank MB 839807. Fig. 15, Fig. 59.

*Typus*: **Ethiopia**, Oromia dist., Arsi, Munessa, on burned *Podocarpus*, May–Aug. 2000, A. Bitew (**holotype** O:Adane 150∗!).  

*Etymology*: venustus (Lat.) — attractive; as a reference to noticeable basidiocarps.  

*Description*: *Basidiocarps* start as arid, circular, coalescing patches. Hymenial surface solid, waxy-gelatinous, yellow, covered with regular *hyphal pegs* ≤ 170 μm in height. Subiculum thin, cottony, white; margins fimbriate, concolourous to subiculum. *Hyphae* without clamps, in subiculum 2.5–4 μm in diam, walls 0.6–0.8 μm in width, masses of octahedral to amorphic crystals present among hyphae. Subhymenial hyphae 1.5–3 μm in diam, walls ∼ 0.3 μm in width. Marginal hyphae simple, cylindrical, 2–3 μm in diam, with walls 0.6–0.8 μm in width. *Hymenium* consists of clavate *basidia* 33–59 × 2.5–6 μm, with sterigmata up to 46 μm in length, basidial wall often thickened towards the base. *Basidiospores* cylindrical, slightly curved, 0–3-septate, often constricted at septa, (12.8–)13.7–16.2(–16.7) × 4.9–6.7(–6.8) μm, L = 14.8 μm, W = 5.5 μm, Q = 2.7, Q’ = 2.1–3.1 (n = 30/1), walls ∼ 0.2–0.4 μm in width.  

*Habitat and distribution*: Gymnosperm wood (*Podocarpus*); Africa (known only from the type locality).  

*Notes*: The species is morphologically similar to *D. grandinioides*, and the easiest way to distinguish them is to compare ITS regions. The type was collected from deeply charred wood.  

**Fig. 60:**
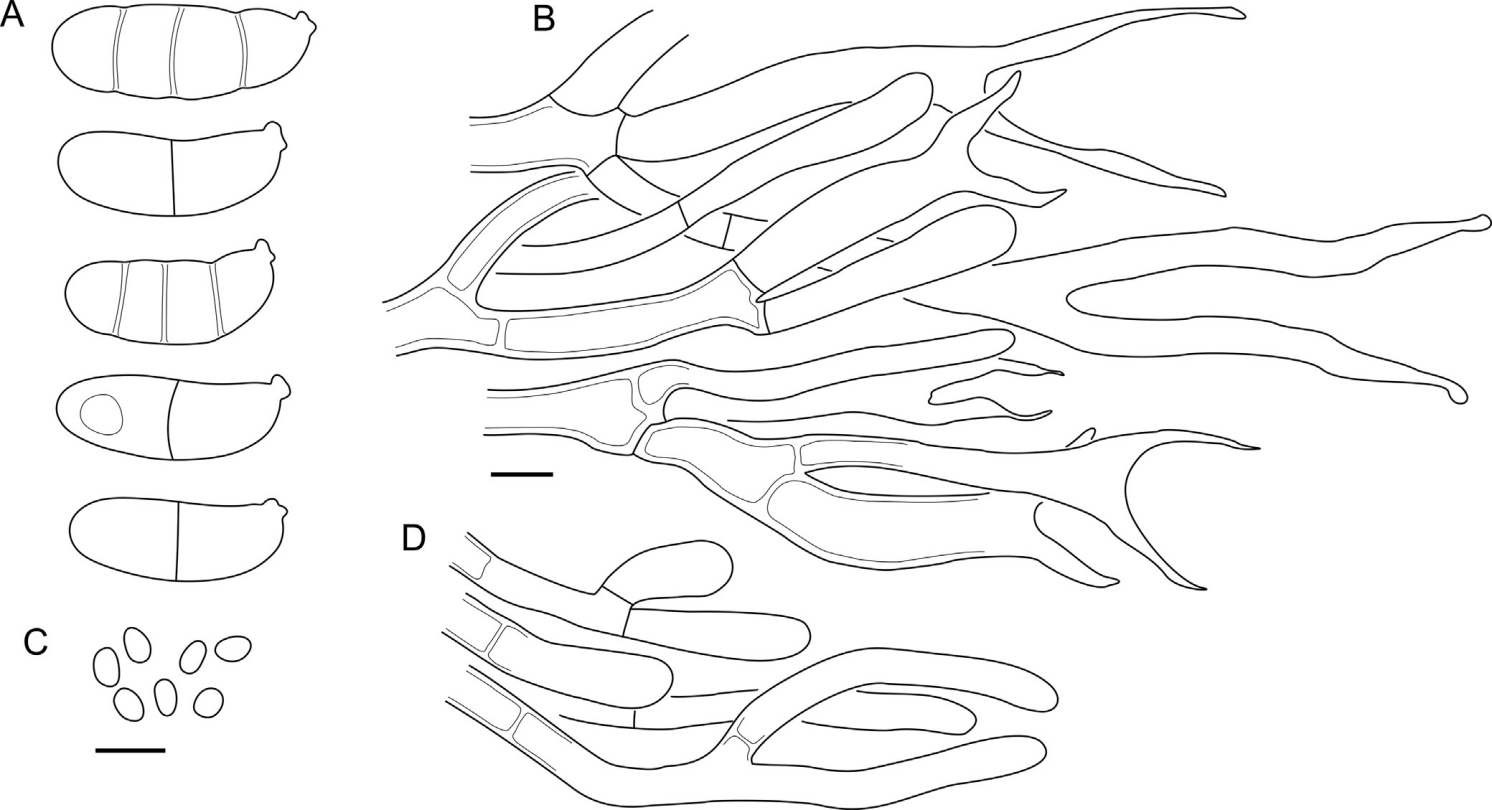
*Dacrymyces aff. venustus 1* micromorphology. **A.** Spores. **B.** Hymenium and subhymenium. **C.** Spore-born conidia. **D.** Marginal hyphae. All drawn from LY7839. Scale bars = 5 μm.

***Dacrymyces aff. venustus 1***. Fig. 15, Fig. 60.

*Description*: *Basidiocarps* appear as patches of thick cottony white subiculum later covered with solid, arid to waxy, light ochraceous to yellow or yellowish brown hymenium. Margin fimbriate, white. *Hyphal pegs* light-coloured, up to 300 μm in height. *Hyphae* without clamps, subicular hyphae 2–4 μm in diam, walls 0.3–1.0 μm in width; in subhymenium 2–4 μm in diam, walls 0.3–1.0 μm in width; in margin terminal hyphae cylindrical to slightly clavate, 2.5–3.5 μm in diam, walls ∼ 0.3 μm in width. Hyphae in pegs agglutinated, densely arranged, similar to marginal. Octahedral transparent crystals abundant in subiculum, with the longest edge up to 20 μm. *Hymenium* densely arranged, consists of clavate *basidia* 17–60 × 3–6 μm with sterigmata up to 34 μm in length (n = 29/1), basidial wall often thickened towards the base. *Basidiospores* cylindrical, slightly curved, 0–3-septate, (13.1–)13.9–18.0(–18.2) × (4.9–)5.0–6.2(–6.3) μm, L = 15.4 μm, W = 5.6 μm, Q = 2.8, Q’ = 2.3–3.2 (n = 32/1), walls ∼ 0.3–0.5 μm in width. Conidia born from basidiospores, subglobose, 2.3–2.7 × 1.3–1.7 μm (n = 6/1).  

*Habitat and distribution*: Unidentified wood; Africa.  

*Material examined*: **Gabon**, Ogooue-Ivindo prov., Makoku, île aux chauve souris, 13 May 1976, J. Boidin (LY7839∗).  

*Notes*: The taxon is almost indistinguishable from *D. venustus* and *D. grandinioides*. Considering this and also scanty molecular data available for analysis, we do not erect a formal species. The studied specimen also includes *Asterostroma* species (*Russulales*).

#### Notes on taxa historically or morphologically related to *Cerinomyces s.l.*

***Ceracea aurea*** Rick, Iheringia, Ser. Bot. 2: 51 (1958).  

Judging from the original description we suggest that this species belongs to *Femsjonia s.l.* or even is not related to dacrymycetes. The protologue says: “Aurea, sicca ochracea, margine albo-lansolo. Sporis grosse obliqueque apiculatis, 12–18 × 10 my. Basidiis 10 my latis, mediis, pedicellatis. Ad corticem. A *C.* [*Arrhytidia*] *flava* videtur diversa.” (Rick 1958). Type not traced.  

***Ceracea brasiliensis*** Rick, Iheringia, Série Botânica 2: 50 (1958).  

The protologue mentions 3-septate ellipsoid basidiospores, allowing to assume that the species is not a member of the *Cerinomycetaceae*. “Effusa, pallida, ceraceo-mollis, sicca lutea. Sporis 6–7 × 4–5 my, in sicco triseptatis, luteolis. Ad lignum mucidum. *Dacryomyces ovisporus* Bref., sporis conveniens, forsan huc ducendus.” (Rick 1958). Type not traced.  

***Ceracea elongata*** Pat., Memoires de L'academie Malgache VI: 9 (1928).  

The species can be related to *Femsjonia*, because it has effused basidiocarps and large 7-septate basidiospores 21–31 × 7–9 μm. Also cited as *Ce. elongata* Pat., Bull. Mus. Hist. nat., Paris: 406 (1928). Type not traced, absent in FH (H. Merchant, 04 Mar. 2020, pers. comm.) and PC (loan requests, visits).  

*Typus*: **Madagascar**, Tananarive, Jan. <1925, M. Waterlot (**holotype**).  

“***Cerinomyces bambusicola***”  

An undescribed taxon proposed by Gminder (2016). The author mentioned brightly orange basidiocarps with meruloid to dentate surface, collected from bamboo and *Hagenia abyssinica* in high altitude (> 2 400 m a.s.l.) forests of Kafa Biosphere Reserve, Ethiopia. We suspect it to belong to the *D. grandinioides* group.  

***Cerinomyces crustulinus var. latisporus*** B. de Vries, Mycotaxon 28 (1): 86 (1987).  

The taxon most probably belongs to the *C. tortus* clade of the *Cerinomycetaceae*; the type material is too scanty to connect it with the existing species*.* Basidiocarps resupinate, gelatinous when water applied, greyish-transparent to light brown and reddish brown; hyphae clamped, 1.4–3(–4) μm in diam; basidia 17–34 × 3.5–8 μm, sterigmata up to 20 μm in length in length (n = 30/1); basidiospores slightly curved-cylindrical, thin-walled, aseptate, (9.0–)9.1–13.0(–15.0) × (3.9–)4.0–5.9 μm, L = 11.2 μm, W = 4.7 μm, Q = 2.4, Q’ = 1.9–3.0 (n = 30/1), walls ∼ 0.2–0.3 μm in width.  

*Typus*: **France**, Côtes-d'Armor dept., Forêt de Beffou, *Taxus*, 24 Jul. 1980, B. de Vries 4103 (**holotype** L0202240!).  

***Dacrymyces adpressus*** Grognot, Plantes Cryptogames–Cellulaires du department de Saone-et-Loire avec des tableaux synoptiques: 200 (1863).  

The species resembles *C. tortus* in general but has 3-septate basidiospores and probably belongs to the *Dacrymycetaceae* as a relative of *D. fennicus*. The studied lectotype has orange basidiocarps; clamped thick-walled gelatinized hyphae 3–4 μm in diam, cylindrical to slightly clavate on margins; basidia 40–75 × 4–6 μm, sterigmata up to 35 μm in length (n = 7/1); basidiospores slightly curved-cylindrical, thin-walled, 0–3-septate, 12.2–16.0 × 4.8–5.8 μm (n = 6/1), walls ∼ 0.2–0.3 μm in width.  

Specimens distributed with Fungi Selecti Gallici exsiccate most likely represent different *Dacrymyces* species and other fungi collected from both angiosperm and gymnosperm wood, and thus should not be automatically treated as isolectotypes.  

*Typus*: **France**, Saône-et-Loire dept., Montjeu, on *Quercus* (?), ≤ 1886, C. Grognot, in Roumeguère, Fungi selecti Gallici exsiccati 2216 (**lectotype** K(M):257626!).  

***Dacrymyces castaneus*** Rabenh., Deutschlands Kryptogamenflora 1: 53 (1844).  

*Dacrymyces castaneus* was described from Italy with a protologue mentioning pustulate gregarious brownish fruitbodies and egg-form spores (Rabenhorst 1844). Concluding from the original description, the species is probably related to *D. ovisporus*. Note that *D. deliquescens var. castaneus* Bourdot is not based on *D. castaneus*: Bourdot (1932) did mention the species in description, but he explicitly pointed out that the two taxa share only the colour. No original material of *D. castaneus* is known.  

***Dacrymyces corticioides var. conigenus*** Ellis & Everh., Journal of Mycology. 2(8):87 (1886).  

The species probably belongs to the *Cerinomycetaceae*, but its precise position cannot be resolved, since the isotypes are extremely scanty and fresh collections are absent. It has a unique substrate among all dacrymycetes, growing on pine cones*.*  

*Typus*: **USA**, New Jersey, Gloucester Co., Newfield, on cones of *Pinus rigida*, 24 May 1886, collector unknown (**isotype** NY00738309!), May 1886, North American Fungi Exsiccate (Second Series) 2028 (**isotype** NY00738308!).  

***Dacrymyces deliquescens var. ardosiacus*** Bourdot & Galzin, Hyménomycètes de France: 68 (1928).  

Concluding from the description, the variety is probably a synonym of *C. aeneus*.  

*Typus*: **France**, Aveyron dept., near St. Leruin, 6 Jan. 1910, A. Galzin 5172 (**lectotype** PC).  

***Dacrymyces deliquescens var. myriadeus*** Bourdot & Galzin, Bulletin Trimestriel de la Société Mycologique de France 25: 33 (1909).  

Following the description, we assume it is a possible synonym of *C. tortus*.  

*Typus*: **France**, Allier dept., Cosne, Forêt de Dreville, 26 Sep. 1906, H. Bourdot 4482 (**lectotype** PC).  

***Dacrymyces deliquescens var. nigricans*** Bourdot & Galzin, Bulletin Trimestriel de la Société Mycologique de France 25: 34 (1909).  

The variety used to be associated with *D. tortus* (Kennedy 1958b), but according to the protologue and revision of McNabb (1973), it possesses at least 3-septate basidiospores and thus belongs to the *Dacrymycetaceae*, probably as a synonym to *D. adpressus*.  

*Typus*: **France**, Allier dept., near St. Priest-en-Murat, 12 Sep. 1906, H. Bourdot 4499 (**lectotype** PC).  

***Dacrymyces enatus var. macrosporus*** L.L. Kenn., Mycologia 50: 902 (1959).  

The variety has prominent branched hyphidia like many taxa in the *Cerinomycetaceae*, but considering regularly 3-septate thick-walled basidiospores, it probably belongs to the *Dacrymycetaceae* as a relative of *D. paraphysatus.* It was first published in a not effective way as *D. enatus var. brunnescens* (Kennedy 1957).  

*Dacrymyces enatus var. macrosporus* has gelatinous basidiocarps dark brown when wet to black when dried, pustulate to pulvinate and cerebriform with short stalks; hyphae clamped, gelatinized; hyphidia finely branched, of 1–2 μm in diam constant through the length, often with several clamps; basidia 31–66 × 3.5–7 μm, sterigmata up to 42 μm in length (n = 30/1); basidiospores slightly curved-cylindrical, 0–3-septate, 13.1–15.1(–15.4) × (5.3–)5.4–6.6(–6.8) μm, L = 14.1 μm, W = 5.9 μm, Q = 2.4, Q’ = 2.2–2.6 (n = 30/1), walls ∼ 0.6 μm in width.  

*Typus*: **Panama**, Canal Zone, Barro Colorado Island, 23 Aug. 1952, G.W. Martin & A.L. Welden 8662 (**holotype** BPI725717! ex IA, **isotypes** NY03684200!, LSU00135945!, TAAM192134!).  

***Dacrymyces fennicus*** Lowy, Sydowia 14: 104 (1960).  

*D. fennicus* is morphologically similar to *D. adpressus* but differs in substrate and distribution; it can also be confused with *C. tortus*, though the latter has aseptate basidiospores. The holotype has light yellow basidiocarps; clamped thin-walled hyphae without heavy gelatinous layer; basidia 35–60 × 3–6 μm, sterigmata up to 34 μm in length (n = 30/1); basidiospores slightly curved-cylindrical, thin-walled (contrary to what is stated in the protologue), 0–3-septate, (11.0–)11.5–14.0(–14.1) × (3.3–)4.0–5.0 μm, L = 12.7 μm, W = 4.5 μm, Q = 2.8, Q’ = 2.4–3.5 (n = 21/1).  

*Typus*: **Finland**, Uusimaa prov., Helsinki, Lauttasaari, on *Pinus*, 22 Jul. 1944, V. Kujala, 25 (**holotype** LSU00135946!).  

*Material examined*: **Finland**, Uusimaa prov., Helsinki, Koskela, on *P. sylvestris*, 18 Feb. 2017, O. Miettinen 20574 (H∗), Veräjämäki, on *Picea abies*, 15 Aug. 2017, O. Miettinen 21174 (H∗).  

***Dacrymyces paraphysatus*** L.S. Olive, Bulletin of the Torrey Botanical Club 85: 106 (1958).  

Basidiocarps of the species are resupinate and gelatinous, of reddish brown colour, highly resembling ones of *C. aeneus*. Basidiospores having three septa *en masse* suggest it belongs to the *Dacrymycetaceae*. Hyphae with clamps, gelatinized; hyphidia well branched, often with few clamps, diam 1–2 μm, constant through the length; basidia 40–78 × 3.5–6 μm, sterigmata up to 41 μm in length (n = 10/1); basidiospores slightly curved-cylindrical, 0–3-septate, 12.8–16.0 × 5.2–6.0 μm, L = 14.4 μm, W = 5.7 μm, Q = 2.5, Q’ = 2.3–3 (n = 10/1), walls ∼ 0.3–1 μm in width.  

*Typus*: **French Polynesia**, Tahiti, Pirae dist., trail to Cascades, on *Citrus limon*, 8 Apr. 1956, L.S. Olive T122 (**holotype** NY00738304!).  

***Dacrymyces pengii*** (B. Liu & L. Fan) A. Savchenko, ***comb. nov.*** MycoBank MB 839819.

*Basionym: Cerinomyces pengii* B. Liu & L. Fan, Journal of Shanxi University, Natural Science Edition: 73 (1988).  

This species is not a *Cerinomyces* and probably belongs to the *D. grandinioides* clade of the *Dacrymycetaceae*: its protologue mentions clampless hyphae, 3-septate basidiospores and resupinate dark yellow basidiocarps. No material except detailed author’s notes was found in the box of the loaned type specimen.  

*Typus*: **China**, Hunan prov., Mengshan mountain, 22 Apr. 1977, Y.B. Peng 1690, ex MHSU720 (**holotype** HMAS85668).  

***Dacrymyces quercicolus*** Sosin, Notuae Systematicae e Sectione Cryptogamica Instituti Botanici Nomine V. I. Komarovii Academiae Scientarum URSS 13: 214 (1960).  

The species might belong to *Cerinomyces*, being potentially a large-spored relative to *C. aeneus*. For the rarity of original publication, we recite the description: “Receptaculum teres, plicatum, sessile, gelatinosum, flavo-brunneum, siccum nigro-brunneum, cartilagineum, 1–3 cm diametro. Basidia clavata, 50–54 × 6–7 μ, sterigmatis elongatis. Sporae amygdaliformes contentu brunneo, episporio laevi, unicellulares, 9–15 × 6–7.5 μ; cystidia clavata 77 × 8.4 μ.”  

The type was originally stored in PWU, but the herbarium could not be reached. It was not found in KW either, to where some of P.E. Sosin’s types were transferred from PWU (M. Zykova, 3 Mar. 2020, pers. comm.).  

*Typus*: **Ukraine**, Vinnytsia reg., Nemyriv dist., Korzhiv, on rotten *Quercus* trunk, 5 Nov. 1939, Balkovsky 708 (**holotype** PWU).  

***Dacrymyces subtristis*** Rick, Iheringia, Série Botânica 2: 52 (1958).  

McNabb (1973) suggested that *D. subtristis* is related to *D. enatus var. macrosporus*, but it has substantially larger 0–3-septate basidiospores 15–25 × 9 μm (Rick 1958). Type not traced.  

***Dacrymyces tristis*** Pat., Énumération Méthodique des Champignons Recueillis à la Guadeloupe et à la Martinique: 11 (1903).  

The species has dark-coloured resupinate to cerebriform basidiocarps but, having clampless septa, should belong to the *Dacrymycetaceae*.  

*Typus*: **Guadeloupe**, Basse-Terre, 1901 (?), A. Duss 514 (**syntype** FH00965285!).  

***Dacryonaema macnabbii*** (D.A. Reid) J.C. Zamora & Ekman, Persoonia 44: 192 (2020).

*Basionym: Dacrymyces macnabbii* D.A. Reid, Transactions of the British Mycological Society 62(3): 456 (1974).  

Basidiocarps generally similar to ones of the *C. tortus* clade members: small, pale ochraceous to brown, pustulate to shallow-cupulate. Clamps often medallion-form, usually present in subhymenium and absent in subiculum, walls of subicular hyphae thick and gelatinized up to 1 μm. Hymenium consists of dendroid hyphidia and clavate basidia 35–56 × 3.5–8.0 μm with sterigmata up to 28 μm in length (n = 17/1). Basidiospores slightly curved-cylindrical, aseptate or exceptionally rarely 1-septate, (11.9–)12.0–16.6(–16.7) × 4.2–5.4(–5.7), L = 14.3 μm, W = 4.9 μm, Q = 2.9, Q’ = 2.4–3.5 (n = 38/2, measured in 1 % KOH), walls ∼ 0.2 μm thick.  

*Typus*: **United Kingdom**, Wester Ross, Kinlochewe, Coille na Glas-Leitire, on *Pinus sylvestris*, 20 Aug. 1963, R.W.G. Dennis (**holotype** K(M):81678∗!).  

*Material examined*: **Sweden**, Jämtland co., Åre mun., ca. 2 km W of Sällsjö, on *P. sylvestris*, 26 Jul. 2018, J.C. Zamora (UPS:F-940995), (UPS:F-940996), (H7009035); Uppsala co., Älvkarleby mun., Långsandsörarna island, on *P. sylvestris*, 17 Apr. 2018, J.C. Zamora, S. Ekman & M. Zuluaga (UPS:F-940954∗, H7009036). For the full list of materials see Zamora & Ekman (2020); cited here specimens were separately studied to produce the description above.  

***Dacryonaema macrosporum*** J.C. Zamora & Ekman, Persoonia 44: 194 (2020).  

One of the isotypes (H6014412) shows a large number of basidiospores distinctly smaller to what was reported by Zamora & Ekman (2020), which can be a sign of spore immaturity. Here we report the data anyway, to claim for attention on this character, which suitability may need to be re-evaluated with further material.  

Basidiocarps of *Da. macrosporum* are similar to *Da. macnabbii* but smaller and darker even in fresh state. Clamps often medallion-form, usually present in subhymenium and absent in subiculum, walls of subicular hyphae thick and gelatinized up to 1 μm. Hymenium includes dendroid hyphidia. Basidia 41–98 × 3.0–8.0 μm, sterigmata up to 26 μm in length (n = 40/1), young probasidia conspicuously clavate. Basidiospores slightly curved-cylindrical to narrowly ellipsoid, aseptate, (10.2–)10.3–16.9(–17.3) × (3.9–)4.0–6.2(–6.4) μm, L = 13.0 μm, W = 5.0 μm, Q = 2.6, Q’ = 2.1–4.0 (n = 33/1), walls ∼ 0.2 μm in width.  

*Typus*: **Finland**, Perä-Pohjanmaa prov., Rovaniemi, on *Pinus sylvestris*, 14 Jul. 2018, J.C. Zamora (**holotype** UPS:F-941001∗!, **isotype** H6014412!). For the full list of materials see Zamora & Ekman (2020); cited here specimens were separately studied to produce the description above.  

***Femsjonia pezizoidea*** (Henn.) McNabb, New Zealand Journal of Botany 3: 226 (1965).

*Basionym: Guepinia pezizoidea* Henn., Hedwigia 43: 197 (1904).

*Synonyms: Ceracea rickii* Bres., Brotéria Série Botânica 5: 9 (1906).

*Ditiola rickii* (Bres.) Bres., Annales Mycologici 18 (1-3): 52 (1920).  

The species belongs to the *Dacrymycetaceae* by virtue of 3-septate basidiospores. The species develops both resupinate and stipitate basidiocarps, as well as intermediate forms; firm-gelatinous, coloured from yellow to brown, ochraceous in old specimens; hyphae clamped, gelatinized; hyphidia long and widely branched, often with few clamps through the length, base ∼ 3 μm in diam, apical part 1–3 μm in diam; basidia 32–65 × 2.5–6 μm, sterigmata up to 40 μm in length (n = 45/4); basidiospores slightly curved-cylindrical, 0–3-septate, (10.0–)10.6–17.6(–18.6) × (3.9–)4.0–6.0(–6.2) μm, L = 13.6 μm, W = 4.7 μm, Q = 2.8, Q’ = 2.1–4.0 (n = 70/3, 8 measured in 1 % KOH), walls ∼ 0.3–1.0 μm in width; conidium straight-cylindrical 3.0 × 1.0.  

*Typus*: **Brazil**, São Paulo, Alto da Serra, 1902, A. Puttemans 761 (**isotype** S:F20949!).  

*Material examined*: **Brazil**, Distrito Federal, Taguatinga dist., Floresta Nacional de Brasília, 12 Jan. 2017, R.L.M. Alvarenga 425 (URM); Rio Grande do Sul, São Leopoldo, on *Bambusa*, 1904, J.E. Rick 9 (**holotype** of *Ceracea rickii*, S:F20231), same loc., 1930, J.E. Rick (FH00304807), same loc., no date (BPI726062), Serra Azul, 1928, J.E. Rick 413 (FH00304805), 495 (FH00304806).  

***Unilacryma bispora*** J.C. Zamora & Ekman, Persoonia 44: 198 (2020).  

Basidiocarps of the species are very small, gelatinous, pustulate to shallow-cupulate, brown. Clamps present, sometimes “unfinished”, rarely absent. Branched hyphidia present. Basidia 56–102 × 5.0–9.0 μm, two sterigmata each up to 37 μm in length (n = 31/2). Basidiospores ellipsoid, with 0–1(–3) early transverse septa and multiple longitudinal and transverse septa at later stages, with long apiculum, (12.1–)12.3–17.4(–18.3) × 6.1–8.7(–8.8) μm, L = 14.7 μm, W = 7.3 μm, Q = 2.0, Q’ = 1.6–2.4 (n = 40/2), walls ∼ 0.3 μm in width. *Unilacryma bispora* was found only in the Northern Europe.  

*Typus*: **Sweden**, Uppsala co., Uppsala mun., Norra Lunsen Nature Reserve, on *Pinus sylvestris*, 19 Nov. 2017, J.C. Zamora (**holotype** UPS:F-941257∗, **isotype** H7009037!).  

*Material examined*: **Sweden**, Jӓmtland co., Åre mun., Mörsil par., Klukshåckren, on *Pinus sylvestris*, 23 Jul. 2018, J.C. Zamora (UPS:F-941264∗), (H7009038). For the full list of materials see Zamora & Ekman (2020); cited here specimens were separately studied to produce the description above.  

***Unilacryma unispora*** (L.S. Olive) Shirouzu, Tokum. & Oberw. Mycologia 105(5): 1120 (2013).

*Basionym: Platygloea unispora* L.S. Olive, The Journal of the Elisha Mitchell Scientific Society 74: 41. (1958).

*Synonyms: Achroomyces unisporus* (L.S. Olive) Wojewoda, Mala Flora Grzybów 2: 205 (1981), not validly published.

*Dacrymyces unisporus* (L.S. Olive) K. Wells, Mycologia 86(1): 31 (1994), not validly published.  

Two of the synonyms above were not published validly (ICN 2018, Art. 6.10) because they were based on an early designation released without a Latin description (Olive 1944), which was fixed later with a valid publication (Olive 1958b). Basidiocarps of *U. unispora* are small, gelatinous, pustulate, of brown colour, generally resemble young basidiocarps of the *C. tortus* clade species, though *U. unispora* can be easily separated from the rest of dacrymycetes by unique unisterigmate basidia. Clamps present, rarely “unfinished” or absent. Basidiospores subglobose to ellipsoid with 0–1(–3) early transverse septa and late longitudinal and additional transverse septa, with long apiculum, 12.0–20.5(–20.8) × (9.5–)10.0–14.8(–18.9) μm, L = 16.9 μm, W = 12.3 μm, Q = 1.4, Q’ = 0.9–1.9 (n = 34/2), walls ∼ 0.5 μm in width. Identification can be problematic, since collapsed basidia sometimes are difficult to recognize. In certain cases, the species may be even confused with immature *Naohidea sebacea*.  

*Typus*: **USA**, North Carolina, Orange co., Chapel Hill, Battle Park, 11 Dec. 1943, L.S. Olive (**holotype** NCU-F-0026842); Georgia, Clarke co., Athens, woods above stadium, on *Juniperus*, 18 Jan. 1946, L.S. Olive Ga77 (**paratype** TENN043282!).  

*Material examined*: **Estonia**, Pärnu co., Lääneranna par., Puhtulaid, on *Pinus sylvestris*, 5 Jun. 2019, A. Savchenko 190605-1240 (TU135055), 190605-1244 (TU135056); Põlva co., Põlva par., vicinities of Aarna, on *Picea abies*, 20 Oct. 2019, A. Savchenko 191020-1400 (TU135075). **Japan**, Honshu, Chūbu reg., Nagano pref., Sugadairakougen, on *Pinus densiflora*, 16 Jun. 2006, T. Shirouzu HNo.332 (TNS-F-15731∗), 2 Jul. 2009, T. Shirouzu HNo.881 (TNS-F-38904∗). For the full list of materials see Zamora & Ekman (2020); cited here specimens were separately studied to produce the description above.

#### Notes on species excluded from dacrymycetes.

***Ceracea subsulphurea*** Rick, Brotéria, Ciências Naturais 5: 74 (1936).  

The species is *Cerocorticium molle s.l.* (*Polyporales*).  

*Material examined*: **Brazil**, **São Leopoldo**, Rio Grande do Sul, 1931, J.E. Rick (original material, FH00304803).  

***Ceracea vernicosa*** Cragin, Bulletin of the Washburn Laboratory of natural History 1 (2): 82 (1885).  

*Synonym: Tremella vernicosa* Cragin, Bulletin of the Washburn Laboratory of natural History 1 (1): 28 (1884), not validly published, *nom. nud*.  

The species is an anamorphic *sporotrichum*-like fungus growing on decayed basidiocarps of a trimitic polypore, probably *Trametes*.  

*Typus*: **USA**, Kansas, Topeka, Shunganunga Creek woods, Feb. 1884, W.F. Cragin, no. 255, 954 (**lectotype** NY01042800!).  

***Cerinomyces megalosporus*** Duhem, Bulletin de la Société Mycologique de France 114 (2): 2 (1998).  

This species does not belong to *Cerinomyces* and should be placed in *Dendrothele s.l.* (*Corticiales*).  

*Typus*: **France**, Haute-Savoie dept., Samoëns, Jardin alpin “La Jaÿsinia”, on *Cornus mas*, 4 Sep. 1995, B. Duhem (BD henceforth) 3472 (**holotype** PC0140726!).  

*Material examined*: **France**, type locality and substrate, 4 Sep. 1995, BD 3473 (PC0706775), 3640 (PC0706776), 7 Sep. 1994, BD 3360 (PC0706777); Provence-Alpes-Côte d'Azur reg., on the side of the D563 road between Seillans and Mons, on *Quercus pubescens*, 17 Dec. 1997, BD 3823 (PC0706778).

## Discussion

Previous phylogenetic studies have settled the boundaries of *Cerinomycetaceae*, separated it from neighbouring families, and shown that *Cerinomyces s.l.* is polyphyletic (Shirouzu *et al.* 2013, Zamora & Ekman 2020). In this work we took the next logical step: a formal reassessment of the family, using both molecular and morphological data on all known taxa. The number of new taxa was unexpectedly high considering how rarely the genus occurs in nature, and how rarely it is collected. Many new species, particularly arid *Cerinomyces*, were recovered by extensive herbarium sampling — this, once again, points to the importance of natural history repositories. Only gelatinous *Cerinomyces* species are sufficiently represented with the recent specimens, while the rest of the genus is covered primarily by material from 1955–1985 collected by experts of “holobasidial” corticioid fungi. In many fungal groups, specimens of this age are considered to be unfit for sequencing, but Sanger sequencing of certain DNA regions (*e.g.*, parts of the nrDNA, such as ITS) of dacrymycetes has a relatively high rate of success.

Such DNA preservation may be facilitated by the ability of dacrymycetes to survive dry periods in a desiccated state and then restore sporulation after humidity increases (Shirouzu *et al.* 2013). We suppose that gelatinization of basidiocarps, a landmark character in the class, is connected to this ability, and gelatinous matter serves as a water storage and protection for hyphal structures. Concluding from the phylogenies, this character was independently lost several times and substituted by corticioid basidiocarps of different degrees of aridness. The ecological background of this change is yet to be understood, but we hypothesize that the transitions can be linked with adjustments to micro-niches. For example, we observe that the gelatinous *Cerinomycetaceae* species grow mostly on fine, hard branches, while corticioids prefer larger, more decayed trunks. In this context, basidiocarps appearing on small, often suspended debris are at risk of rapid drying that interrupts sporulation, and therefore they depend on gelatinization for revival. Meanwhile, species preferring stably wet habitats like half-decomposed logs do not need to prepare for several sporulation episodes. In this case the corticioid morphotype is beneficial, providing a larger hymenial surface and relief from production of voluminous gelatinous matrices.

Compared to the last revision (McNabb 1964), the main changes made to *Cerinomyces s.s.* are: the exclusion of the brightly pigmented *femsjonia*-like corticioids and species lacking hyphal clamps, and the incorporation of *dacrymyces*-like members with gelatinous pale ochraceous and brownish basidiocarps. Following this pattern, a specimen can be rather reliably identified as *Cerinomyces* without a microscope just by the visibly low carotenoid content, regardless of whether it is gelatinous or corticioid. When working with gelatinous species, one should also consider *Unilacrymaceae* and *Dacryonaemataceae*, but basidiocarps in these families are generally much smaller than in *Cerinomyces.* There are a few species in the *Dacrymycetaceae* with brownish basidiocarps (*e.g.*, *Calocera fusca* Lloyd, *Guepiniopsis fulva* P. Delivorias), but they have unusual shapes for the *Cerinomycetaceae*. Lack of colour or semitransparency are not rare in gelatinous dacrymycetes, but this normally appears when the basidiocarps have developed in darkness (Bulat 1954, Vail & Lilly 1968). This is held to be a stable character in only a few species (*e.g.*, *Ditiola haasii* Oberw., *Dacrymyces cylindricus* Shirouzu), which differ from *Cerinomyces* by basidiocarp morphologies. Discoloured morphs in *Cerinomyces* were seen in *C. cokeri* (Fig. 11 C) and *C. tortus*, but can be expected in other species growing in the shade or during the darkest part of the year.

Introducing new taxa to *Cerinomyces s.s.* is relatively straightforward now that the family *Cerinomycetaceae* consists of one genus only. At the same time, transfer of brightly coloured corticioids to the *Dacrymycetaceae* raises the question of how to choose generic names for them. Genus division in this family is clearly a difficult task, complicated by the poly- and paraphyly of the current genera, numerous morphological homoplasies, and lack of molecular data. These uncertainties explain the minimalistic approach to nomenclature we have adopted here. First, for the *Dacrymyces corticioides* and “*Cerinomyces*” *canadensis* lineages we decided to postpone new combinations until the upcoming studies of *Femsjonia*. Second, we included the *D. grandinioides* clade members to the genus *Dacrymyces*, judging from their close position to *Dacrymyces s.s.* in the core *Dacrymycetaceae*. The alternative solution of establishing a new genus is impractical from our viewpoint. Considering the diversity of undescribed taxa in the vicinity of the *D. grandinioides* clade, premature generic splitting may only multiply the problems in this part of the family. Moreover, lack of a common stark trait in *Dacrymyces* should not be a sole reason for splitting. Morphological plasticity even within the well-defined and monophyletic genera of dacrymycetes seems to be the norm rather than the exception. *Unilacryma* has members with 1- and 2-sterigmate basidia, *Dacryonaema* includes taxa with synnematous and pustulate basidiocarps, and *Cerinomyces* now unites arid and gelatinous species. In the same way, *Dacrymyces* can be potentially redefined as both monophyletic and morphologically inclusive genus.

For the convenience of identification, we provided detailed information on all dacrymycetous corticioids from the two families. In certain morphogroups many of the reviewed traits are not diagnostic alone, and instead a combination of characters is needed for identification. This prevented us from extending the dichotomic key to the species level in all cases. As a substitution, identification tables (Table 3, Table 4, Table 5) should be sufficient for most identification purposes. However, there are a few morphological species groups that entail difficulties in identification of young or weathered basidiocarps. These include North American corticioids *C. atrans*, *C. favonius*, *C. fugax*, and *C. tristis*; European gelatinous *C. creber*, *C. lipoferus*, *C. neuhoffii*, and *C. tortus* together with undescribed taxa; and possible, yet uncovered, complexes of European angiosperm-dwelling members related to *C. crustulinus* or *C. aeneus*. The *D. grandinioides* clade is particularly difficult, and in many cases morphological identification within the group seems impossible. While the South American peg-lacking species *D. cereus*, *D. lagerheimii* and *D. pulchrus* can be distinguished using spore size and septation, identification of their peg-bearing relatives is challenging. For both the African complex of *D. grandinioides*, *D. venustus*, and *D. aff. venustus 1*; and North American complex of *D. burdsallii*, *D. grandii*, *D. ceraceus*, and *D. sobrius*, we detected wide variation and overlap in trait measurements. More material is needed to bring higher morphological resolution to the group and provide new ways for identification. For example, many specimens identified in the *D. grandinioides* group demonstrate abundant conidia and conidiogenous structures of largely unexplored diagnostic value. Anamorphic development has a high importance in some species in dacrymycetes, up to apparent loss of teleomorphic stages (members of *Dacryoscyphus*), hence this area should be assessed in taxonomic studies in more detail.

We argue that biogeography is a working determination criterion even for species based on a single specimen or with exceedingly restricted known localities. Species ranges in the studied groups are likely to be confined to continents and biogeographical zones, judging from the overall low number of cosmopolite species in dacrymycetes. Only three species in this study have a multicontinental distribution, found in both East Asia and North America (“*Cerinomyces*” *canadensis*, *C. enatus*, *D. corticioides*). Notably, there are no findings of *Cerinomyces* from the African region, except for *C. albosporus* found at the island of Réunion, and no gelatinous species are recorded in South America. Conversely, corticioid species from the *D. grandinioides* clade were found only in Africa and the Americas, which is unlikely to be caused by sampling density alone.

Tree host specialization in *Cerinomyces s.l.* is generally limited to angiosperm or gymnosperm wood; association with a certain tree species cannot be confidently established. The majority of *Cerinomyces s.s.* prefer coniferous wood, except for *C. aeneus*, *C. crustulinus*, and *C. pallidus* that occur only on angiosperm trees; *C. albosporus* on *Asteraceae* shrubs; *C. enatus* on both angio- and gymnosperms; and a few more species with dubious preferences or unidentified hosts. Shirouzu *et al.* (2014) provided additional insight on host specificity with their wood block decay test, which confirmed the ability of *C. enatus* to decompose both *Pinus densiflora* and *Fagus crenata* (data under *C. canadensis* in Shirouzu *et al.* 2014, but also mind our note under *C. enatus*). At the same time, members of the *D. grandinioides* clade seem to occur on a high variety of substrates including shrubs, bamboos, charred wood, *etc*. Hence, it is difficult to judge host preferences, particularly when only few specimens are available.

Spore measurements have always been a principal component of dacrymycete identification. However, not all of the spore characteristics are equally useful. The maximal number of internal septa in basidiospores should be used with caution: this number fluctuates within one specimen and changes during the basidiocarp lifetime. It is very common that in fresh specimens basidiospores are aseptate while still attached, but after discharge they can massively generate 1–3 septa before germination (Maekawa 1987). This quality led to errors in protologues of *C. albosporus*, *Dacrymyces confluens*, and *D. lagerheimii* for which only one septum was reported, instead of the actual maximal three. In case of uncertainty, we strongly recommend checking the number of septa with germinating spore prints on agar. If this material is not viable, one can look for discharged basidiospores in substrate scrapes close to the basidiocarp.

On a methodological note, we observed that microscopic slide mountants affect the measurements and shapes of microstructures. In this work we used Cotton Blue (CB) in lactic acid, providing sufficient contrast and stability of microstructures. Notably, use of KOH-based solutions in concentrations conventional for studies of aphyllophoroid fungi can lead to unreproducible results, as gelatinous matter that covers hyphae can thicken or even dissolve depending on the concentration of KOH and exposure time. Spore walls easily swell, most dramatically in thick-walled basidiospores of the *Dacrymycetaceae*, adding several micrometers to spore dimensions. On the other hand, preparations with KOH (with or without a stain) help to reveal the organization of densely interwoven hyphae in cases where CB fails. Therefore, for measurements in dacrymycetes we recommend using CB as a primary mountant and a weak solution of KOH (*e.g*., 1 %) where necessary.

Aiming to reduce number of superfluous terms describing fungal morphology, we propose to replace the word “dikaryophysis” (sterile element in hymenium of dacrymycetes) with the more general term “hyphidium”. The term “dikaryoparaphysis” was introduced by Lowy (1954) as a name for sterile hymenial hyphae in all basidiomycetes — in such a manner the author wanted to counterpose it to ascomycetous monokaryotic “paraphysis”. Kennedy (1957) started a tradition to use it in a simplified form: “dikaryophysis”. The term has never been widely used and apparently survived only in dacrymycete-related works, adding unnecessary confusion. Here we report hyphidia only when they are dendroid (the *C. tortus*, *C. enatus* clades), simple with a thickened base and long thin apical part (the *C. pallidus* clade), or simple cylindrical of even width (the *D. corticioides* lineage). We would not use “hyphidia” to describe occasional subhymenium extensions or young cylindrical probasidia that can be found in most dacrymycetes.

Further sequencing of more gene regions in the least-represented groups of the *Cerinomycetaceae* will allow for better-supported phylogenies. Building a robust multigene phylogeny for the *Dacrymycetaceae* will help to refine nomenclature of former *Cerinomyces* species if they need to be treated as separate genera. Continuing collection efforts and herbarium searches in unidentified *Dacrymyces* will certainly uncover more new species. This applies not only to understudied regions, but also to Europe, where knowledge on diversity of gelatinous species is still incomplete. Higher sampling density and established distributions will facilitate recognition of endangered species as potential red lists candidates. Ecological roles and requirements of dacrymycetes are poorly understood and deserve be studied more extensively. For example, several dacrymycetes including *C. tortus* are abundant in the pine wood-decay communities in the Northern boreal zone, but their habitat specialization strategies, if any, are mostly unknown. Environmental DNA obtained from wood will be invaluable in expanding both the taxonomical and ecological knowledge of this group. Finally, genomic and enzymatic studies in *Cerinomycetaceae* and minor families of *Dacrymycetes* can help to reveal origins and evolution of brown wood rot mechanisms.
